# A new generation M^pro^ inhibitor with potent activity against SARS-CoV-2 Omicron variants

**DOI:** 10.1038/s41392-023-01392-w

**Published:** 2023-03-16

**Authors:** Chong Huang, Huiping Shuai, Jingxin Qiao, Yuxin Hou, Rui Zeng, Anjie Xia, Lingwan Xie, Zhen Fang, Yueyue Li, Chaemin Yoon, Qiao Huang, Bingjie Hu, Jing You, Baoxue Quan, Xiu Zhao, Nihong Guo, Shiyu Zhang, Ronggang Ma, Jiahao Zhang, Yifei Wang, Ruicheng Yang, Shanshan Zhang, Jinshan Nan, Haixing Xu, Falu Wang, Jian Lei, Hin Chu, Shengyong Yang

**Affiliations:** 1 State Key Laboratory of Biotherapy and Cancer Center and National Clinical Research Center for Geriatrics, West China Hospital, Sichuan University, Chengdu, Sichuan 610041 China; 2 State Key Laboratory of Emerging Infectious Diseases, Li Ka Shing Faculty of Medicine, The University of Hong Kong, Pokfulam, Hong Kong Special Administrative Region, China; 3 Department of Microbiology, Li Ka Shing Faculty of Medicine, The University of Hong Kong, Pokfulam, Hong Kong Special Administrative Region, China; 4 Key Laboratory of Drug Targeting and Drug Delivery Systems, Ministry of Education, West China School of Pharmacy, Sichuan University, Chengdu, Sichuan 610041 China

**Keywords:** Medicinal chemistry, Industrial microbiology

## Abstract

Emerging SARS-CoV-2 variants, particularly the Omicron variant and its sublineages, continually threaten the global public health. Small molecule antivirals are an effective treatment strategy to fight against the virus. However, the first-generation antivirals either show limited clinical efficacy and/or have some defects in pharmacokinetic (PK) properties. Moreover, with increased use of these drugs across the globe, they face great pressure of drug resistance. We herein present the discovery and characterization of a new generation antiviral drug candidate (SY110), which is a potent and selective inhibitor of SARS-CoV-2 main protease (M^pro^). This compound displayed potent in vitro antiviral activity against not only the predominant SARS-CoV-2 Omicron sublineage BA.5, but also other highly pathogenic human coronaviruses including SARS-CoV-1 and MERS-CoV. In the Omicron-infected K18-hACE2 mouse model, oral treatment with SY110 significantly lowered the viral burdens in lung and alleviated the virus-induced pathology. Importantly, SY110 possesses favorable PK properties with high oral drug exposure and oral bioavailability, and also an outstanding safety profile. Furthermore, SY110 exhibited sensitivity to several drug-resistance M^pro^ mutations. Collectively, this investigation provides a promising new drug candidate against Omicron and other variants of SARS-CoV-2.

## Introduction

Since the outbreak of the Coronavirus Disease 2019 (COVID-19) pandemic due to the severe acute respiratory syndrome coronavirus 2 (SARS-CoV-2) in late 2019, more than three years have passed.^1,2^ In the battle against this virus, vaccines have played a decisive role. However, continuous viral mutations, from Alpha, Beta, to the latest Omicron, bring challenges to the protection effect of existing vaccines.^3–7^ Especially, the Omicron variant and its sublineages have more than 30 mutations on the spike protein, which is the main target of vaccines, and have shown much higher transmissibility and escape of immune elicited by natural infection or vaccines than other SARS-CoV-2 variants.^8–11^ Furthermore, historical experiences tell us that the long-term existence of SARS-CoV-2 is perhaps inevitable.^12^ Therefore, besides vaccines, effective antivirals are also an important strategy to address the current epidemic and future threats.

SARS-CoV-2 is a positive-sense single stranded (+ss) RNA virus.^2,13^ It belongs to the genus β-coronavirus of the family *Coronaviridae*.^2^ The SARS-CoV-2 genome encodes 14 open reading frames (ORFs) with a length of around 30 kb. These ORFs encodes two long polyproteins, pp1a and pp1ab, four structural proteins and nine accessory proteins. The two polyproteins, pp1a and pp1ab, are self-catalyzed and cleaved into 16 non-structural proteins (NSPs) by two cysteine proteases, the main protease (M^pro^, also referred to as 3CL^pro^) and the papain-like protease (PL^pro^).^14,15^ M^pro^ is a conserved gene in SARS-CoV-2 and its variants, as well as other highly pathogenic coronavirus, including SARS-CoV-1, MERS-CoV.^15–18^ This protease is responsible for the generation of 13 NSPs by cleaving pp1a and pp1ab, including RNA-dependent RNA polymerase (RdRp), helicase, exoribonucleases, 2′-O-methyltransferase and uridine-specific endoribonuclease.^15,19^ Of note is that M^pro^ recognizes and cleaves amino acid sequence of the substrate with specificity and its most cleavage sites at sequences Leu-Gln ↓ (Ser, Ala, or Gly) (↓ represents the cleavage site), while no known human proteases have the similar specificity.^20^ The critical role of M^pro^ in viral replication and its high conservation, combined with no known human proteases with similar cleavage specificity, render it an attractive target for the development of antivirals. The RdRp, a highly versatile enzyme that assists in RNA synthesis by catalyzing the RNA-template-dependent formation of phosphodiester bonds, is another important antiviral target, and has also been extensively studied.^18,19^

Currently, several small-molecule antivirals targeting M^pro^ or RdRp have been approved to use in some countries and regions worldwide. For example, three small-molecule antivirals, Remdesivir, Molnupiravir, and Paxlovid (a combination of Nirmatrelvir and Ritonavir), have been approved to use clinically by US Food and Drug Administration.^21–23^ Ensitrelvir has been approved by the Ministry of Health, Labour and Welfare of Japan (MHW).^24^ China National Medical Products Administration also approved three antivirals, Azvudine, Renmindevir, and Xiannuoxin (a combination of Simnotrelvir and Ritonavir).^25,26^ Of them, Remdesivir,^27^ Molnupiravir,^28^ Renmindevir,^29^ and Azvudine^30^ are inhibitors of SARS-CoV-2 RdRp, and Nirmatrelvir^31^ (the active ingredient of Paxlovid), Ensitrelvir,^32^ and Simnotrelvir^26^ (the active ingredient of Xiannuoxin) are inhibitors against SARS-CoV-2 M^pro^. However, due to the need of emergency response to the pandemic, they have more or less defects, for example, suboptimal potency, toxicity, or imperfect pharmacokinetic (PK) properties, including low oral drug exposure, poor oral bioavailability, and moderate stability in human liver microsomes (HLM).^26–29,31,33^ Moreover, drug resistance variants against currently approved drugs have already emerged. For examples, variants bearing E166N/V, M165T, G143S, Q189E, A173V, H172F/Q/Y, or Q192S/T/V mutants in M^pro^ have been reported to be resistant to Nirmatrelvir treatment.^34–41^ Therefore, developing next-generation antivirals is urgent.

In this investigation, we report the discovery of a new potent and selective M^pro^ inhibitor (SY110). The X-ray crystal structure of the M^pro^-SY110 complex indicates that SY110 occupies only the M^pro^ substrate binding sites S1′, S1, and S2 with an atypical binding mode. Meanwhile, SY110 showed potent antiviral activity against SARS-CoV-2 Omicron and its sublineages, as well as other highly pathogenic human coronaviruses including SARS-CoV-1 and MERS-CoV in vitro, and alleviated the virus-induced pathology in the Omicron-infected K18-hACE2 mouse model. Importantly, SY110 demonstrated favorable PK properties with high oral drug exposure and excellent oral bioavailability, as well as an outstanding safety profile, while can also partially overcome drug resistance mutations of M^pro^. Taken together, our studies demonstrate that SY110 could be a therapeutic candidate for COVID-19.

## Results

### Retrieval of a hit compound and its binding mode with M^pro^

**Fig. 1:**
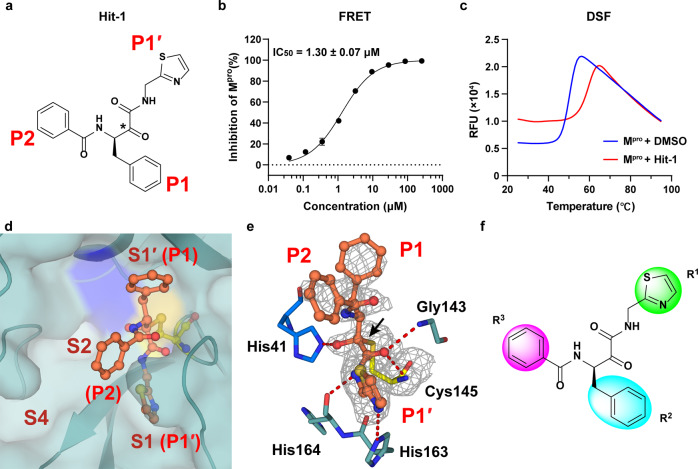
Discovery of a hit compound against SARS-CoV-2 M^pro^. **a** The chemical structure of Hit-1. The P1′, P1, and P2 moieties of Hit-1 are labeled. The warhead carbon is marked with a black asterisk. **b** Dose-activity curve of Hit-1 against SARS-CoV-2 M^pro^ in the FRET assay. Data shown are the mean ± standard deviation (SD) from three independent experiments. **c** Differential scanning fluorimetry analysis of the effect of Hit-1 on SARS-CoV-2 M^pro^ stability. Exposure of hydrophobic residues monitored by an increase in relative fluorescence units (RFUs). Curves represent the average of three experiments. **d** Hit-1 (orange) is located at the substrate-binding pocket of M^pro^ (cyan). His41 of M^pro^ is in blue, Cys145 is yellow. Pockets (S1′, S1, S2, and S4) of M^pro^ and moieties (P1′, P1 and P2) of Hit-1 are both labeled. **e** Interactions between Hit-1 and M^pro^. *Fo* – *Fc* density map is shown for Hit-1 (gray mesh, *σ* = 2.5) and Cys145. Covalent bond is shown by a black arrow and hydrogen bonds are displayed by red dashed lines. **f** Regions of Hit-1 for structural optimization. Images in **d**, **e** were processed by using PyMOL (https://pymol.org)

To obtain a new starting active compound for the drug development targeting M^pro^, we first screened an in-house chemical library containing about 30,000 compounds by fluorescence resonance energy transfer (FRET) assay. Four compounds were found to be able to inhibit the enzymatic activity of M^pro^ with 50% inhibition concentration (IC_50_) values less than 50 μM (Supplementary Fig. S1). Of them, the most potent compound, Hit-1, exhibited an IC_50_ value of 1.30 μM (Fig. 1a, b). The activity of Hit-1 was further validated by differential scanning fluorimetry (DSF) assay, which gave a thermal shift (Δ*T*_m_) value of 8.45 ± 0.35 °C (Fig. 1c), indicating a direct binding between Hit-1 and M^pro^.

To facilitate the subsequent structural optimization, we solved the co-crystal structure of M^pro^-Hit-1 complex (PDB ID: 8HHT, Supplementary Table S1). As shown in Fig. 1d, the S1′ and S1 pockets of M^pro^ were filled with benzyl (P1) and thiazole (P1′) moieties of Hit-1, respectively. Compared with other peptidomimetic M^pro^ inhibitors, the P1 and P1′ moieties present an inverted conformation, indicating an atypical binding mode to M^pro^. The P2 group directly points to the solvent region, rather than occupying the expected S2 pocket. The warhead carbon of Hit-1 is covalently linked to the sulfur atom of the catalytic Cys145 (Fig. 1e). The hydroxyl (or oxyanion) moiety of the thiohemiketal group generates a 2.5 Å hydrogen bond with the side-chain of His41. The amide oxygen of Hit-1 points to the canonical “oxyanion hole” and generates two hydrogen bonds with the backbones of Gly143 (3.0 Å) and Cys145 (3.1 Å), respectively. In addition, the amide nitrogen of P1′ forms a hydrogen bond with the main-chain of His164 (2.7 Å). Whereas the thiazole nitrogen of P1′ forms the other 3.3 Å hydrogen bond with the side-chain of His163 (Fig. 1e).

### Structural optimization of Hit-1

**Fig. 2:**
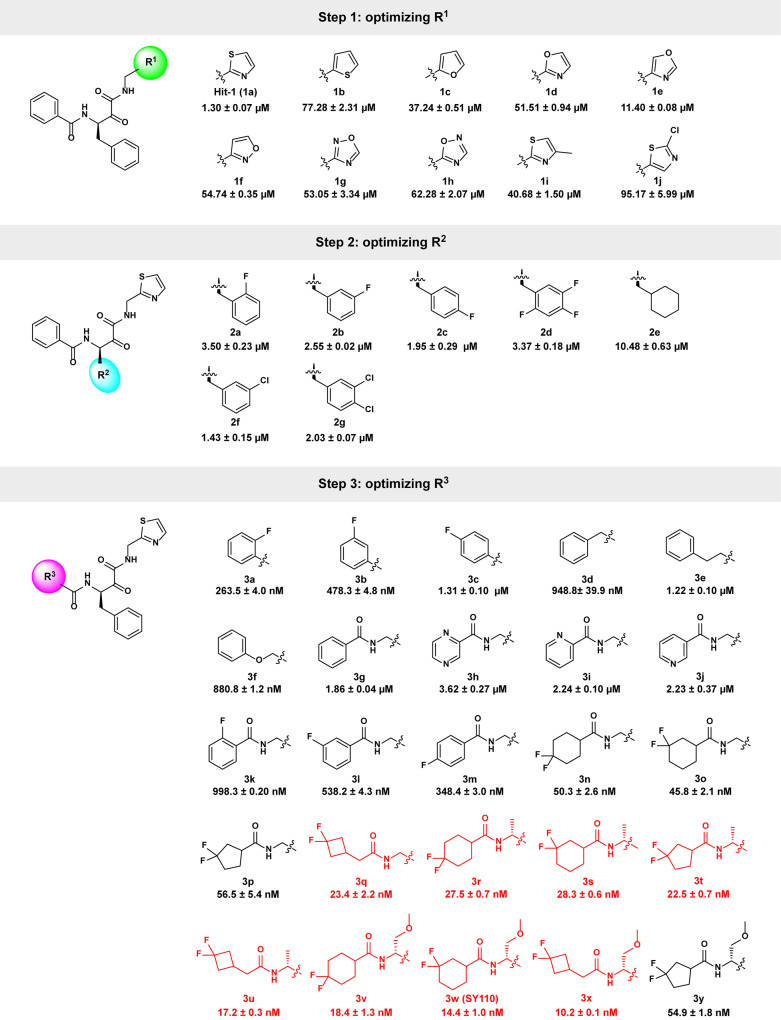
The stepwise structural optimization process of Hit-1. Bioactivities were measured by the FRET assay and are represented as the mean ± SD from three experiments. Molecules with IC_50_ < 30 nM are shown in red color

To improve the potency of Hit-1 (1a), we carried out a stepwise structural optimization, which focused on three regions: thiazole (P1′, R^1^), benzyl (P1, R^2^), and phenyl (P2, R^3^) (Fig. 1f). Firstly, we fixed R^2^ and R^3^ and optimized R^1^. A total of 9 new compounds (1b-j) with different R^1^ were synthesized. Unfortunately, the newly synthesized compounds did not exhibit improved activity against SARS-CoV-2 M^pro^ (Fig. 2). Secondly, we optimized R^2^ with R^1^ and R^3^ fixed as its original subgroups. Another 7 compounds (2a-g) with varied R^2^ were prepared and 1a was still the most potent compound (Fig. 2). We finally retained R^1^ and R^2^ and optimized R^3^. 25 new compounds (3a-y) were synthesized and 20 compounds showed elevated potency compared with 1a. Of special note are compounds 3q-3x, which showed IC_50_ < 30 nM (Fig. 2).

### Selection of SY110 as the candidate compound for further studies

**Fig. 3:**
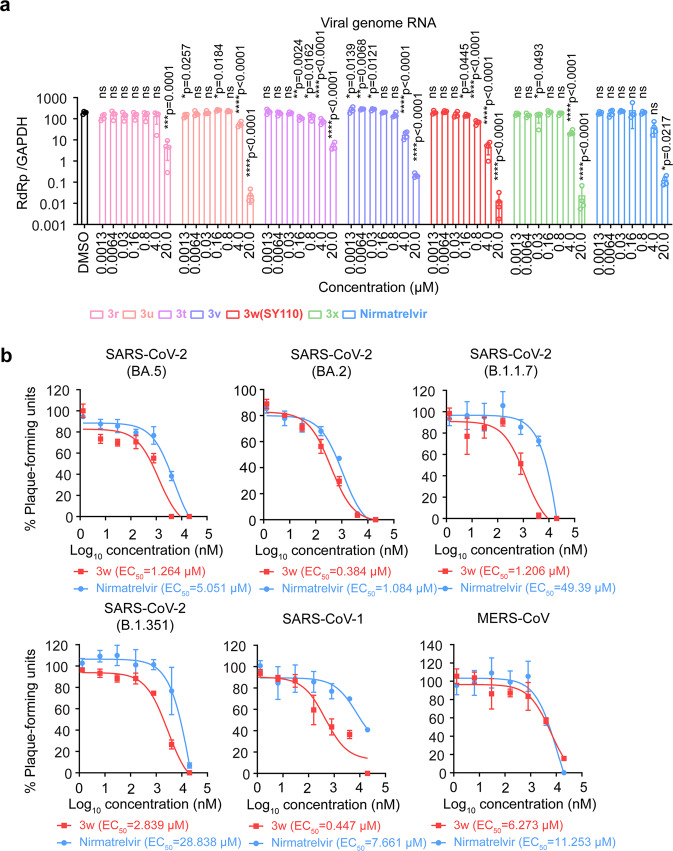
In vitro cellular antiviral activity of SY110. **a** Cells were infected with Omicron BA.1 sublineage at 0.1 MOI and treated with 3r, 3 u, 3t, 3 v, 3w (SY110), 3x, Nirmatrelvir or DMSO at 1 hpi.. At 24 hpi., cell lysates were harvested. Viral genome copies were quantified by probe-based RT-qPCR (*n* = 4). **b** Cells were infected and treated with 3w (SY110) or Nirmatrelvir at 2 hpi. (*n* = 3). Cells were fixed at 72 hpi. for plaque formation units quantification by visualization with 0.5% crystal violet staining. Plaque formation units were normalized to that of the vehicle group. Statistical significance is evaluated by one-way ANOVA in **a** compared to vehicle group. *p < 0.05; **p < 0.01; ***p < 0.001; ****p < 0.0001. ns, not statistically significant

To select a better candidate compound for further studies, we investigated the metabolic stability, bioavailability and toxicity of the eight most potent compounds (3q-x, Fig. 2) from the enzymatic assay. Six compounds (3r, 3t-x) showed comparable or higher stability compared with Nirmatrelvir (Supplementary Table S2). We next measured the PK parameters of the six compounds in Sprague-Dawley (SD) rats. All six compounds exhibited good PK properties, and compound 3w (referred to as SY110 hereafter) displayed much higher oral area under the curve (AUC) (19018.08 h*ng*mL^−1^) and oral bioavailability (*F*: 82.75%) than others (Supplementary Table S3). Finally, we investigated the cytotoxicity of the six compounds in different cells by MTT assay, including BEAS-2B, VeroE6, and HUVEC cells. As shown in Supplementary Table S4, except compound 3v, which showed relatively weak cytotoxicity (CC_50_: 129.2 - 158.6 μM), all other compounds did not display cytotoxicity (CC_50_ [concentration cytotoxicity 50%] > 500 μM). We carried on to examine the antiviral activity of the six compounds using the authentic SARS-CoV-2 Omicron BA.1 (B.1.1.529.1). Among the compounds tested, SY110 showed the most potent antiviral activity in VeroE6-TMPRSS2 (Fig. 3a). Comparing with Nirmatrelvir, SY110 suppressed the viral genome copies to significantly lower level at 0.8, 4 and 20 µM concentration (viral genome RNA [Nirmatrelvir *vs* SY110]: 3- (*p* = 0.0001), 8- (*p* = 0.0356), 9-fold (*p* = 0.0101) higher) (Fig. 3a). We further determined the antiviral potency of SY110 with plaque reduction assay. Consistent with the in vitro screening results, the 50% effective concentration (EC_50_) of SY110 determined with plaque reduction assay was 4-fold lower (EC_50_ [Nirmatrelvir *vs* SY110]: 5.051 µM *vs* 1.264 µM) than Nirmatrelvir against Omicron BA.5 sublineage (Fig. 3b). Since M^pro^ is evolutionarily conserved, we further evaluated whether SY110 demonstrated pan-coronavirus antiviral efficacy. SY110 consistently showed improved antiviral potency compared with Nirmatrelvir (Fig. 3b) against not only SARS-CoV-2 variants of concern (VOCs) B.1.1.7, B.1.351, and BA.2 but also SARS-CoV-1 and MERS-CoV. Together, SY110 demonstrated highly potent pan-coronavirus antiviral efficacy in vitro with high metabolic stability and remarkable in vivo bioavailability. Therefore, further in-depth studies are carried out on SY110 in the follows.

### Crystal structure of SARS-CoV-2 Omicron variant M^pro^ in complex with SY110

**Fig. 4:**
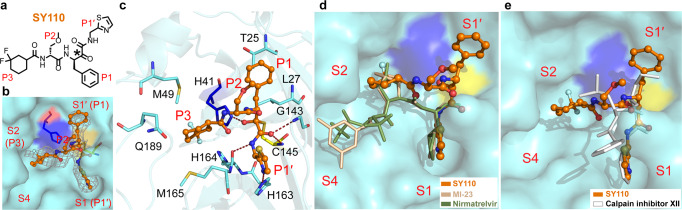
Crystal structure of SARS-CoV-2 Omicron variant M^pro^ in complex with SY110. **a** The chemical structure of SY110. The warhead carbon is marked with a black asterisk. **b** Close-up view of SY110 with the substrate-binding pocket of M^pro^. SY110 is in orange, M^pro^ is aquamarine, His41 of M^pro^ blue, Cys145 yellow. Four sites S1′, S1, S2, and S4 of M^pro^, and four moieties P1′, P1, P2, and P3 of SY110 are labeled, respectively. *Fo – Fc* density map of SY110 is shown (gray mesh, *σ* = 2.5). **c** Interactions between M^pro^ and SY110. The residues of M^pro^ involved in SY110 binding are displayed by sticks. The hydrogen bonds are displayed as red dashed lines. **d** Comparison of the binding modes of SY110 (orange, PDB ID: 8HHU), 11a (blue, PDB ID: 6LZE), MI-23 (wheat, PDB ID: 7D3I) and Nirmatrelvir (green, PDB ID: 7RFW). **e** Comparison of the binding modes between M^pro^-SY110 and M^pro^-calpain inhibitor XII (white, PDB: 6XFN). Images **b**–**e** were prepared using PyMOL (https://pymol.org)

The crystal structure of Omicron variant M^pro^-SY110 complex is in space group *C2* with one protomer per asymmetric unit (PDB ID: 8HHU, Supplementary Table S1). The electron density map illustrates a unique interaction mode of SY110 with M^pro^ (Fig. 4a–c). The P1′ thiazole ring of SY110 inserts into the S1 pocket while the P1 benzyl group occupies the S1′ site. The P2 chiral ether chain points to the solvent region, and the P3 3,3-difluorocyclohexyl group is placed in the S2 pocket (Fig. 4a, b). In detail, the carbonyl carbon of the SY110 α-ketoamide motif generates a 1.8 Å reversible covalent bond with the Cys145 in the (*R*) configuration (Fig. 4c). The oxyanion of this thiohemiketal moiety forms a hydrogen bond with the side-chain of His41 (2.3 Å). Meanwhile, the amide oxygen of SY110 is located into the canonical “oxyanion hole” and generates two hydrogen bonds with the backbone amides of Cys145 (3.0 Å) and Gly143 (3.2 Å), respectively. The amide nitrogen of P1′ generates a 3.0 Å hydrogen bond with the main-chain oxygen of His164, and the thiazole nitrogen of P1′ adopts another hydrogen bond with the side-chain of His163 (3.0 Å). The P1 benzyl moiety displays the hydrophobic interactions with the Cα, Cβ, Cγ of Thr25 and the Cβ, Cδ of Leu27 (Fig. 4c), while the P2 chiral ether chain is near to the flexible loop (_141_Leu-Asn-Gly-Ser_144_). The P3 3,3-difluorocyclohexyl moiety generates the hydrophobic interactions with residues Met49, Met165 and Gln189 (Fig. 4c).

The inhibitor SY110 P1′ and P1 moieties displays the inverted interaction mode with M^pro^, which is dissimilar to the previously reported peptidomimetic inhibitors (Fig. 4d), such as 13b,^20^ 11a,^42^ MI-23,^43^ and Nirmatrelvir.^31^ However, the similar inverted conformation of P1′ and P1 groups appeared in calpain inhibitor XII.^44^ We noticed that the Cα of P2 in the calpain inhibitor XII adopts the (*S*) configuration, which makes its P3 group point to the solvent region (Fig. 4e). In contrast, the Cα of P2 in SY110 adopts the (*R*) configuration, thus causing the SY110 P3 moiety to point into the S2 pocket and form the extensive hydrophobic interactions with M^pro^ (Fig. 4e).

### Evaluations of the drug metabolism and pharmacokinetics (DMPK)

**Fig. 5:**
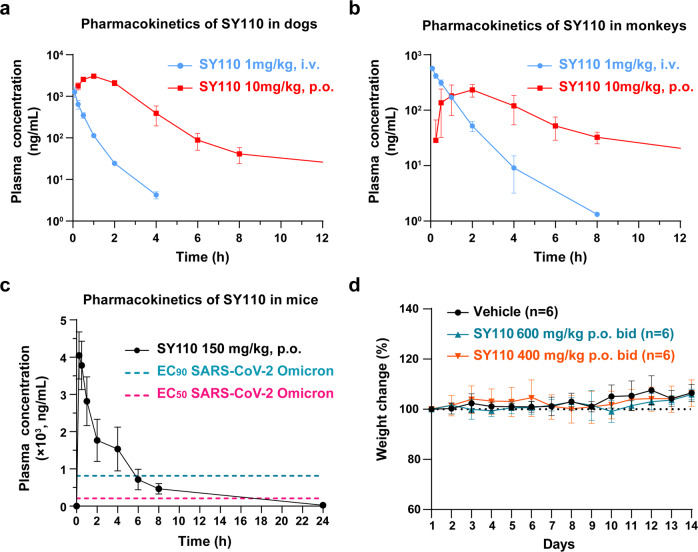
In vivo pharmacokinetics and safety data of SY110.SY110 was administered by intravenous injection (i.v.) or oral gavage (p.o.) route, then the blood samples of the dogs (**a**), monkeys (**b**), and mice (**c**) were analyzed. The two dash lines in (**c**) indicate the EC_50_ and EC_90_ values for SY110 against BA.2 variant (Fig. 3b). **d** The weight change (percentage of initial weight) of mice treated with SY110 by oral administration for two weeks (*n* = 6)

To evaluate the druggability of SY110, we examined the DMPK properties of SY110. Firstly, the inhibitory activity of SY110 against human cytochrome P450 enzymes was measured. In this assay, SY110 did not show obvious inhibitory activity against CYP1A2, 2B6, 2C8, 2C9, 2C19, 2D6, and 3A4 (IC_50_ values > 30 µM) (Supplementary Table S5), implying a low risk of drug-drug interaction (DDI). Then the human plasma protein binding of SY110 was examined under equilibrium dialysis conditions, which gave a plasma unbound fraction of 0.301, indicating moderate plasma protein binding (Supplementary Table S6). Next, we evaluated the pharmacokinetic properties of SY110 in beagle dogs, monkeys, and mice. SY110 displayed oral bioavailability of 131.99%, 24.36%, and 67.42%, respectively (Fig. 5a–c and Supplementary Table S7). Notably, SY110 demonstrated greater drug exposure, improved oral bioavailability, and a longer half-life in monkeys than Nirmatrelvir.^31^

### Safety evaluation of SY110

We subsequently evaluated the safety of SY110. Before these evaluations, we measured the inhibitory activity of SY110 against human proteases. SY110 did not exhibit activity against the tested various human proteases (IC_50_ > 100 µM, Supplementary Table S8), suggesting lower off-target toxicity. For the safety evaluation, we firstly carried out an Ames mutagenicity test and a chromosomal aberrations assay, which showed negative results in both experiments. Then the hERG channel blockade assay was performed, which revealed that SY110 is unlikely to cause cardiotoxicity (IC_50_ > 30 µM). No mice died following treatment with 1000 mg/kg of SY110 by oral gavage (p.o.) (Supplementary Table S9) during the acute toxicity study. All mice tolerated oral administration of SY110 at 600 mg/kg twice daily for 14 days without death, significant body weight change, or pathological changes in the heart, liver, lung, kidney, or spleen, indicating low in vivo toxicity (Fig. 5d and Supplementary Table S9). We also evaluated the reproductive toxicity of SY110. Results of the embryo-fetal development studies in rats showed that there were no observed SY110-related maternal toxicity, reproductive toxicity, and effect on embryo-fetal viability (including fetal external, visceral or skeletal morphological development) at 1000 mg/kg/day (the highest dose tested), indicating lower teratogenic risk (Supplementary Table S10). Overall, SY110 showed outstanding preclinical safety.

### In vivo antiviral activity of SY110 in the K18-hACE2 transgenic mouse model

**Fig. 6:**
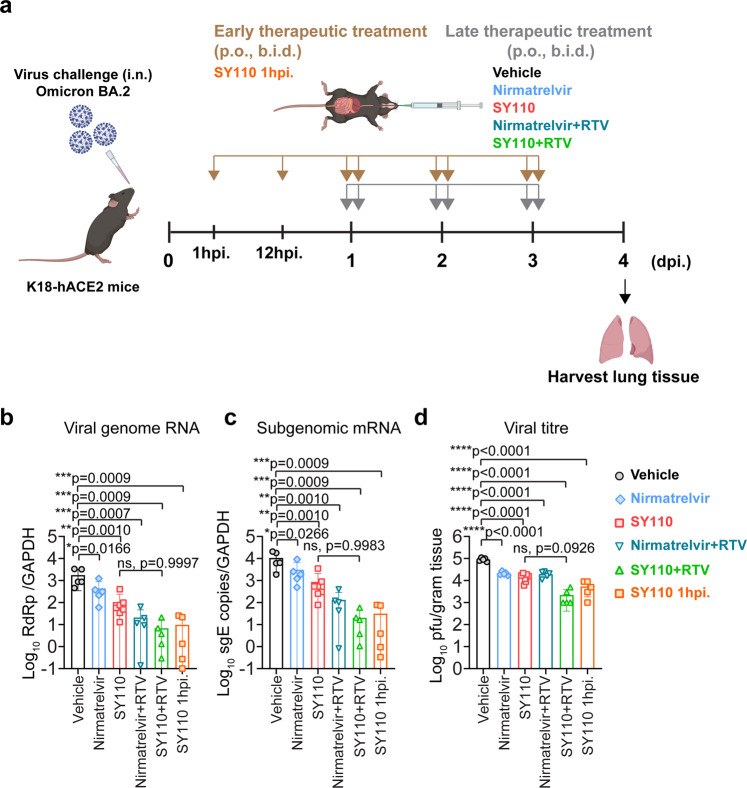
In vivo antiviral activity of SY110. **a** The schematic illustration of experiment design. **b** The viral RNA-dependent RNA polymerase (RdRp) gene copies were quantified by probe-based RT-qPCR (*n* = 5–7) or **c** subgenomic envelope (sgE) mRNA copies were quantified by probe-based RT-qPCR (*n* = 5–7) or **d** viral titer quantification with plaque assay (*n* = 5–7). All data were obtained from three times of independent experiments and shown as mean ± SD. Statistical differences were determined with one-way analysis of variance (ANOVA) with Tukey’s multiple comparison test in **b**–**d**. *p < 0.05; **p < 0.01; ***p < 0.001; ****p < 0.0001

**Fig. 7:**
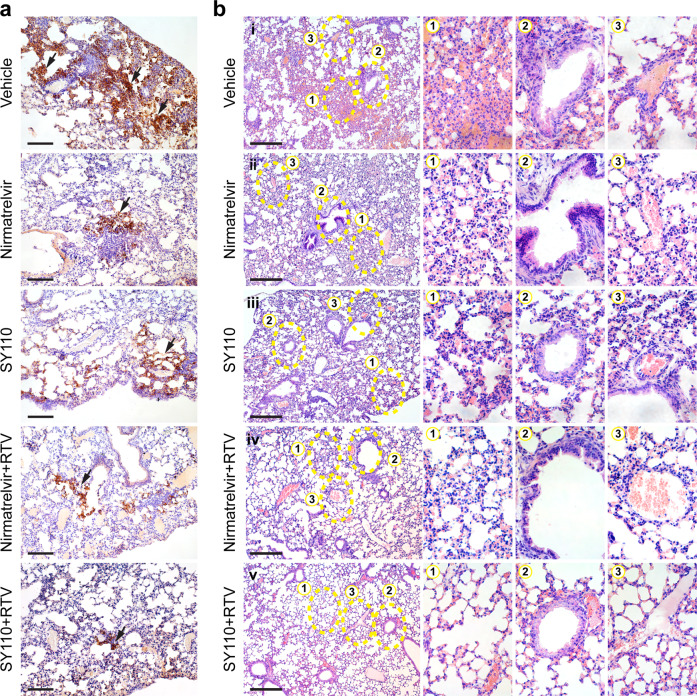
Immunohistochemistry and H&E analysis of the infected mice lung tissue. **a** Representative images of immunohistochemistry staining visualizing the SARS-CoV-2 nucleocapsid protein (brown, indicated by black arrows) in the lung of the infected mice at 4 dpi. (*n* = 5–7). Scale bar, 200 µm. **b** Representative images of H&E staining visualizing the virus-induced pathology in the lung of the infected mice at 4 dpi. (*n* = 5–7). Representative images of alveoli, bronchioles, and blood vessels of the lung indicated by yellow dashed circle were enlarged with numbers 1, 2, and 3, respectively. Scale bar, 500 µm

To assess the in vivo antiviral efficacy of SY110 by oral delivery, we utilized the previously established K18-hACE2 transgenic mouse model infected with the Omicron BA.2 sublineage (Fig. 6a).^45^ Early therapeutic treatment with SY110 at 1 hpi. substantially lowered viral genomic RNA (vRNA) in the lung by 179 folds (*p* = 0.0009) at 4 dpi. (Fig. 6b). In parallel, production of the subgenomic mRNA of the envelope gene (sgE) was inhibited by 337 folds (*p* = 0.0009) under the same treatment condition (Fig. 6c). Even if SY110 treatment was postponed to 24 hpi., vRNA and sgRNA copies in the lung was reduced by 16- (*p* = 0.001) and 12- (*p* = 0.001) folds compared with mock-treated mice at 4 dpi., respectively (Fig. 6b, c). In comparison, vRNA and viral sgE copies of the Nirmatrelvir-treated mice was 3- (*p* = 0.9226) and 2-folds (*p* = 0.8916) higher that of the SY110-treated mice, respectively. Meanwhile, we measured the infectious viral burden in the lung with plaque assays. In accordance with viral gene copy quantification, production of the infectious virus particles in the lung was effectively suppressed by therapeutic treatment with SY110 (Fig. 6d). We and others previously reported co-administration with Ritonavir (RTV) delayed the clearance of antiviral compounds by liver microsomes, thus enhancing their pharmacokinetics and antiviral efficacy in vivo.^31,46^ Therefore, we also explored the synergistic therapeutic effect of co-administration with SY110 and RTV. Remarkably, therapeutic treatment initiated at 24 hpi. with SY110/RTV combined further alleviated viral burden in the lung by 15- (vRNA, *p* = 0.9997), 43- (sgRNA, *p* = 0.9983), and 6-folds (infectious viral titer, *p* = 0.0926) compared to single treatment, respectively. Importantly, infectious viral titers in the mice lung with SY110/RTV was significantly decreased by ~90% (*p* = 0.0127) compared to those co-treated with Nirmatrelvir/RTV (Fig. 6d). In addition, viral antigen expression in the lung tissue was visualized with immunohistochemistry staining against the SARS-CoV-2 nucleocapsid (N) protein. Consistent with the virological assessment findings, treatment with SY110 substantially reduced the viral N protein expression in the lung tissue compared with the mock-treated mice (Fig. 7a). Moreover, combination treatment of SY110/RTV further restricted the viral antigen expression to much lower level in the infected mouse lung tissue (Fig. 7a). Next, pathological lesions induced by SARS-CoV-2 infection in the lung tissue was examined with haematoxylin-eosin (H&E) staining (Fig. 7b). Despite robust virus replication in the airway, Omicron infection induced attenuated pathological lesions compared with other variants.^47–49^ The most prominent lung pathology of the vehicle control group was characterized by multi-focal inflammatory infiltrations in the alveolar septa, peribronchiolar regions and perivascular areas (Fig. 7b). In contrast, only scattered inflammatory cell infiltrates can be occasionally found in the alveolar interstitium of the mice therapeutically treated with SY110 (Fig. 7b). Evidently, SY110/RTV co-administration further improved the lung architecture compared with SY110 single treatment. Together, our in vivo results showed that therapeutic treatment with SY110, especially when co-administered with Ritonavir, potently suppressed Omicron infection in the airway and alleviated virus-induced lung pathology in the infected animals.

### Inhibitory activities of SY110 against Nirmatrelvir-resistance M^pro^ mutants

**Fig. 8:**
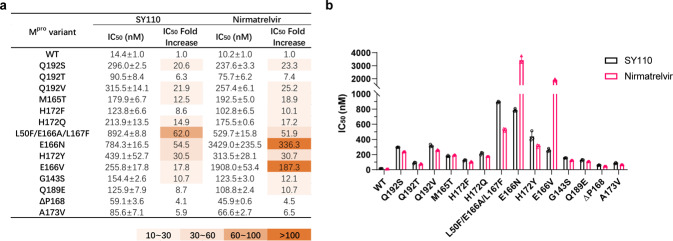
Effects of resistance mutations on the inhibition of SARS-CoV-2 M^pro^ by SY110 and Nirmatrelvir. **a** Inhibition of SY110 and Nirmatrelvir against SARS-CoV-2 M^pro^ polymorphisms. IC_50_ fold increase is relative to the WT. **b** IC_50_ determination of SY110 and Nirmatrelvir against M^pro^ mutants. Data are shown as mean ± SD based on three independent experiments

Nirmatrelvir is the clinically-approved specific M^pro^ inhibitor for COVID-19 treatment currently. However, with increasing application of Nirmatrelvir, the emergence of drug-resistant mutant strains is posing an imminent challenge to the healthcare system. E166N/V M165T, G143S, Q189E, A173V, H172F/Q/Y or Q192S/T/V mutation in the M^pro^ was recently shown to be associated with Nirmatrelvir resistance.^34–41^ To evaluate the efficacy of SY110 against these mutated M^pro^, we expressed a panel of 14 mutated recombinant M^pro^ and tested their enzymatic sensitivity to SY110 and Nirmatrelvir by FRET assay. As shown in Fig. 8, Nirmatrelvir displayed obviously reduced activity against all these M^pro^ mutants. Especially E166N and E166V are the most resistant mutants for Nirmatrelvir with IC_50_ values of 3.429 μM (336.3 folds) and 1.908 μM (187.3 folds), respectively. Although SY110 also displayed reduced activity against most of these mutants, it exhibited substantially elevated activity against the E166N and E166V mutants, indicating that SY110 can, at least partially, overcome these two kinds of Nirmatrelvir-resistances. A possible explanation for this might be as follows: E166 is a hotspot for Nirmatrelvir-resistant mutation,^34–41^ which locates at the S1 pocket. The side chain oxygen of E166 forms a 3.2 Å hydrogen bond with the lactam ring of Nirmatrelvir.^31^ The valine (E166V) or asparagine (E166N) substitution at this position directly impacted the ligand interactions of Nirmatrelvir in the S1 site, which results in the strong drug resistance observed. Unlike Nirmatrelvir, SY110 does not form any hydrogen bonding interaction with the E166 (Fig. 4c). Although E166 mutations also lead to a decrease in activity of SY110, the reduction is not too significant.

## Discussion

Omicron and its sublineages were characterized as a variant of concern by WHO due to their higher transmissibility and altered virological features.^50,51^ Despite vaccination, mortality and morbidity were still observed due to the Omicron infection.^52^ Therefore, small-molecule antivirals targeting conserved viral genes are particularly important. M^pro^ is an appealing target for the development of antiviral compounds, due to its critical role in the viral life cycle and its high conservation among different coronaviruses and the continuously emerging mutants of SARS-CoV-2.^15–18^ Currently many M^pro^ inhibitors have been reported.^20,31,32,42,43,53–56^ Nevertheless, a majority of them have a low potency and/or a poor pharmacokinetic profile, which obstructs their further advance. The marketed M^pro^ inhibitor, Nirmatrelvir, showed high potency against M^pro^, but still has some defects in PK properties.^31^ Moreover, Nirmatrelvir resistance has also been reported although it has been on the market for less than one year.^34–41^ Therefore, developing more M^pro^ inhibitors with new chemical structures and/or new action modes is necessary.

SY110 reported here is a new potent and selective M^pro^ inhibitor with IC_50_ of 14.4 nM against M^pro^. This compound has good PK properties in mice, rats, dogs and cynomolgus monkeys, with oral bioavailability of 67.42%, 82.75%, 131.99%, and 24.36%, respectively. It displayed robust in vitro antiviral potency against SARS-CoV-2 Alpha (B.1.1.7), Beta (B.1.351), Omicron (B.1.1.529) BA.2 and BA.5 sublineages. As a potent pan-coronavirus antiviral inhibitor, SY110 also has notable efficacy on other coronaviruses like SARS-CoV-1 and MERS-CoV-1. In K18-hACE2 mice of Omicron (B.1.1.529) infection, oral treatment with SY110 significantly improved pathological damage in both turbinate and lung. Moreover, SY110 exhibited favorable in vitro safety profiles in the hERG test, the CYP assay, the Ames test, and chromosome aberration test. The maximal tolerated single doses of SY110 were at least 1.0 g/kg in rats and no observed adverse effect levels at 600 mg/kg in rats in the 14 days repeated dose toxicity studies. Collectively, the data presented here demonstrate that SY110 is an effective and safe antiviral drug candidate against SARS-CoV-2.

Antiviral drug resistance is an increasing concern, particularly in immunocompromised patient populations, which has been often observed in the therapy of influenza, hepatitis, and human immunodeficiency virus. Despite only very short time since the use of antivirals against SARS-CoV-2, drug resistance has also been found, mainly due to the mutations of viral genome. For example, SARS-CoV-2 viruses carrying M^pro^ mutants E166N/V, M165T, G143S, Q189E, A173V, H172F/Q/Y, or Q192S/T/V are found to be resistant to Paxlovid.^34–41^ To predict the ability of SY110 to overcome the drug-resistance, we in this investigation expressed a panel of 14 mutated recombinant M^pro^; viruses carrying these M^pro^ mutants are reported to be resistant to Nirmatrelvir. Our data show that SY110 can, to some extent, overcome the Nirmatrelvir-resistances caused by mutations at E166N (IC_50_ folds: 54.5 vs 336.3) and E166V (IC_50_ folds: 17.8 *vs* 187.3). Pfizer reported in the Paxlovid label that in the EPIC-HR clinical trial, M^pro^ E166V was detected in more patients after Paxlovid treatment than those after placebo treatment.^57^ This means that SY110 can be used as an alternative drug of Paxlovid or in combination use with Paxlovid in future.

Compared with Nirmatrelvir and other reported peptidomimetic M^pro^ inhibitors, SY110 possesses the following unique features and advantages: 1) SY110 adopts a distinct binding mode with M^pro^: the inverted conformation of P1′ and P1, the *(R)* configuration of Cα in P2 as well as the P3 pointing to the S2 pocket, present a unique binding to M^pro^. Particularly, SY110 does not form a hydrogen bond with the side chain of E166, while this hydrogen bond is crucial for Nirmatrelvir to maintain its inhibition effect. With the E166V mutation in M^pro^, the IC_50_ value of Nirmatrelvir reduces about 100 folds.^39^ Currently, SARS-CoV-2 variant with the E166V mutation has appeared (GenBank: OP814611.1). Therefore, SY110 could preserve the inhibition on such mutated variant because of its specific binding fashion. 2) SY110 possesses favorable PK properties, especially high oral drug exposure and oral bioavailability, and a long half-life period (*T*_1/2_). Of note is that the drug concentration in plasma after p.o. 150 mg/kg administration in mice could maintain EC_90_ for at least 6 h (Fig. 5c), indicating that a single use of SY110 by 3-4 times oral administration per day might achieve a better therapeutic effect. 3) It has excellent target selectivity and preclinical safety. Even so, the clinical therapeutic efficacy and safety need to be extensively evaluated in the subsequent clinical trial studies. Additionally, we have to mention that, despite possessing favorable PK properties, there is still a room to further improve the HLM stability for SY110. Overall, SY110 is a promising antiviral drug candidate against Omicron and other SARS-CoV-2 variants, deserves further clinical development.

## Materials and methods

### Ethics statements

All procedures related to animal in pharmacokinetic (PK) studies were performed according to the guidelines approved by the Institute Animal Care and Use Committee (IACUC) of Sichuan Greentech Biotechnology Co. Ltd, ZLA (Beijing) Pharmaceutical Technology Co. Ltd, and Shanghai Medicilon Inc. All procedures related to animal in toxicity study was approved by the IACUC (20211063A). All procedures related to animal in antiviral study was approved by the CULATR (5779-21).

### Compounds, cell lines, and viruses

The compounds (1a-j, 2a-g, and 3a-y) shown in Fig. 2 were synthesized in our internal facilities. And the detailed synthesis methods and characterization data of these are listed in the supplementary materials. BEAS-2B cells (GNHu27), HUVEC (GDC0635), VeroE6 cells (CRL-1586) and VeroE6-TMPRSS2 (JCRB 1819) cells were cultured in DEME (Gibco) with fetal bovine serum (10%) and penicillin-streptomycin (1%). Cells were grown at 37 °C in a humidified atmosphere of 5% CO_2_.

SARS-CoV-2 B.1.1.7 (GISAID: EPI_ISL_1273444), B.1.351 (GISAID: EPI_ISL_2423556), B.1.1.529 BA.2 subvariant (GISAID: EPI_ISL_9845731) and B.1.1.529 BA.5 subvariant (GISAID: EPI_ISL_13777658) were derived from laboratory-confirmed COVID-19 patients in Hong Kong. R. Fouchier kindly provided the MERS-CoV strain (EMC/2012). SARS-CoV-1 GZ50 were archived clinical isolates at Department of Microbiology, HKU. Viruses were titrated by plaque assays in VeroE6-TMPRSS2 cells. All infectious studies followed the BSL-3 facility at the Department of Microbiology, HKU.

### Production of authentic SARS-CoV-2 main protease and its mutants

Main protease (M^pro^) of wide type SARS-CoV-2 (GenBank: MN908947.3) and Omicron variant (BA.5, GenBank: OP054053) was cloned into pET28b vector with E. coli codon optimization. Using the former plasmid as template, M^pro^ mutations were gained by site-directed mutagenesis PCR. And the corresponding primers are presented in Supplementary Table S11. After digested by DpnI (TakaRa), the PCR products were transformed into DH5α (Novagen) and the plasmids were extracted and verified.

The detailed M^pro^ and its mutant genes cloning, expression and purification used the same approach as we reported previously.^43,46^ Briefly, the cDNA sequence was designed as the M^pro^ cleavage-site at N-terminus and the PreScission cleavage-site (removing the hexa-histidine tag) at the C-terminus. The plasmid of WT (or each mutant) M^pro^ was transformed to E. coli BL21 cells and cultured with kanamycin (50 μg/mL, 37 °C). Until the E. coli BL21 cells were grown to an optical density at 600 nm of ~0.8, expression was induced by adding isopropyl-D-thiogalactoside [0.5 mM (final concentration)] to the cell culture at 18 °C, 16–18 h. The cell pellets were resuspended, lysed and centrifugation. Then, the supernatants were loaded onto HisTrap FF column (GE Healthcare) and the protein was eluted. PreScission protease cleaved the target protein to remove its hexa-histidine tag. The processed target protein dialyzed overnight. The flowthrough containing M^pro^ was dealt with GSTtrap FF (GE Healthcare) and HisTrap FF column, then purified through gel filtration (GE Healthcare).

### Crystallization, data collection, phase determination, and refinement

M^pro^ (~5 mg/mL) incubated with Hit-1 or SY110 at a molar ratio of 1:10 for 12–14 h (4 °C). Crystallization was performed at 291 K employing the sitting-drop vapor-diffusion method, 1 μL + 1 μL drops. The best crystal of M^pro^-Hit-1 was observed under condition No. 85 of Index^TM^ (Hampton research): 0.2 M Magnesium chloride hexahydrate, 25% w/v PEG3350, 0.1 M Tris pH 8.5. M^pro^-SY110 was obtained under condition No. 42 of Index^TM^: 25% w/v PEG3350, 0.2 M BIS-TRIS pH 5.5. Then, the fished crystals were flash-cooled in liquid nitrogen.

X-ray diffraction experiment for M^pro^-Hit-1 was collected at 0.97852 Å at beamline BL18U1 (Shanghai Synchrotron Radiation Facility, Shanghai, China). M^pro^-SY110 was carried out at 0.976246 Å at the beamline BioMax (MAX IV Laboratory, Sweden). After *XDS*^58^ processing, the datasets further scaled with *Aimless*^59^ in CCP4. And the structures of both SARS-CoV-2 M^pro^-Hit-1 and SARS-CoV-2 M^pro^-SY110 were determined by molecular replacement, using the M^pro^ (PDB ID: 7C7P) as a search model. M^pro^-Hit-1 structure was refined with Phenix.refine,^60^ while M^pro^-SY110 was refined with program *BUSTER*.^61^ The two models were inspected and rebuilt using *Coot*.^62^ In the final structures of M^pro^-Hit-1 and M^pro^-SY110, Rfactor/Rfree values are 0.20/0.25 and 0.19/0.23, respectively. 98.3% of M^pro^-Hit-1 and 98.7% of M^pro^-SY110 residues are in the the “Ramachandran plot” statistical regions. Supplementary Table S1 summarizes the diffraction dataset and statistics of final refinement.

### FRET-based enzymatic assay

For in-house chemical library screen, 10 mM DMSO stock solutions of compounds were diluted in assay buffer with final concentration of 100 μM. The protease (final concentration: 100 nM) was incubated with diluted compounds at 37 °C for 10 min, followed by the addition of 25 μL of assay buffer containing substrate (MCA-AVLQSGFR-Lys (DNP) -Lys-NH2, final concentration: 20 μM). The fluorescence signal was read at 320 nm excitation and 405 nm emission on the microplate reader (BMG). By using the simple liner regression model in GraphPad Prism 8.0 software, the primal velocities were calculated. Inhibition percentage was calculated by normalizing to DMSO controls.

For IC_50_ measurement, recombinant protease with optimized concentrations was added with each compound for 10 min. Then, the process of adding fluorescent substrate to initiate the reaction, using the microplate reader (BMG) to read, and using a dose-response model to calculate the IC_50_ values.

### Differential scanning fluorimetry (DSF) assay

M^pro^ was mixed with tested compounds and 5 × SYPRO Orange dye (Sigma) avoiding from light. The thermal denaturation spectra were run from 20 °C to 95 °C at a gradient of 1.5 °C/min (CFX96, BioRad). Data were analyzed by Boltzmann model to calculate the melt temperature (*T*_m_). The equation Δ*T*_m_ = *T*_m(compound)_ − *T*_m(DMSO)_ was used to calculate the thermal shift (Δ*T*_m_). The values are presented as mean ± SD (*n* = 3).

### Mammalian proteases activity assay

The mammalian proteases activity assay was analyzed using the assay kit (Biovision, USA) in triplicate by following manufacturer’s manual.

### Cytotoxicity assay

Cells were seeded and grown in 96 well plates overnight. Then various concentrations of compounds in fresh medium were added and incubated for 72 h. Using MTT (Sigma) assay to determine the cell viability.

### Pharmacokinetic properties

The information of compounds, doses and administration scheme for PK studies are showed in Supplementary Table S3 and Supplementary Table S7. Briefly, male SD rats (*n* = 3), male ICR mice *n* = 3), male beagle dogs (*n* = 3), or male cynomolgus monkeys (*n* = 3) were treated by intravenous or gavage (p.o.). For male SD rats, the vehicle was consisted with 5% DMSO, 40% PEG400, 52% saline, and 3% Poloxamer-188. For the mice, dogs, and monkeys, the vehicle was consisted with 5% DMSO, 40% PEG400, 52% saline, and 3% Solutol HS-15.

After administration, the blood samples were collected at indicated time points and analyzed by liquid chromatography tandem mass spectrometry (LC-MS/MS).

### Metabolic stability evaluation

Compounds were incubated with human liver microsomes (0.5 mg/mL) at room temperature. Samples were collected and analyzed by LC-MS/MS at specific time points. The equation: *T*_1/2_ = 0.693/*K* (*K* is the rate constant from a plot of ln [concentration] vs. incubation time) and CL_int_ = (0.693/*T*_1/2_) × {1/[microsomal protein concentration (0.5 mg/mL)]} × Scaling Factors (1254.2) were used to calculate the substrate depletion half-life (*T*_1/2_) and intrinsic clearance (CL_int_).

### Human plasma protein binding determination of SY110

The frozen plasma was thawed at 37 °C and centrifuged at 12,000 rpm for 5 min and collect the supernatant. Soak the dialysis membrane strips in distilled water and ethanol for 1 h and 20 min, respectively, and then rinse the membrane strips in distilled water three times to pretreatment the membrane strips. Spike 20 µL of test solutions containing the compound or the reference compound (final test concentration 1 µM) into the pre-loaded plasma in the 96-well plate. Apply aliquots of 100 µL of blank dialysis buffer to the receiver side of dialysis chambers. Then apply aliquots of 100 µL of the plasma spiked with test and reference compounds to the donor side of the dialysis chambers. Aliquot 25 µL of the plasma spiked with test and reference compounds into a 96-well sample preparation plate as *T*_0_ samples. The entire dialysis apparatus was placed in a shaker (600 rpm) at 37 °C for 5 h. Samples from the specified time point of 0–5 h were collected for LC/MS analysis.

### Human CYP450 inhibition test of SY110

Preheat 0.1 M pH 7.4 K/Mg-buffer at 37 °C. The reference inhibitors for this assay are α-Naphthoflavone (1A2), Ticlopidine (2B6), Montelukast (2C8), Sulfaphenazole (2C9), Omeprazole (2C19), Quinidine (2D6), Ketoconazole (3A4). Test compound and reference inhibitors were dissolved in ACN. NADPH cofactor was dissolved in K/Mg-buffer. Each CYP450 isoform was prepared 2 mL substrate (1A2: Phenacetin, 2B6: Amfebutamone HCl, 2C8: Paclitaxel, 2C9: Diclofenac, 2C19: S-Mephenytoin, 2D6: Dextromethorphan, 3A4: Midazolam and Testosterone). Add 400 μL 0.2 mg/mL HLM and 2 μL of test compound set (serially diluted) to the assay wells. And then add 30 μL HLM-compound mixture and 15 μL substrate solution in the 96-well assay plate on ice. Then incubate the 96-well assay plate at 37 °C for 5 min. Add 15 µL of pre-warmed (37 °C) 8 mM NADPH solution to initiate the enzyme reaction. Incubate the assay plate at 37 °C for 5–45 min and quench the reaction. Then collect samples for LC/MS analysis.

### In vivo toxicity study

Male and female ICR mice (6–8 weeks, 16–25 g) were used to assess the in vivo toxicity of SY110. SY110 were dissolved in 5:40:3:52 DMSO/PEG400/Solutol HS-15/saline. The specific information is showed in Supplementary Table S9. During the experiment, monitor the toxic signs (behaviors, food intake and weight) of the animals at least twice a day. At the last day, samples of visceral tissue were collected and analyzed by histological examine.

### Embryo-fetal development study of SY110

SD rats (9–14 weeks and 230–320 g at study beginning) were randomly assigned to dose groups (at least seven successful mating animals in each group). Rats were administered SY110 (100, 300, or 1000 mg/kg, po) or vehicle from gestation day (GD) 6 through day 17. According to the ICH guidelines, the limit dose of SY110 is 1000 mg/kg/day. Observe the clinical signs, gestation body weights, weight changes and food consumption of animals during the study. On GD 20, the rats were weighed, euthanized by CO_2_ inhalation, exsanguinated, and necropsied.

### In vitro antiviral screening

VeroE6-TMPRSS2 cells were infected with Omicron (B.1.1.529) BA.1 subvariant at 0.1 MOI and treated with 3r, 3u, 3t, 3v, 3w (SY110), 3× or Nirmatrelvir. Cell lysates were collected and extracted for RNA by RNeasy Mini kit (Qiagen) at 24 hpi. Quantify viral genome copies with one-step RT-qPCR kit (Qiagen) and Real-time PCR system (LightCycler480, Roche).^63^

### Plaque reduction assay

Cells (VeroE6-TMPRSS2) were infected with MERS-CoV, SARS-CoV-1, SARS-CoV-2 Alpha (B.1.1.7), Beta (B.1.351), Omicron (B.1.1.529) BA.2 and BA.5 sublineages at 50-70 PFU. After 2 h inoculation, gently wash the cells with PBS 3–5 times. Then use 2 × DMEM/2% FBS-2% agarose/PBS mixture cover the cells followed by treatment with SY110 or Nirmatrelvir which were fivefold serially diluted from 20 µM to 0.0013 µM at 2 hpi.. At 48–72 hpi., cells were fixed and stained for 10 min by crystal violet.

### In vivo SARS-CoV-2 infection

Female or male K18-hACE2 transgenic mice (6–8 weeks, The Jackson Laboratory) were used for the in vivo antiviral study. Mice were inoculated with Omicron BA.2 subvariant (2000 PFU) by intranasally. For early therapeutic treatment, mice were treated with SY110 (150 mg/kg, po, bid) from the day of infection until 4 dpi. For late therapeutic treatment, mice were administered with Nirmatrelvir (150 mg/kg, po, bid), SY110 (150 mg/kg, po, bid) with or without co-administration of Ritonavir (10 mg/kg, po, bid) until 4 dpi. Mice treated with vehicle (5% DMSO/3% Solutol HS-15/40% PEG400/normal saline) were included as controls. Mice were sacrificed at 4 dpi. and lung tissues were sampled for virological and histopathological analyses as previously described.^64^

### RNA extraction and quantitative RT-PCR

Lung samples were homogenized using the TissueLyzer II (Qiagen). After lysed by RLT buffer (Qiagen), tissue lysates were extracted with the RNeasy Mini kit (Qiagen) following the manufacturer’s protocol. RNA was extracted, and RT-qPCR analysis was determined using the one-step RT-qPCR Kit (Qiagen) on the Real-Time PCR System (LightCycler 480, Roche).^65^

### Plaque assays

Cells (VeroE6-TMPRSS2) were grown in 12-well plates overnight. Next day, cells were incubated with serially diluted samples for 1 h at room temperature. Then cells were washed 3–5 times by PBS and covered with 2 × DMEM/2% FBS, 2% agarose / PBS mixture at 1:1 ratio. The cells were incubated with the mixture at 37 °C for 72 h. After fixation, 10 min was stained with 25% (v/w) ethanol/ water by 0.5% crystal violet to show plaques.^45^

### H&E staining and immunohistochemistry assays

The assays were conducted using previous protocols.^43^ Lung simples of the infected animals were fixed with 10% neutral-buffered paraformaldehyde solution and processed with semi-automatic tissue processor (Leica, Germany). Processed tissues are embedded with paraffin and cut at 5 μm thickness. Dewaxed and rehydrated tissues were treated for antigen exposure before being incubated with the rabbit anti-SARS-CoV-2-N immune serum (in-house) at 4 °C overnight. Next day, tissues were treated with the goat anti-rabbit IgG antibody (biotinylated, BA-1000-1.5, Vector aboratories) at room temperature for 1 h. Then DAB (3,3’-diaminobenzidine) substrate kit (Vector Laboratories) was used for visualization of signal. Tissue slides were counterstained with Gill’s Hematoxylin for visualization of the cell nuclei. For H&E staining, lung tissue slides were stained with Gill’s hematoxylin (Vector Laboratories) and eosin Y (Thermo Fisher Scientific). Images were obtained with the inverted light microscope (BX53, Olympus Life Science). Images shown represented unbiased description for the pathological damage in the lung which have been confirmed by an experienced pathologist.

### Statistical analysis

All data were represented as means and standard deviations and analyzed with GraphPad Prism 8.0 software. Statistical comparison between different groups was performed by one-way ANOVA, and details are showed in the figure legends, respectively (Figs. 3, 6). Differences were considered statistically significant when *p* < 0.05 (*p < 0.05; **p < 0.01; ***p < 0.001; ****p < 0.0001).

## Supplementary information

Supplemental material .docx

## Data Availability

All data related to this research is listed in the main text or Supplementary Materials, and are available from the corresponding authors (S.Y., J.L., or H.C.) upon reasonable request. The coordinates and structure factors of SARS-CoV-2 M^pro^ in complex with Hit-1 and SY110 have been deposited into PDB with accession numbers 8HHT and 8HHU, respectively.
