# Supplementary material for: A new generation Mpro inhibitor with potent activity against SARS-CoV-2 Omicron variants
Source: Signal Transduct Target Ther. 2023 Mar 16;8:128. doi: 10.1038/s41392-023-01392-w (PMC10018608; doi:10.1038/s41392-023-01392-w)
Supplement: Supplementary file 1 — Supplemental material .docx [file 41392_2023_1392_MOESM1_ESM.docx]

Supplementary Materials for

A new generation M^pro^ inhibitor with potent activity against SARS-CoV-2 Omicron variants

Chong Huang^1†^, Huiping Shuai ^2,3†^, Jingxin Qiao^1†^, Yuxin Hou^2,3†^, Rui Zeng^1†^, Anjie Xia^1†^, Lingwan Xie^1†^, Zhen Fang^1†^, Yueyue Li^1^, Chaemin Yoon^2,3^, Qiao Huang^4^, Bingjie Hu^2,3^, Jing You^1^, Baoxue Quan^1^, Xiu Zhao^1^, Nihong Guo^4^, Shiyu Zhang^4^, Ronggang Ma^1^, Jiahao Zhang^1^, Yifei Wang^1^, Ruicheng Yang^1^, Shanshan Zhang^1^, Jinshan Nan^1^, Haixing Xu^1^, Falu Wang^1^, Jian Lei^1^*, Hin Chu^2,3^*, and Shengyong Yang^1^*

*Corresponding author. Email: Shengyong Yang, [yangsy@scu.edu.cn](mailto:yangsy@scu.edu.cn); Hin Chu, [hinchu@hku.hk](mailto:hinchu@hku.hk); Jian Lei, [leijian@scu.edu.cn](mailto:leijian@scu.edu.cn)

**This PDF file includes:**

Materials and Methods

Supplementary Fig. S1

Supplementary Tables S1 to S11

Supplementary references

**Materials and Methods**

**General Methods for Compound Synthesis/Analysis**

Dichloromethane, ethanol, methanol, petroleum ether, ethyl acetate and 1,4-dioxane were obtained from standard suppliers and used without further purification. Anhydrous solvents were purchased from TCI AMERICA or Alfa Chemistry. All chemicals were purchased from Bidepharm Technology Co.Ltd, Energy Chemical or Adamas-beta^®^ and used without further purification. All reactions were monitored by thin layer chromatography (TLC) and visualization was achieved by using ultraviolet light (254 nm) or displayed by iodine reagent and ninhydrin reagent. Column chromatography was carried out using Biotage Isolera flash purification system under proper pressure. NMR spectra were recorded on a Bruker Ascend 400 MHz spectrometer or a Bruker Ascend 600 MHz spectrometer using deuterated DMSO as solvent. Coupling constants (*J*) are expressed in hertz (Hz). Chemical shifts (*δ*) of NMR are reported in parts per million (ppm) units. Low-resolution ESI-MS readings were recorded on an Agilent 1200-G6410A mass spectrometer. High-resolution mass spectra were recorded on Q-TOF Premier mass spectrometer (Micromass, Manchester, UK).

**Synthesis of M^pro^ Inhibitors**

**Scheme 1. Synthesis of compound 1a.*^a^***

*^a^* Reaction conditions: (i) SOCl_2_, MeOH, 0-25°C, 4 h; (ii) HATU, DIEA, CH2Cl2, 25°C, 6 h; (iii) LiOH (aq., 1M), MeOH/H_2_O, 0°C, 3 h; (iv) HATU, DIEA, CH2Cl2, 25°C, 6 h; (v) Dess-martin reagent, dry CH2Cl2, 0°C, 2 h.

**Scheme 2. Synthesis of compound 2c.*^a^***

*^a^* Reaction conditions: (i) HATU, DIEA, CH2Cl2, 25°C, 6 h; (ii) LiAlH_4_ in THF (1.0 M), dry THF, 0°C, 1h; (iii) Ethyl formate, 80°C, 6 h; (iv) Burgess reagent, dry CH2Cl2, 25°C, overnight; (v) CH_3_COOH, CH2Cl2, 25°C, overnight; (vi) LiOH (aq., 1M), MeOH/H_2_O, 0°C, 3 h; (vii) HCl in 1,4-Dioxane (4M), CH2Cl2, 25°C, overnight; (viii) HATU, DIEA, CH2Cl2, 25°C, 6 h; (ix) Dess-martin reagent, dry CH2Cl2, 0°C, 2 h.

**Scheme 3. Synthesis of compound 3a.*^a^***

*^a^* Reaction conditions: (i) HATU, DIEA, CH2Cl2, 25°C, 6 h; (ii) HCl in 1,4-Dioxane (4M), CH2Cl2, 25°C, overnight; (iii) HATU, DIEA, CH2Cl2, 25°C, 6 h; (iv) Dess-martin reagent, dry CH2Cl2, 0°C, 2 h.

**Scheme 4. Synthesis of compound 3w.*^a^***

*^a^* Reaction conditions: (i) HATU, DIEA, CH2Cl2, 25°C, 6 h; (ii) HCl in 1,4-Dioxane (4M), CH2Cl2, 25°C, overnight; (iii) HATU, DIEA, CH2Cl2, 25°C, 6 h; (iv) Dess-martin reagent, dry CH2Cl2, 0°C, 2 h.

***General procedure A (Compounds 1a-j were prepared with various heterocyclic methylamine derivatives using General procedure A), in which the synthesis of compound 1a is shown below:***

***methyl (2S,3R)-3-amino-2-hydroxy-4-phenylbutanoate hydrochloride* (T1)**

To a solution of (2*S*,3*R*)-3-((tert-butoxycarbonyl)amino)-2-hydroxy-4-phenylbutanoic acid (590 mg, 2 mmol) in MeOH at ice cold condition, 0.3 ml SOCl_2_ was added. The reaction mixture was stirred for 4 h at 25 °C, whereupon it was concentrated under reduced pressure. The trituration of the residue with CH2Cl2 provided the **T1** as a white solid, which was used in the next step without further purification. MS (ESI) (m/z): 210.1 [M + H] ^+^.

***methyl (2S,3R)-3-benzamido-2-hydroxy-4-phenylbutanoate* (T2)**

HATU (228 mg, 0.6 mmol) and DIEA (257 μl, 1.5 mmol) were added to a 25 °C solution of **T1** (132 mg, 0.5 mmol) and benzoic acid (61 mg, 0.5 mmol) in dry CH2Cl2 (10 ml). After the mixture stirred for 6 h, it was diluted with CH2Cl2, washed sequentially with saturated ammonium chloride solution, saturated sodium bicarbonate solution, and saturated aqueous sodium chloride solution, dried over anhydrous sodium sulfate, filtered, and concentrated. Chromatography on silica gel given **T2** as a white solid (125 mg, 80%). ^1^H NMR (400 MHz, DMSO-d6) δ 8.10 (d, *J* = 9.0 Hz, 1H), 7.77 – 7.70 (m, 2H), 7.54 – 7.47 (m, 1H), 7.47 – 7.38 (m, 2H), 7.28 (h, *J* = 6.2 Hz, 4H), 7.20 – 7.15 (m, 1H), 5.66 (dd, *J* = 7.1, 1.6 Hz, 1H), 4.57 – 4.46 (m, 1H), 4.15 (dd, *J* = 7.5, 2.9 Hz, 1H), 3.56 (s, 3H), 2.98 (dd, *J* = 13.7, 6.2 Hz, 1H), 2.89 (dd, *J* = 13.3, 8.7 Hz, 1H). MS (ESI) (m/z): 314.0 [M + H] ^+^.

***(2S,3R)-3-benzamido-2-hydroxy-4-phenylbutanoic acid (T3)***

The intermediate **T2** (125 mg, 0.4 mmol) obtained in the previous step was dissolved in MeOH/H_2_O (2 ml: 2 ml), and a solution of LiOH (1M; 1.2 ml, 1.2 mmol) was added. The reaction mixture was stirred under 0°C for 3 h. After the reaction completed, added 10 ml of water to the reaction solution, adjusted the pH to 1 with hydrochloric acid, and extracted with ethyl acetate (10 ml × 3). The combined the solvents, then dried over anhydrous Na2SO4 and concentrated under reduced pressure. Obtained 114 mg of crude product, and used in the next step without further purification. MS (ESI) (m/z): 300.1 [M + H] ^+^.

***N-((2R,3S)-3-hydroxy-4-oxo-1-phenyl-4-((thiazol-2-ylmethyl)amino)butan-2-yl)benzamide* (T4)**

To a solution of **T3** (114 mg, 0.38 mmol) in dry CH2Cl2, thiazol-2-ylmethanamine (46 mg, 0.4 mmol), HATU (217 mg, 0.57 mmol), DIEA (228 μl, 1.33 mmol) was added. The reaction mixture was stirred at 25 °C for 6 h. After the reaction completed, the mixture was washed with saturated NH_4_Cl solution, saturated NaHCO_3_ solution and brine, then dried over anhydrous Na2SO4 and concentrated under reduced pressure. The residue was purified with Biotage flash column chromatography to afford **T4** as a white solid (123 mg, 82%). ^1^H NMR (400 MHz, DMSO-*d*_6_) δ 8.83 (t, *J* = 6.2 Hz, 1H), 7.91 (d, *J* = 8.9 Hz, 1H), 7.76 – 7.71 (m, 2H), 7.63 (d, *J* = 3.2 Hz, 1H), 7.53 – 7.46 (m, 2H), 7.43 (t, *J* = 7.5 Hz, 2H), 7.32 – 7.24 (m, 4H), 7.20 – 7.14 (m, 1H), 6.29 – 6.13 (m, 1H), 4.65 – 4.55 (m, 1H), 4.53 – 4.44 (m, 2H), 4.09 – 4.05 (m, 1H), 2.99 (dd, *J* = 13.5, 6.2 Hz, 1H), 2.89 – 2.83 (m, 1H).MS (ESI) (m/z): 396.1 [M + H] ^+^.

***(R)-N-(3,4-dioxo-1-phenyl-4-((thiazol-2-ylmethyl)amino)butan-2-yl)benzamide* (1a)**

To a solution of **T4** (123 mg, 0.31 mmol) in dry CH2Cl2 (10 ml) was added Dess-Martin Periodinane (158 mg, 0.37 mmol). The reaction mixture was stirred at 0 °C for 2 h and quenched with sodium thiosulfate solution. The mixture was extracted with CH2Cl2 (30 ml ×2), the combined organic layer was washed with saturated NaHCO3 and brine, then dried over Na2SO4 and concentrated under reduced pressure. The residue was purified with Biotage flash column chromatography to give product **1a** (80 mg, 65%) as a white solid. ^1^H NMR (400 MHz, DMSO-*d*_6_) δ 9.61 (t, *J* = 6.2 Hz, 1H), 8.93 (d, *J* = 7.2 Hz, 1H), 7.80 (d, *J* = 7.5 Hz, 2H), 7.72 (t, *J* = 2.8 Hz, 1H), 7.61 (t, *J* = 2.7 Hz, 1H), 7.58 – 7.52 (m, 1H), 7.48 (d, *J* = 7.4 Hz, 2H), 7.38 – 7.27 (m, 4H), 7.22 (t, *J* = 7.8 Hz, 1H), 5.42 – 5.31 (m, 1H), 4.69 – 4.60 (m, 2H), 3.29 – 3.21 (m, 1H), 3.08 – 2.95 (m, 1H). ^13^C NMR (101 MHz, DMSO) δ 196.48, 168.17, 167.08, 161.68, 142.66, 138.35, 133.82, 132.04, 129.46, 128.80, 128.75, 127.94, 126.97, 120.75, 56.98, 40.87, 34.95. HRMS (ESI-TOF) *m/z* calcd. for C_21_H_19_N_3_O_3_S [M + H] ^+^ 394.1220, found 394.1212.

***General procedure B (Compounds 2a-g were prepared with various amino acid derivatives using General procedure B), in which the synthesis of compound 2c is shown below:***

***tert-butyl (R)-(3-(4-fluorophenyl)-1-(methoxy(methyl)amino)-1-oxopropan-2-yl)carbamate* (T5)**

To a solution of (*R*)-2-((tert-butoxycarbonyl)amino)-3-(4-fluorophenyl)propanoic acid (566 mg, 2 mmol) in dry CH2Cl2, *N,O*-dimethylhydroxylamine hydrochloride (214 mg, 2.2 mmol), HATU (1.14 g, 3 mmol), DIEA (1.03 ml, 6 mmol) was added. The reaction mixture was stirred under 25 °C for 6 h. After the reaction completed, the mixture was diluted with CH2Cl2 and washed with saturated NH_4_Cl, saturated NaHCO_3_ and brine, then dried over anhydrous Na2SO4 and concentrated under reduced pressure. The residue was purified with Biotage flash column chromatography to afford **T5** as a light yellow oil (554 mg, 85%). ^1^H NMR (400 MHz, DMSO-*d*_6_) δ 7.30 – 7.24 (m, 2H), 7.17 (d, *J* = 8.6 Hz, 1H), 7.10 (t, *J* = 8.7 Hz, 2H), 4.57 – 4.47 (m, 1H), 3.72 (s, 3H), 3.10 (s, 3H), 2.88 – 2.81 (m, 1H), 2.76 – 2.65 (m, 1H), 1.31 (s, 9H). MS (ESI) (m/z): 327.2 [M + H] ^+^.

***tert-butyl (R)-(1-(4-fluorophenyl)-3-oxopropan-2-yl)carbamate*(T6)**

The intermediate **T5** (554 mg, 1.7 mmol) obtained in the previous step was dissolved in THF under 0°C, and a solution of LiAlH_4_ in THF (1.0 M; 0.85 ml, 0.85 mmol) was added dropwise. The mixture was stirred for 1 h under nitrogen atmosphere. After the reaction was complete, it was quenched with a small amount of water. After concentrated under reduced pressure, the residue was purified with Biotage flash column chromatography to given **T6** as an oil (331 mg, 73%). MS (ESI) m/z: 268.1 [M + H] ^+^.

***N-(thiazol-2-ylmethyl)formamide*(T7)**

A suspension of (2-thiazolyl)methylamine(1.0 g, 8.76 mmol) in ethyl formate (10 ml) was refluxed for 6 hours and then cooled to room temperature, the solvent was removed under reduced pressure to give the residue, and purified with column chromatography to obtain **T7** as brown oil (1.18 g, 95%). ^1^H NMR (400 MHz, DMSO-*d*_6_) δ 8.87 (s, 1H), 8.25 – 8.19 (m, 1H), 7.74 (d, *J* = 3.3 Hz, 1H), 7.63 (d, *J* = 3.3 Hz, 1H), 4.62 (dd, *J* = 6.2, 0.7 Hz, 2H). MS (ESI) m/z: 143.0 [M + H] ^+^.

***2-(isocyanomethyl)thiazole*(T8)**

To a solution of *N*-(thiazol-2-ylmethyl)formamide (231 mg, 1.86 mmol) in 10 mL anhydrous CH2Cl2, Burgess reagent (213 mg, 1.86 mmol) was added. The mixture was stirred at room temperature overnight, and it can be used directly for the next step. MS (ESI) m/z: 125.0 [M + H] ^+^.

***(3R)-3-((tert-butoxycarbonyl)amino)-4-(4-fluorophenyl)-1-oxo-1-((thiazol-2-ylmethyl)amino)butan-2-yl acetate*(T9)**

To a solution of aldehyde **T6** (331 mg, 1.24 mmol) and 2-(isocyanomethyl)thiazol (230 mg, 1.86 mmol) in anhydrous CH2Cl2 (10 ml) was added acetic acid (112 mg, 1.86 mmol). The reaction mixture was stirred at 25 ^o^C overnight. When reaction completed, reaction mixture was poured into NaHCO_3_ (aq., 20 mL) and extracted with CH2Cl2 (20 ml × 2). The combined organic layers were dried over Na_2_SO_4_ and concentrated under reduced pressure, the residue was purified by Biotage flash column chromatography to give product **T9** (296 mg, 53%) as white solid. MS (ESI) m/z: 452.2 [M + H] ^+^.

***tert-butyl ((2R)-1-(4-fluorophenyl)-3-hydroxy-4-oxo-4-((thiazol-2-ylmethyl)amino)butan-2-yl)carbamate*(T10)**

The intermediate **T9** (296 mg, 0.66 mmol) obtained in the previous step was dissolved in MeOH/H_2_O (2ml: 2ml), and a solution of LiOH (1M; 1.64 ml, 1.64 mmol) was added. The reaction mixture was stirred under 0 °C for 3 h. After the reaction completed, added 10ml of water to the reaction solution, adjusted the pH to 1 with hydrochloric acid, and extracted with ethyl acetate (3 × 10ml). The combined the solvents, then dried over anhydrous Na2SO4 and concentrated under reduced pressure. Obtained 114 mg of crude product, and used in the next step without further purification. MS (ESI) (m/z): 410.1 [M + H] ^+^.

***(3R)-3-amino-4-(4-fluorophenyl)-2-hydroxy-N-(thiazol-2-ylmethyl)butanamide hydrochloride*(T11)**

The intermediate **T10** obtained in the previous step was dissolved in CH2Cl2, and a solution of HCl in 1,4-dioxane (4 M; 653 μl, 2.61 mmol) was added. The mixture was stirred overnight under the nitrogen atmosphere. After concentrated under reduced pressure, the residue was treated with CH2Cl2 provided the **T11** as a brown solid, which was used in the next step without further purification. MS (ESI) (m/z): 310.1 [M + H] ^+^.

***N-((2R)-1-(4-fluorophenyl)-3-hydroxy-4-oxo-4-((thiazol-2-ylmethyl)amino)butan-2-yl)benzamide*(T12)**

HATU (238 mg, 0.63 mmol) and DIEA (253 μl, 1.46 mmol) were added to a 25 °C solution of **T11** (146 mg, 0.42 mmol) and benzoic acid (51mg, 0.42 mmol) in dry CH2Cl2 (10 ml). After the mixture stirred for 6 h, it was diluted with CH2Cl2, washed sequentially with saturated NH_4_Cl, saturated NaHCO_3_ and brine, dried with sodium sulfate, filtered, and concentrated. Chromatography on silica gel given **T12** as a white solid (114 mg, 66%). ^1^H NMR (400 MHz, DMSO-*d*_6_) δ 8.85 (t, *J* = 6.2 Hz, 1H), 8.26 (d, *J* = 8.6 Hz, 1H), 7.83 – 7.76 (m, 2H), 7.70 (d, *J* = 3.3 Hz, 1H), 7.60 (d, *J* = 3.3 Hz, 1H), 7.53 – 7.48 (m, 1H), 7.43 (dd, *J* = 8.2, 6.6 Hz, 2H), 7.25 (dd, *J* = 8.5, 5.6 Hz, 2H), 7.05 (t, *J* = 8.8 Hz, 2H), 6.15 (d, *J* = 5.9 Hz, 1H), 4.72 – 4.65 (m, 1H), 4.60 – 4.49 (m, 2H), 4.26 – 4.19 (m, 1H), 2.99 – 2.92 (m, 1H), 2.73 – 2.67 (m, 1H). MS (ESI) m/z: 414.1 [M + H] ^+^.

***(R)-N-(1-(4-fluorophenyl)-3,4-dioxo-4-((thiazol-2-ylmethyl)amino)butan-2-yl)benzamide*(2c)**

To a solution of **T12** (114 mg, 0.275 mmol) in dry CH2Cl2 (10 mL) was added Dess-Martin Periodinane (140 mg, 0.33mmol). The reaction mixture was stirred at 0 °C for 2 h and quenched with sodium thiosulfate solution. The mixture was extracted with CH2Cl2 (30 ml × 2), the combined organic layer was washed with saturated NaHCO3 and brine, then dried over Na2SO4 and concentrated under reduced pressure. The residue was purified with Biotage flash column chromatography to provide product **2c** (68 mg, 60%) as a white solid. ^1^H NMR (400 MHz, DMSO-*d*_6_) δ 9.58 (t, *J* = 6.2 Hz, 1H), 8.93 (d, *J* = 7.1 Hz, 1H), 7.80 – 7.76 (m, 2H), 7.71 (d, *J* = 3.3 Hz, 1H), 7.60 (d, *J* = 3.2 Hz, 1H), 7.56 – 7.52 (m, 1H), 7.46 (dd, *J* = 8.1, 6.6 Hz, 2H), 7.37 – 7.32 (m, 2H), 7.15 – 7.09 (m, 2H), 5.34 – 5.25 (m, 1H), 4.63 (d, *J* = 6.3 Hz, 2H), 3.26 – 3.18 (m, 1H), 3.02 – 2.93 (m, 1H). ^13^C NMR (101 MHz, DMSO) δ 196.38, 168.15, 167.07, 162.71, 161.69, 160.30, 142.65, 134.46, 134.43, 133.77, 132.07, 131.36, 131.28, 128.76, 127.93, 120.74, 115.63, 115.42, 56.94, 40.86, 34.19. HRMS (ESI-TOF) *m/z* calcd. for C_21_H_18_FN_3_O_3_S [M + H] ^+^ 412.1126, found 412.1131.

***General procedure C (Compounds 3a-f were prepared with acid derivatives using General procedure C), in which the synthesis of compound 3a is shown below:***

***tert-butyl ((2R,3S)-3-hydroxy-4-oxo-1-phenyl-4-((thiazol-2-ylmethyl)amino)butan-2-yl)carbamate* (T13)**

To a solution of (2*S*, 3*R*)-3-((tert-butoxycarbonyl)amino)-2-hydroxy-4-phenylbutanoic acid (590 mg, 2 mmol) in dry CH_2_Cl_2_, thiazol-2-ylmethanamine (228 mg, 2 mmol), HATU (1.14 g, 3 mmol), DIEA (1.03 ml, 6 mmol) was added. The reaction mixture was stirred under 25 °C for 6 h. After the reaction completed, the mixture was diluted with CH_2_Cl_2_ and washed with saturated NH_4_Cl, saturated NaHCO_3_ and brine, then dried over anhydrous Na_2_SO_4_ and concentrated under reduced pressure. The residue was purified with Biotage flash column chromatography to afford **T13** as a light yellow oil (665 mg, 85%). ^1^H NMR (400 MHz, DMSO-*d*_6_) δ 8.74 (t, *J* = 6.2 Hz, 1H), 7.69 (d, *J* = 3.3 Hz, 1H), 7.58 (d, *J* = 3.3 Hz, 1H), 7.33 – 7.12 (m, 5H), 6.13 (d, *J* = 9.3 Hz, 1H), 5.94 (d, *J* = 6.4 Hz, 1H), 4.64 – 4.46 (m, 2H), 4.06 – 3.95 (m, 1H), 3.95 – 3.87 (m, 1H), 2.87 – 2.77 (m, 1H), 2.75 – 2.64 (m, 1H), 1.29 (s, 9H). MS (ESI) (m/z): 392.2 [M + H] ^+^.

***(2S,3R)-3-amino-2-hydroxy-4-phenyl-N-(thiazol-2-ylmethyl)butanamide hydrochloride*(T14)**

The intermediate **T13** (587 mg, 1.5 mmol) obtained in the previous step was dissolved in CH2Cl2, and a solution of HCl in 1,4-dioxane (4 M; 1.87 ml, 7.5 mmol) was added. The mixture was stirred overnight under the nitrogen atmosphere. After concentrated under reduced pressure, the residue was treated with CH2Cl2 provided the **T14** as a white solid, which was used in the next step without further purification. MS (ESI) (m/z): 292.1 [M + H] ^+^.

***2-fluoro-N-((2R,3S)-3-hydroxy-4-oxo-1-phenyl-4-((thiazol-2-ylmethyl)amino)butan-2-yl)benzamide*(T15)**

To a solution of **T14** (327 mg, 1.0 mmol) in dry CH2Cl2, 2-fluorobenzoic acid (140 mg, 1.0 mmol), HATU (495 mg, 1.3 mmol), DIEA (522 μl, 3.0 mmol) was added. The reaction mixture was stirred at 25 °C for 6 h. After the reaction completed, the mixture was washed with saturated NH_4_Cl solution, saturated NaHCO_3_ solution and brine, then dried over anhydrous Na2SO4 and concentrated under reduced pressure. The residue was purified with Biotage flash column chromatography to afford **T15** as a white solid (322 mg, 78%).^1^H NMR (400 MHz, DMSO-*d*_6_) δ 8.81 (t, *J* = 6.2 Hz, 1H), 7.77 (dd, *J* = 9.0, 6.2 Hz, 1H), 7.62 (d, *J* = 3.3 Hz, 1H), 7.60 (dd, *J* = 7.6, 1.9 Hz, 1H), 7.56 – 7.50 (m, 1H), 7.46 (d, *J* = 3.3 Hz, 1H), 7.31 – 7.23 (m, 6H), 7.22 – 7.18 (m, 1H), 6.34 (d, *J* = 5.9 Hz, 1H), 4.63 – 4.44 (m, 3H), 4.04 (dd, *J* = 5.9, 2.8 Hz, 1H), 3.03 – 2.90 (m, 1H), 2.85 – 2.76 (m, 1H). MS (ESI) m/z: 414.2 [M + H] ^+^.

***(R)-N-(3,4-dioxo-1-phenyl-4-((thiazol-2-ylmethyl)amino)butan-2-yl)-2-fluorobenzamide*(3a)**

To a solution of **T15** (41 mg, 0.1 mmol) in 10 ml dry CH2Cl2 was added Dess-Martin Periodinane (51 mg, 0.12 mmol). The reaction mixture was stirred at 0 °C for 2 h and quenched with sodium thiosulfate solution. Mixture was extracted with CH2Cl2 (30 ml × 2), the combined organic layer was washed with saturated NaHCO3 and brine, then dried over Na2SO4 and concentrated under reduced pressure. The residue was purified with Biotage flash column chromatography to provide product **3a** (27.5 mg, 67%) as a white solid. ^1^H NMR (400 MHz, DMSO-*d*_6_) δ 9.64 (t, *J* = 6.3 Hz, 1H), 8.75 (dd, *J* = 7.3, 2.4 Hz, 1H), 7.74 (d, *J* = 3.3 Hz, 1H), 7.64 (d, *J* = 3.0 Hz, 1H), 7.58 – 7.47 (m, 2H), 7.34 – 7.20 (m, 7H), 5.44 – 5.34 (m, 1H), 4.66 (d, *J* = 6.3 Hz, 2H), 3.29 – 3.18 (m, 1H), 3.01 – 2.89 (m, 1H). ^13^C NMR (101 MHz, DMSO) δ 195.90, 168.08, 164.36, 161.45, 160.94, 158.46, 142.67, 137.89, 133.32, 133.23, 130.59, 130.56, 129.53, 128.80, 127.08, 124.91, 124.88, 123.51, 123.37, 120.81, 116.75, 116.53, 56.90, 40.90, 35.21. HRMS (ESI-TOF) *m/z* calcd. for C_21_H_18_FN_3_O_3_S [M + H] ^+^ 412.1126, found 412.1119.

***General procedure D (Compounds 3g-y were prepared with various amino acid derivatives and acid derivatives using General procedure D), in which the synthesis of compound 3w is shown below:***

***tert-butyl ((R)-1-(((2R,3S)-3-hydroxy-4-oxo-1-phenyl-4-((thiazol-2-ylmethyl)amino)butan-2-yl)amino)-3-methoxy-1-oxopropan-2-yl)carbamate*(T16)**

HATU (572 mg, 1.5 mmol) and DIEA (520 μl, 3.0 mmol) were added to a 25 °C solution of **T14** (328 mg, 1.0 mmol) and *N*-(tert-butoxycarbonyl)-O-methyl-*D*-serine (220 mg, 1.0 mmol) in dry CH2Cl2 (10 ml). After the mixture stirred for 6 h, it was diluted with CH2Cl2, washed sequentially with saturated ammonium chloride solution, saturated sodium bicarbonate solution, and saturated sodium chloride solution, dried over sodium sulfate, filtered, and concentrated. Chromatography on silica gel given **T16** as an oil (385 mg, 78%). ^1^H NMR (400 MHz, DMSO-*d*_6_) δ 8.62 (t, *J* = 6.2 Hz, 1H), 7.69 (d, *J* = 3.2 Hz, 1H), 7.60 – 7.53 (m, 2H), 7.33 – 7.16 (m, 5H), 6.92 (d, *J* = 8.2 Hz, 1H), 6.28 (d, *J* = 5.7 Hz, 1H), 4.53 (d, *J* = 6.2 Hz, 2H), 4.30 – 4.21 (m, 1H), 4.14 – 4.04 (m, 1H), 3.90 (dd, *J* = 5.8, 2.5 Hz, 1H), 3.32 – 3.28 (m, 2H), 3.18 (s, 3H), 2.90 – 2.81 (m, 1H), 2.67 – 2.57 (m, 1H), 1.38 (s, 9H). MS (ESI) (m/z): 493.2 [M + H] ^+^.

***(2S,3R)-3-((R)-2-amino-3-methoxypropanamido)-2-hydroxy-4-phenyl-N-(thiazol-2-ylmethyl)butanamide hydrochloride*(T17)**

The intermediate **T16** (385 mg, 0.78 mmol) obtained in the previous step was dissolved in CH2Cl2, and a solution of HCl in 1,4-dioxane (4 M; 977 μl, 3.9 mmol) was added. The mixture was stirred overnight under the nitrogen atmosphere. After concentrated under reduced pressure, the residue was treated with CH2Cl2 provided the **T17** as a white solid, which was used in the next step without further purification. MS (ESI) (m/z): 393.2 [M + H] ^+^.

***3,3-difluoro-N-((R)-1-(((2R,3S)-3-hydroxy-4-oxo-1-phenyl-4-((thiazol-2-ylmethyl)amino)butan-2-yl)amino)-3-methoxy-1-oxopropan-2-yl)cyclohexane-1-carboxamide*(T18)**

To a solution of **T17** (214 mg, 0.5 mmol) in dry CH2Cl2, 3,3-difluorocyclohexane-1-carboxylic acid (82 mg, 0.5 mmol), HATU (285 mg, 0.75 mmol), DIEA (345 μl, 2 mmol) was added. The reaction mixture was stirred under 25 °C for 6 h. After the reaction completed, mixture was washed with saturated NH_4_Cl solution, saturated NaHCO_3_ solution and brine, then dried over anhydrous Na2SO4 and concentrated under reduced pressure. The residue was purified with Biotage flash column chromatography to afford **T18** as a white solid (215 mg, 80%).^1^H NMR (400 MHz, DMSO-*d*_6_) δ 8.70 – 8.57 (m, 1H), 8.18 (d, *J* = 8.0 Hz, 1H), 7.69 (t, *J* = 2.5 Hz, 1H), 7.61 – 7.48 (m, 2H), 7.36 – 7.14 (m, 5H), 6.33 – 6.20 (m, 1H), 4.61 – 4.47 (m, 2H), 4.42 – 4.34 (m, 1H), 4.29 – 4.18 (m, 1H), 3.94 – 3.85 (m, 1H), 3.41 – 3.34 (m, 2H), 2.91 – 2.80 (m, 1H), 2.69 – 2.57 (m, 1H), 2.13 – 1.93 (m, 2H), 1.89 – 1.61 (m, 4H), 1.50 – 1.19 (m, 3H). MS (ESI) (m/z): 539.2 [M + H] ^+^.

***N-((R)-1-(((R)-3,4-dioxo-1-phenyl-4-((thiazol-2-ylmethyl)amino)butan-2-yl)amino)-3-methoxy-1-oxopropan-2-yl)-3,3-difluorocyclohexane-1-carboxamide*(3w, SY110)**

To a solution of **T18** (54 mg, 0.1 mmol) in dry CH2Cl2 (10 ml) was added Dess-Martin Periodinane (51 mg, 0.12 mmol). The reaction mixture was stirred at 0 °C for 2 h and quenched with sodium thiosulfate solution. The mixture was extracted with CH2Cl2 (30 ml × 2), the combined organic layer was washed with saturated NaHCO3 and brine, then dried over Na2SO4 and concentrated under reduced pressure. The residue was purified with Biotage flash column chromatography to provide product **3w** (**SY110**; 32 mg, 60%) as a white solid. ^1^H NMR (400 MHz, DMSO-*d*_6_) δ 9.60 – 9.48 (m, 1H), 8.38 – 8.28 (m, 1H), 8.12 (d, *J* = 8.1 Hz, 1H), 7.73 (d, *J* = 3.3 Hz, 1H), 7.68 – 7.62 (m, 1H), 7.30 – 7.24 (m, 2H), 7.24 – 7.18 (m, 3H), 5.26 – 5.13 (m, 1H), 4.67 – 4.56 (m, 2H), 4.55 – 4.45 (m, 1H), 3.48 – 3.37 (m, 2H), 3.22 (d, *J* = 3.1 Hz, 3H), 3.18 – 3.09 (m, 1H), 2.94 – 2.85 (m, 1H), 2.06 – 1.90 (m, 2H), 1.87 – 1.63 (m, 4H), 1.47 – 1.35 (m, 1H), 1.33 – 1.21 (m, 1H). ^13^C NMR (101 MHz, DMSO) δ 195.81, 195.78, 173.49, 173.43, 170.22, 170.19, 168.17, 161.42, 161.32, 142.66, 137.58, 137.51, 129.59, 128.73, 127.14, 127.00, 124.77, 122.37, 120.78, 72.30, 58.62, 55.97, 52.73, 40.97, 40.87, 36.45, 36.40, 36.22, 36.15, 35.98, 35.92, 35.50, 35.45, 33.45, 33.22, 32.99, 28.04, 22.11, 22.01. HRMS (ESI-TOF) *m/z* calcd. for C_25_H_30_F_2_N_4_O_5_S [M + H] ^+^ 537.1978, found 537.1969. Melting point: 185.03 ℃.

**Characterization of M^pro^ Inhibitors**

| **Number** | **Structure** | **NMR** | **HRMS** |
| --- | --- | --- | --- |
| **1a** |  | ^1^H NMR (400 MHz, DMSO-*d*_6_) δ 9.61 (t, *J* = 6.2 Hz, 1H), 8.93 (d, *J* = 7.2 Hz, 1H), 7.80 (d, *J* = 7.5 Hz, 2H), 7.72 (t, *J* = 2.8 Hz, 1H), 7.61 (t, *J* = 2.7 Hz, 1H), 7.58 – 7.52 (m, 1H), 7.48 (d, *J* = 7.4 Hz, 2H), 7.38 – 7.27 (m, 4H), 7.22 (t, *J* = 7.8 Hz, 1H), 5.42 – 5.31 (m, 1H), 4.69 – 4.60 (m, 2H), 3.29 – 3.21 (m, 1H), 3.08 – 2.95 (m, 1H).  ^13^C NMR (101 MHz, DMSO) δ 196.48, 168.17, 167.08, 161.68, 142.66, 138.35, 133.82, 132.04, 129.46, 128.80, 128.75, 127.94, 126.97, 120.75, 56.98, 40.87, 34.95. | [M+H] ^+^  394.1212 |
| **1b** |  | ^1^H NMR (400 MHz, DMSO-*d*_6_) δ 9.38 (t, *J* = 6.4 Hz, 1H), 8.90 (d, *J* = 7.3 Hz, 1H), 7.78 (d, *J* = 7.6 Hz, 2H), 7.54 (t, *J* = 7.4 Hz, 1H), 7.46 (t, *J* = 7.6 Hz, 2H), 7.38 – 7.26 (m, 5H), 7.20 (t, *J* = 7.3 Hz, 1H), 6.99 – 6.96 (m, 1H), 6.93 (d, *J* = 4.4 Hz, 1H), 5.41 – 5.31 (m, 1H), 4.55 – 4.47 (m, 2H), 3.27 – 3.19 (m, 1H), 3.02 – 2.93 (m, 1H).  ^13^C NMR (101 MHz, DMSO) δ 196.88, 167.07, 161.25, 141.50, 138.44, 133.87, 132.02, 129.45, 128.79, 128.74, 127.92, 127.11, 126.95, 126.35, 125.69, 57.03, 37.63, 34.98. | [M+H] ^+^  393.1262 |
| **1c** |  | ^1^H NMR (400 MHz, DMSO-*d*_6_) δ 9.21 (t, *J* = 6.1 Hz, 1H), 8.89 (d, *J* = 7.2 Hz, 1H), 7.78 (d, *J* = 7.6 Hz, 2H), 7.57 – 7.51 (m, 2H), 7.46 (t, *J* = 7.7 Hz, 2H), 7.36 – 7.27 (m, 4H), 7.23 – 7.18 (m, 1H), 6.39 – 6.35 (m, 1H), 6.23 (d, *J* = 3.1 Hz, 1H), 5.38 – 5.30 (m, 1H), 4.38 – 4.29 (m, 2H), 3.22 (dd, *J* = 14.3, 3.6 Hz, 1H), 3.01 – 2.92 (m, 1H).  ^13^C NMR (101 MHz, DMSO) δ 196.84, 167.07, 161.35, 151.73, 142.61, 138.45, 133.87, 132.02, 129.44, 128.78, 128.75, 127.90, 126.94, 110.94, 107.66, 57.00, 35.96, 34.98. | [M+H] ^+^  377.1494 |
| **1d** |  | ^1^H NMR (400 MHz, DMSO-*d*_6_) δ 9.39 (t, *J* = 6.2 Hz, 1H), 8.89 (d, *J* = 7.4 Hz, 1H), 8.02 – 7.97 (m, 1H), 7.78 (d, *J* = 7.7 Hz, 2H), 7.58 – 7.50 (m, 1H), 7.46 (t, *J* = 7.5 Hz, 2H), 7.37 – 7.25 (m, 4H), 7.20 (t, *J* = 7.1 Hz, 1H), 7.17 – 7.10 (m, 1H), 5.34 (d, *J* = 9.5 Hz, 1H), 4.52 – 4.42 (m, 2H), 3.28 – 3.19 (m, 1H), 3.03 – 2.90 (m, 1H). | [M+H] ^+^  378.1445 |
| **1e** |  | ^1^H NMR (400 MHz, DMSO-*d*_6_) δ 9.15 (t, *J* = 6.0 Hz, 1H), 8.89 (d, *J* = 7.2 Hz, 1H), 8.30 (s, 1H), 7.87 (s, 1H), 7.78 (dt, *J* = 7.1, 1.4 Hz, 2H), 7.57 – 7.50 (m, 1H), 7.46 (dd, *J* = 8.4, 7.0 Hz, 2H), 7.36 – 7.24 (m, 4H), 7.24 – 7.17 (m, 1H), 5.41 – 5.30 (m, 1H), 4.31 – 4.22 (m, 2H), 3.26 – 3.20 (m, 1H), 3.01 – 2.91 (m, 1H).  ^13^C NMR (101 MHz, DMSO) δ 196.76, 167.06, 161.41, 152.52, 138.45, 137.26, 136.57, 133.84, 132.04, 129.45, 128.77, 127.89, 126.93, 56.89, 35.20, 35.02. | [M+H] ^+^  378.1444 |
| **1f** |  | ^1^H NMR (400 MHz, DMSO-*d*_6_) δ 9.34 (t, *J* = 6.2 Hz, 1H), 8.92 (d, *J* = 7.1 Hz, 1H), 8.81 (d, *J* = 1.8 Hz, 1H), 7.81 – 7.74 (m, 2H), 7.58 – 7.49 (m, 1H), 7.50 – 7.39 (m, 2H), 7.37 – 7.24 (m, 4H), 7.25 – 7.16 (m, 1H), 6.42 (t, *J* = 1.9 Hz, 1H), 5.38 – 5.26 (m, 1H), 4.45 – 4.37 (m, 2H), 3.27 – 3.17 (m, 1H), 3.03 – 2.91 (m, 1H).  ^13^C NMR (101 MHz, DMSO) δ 196.55, 167.07, 161.62, 160.67, 160.42, 138.37, 133.79, 132.05, 129.47, 128.78, 128.76, 127.91, 126.95, 104.48, 56.85, 35.03, 34.66. | [M+H] ^+^  378.1447 |
| **1g** |  | ^1^H NMR (400 MHz, DMSO-*d*_6_) δ 9.56 (s, 1H), 9.43 (t, *J* = 5.9 Hz, 1H), 8.88 (d, *J* = 7.3 Hz, 1H), 7.79 (d, *J* = 7.6 Hz, 2H), 7.54 (t, *J* = 7.3 Hz, 1H), 7.46 (t, *J* = 7.5 Hz, 2H), 7.37 – 7.27 (m, 4H), 7.21 (t, *J* = 7.1 Hz, 1H), 5.42 – 5.34 (m, 1H), 4.56 – 4.52 (m, 5H), 3.28 – 3.20 (m, 1H), 3.01 – 2.92 (m, 1H).  ^13^C NMR (101 MHz, DMSO) δ 196.37, 167.64, 167.09, 167.03, 161.53, 138.42, 133.91, 132.02, 129.42, 128.80, 128.75, 127.91, 126.97, 57.03, 34.92, 34.86. | [M+H] ^+^  379.1399 |
| **1h** |  | ^1^H NMR (400 MHz, DMSO-*d*_6_) δ 9.55 (t, *J* = 6.0 Hz, 1H), 8.94 (s, 1H), 8.92 (d, *J* = 7.1 Hz, 1H), 7.81 – 7.76 (m, 2H), 7.54 (td, *J* = 7.2, 1.5 Hz, 1H), 7.49 – 7.44 (m, 2H), 7.36 – 7.26 (m, 4H), 7.23 – 7.18 (m, 1H), 5.37 – 5.29 (m, 1H), 4.72 – 4.65 (m, 2H), 3.27 – 3.20 (m, 1H), 3.02 – 2.94 (m, 1H). | [M+H] ^+^  379.1396 |
| **1i** |  | ^1^H NMR (400 MHz, DMSO-*d*_6_) δ 9.22 (t, *J* = 6.2 Hz, 1H), 8.93 (d, *J* = 7.1 Hz, 1H), 7.85 – 7.76 (m, 2H), 7.54 (dd, *J* = 8.3, 6.4 Hz, 1H), 7.46 (t, *J* = 7.5 Hz, 2H), 7.31 (dt, *J* = 14.8, 7.4 Hz, 4H), 7.20 (t, *J* = 7.0 Hz, 1H), 7.10 (s, 1H), 5.38 – 5.28 (m, 1H), 4.48 – 4.31 (m, 2H), 3.29 – 3.21 (m, 1H), 3.04 – 2.93 (m, 1H), 2.60 (s, 3H).  ^13^C NMR (101 MHz, DMSO) δ 196.89, 167.04, 165.87, 161.53, 152.90, 138.47, 133.80, 132.05, 129.48, 128.77, 127.91, 126.92, 114.99, 56.98, 35.05, 19.16. | [M+H] ^+^  408.1371 |
| **1j** |  | ^1^H NMR (400 MHz, DMSO-*d*_6_) δ 9.42 (t, *J* = 6.1 Hz, 1H), 8.97 (d, *J* = 6.9 Hz, 1H), 7.76 (d, *J* = 7.6 Hz, 2H), 7.53 (d, *J* = 7.3 Hz, 2H), 7.45 (t, *J* = 7.5 Hz, 2H), 7.36 – 7.25 (m, 4H), 7.24 – 7.15 (m, 3H), 5.28 – 5.20 (m, 1H), 4.51 – 4.38 (m, 2H), 3.22 (dd, *J* = 13.8, 4.4 Hz, 1H), 3.07 – 2.95 (m, 1H).  ^13^C NMR (101 MHz, DMSO) δ 196.49, 167.07, 161.80, 150.37, 140.23, 138.92, 138.29, 133.70, 132.05, 128.76, 128.74, 127.88, 126.92, 56.88, 35.53, 35.07. | [M+H] ^+^  428.0826 |
| **2a** |  | ^1^H NMR (400 MHz, DMSO-*d*_6_) δ 9.53 (t, *J* = 6.3 Hz, 1H), 8.98 (d, *J* = 7.0 Hz, 1H), 7.76 (d, *J* = 7.6 Hz, 2H), 7.69 (d, *J* = 3.3 Hz, 1H), 7.58 (d, *J* = 3.3 Hz, 1H), 7.54 (t, *J* = 7.5 Hz, 1H), 7.45 (t, *J* = 7.6 Hz, 2H), 7.37 (t, *J* = 7.7 Hz, 1H), 7.26 (t, *J* = 6.9 Hz, 1H), 7.17 – 7.08 (m, 2H), 5.34 – 5.24 (m, 1H), 4.64 – 4.53 (m, 2H), 3.30 – 3.26 (m, 1H), 3.11 – 3.03 (m, 1H).  ^13^C NMR (101 MHz, DMSO) δ 196.22, 168.26, 167.04, 162.46, 161.92, 160.03, 142.61, 133.77, 132.13, 132.08, 132.03, 129.24, 129.16, 128.72, 127.92, 124.81, 124.70, 124.66, 120.68, 115.70, 115.48, 55.19, 40.83, 28.86. | [M+H] ^+^  412.1132 |
| **2b** |  | ^1^H NMR (400 MHz, DMSO-*d*_6_) δ 9.59 (t, *J* = 6.2 Hz, 1H), 8.97 (d, *J* = 7.1 Hz, 1H), 7.82 – 7.75 (m, 2H), 7.71 (d, *J* = 3.2 Hz, 1H), 7.60 (d, *J* = 3.1 Hz, 1H), 7.57 – 7.52 (m, 1H), 7.50 – 7.44 (m, 2H), 7.37 – 7.30 (m, 1H), 7.19 – 7.12 (m, 2H), 7.08 – 7.01 (m, 1H), 5.37 – 5.26 (m, 1H), 4.68 – 4.59 (m, 2H), 3.31 – 3.23 (m, 1H), 3.09 – 2.98 (m, 1H).  ^13^C NMR (101 MHz, DMSO) δ 196.18, 168.16, 167.09, 163.71, 161.73, 161.29, 142.64, 141.25, 141.17, 133.73, 132.09, 130.70, 130.61, 128.77, 127.91, 125.64, 125.61, 120.73, 116.37, 116.16, 113.91, 113.70, 56.63, 40.86, 34.67. | [M+H] ^+^  412.1123 |
| **2c** |  | ^1^H NMR (400 MHz, DMSO-*d*_6_) δ 9.58 (t, *J* = 6.2 Hz, 1H), 8.93 (d, *J* = 7.1 Hz, 1H), 7.80 – 7.76 (m, 2H), 7.71 (d, *J* = 3.3 Hz, 1H), 7.60 (d, *J* = 3.2 Hz, 1H), 7.56 – 7.52 (m, 1H), 7.46 (dd, *J* = 8.1, 6.6 Hz, 2H), 7.37 – 7.32 (m, 2H), 7.15 – 7.09 (m, 2H), 5.34 – 5.25 (m, 1H), 4.63 (d, *J* = 6.3 Hz, 2H), 3.26 – 3.18 (m, 1H), 3.02 – 2.93 (m, 1H).  ^13^C NMR (101 MHz, DMSO) δ 196.38, 168.15, 167.07, 162.71, 161.69, 160.30, 142.65, 134.46, 134.43, 133.77, 132.07, 131.36, 131.28, 128.76, 127.93, 120.74, 115.63, 115.42, 56.94, 40.86, 34.19. | [M+H] ^+^  412.1131 |
| **2d** |  | ^1^H NMR (400 MHz, DMSO-*d*_6_) δ 9.56 (t, *J* = 6.2 Hz, 1H), 9.08 (d, *J* = 6.8 Hz, 1H), 7.83 – 7.77 (m, 2H), 7.74 – 7.72 (m, 1H), 7.63 – 7.57 (m, 2H), 7.54 – 7.48 (m, 4H), 5.29 – 5.21 (m, 1H), 4.67 – 4.58 (m, 2H), 3.33 – 3.21 (m, 1H), 3.17 – 3.07 (m, 1H).  ^13^C NMR (101 MHz, DMSO) δ 195.78, 168.26, 167.08, 161.93, 157.79, 157.66, 155.34, 155.22, 149.84, 149.70, 149.57, 147.39, 147.27, 147.11, 145.04, 144.88, 142.60, 133.60, 132.11, 128.77, 127.88, 122.14, 122.10, 122.04, 121.96, 121.91, 121.86, 120.61, 119.96, 119.90, 119.77, 119.71, 106.35, 106.14, 106.06, 105.85, 55.00, 40.82, 28.31. | [M+H] ^+^  448.0934 |
| **2e** |  | ^1^H NMR (400 MHz, DMSO-*d*_6_) δ 9.55 (t, *J* = 6.1 Hz, 1H), 8.76 (d, *J* = 6.8 Hz, 1H), 7.88 (d, *J* = 7.6 Hz, 2H), 7.72 (d, *J* = 3.2 Hz, 1H), 7.63 (d, *J* = 3.2 Hz, 1H), 7.56 (t, *J* = 7.5 Hz, 1H), 7.48 (t, *J* = 7.5 Hz, 2H), 5.31 – 5.23 (m, 1H), 4.63 (d, *J* = 6.2 Hz, 2H), 1.85 – 1.77 (m, 1H), 1.72 – 1.59 (m, 6H), 1.50 – 1.44 (m, 1H), 1.18 – 1.08 (m, 2H), 1.01 – 0.84 (m, 3H).  ^13^C NMR (101 MHz, DMSO) δ 197.61, 168.22, 167.06, 161.75, 142.66, 133.93, 131.99, 128.74, 128.02, 120.75, 52.87, 40.83, 36.46, 34.49, 33.73, 31.92, 26.81, 26.48, 26.15, 25.94. | [M+H] ^+^  400.1691 |
| **2f** |  | ^1^H NMR (400 MHz, DMSO-*d*_6_) δ 9.59 (t, *J* = 6.2 Hz, 1H), 8.97 (d, *J* = 7.1 Hz, 1H), 7.81 – 7.75 (m, 2H), 7.71 (d, *J* = 3.3 Hz, 1H), 7.60 (d, *J* = 3.3 Hz, 1H), 7.57 – 7.52 (m, 1H), 7.47 (dd, *J* = 8.2, 6.7 Hz, 2H), 7.39 (t, *J* = 1.8 Hz, 1H), 7.35 – 7.30 (m, 1H), 7.29 – 7.24 (m, 2H), 5.32 – 5.26 (m, 1H), 4.62 (d, *J* = 6.3 Hz, 2H), 3.27 – 3.21 (m, 1H), 3.05 – 2.97 (m, 1H).  ^13^C NMR (101 MHz, DMSO) δ 196.11, 168.16, 167.11, 161.73, 142.65, 140.92, 133.74, 133.30, 132.09, 130.62, 129.41, 128.77, 128.24, 127.92, 126.99, 120.73, 56.66, 40.86, 34.57. | [M+H] ^+^  428.0833 |
| **2g** |  | ^1^H NMR (400 MHz, DMSO-*d*_6_) δ 9.57 (t, *J* = 6.3 Hz, 1H), 8.99 (d, *J* = 7.1 Hz, 1H), 7.81 – 7.75 (m, 2H), 7.72 – 7.69 (m, 1H), 7.61 – 7.52 (m, 4H), 7.47 (t, *J* = 8.0 Hz, 2H), 7.29 (dd, *J* = 8.3, 2.0 Hz, 1H), 5.30 – 5.22 (m, 1H), 4.61 (d, *J* = 6.2 Hz, 2H), 3.26 – 3.19 (m, 1H), 3.05 – 2.97 (m, 1H).  ^13^C NMR (101 MHz, DMSO) δ 200.68, 172.93, 171.86, 166.52, 147.39, 144.33, 138.41, 136.87, 136.40, 135.95, 135.63, 134.77, 134.39, 133.53, 132.68, 125.44, 61.17, 45.61, 38.87. | [M+H] +  462.0449 |
| **3a** |  | ^1^H NMR (400 MHz, DMSO-*d*_6_) δ 9.64 (t, *J* = 6.3 Hz, 1H), 8.75 (dd, *J* = 7.3, 2.4 Hz, 1H), 7.74 (d, *J* = 3.3 Hz, 1H), 7.64 (d, *J* = 3.0 Hz, 1H), 7.58 – 7.47 (m, 2H), 7.34 – 7.20 (m, 7H), 5.44 – 5.34 (m, 1H), 4.66 (d, *J* = 6.3 Hz, 2H), 3.29 – 3.18 (m, 1H), 3.01 – 2.89 (m, 1H).  ^13^C NMR (101 MHz, DMSO) δ 195.90, 168.08, 164.36, 161.45, 160.94, 158.46, 142.67, 137.89, 133.32, 133.23, 130.59, 130.56, 129.53, 128.80, 127.08, 124.91, 124.88, 123.51, 123.37, 120.81, 116.75, 116.53, 56.90, 40.90, 35.21. | [M+H] ^+^  412.1119 |
| **3b** |  | ^1^H NMR (400 MHz, DMSO-*d*_6_) δ 9.61 (t, *J* = 6.4 Hz, 1H), 9.03 (d, *J* = 7.2 Hz, 1H), 7.72 (d, *J* = 3.3 Hz, 1H), 7.67 – 7.51 (m, 4H), 7.44 – 7.37 (m, 1H), 7.34 – 7.26 (m, 4H), 7.23 – 7.17 (m, 1H), 5.40 – 5.29 (m, 1H), 4.64 (d, *J* = 6.3 Hz, 2H), 3.27 – 3.19 (m, 1H), 3.05 – 2.92 (m, 1H). | [M+H] ^+^  412.1121 |
| **3c** |  | ^1^H NMR (400 MHz, DMSO-*d*_6_) δ 9.60 (t, *J* = 6.2 Hz, 1H), 8.97 (d, *J* = 7.1 Hz, 1H), 7.90 – 7.83 (m, 2H), 7.71 (d, *J* = 3.3 Hz, 1H), 7.61 (d, *J* = 3.3 Hz, 1H), 7.36 – 7.26 (m, 6H), 7.24 – 7.18 (m, 1H), 5.37 – 5.29 (m, 1H), 4.70 – 4.60 (m, 2H), 3.27 – 3.20 (m, 1H), 3.04 – 2.92 (m, 1H).  ^13^C NMR (101 MHz, DMSO) δ 196.45, 168.14, 166.03, 165.77, 163.30, 161.69, 142.65, 138.30, 130.68, 130.59, 130.30, 130.27, 129.45, 128.80, 126.98, 120.74, 115.83, 115.62, 57.05, 40.87, 34.97. | [M+H] ^+^  412.1122 |
| **3d** |  | ^1^H NMR (400 MHz, DMSO-*d*_6_) δ 9.55 (t, *J* = 6.2 Hz, 1H), 8.60 (d, *J* = 7.1 Hz, 1H), 7.73 (d, *J* = 3.3 Hz, 1H), 7.64 (d, *J* = 3.3 Hz, 1H), 7.36 – 7.11 (m, 10H), 5.22 – 5.11 (m, 1H), 4.64 – 4.52 (m, 2H), 3.43 (s, 2H), 3.20 – 3.10 (m, 1H), 2.92 – 2.74 (m, 1H). | [M+H] ^+^  408.1372 |
| **3e** |  | ^1^H NMR (400 MHz, DMSO-*d*_6_) δ 9.54 (t, *J* = 6.3 Hz, 1H), 8.40 (d, *J* = 7.1 Hz, 1H), 7.73 (d, *J* = 3.2 Hz, 1H), 7.64 (d, *J* = 3.4 Hz, 1H), 7.32 – 7.08 (m, 10H), 5.24 – 5.11 (m, 1H), 4.62 (d, *J* = 6.2 Hz, 2H), 3.11 (dd, *J* = 14.0, 4.3 Hz, 1H), 2.83 – 2.76 (m, 1H), 2.72 (t, *J* = 8.1 Hz, 2H), 2.38 (t, *J* = 7.9 Hz, 2H). | [M+H] ^+^  422.1528 |
| **3f** |  | ^1^H NMR (400 MHz, DMSO-*d*_6_) δ 9.62 (t, *J* = 6.3 Hz, 1H), 8.49 (d, *J* = 7.5 Hz, 1H), 7.72 (d, *J* = 3.3 Hz, 1H), 7.61 (d, *J* = 3.3 Hz, 1H), 7.39 – 7.09 (m, 7H), 6.96 (t, *J* = 7.3 Hz, 1H), 6.88 (d, *J* = 8.1 Hz, 2H), 5.35 – 5.20 (m, 1H), 4.64 (d, *J* = 6.3 Hz, 2H), 4.48 (s, 2H), 3.23 – 3.13 (m, 1H), 3.02 – 2.87 (m, 1H).  ^13^C NMR (101 MHz, DMSO) δ 195.89, 168.45, 168.09, 161.41, 158.14, 142.68, 137.63, 129.92, 129.52, 128.82, 127.06, 121.67, 120.81, 115.13, 66.93, 55.89, 40.90, 35.38. | [M+H] ^+^  424.1321 |
| **3g** |  | ^1^H NMR (400 MHz, DMSO-*d*_6_) δ 9.57 (t, *J* = 6.0 Hz, 1H), 8.68 (t, *J* = 5.8 Hz, 1H), 8.40 (d, *J* = 7.1 Hz, 1H), 7.89 – 7.82 (m, 2H), 7.73 (d, *J* = 3.2 Hz, 1H), 7.63 (d, *J* = 3.2 Hz, 1H), 7.58 – 7.50 (m, 1H), 7.49 – 7.44 (m, 2H), 7.29 – 7.16 (m, 8H), 5.30 – 5.21 (m, 1H), 4.66 – 4.60 (m, 2H), 4.00 – 3.81 (m, 2H), 3.19 – 3.10 (m, 1H), 2.92 – 2.82 (m, 1H). | [M+H] ^+^  451.1432 |
| **3h** |  | ^1^H NMR (400 MHz, DMSO-*d*_6_) δ 9.58 (t, *J* = 5.9 Hz, 1H), 9.19 (s, 1H), 9.00 – 8.87 (m, 2H), 8.75 (s, 1H), 8.51 (d, *J* = 7.1 Hz, 1H), 7.72 (t, *J* = 2.3 Hz, 1H), 7.62 (dd, *J* = 3.2, 1.6 Hz, 1H), 7.32 – 7.16 (m, 5H), 5.30 – 5.20 (m, 1H), 4.62 (d, *J* = 6.2 Hz, 2H), 4.06 – 3.90 (m, 2H), 3.17 – 3.08 (m, 1H), 2.93 – 2.79 (m, 1H). | [M+H] ^+^  453.1338 |
| **3i** |  | ^1^H NMR (400 MHz, DMSO-*d*_6_) δ 9.58 (t, *J* = 5.9 Hz, 1H), 8.81 (d, *J* = 5.4 Hz, 1H), 8.65 (d, *J* = 4.3 Hz, 1H), 8.51 (d, *J* = 6.8 Hz, 1H), 8.09 – 7.95 (m, 2H), 7.72 (d, *J* = 3.0 Hz, 1H), 7.64 – 7.60 (m, 2H), 7.22 (q, *J* = 7.6, 5.7 Hz, 5H), 5.24 (d, *J* = 4.0 Hz, 1H), 4.62 (d, *J* = 6.1 Hz, 2H), 4.08 – 3.87 (m, 2H), 3.18 – 3.10 (m, 1H), 2.92 – 2.82 (m, 1H). | [M+H] ^+^  452.1384 |
| **3j** |  | ^1^H NMR (400 MHz, DMSO-*d*_6_) δ 9.58 (t, *J* = 5.6 Hz, 1H), 9.04 – 8.98 (m, 1H), 8.96 – 8.89 (m, 1H), 8.74 – 8.69 (m, 2H), 8.46 (d, *J* = 6.5 Hz, 1H), 8.19 (dd, *J* = 5.2, 2.7 Hz, 1H), 7.73 (t, *J* = 2.9 Hz, 1H), 7.63 (t, *J* = 2.9 Hz, 1H), 7.52 (dd, *J* = 7.8, 4.9 Hz, 2H), 7.30 – 7.14 (m, 5H), 5.31 – 5.21 (m, 1H), 4.68 – 4.61 (m, 2H), 3.96 – 3.89 (m, 2H), 3.18 – 3.09 (m, 1H), 2.92 – 2.79 (m, 1H). | [M+H] ^+^  452.1387 |
| **3k** |  | ^1^H NMR (400 MHz, DMSO-*d*_6_) δ 9.59 (t, *J* = 5.5 Hz, 1H), 8.43 (d, *J* = 7.1 Hz, 1H), 8.41 – 8.32 (m, 1H), 7.73 (d, *J* = 2.8 Hz, 1H), 7.69 (t, *J* = 7.5 Hz, 1H), 7.63 (d, *J* = 2.8 Hz, 1H), 7.59 – 7.52 (m, 1H), 7.33 – 7.19 (m, 7H), 5.31 – 5.22 (m, 1H), 4.66 – 4.60 (m, 2H), 4.00 – 3.82 (m, 2H), 3.20 – 3.10 (m, 1H), 2.92 – 2.81 (m, 1H). | [M+H] ^+^  469.1341 |
| **3l** |  | ^1^H NMR (400 MHz, DMSO-*d*_6_) δ 9.57 (t, *J* = 6.0 Hz, 1H), 8.72 (t, *J* = 5.7 Hz, 1H), 8.41 (d, *J* = 7.0 Hz, 1H), 8.00 – 7.89 (m, 2H), 7.73 (d, *J* = 3.2 Hz, 1H), 7.63 (d, *J* = 3.2 Hz, 1H), 7.31 (t, *J* = 8.7 Hz, 2H), 7.27 – 7.11 (m, 5H), 5.30 – 5.16 (m, 1H), 4.62 (d, *J* = 6.3 Hz, 2H), 3.97 – 3.81 (m, 2H), 3.18 – 3.07 (m, 1H), 2.91 – 2.80 (m, 1H).  ^13^C NMR (101 MHz, DMSO) δ 196.09, 169.63, 168.17, 165.82, 165.64, 163.17, 161.34, 142.67, 137.58, 130.92, 130.89, 130.48, 130.39, 129.54, 128.80, 127.06, 120.80, 115.78, 115.56, 55.94, 42.58, 40.89, 35.71. | [M+H] ^+^  469.1342 |
| **3m** |  | ^1^H NMR (400 MHz, DMSO-*d*_6_) δ 9.58 (t, *J* = 5.7 Hz, 1H), 8.81 (t, *J* = 5.3 Hz, 1H), 8.43 (d, *J* = 6.9 Hz, 1H), 7.75 – 7.68 (m, 2H), 7.65 (t, 2H), 7.54 (q, *J* = 7.1 Hz, 1H), 7.40 (t, *J* = 8.0 Hz, 1H), 7.29 – 7.18 (m, 5H), 5.31 – 5.21 (m, 1H), 4.63 (d, *J* = 5.5 Hz, 2H), 4.00 – 3.83 (m, 2H), 3.18 – 3.11 (m, 1H), 2.93 – 2.79 (m, 1H).  ^13^C NMR (101 MHz, DMSO) δ 196.09, 169.49, 168.16, 165.56, 165.53, 163.61, 161.34, 161.18, 142.67, 137.59, 136.82, 136.76, 130.99, 130.91, 129.54, 128.79, 127.05, 123.94, 120.80, 118.82, 118.61, 114.71, 114.48, 55.96, 42.59, 40.90, 35.69. | [M+H] ^+^  469.1339 |
| **3n** |  | ^1^H NMR (400 MHz, DMSO-*d*_6_) δ 9.59-9.56 (m, 1H), 8.27 (d, *J* = 7.0 Hz, 1H), 8.04-8.01(m, 1H), 7.73 (d, *J* = 3.4 Hz, 1H), 7.64 (d, *J* = 3.2 Hz, 1H), 7.29-7.20 (m, 5H), 5.26-5.18 (m, 1H), 4.65-4.55 (m, 2H), 3.75-3.63 (m, 2H), 3.15-3.10 (m, 1H), 2.86-2.80 (m, 1H), 2.33-2.30 (m, 1H), 2.02-2.01 (m, 2H), 1.84-1.70 (m, 4H), 1.66-1.56 (m, 2H).  ^13^C NMR (101 MHz, DMSO) δ 196.09, 174.50, 169.62, 168.14, 161.30, 142.68, 137.51, 129.53, 128.81, 127.07, 126.59, 124.19, 121.83, 120.81, 55.89, 41.89, 41.12, 40.90, 35.75, 32.89, 32.65, 32.42, 25.99, 25.90. | [M+H] ^+^  493.1717 |
| **3o** |  | ^1^H NMR (400 MHz, DMSO-*d*_6_) δ 9.63 – 9.51 (m, 1H), 8.33 – 8.24 (m, 1H), 8.20 – 8.09 (m, 1H), 7.76 – 7.68 (m, 1H), 7.67 – 7.58 (m, 1H), 7.34 – 7.12 (m, 5H), 5.28 – 5.17 (m, 1H), 4.67 – 4.55 (m, 2H), 3.77 – 3.59 (m, 2H), 3.18 – 3.08 (m, 1H), 2.85 (d, *J* = 11.6 Hz, 1H), 2.46 – 2.40 (m, 1H), 2.11 – 1.94 (m, 2H), 1.87 – 1.64 (m, 4H), 1.36 – 1.18 (m, 2H).  ^13^C NMR (101 MHz, DMSO) δ 196.09, 173.64, 169.53, 168.14, 161.35, 142.67, 137.54, 130.83, 129.71, 129.53, 128.80, 128.36, 127.06, 120.80, 55.91, 41.93, 41.12, 41.02, 40.89, 36.51, 36.27, 36.02, 35.70, 27.91, 22.11, 22.01. | [M+H] ^+^  493.1718 |
| **3p** |  | ^1^H NMR (400 MHz, DMSO-*d*_6_) δ 9.58 (t, *J* = 6.2 Hz, 1H), 8.33 (d, *J* = 7.1 Hz, 1H), 8.16-8.13 (m, 1H), 7.73 (d, *J* = 3.3 Hz, 1H), 7.64 (d, *J* = 3.2 Hz, 1H), 7.30-7.20 (m, 5H), 5.26-5.22 (m, 1H), 4.68-4.57 (m, 2H), 3.77-3.64 (m, 2H), 3.14-3.11 (m, 1H), 2.96-2.80 (m, 2H), 2.26-1.97 (m, 5H), 1.83-1.74 (m, 1H).  ^13^C NMR (101 MHz, DMSO) δ 196.09, 173.50, 169.48, 168.12, 161.32, 142.67, 137.54, 135.70, 133.22, 130.78, 129.51, 128.80, 127.07, 120.80, 55.91, 42.05, 41.29, 40.90, 38.85, 38.59, 38.34, 35.72, 35.56, 35.31, 35.07, 27.25, 27.22, 27.18. | [M+H] ^+^  479.1559 |
| **3q** |  | ^1^H NMR (400 MHz, DMSO-*d*_6_) δ 9.56 (t, J = 6.2 Hz, 1H), 8.31 (d, *J* = 7.2 Hz, 1H), 8.09-8.06 (m, 1H), 7.73 (d, *J* = 3.3 Hz, 1H), 7.64 (d, *J* = 3.1 Hz, 1H), 7.30-7.20 (m, 5H), 5.27-5.23 (m, 1H), 4.67-4.56 (m, 2H), 3.76-3.62 (m, 2H), 3.15-3.10 (m 1H), 2.86-2.80 (m, 1H), 2.65-2.57 (m, 2H), 2.28-2.19 (m, 4H).  ^13^C NMR (101 MHz, DMSO) δ 196.10, 171.24, 169.57, 168.15, 161.31, 142.67, 137.55, 129.52, 128.80, 127.07, 124.16, 121.34, 120.80, 118.62, 55.88, 41.82, 40.89, 40.77, 40.49, 35.70, 20.44, 20.37, 20.31, 20.24. | [M+H] ^+^  479.1557 |
| **3r** |  | ^1^H NMR (400 MHz, DMSO-*d*_6_) *δ* 9.54 (t, *J* = 6.2 Hz, 1H), 8.25 (d, *J* = 6.9 Hz, 1H), 7.94 (d, *J* = 7.7 Hz, 1H), 7.73 (d, *J* = 3.2 Hz, 1H), 7.64 (d, *J* = 3.3 Hz, 1H), 7.33 – 7.18 (m, 5H), 5.24 – 5.14 (m, 1H), 4.61 (d, *J* = 6.1 Hz, 2H), 4.37 – 4.24 (m, 1H), 3.18 – 3.08 (m, 1H), 2.93 – 2.81 (m, 1H), 2.35 – 2.24 (m, 1H), 2.09 – 1.95 (m, 2H), 1.86 – 1.66 (m, 4H), 1.62 – 1.47 (m, 2H), 1.15 (d, *J* = 7.0 Hz, 3H).  ^13^C NMR (151 MHz, DMSO-*D*_6_) δ 196.15, 174.02, 173.12, 168.20, 161.47, 142.74, 137.61, 129.62, 128.83, 127.10, 125.84, 124.25, 122.66, 120.85, 55.92, 48.08, 41.10, 40.92, 35.64, 32.92, 32.76, 32.60, 26.02, 25.96, 18.61. | [M+H] ^+^  507.1983 |
| **3s** |  | ^1^H NMR (400 MHz, DMSO-*d*_6_) δ 9.54-9.52 (m, 1H), 8.28-8.23 (m, 1H), 8.05 (t, *J* = 6.1 Hz, 1H), 7.73 (d, *J* = 3.3 Hz, 1H), 7.64 (d, *J* = 3.6 Hz, 1H), 7.29-7.21 (m, 5H), 5.23-5.14 (m, 1H), 4.64-4.60 (m, 2H), 4.31-4.26 (m, 1H), 3.14-3.07 (s, 1H), 2.89-2.83 (m, 1H), 2.40-2.38 (m, 1H), 2.05-1.98 (m, 2H), 1.86-1.62 (m, 4H), 1.44-1.30 (m, 2H), 1.04-1.02 (m, 3H).  ^13^C NMR (101 MHz, DMSO) δ 196.07, 196.05, 172.99, 172.96, 168.12, 168.11, 161.47, 161.38, 142.65, 137.60, 137.53, 129.55, 128.75, 128.73, 128.50, 127.16, 127.01, 120.78, 55.90, 55.84, 48.12, 48.07, 40.99, 40.97, 40.95, 40.87, 40.85, 36.26, 36.13, 35.54, 35.52, 33.44, 33.19, 32.96, 28.00, 27.90, 22.09, 22.00, 18.49, 18.43. | [M+H] ^+^  507.1875 |
| **3t** |  | ^1^H NMR (400 MHz, DMSO-*d*_6_) δ 9.57 (dt, *J* = 26.9, 5.8 Hz, 1H), 8.37 – 8.28 (m, 1H), 8.10 – 8.01 (m, 1H), 7.73 (dt, *J* = 4.0, 1.9 Hz, 1H), 7.64 (dt, *J* = 3.8, 2.0 Hz, 1H), 7.31 – 7.11 (m, 5H), 5.29 – 5.12 (m, 1H), 4.68 – 4.57 (m, 2H), 4.38 – 4.26 (m, 1H), 3.19 – 3.06 (m, 1H), 2.99 – 2.76 (m, 2H), 2.28 – 1.85 (m, 6H), 1.82 – 1.68 (m, 1H), 1.09 (dd, *J* = 49.8, 7.0 Hz, 3H).  ^13^C NMR (101 MHz, DMSO) δ 196.21, 196.08, 172.96, 172.95, 172.82, 172.79, 168.13, 168.06, 161.37, 161.33, 142.66, 137.61, 137.58, 135.77, 135.72, 133.32, 133.29, 130.85, 130.80, 129.57, 129.54, 128.75, 128.70, 127.02, 120.81, 120.78, 55.91, 55.84, 48.29, 48.20, 41.19, 41.13, 40.88, 40.86, 40.85, 38.86, 38.60, 38.49, 38.35, 35.84, 35.52, 35.32, 35.07, 27.23, 27.19, 18.80, 18.79, 18.62. | [M+H] ^+^  493.1728 |
| **3u** |  | ^1^H NMR (400 MHz, DMSO-*d*_6_) δ 9.54 (t, *J* = 6.3 Hz, 1H), 8.30 (d, *J* = 7.0 Hz, 1H), 8.01 (d, *J* = 7.5 Hz, 1H), 7.73 (d, *J* = 3.3 Hz, 1H), 7.64 (d, *J* = 3.2 Hz, 1H), 7.30 – 7.18 (m, 5H), 5.22 – 5.15 (m, 1H), 4.63 – 4.57 (m, 2H), 4.36 – 4.26 (m, 1H), 3.20 – 3.09 (m, 1H), 2.90 – 2.81 (m, 1H), 2.68 – 2.54 (m, 2H), 2.40 – 2.18 (m, 5H), 1.14 (d, *J* = 7.0 Hz, 3H).  ^13^C NMR (101 MHz, DMSO) δ 196.21, 172.87, 170.56, 168.10, 161.36, 142.68, 137.63, 129.59, 128.72, 127.03, 124.17, 121.45, 120.82, 118.63, 55.80, 48.03, 40.88, 40.62, 40.41, 40.20, 39.99, 39.78, 39.57, 39.36, 35.82, 20.35, 20.29, 18.86. | [M+H] ^+^  493.1716 |
| **3v** |  | ^1^H NMR (400 MHz, DMSO-*d*_6_) δ 9.56 (t, *J* = 6.3 Hz, 1H), 8.33 (d, *J* = 7.0 Hz, 1H), 7.99 (d, *J* = 8.2 Hz, 1H), 7.73 (d, *J* = 3.3 Hz, 1H), 7.64 (d, *J* = 3.3 Hz, 1H), 7.31 – 7.17 (m, 5H), 5.25 – 5.14 (m, 1H), 4.61 (d, *J* = 6.2 Hz, 2H), 4.55 – 4.48 (m, 1H), 3.48 – 3.36 (m, 2H), 3.22 (s, 3H), 3.17 – 3.09 (m, 1H), 2.93 – 2.85 (m, 1H), 2.40 – 2.31 (m, 1H), 2.09 – 1.97 (m, 2H), 1.84 – 1.69 (m, 4H), 1.64 – 1.51 (m, 2H).  ^13^C NMR (151 MHz, DMSO-*D*_6_) δ 195.88, 174.42, 170.34, 168.21, 161.41, 142.74, 137.54, 129.64, 128.83, 127.10, 125.86, 124.27, 122.68, 120.86, 72.40, 58.69, 55.95, 52.57, 41.07, 40.94, 35.63, 32.91, 32.75, 32.59, 26.06, 26.00. | [M+H] ^+^  537.1969 |
| **3w** |  | ^1^H NMR (400 MHz, DMSO-*d*_6_) δ 9.60 – 9.48 (m, 1H), 8.38 – 8.28 (m, 1H), 8.12 (d, *J* = 8.1 Hz, 1H), 7.73 (d, *J* = 3.3 Hz, 1H), 7.68 – 7.62 (m, 1H), 7.30 – 7.24 (m, 2H), 7.24 – 7.18 (m, 3H), 5.26 – 5.13 (m, 1H), 4.67 – 4.56 (m, 2H), 4.55 – 4.45 (m, 1H), 3.48 – 3.37 (m, 2H), 3.22 (d, *J* = 3.1 Hz, 3H), 3.18 – 3.09 (m, 1H), 2.94 – 2.85 (m, 1H), 2.06 – 1.90 (m, 2H), 1.87 – 1.63 (m, 4H), 1.47 – 1.35 (m, 1H), 1.33 – 1.21 (m, 1H).  ^13^C NMR (101 MHz, DMSO) δ 195.81, 195.78, 173.49, 173.43, 170.22, 170.19, 168.17, 161.42, 161.32, 142.66, 137.58, 137.51, 129.59, 128.73, 127.14, 127.00, 124.77, 122.37, 120.78, 72.30, 58.62, 55.97, 52.73, 40.97, 40.87, 36.45, 36.40, 36.22, 36.15, 35.98, 35.92, 35.50, 35.45, 33.45, 33.22, 32.99, 28.04, 22.11, 22.01. | [M+H] ^+^  537.1969 |
| **3x** |  | ^1^H NMR (400 MHz, DMSO-*d*_6_) δ 9.55 (t, *J* = 6.4 Hz, 1H), 8.38 (d, *J* = 7.0 Hz, 1H), 8.07 (d, *J* = 8.2 Hz, 1H), 7.73 (d, *J* = 3.2 Hz, 1H), 7.64 (d, *J* = 3.2 Hz, 1H), 7.26 (d, *J* = 6.9 Hz, 2H), 7.21 (d, *J* = 7.5 Hz, 3H), 5.20 (q, *J* = 6.8, 5.9 Hz, 1H), 4.61 (d, *J* = 6.2 Hz, 2H), 4.53 (q, *J* = 6.7 Hz, 1H), 3.41 (d, *J* = 6.7 Hz, 2H), 3.22 (s, 3H), 3.18 – 3.09 (m, 1H), 2.92 – 2.84 (m, 1H), 2.67 – 2.55 (m, 2H), 2.41 – 2.20 (m, 5H).  ^13^C NMR (101 MHz, DMSO) δ 195.82, 171.00, 170.24, 168.16, 161.33, 142.69, 137.52, 129.56, 128.75, 127.05, 124.16, 121.34, 120.81, 118.62, 72.40, 58.59, 55.91, 52.38, 40.88, 40.61, 40.40, 40.19, 39.98, 39.77, 39.56, 39.35, 35.66, 35.49, 20.50, 20.43. | [M+H] ^+^  523.1873  [M+K] ^+^  561.1408 |
| **3y** |  | ^1^H NMR (400 MHz, DMSO-*d*_6_) δ 9.56-9.51 (m, 1H), 8.39 (t, *J* = 7.3 Hz, 1H), 8.11 (d, *J* = 8.4 Hz, 1H), 7.73 (d, *J* = 3.1 Hz, 1H), 7.64 (d, *J* = 3.0 Hz, 1H), 7.27-7.20 (m, 5H), 5.25-5.14 (m, 1H), 4.62-4.51 (m, 3H), 3.45-3.39 (m, 2H), 3.22 (s, 3H), 3.14-3.10 (m, 1H), 3.01- 2.84 (m, 2H), 2.26-2.11 (m, 3H), 2.02-1.90 (m, 2H), 1.78-171 (m, 1H).  ^13^C NMR (101 MHz, DMSO) δ 195.80, 173.33, 170.14, 168.12, 161.35, 142.66, 137.51, 129.55, 128.75, 127.03, 120.78, 72.36, 58.66, 55.94, 52.71, 52.70, 41.07, 40.87, 38.83, 35.54, 35.31, 35.07, 28.01. | [M+H] ^+^  523.1826  [M+Na] ^+^  555.2079 |

**^1^H and ^13^C NMR spectra of products**


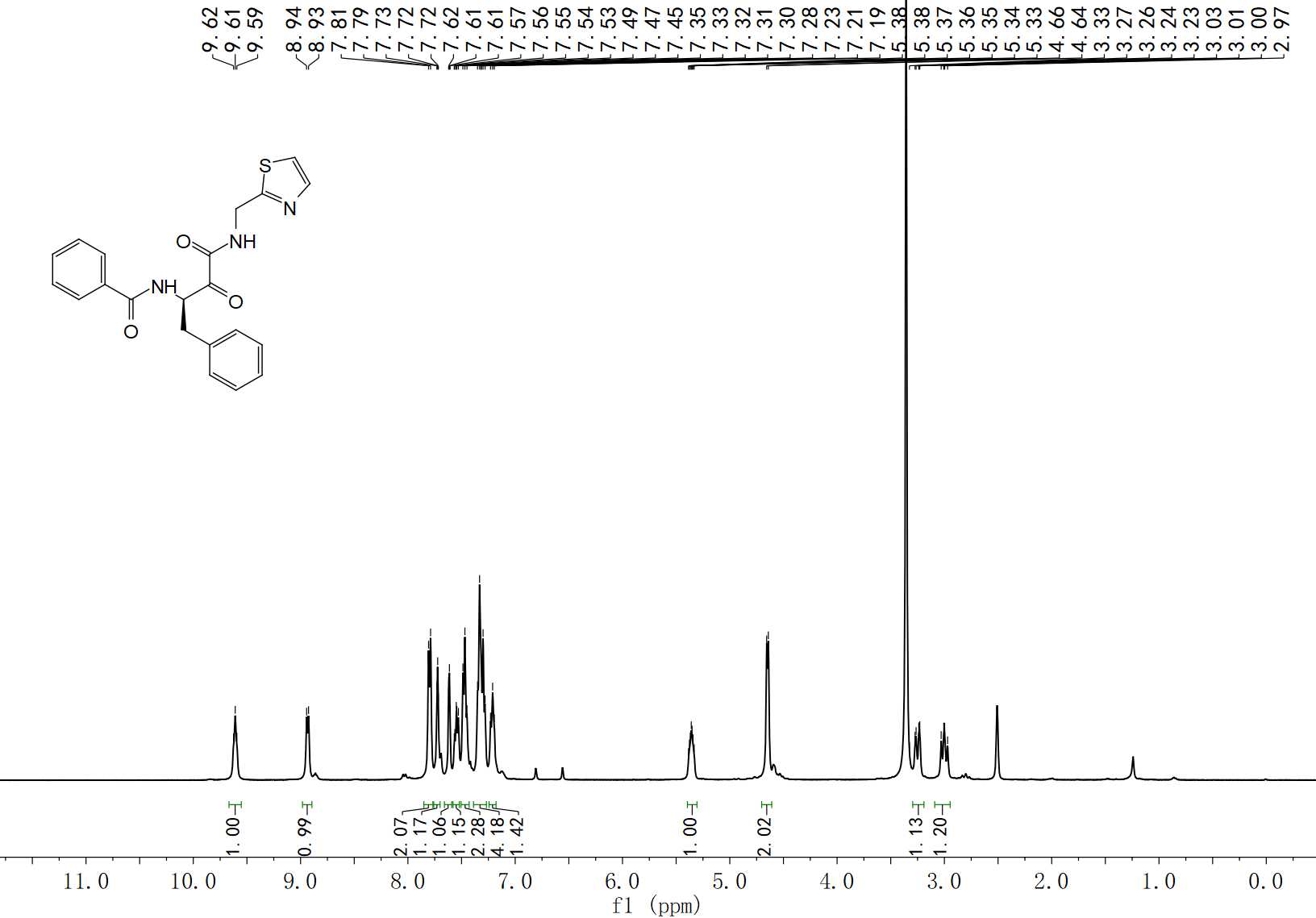


**^1^H NMR of 1a**


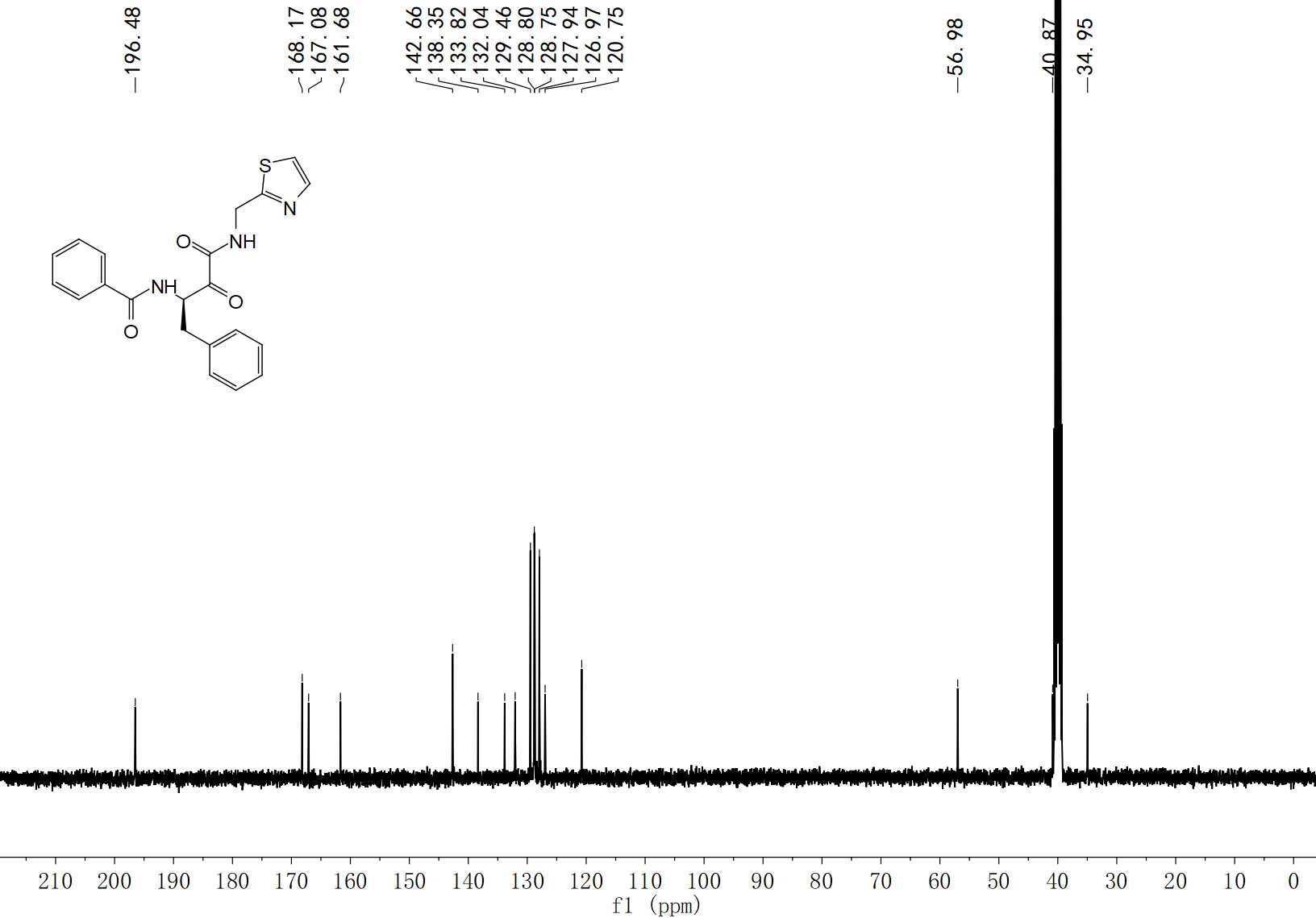


**^13^C NMR of 1a**


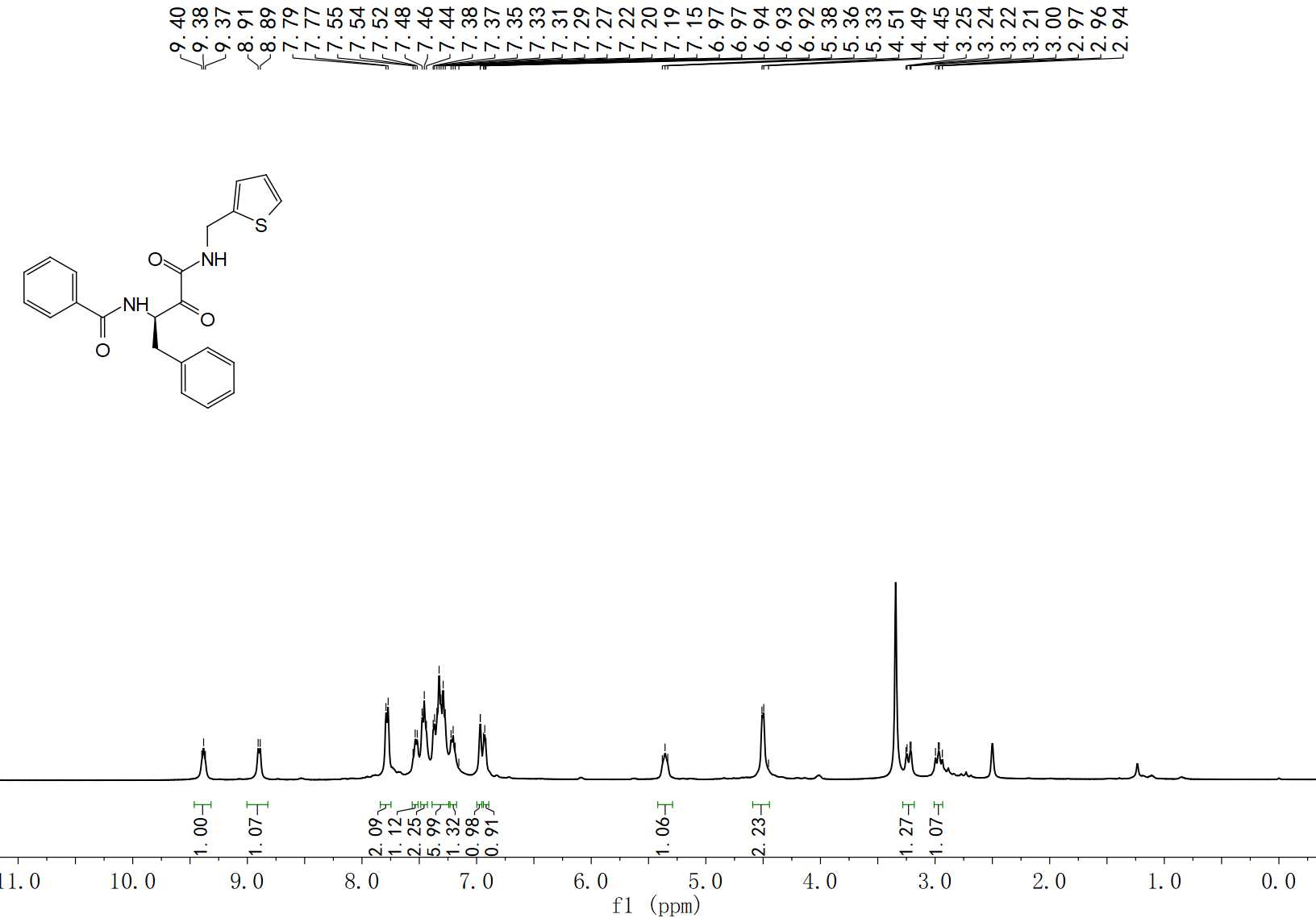


**^1^H NMR of 1b**


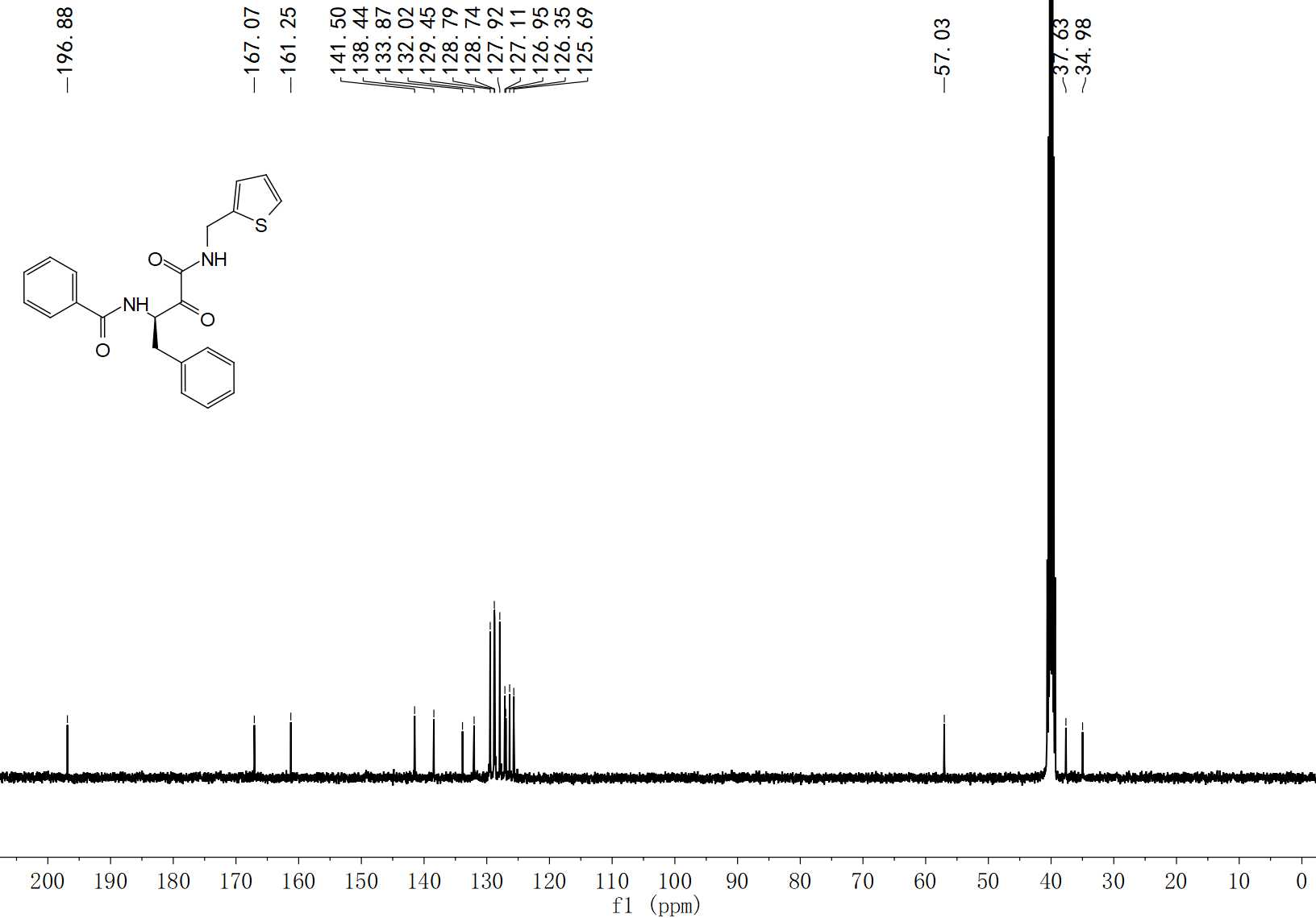


**^13^C NMR of 1b**


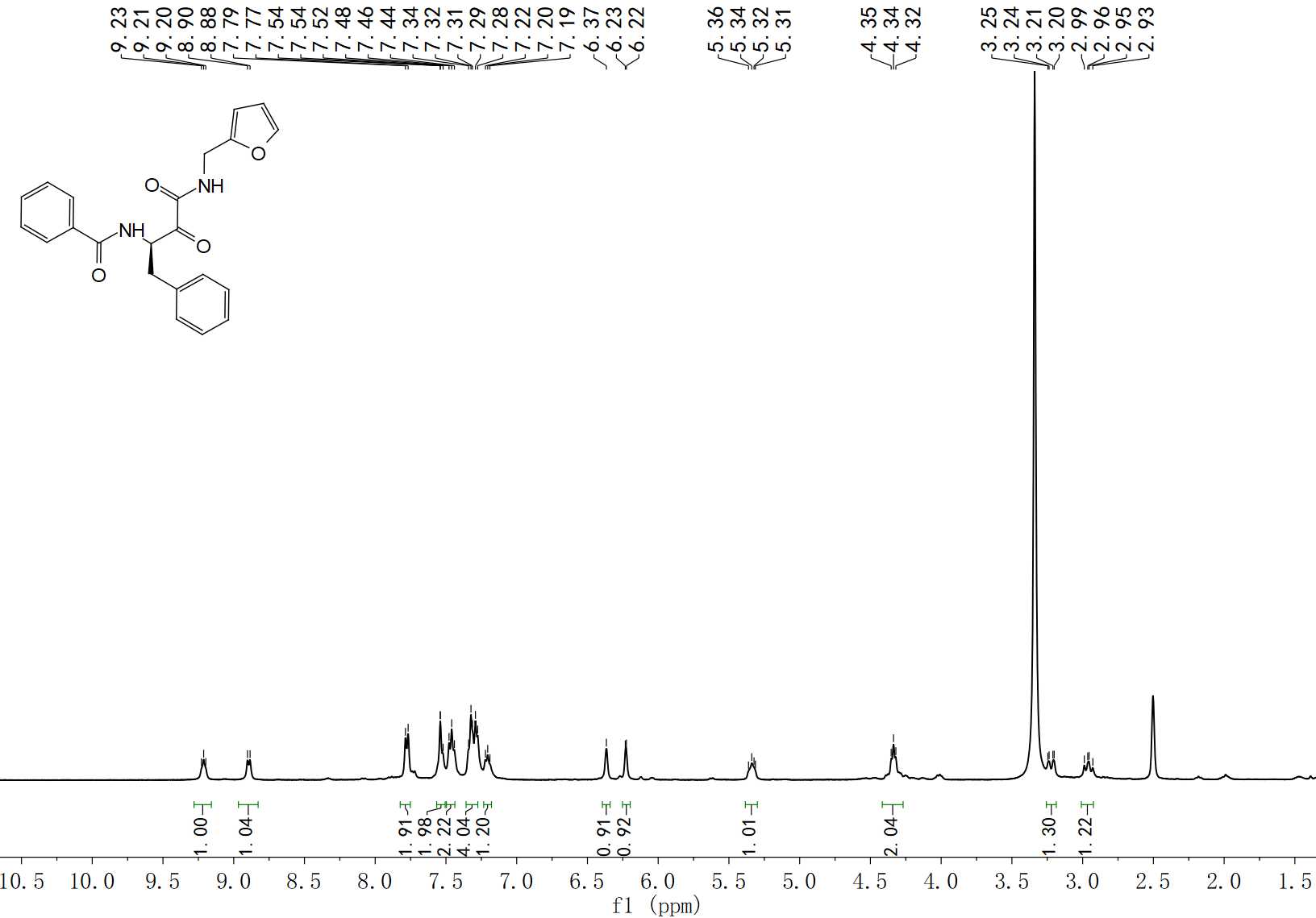


**^1^H NMR of 1c**

**
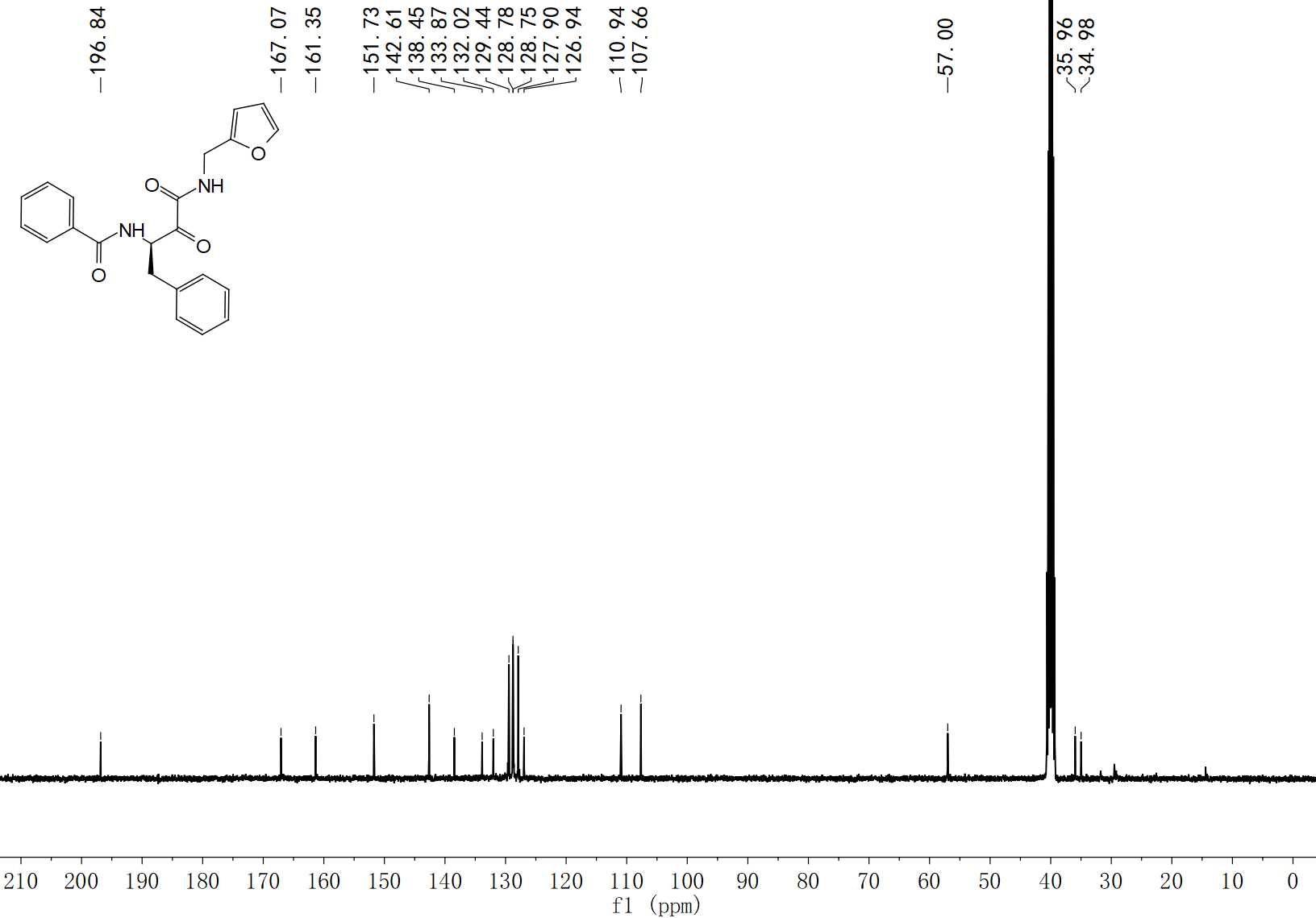
**

**^13^C NMR of 1c**


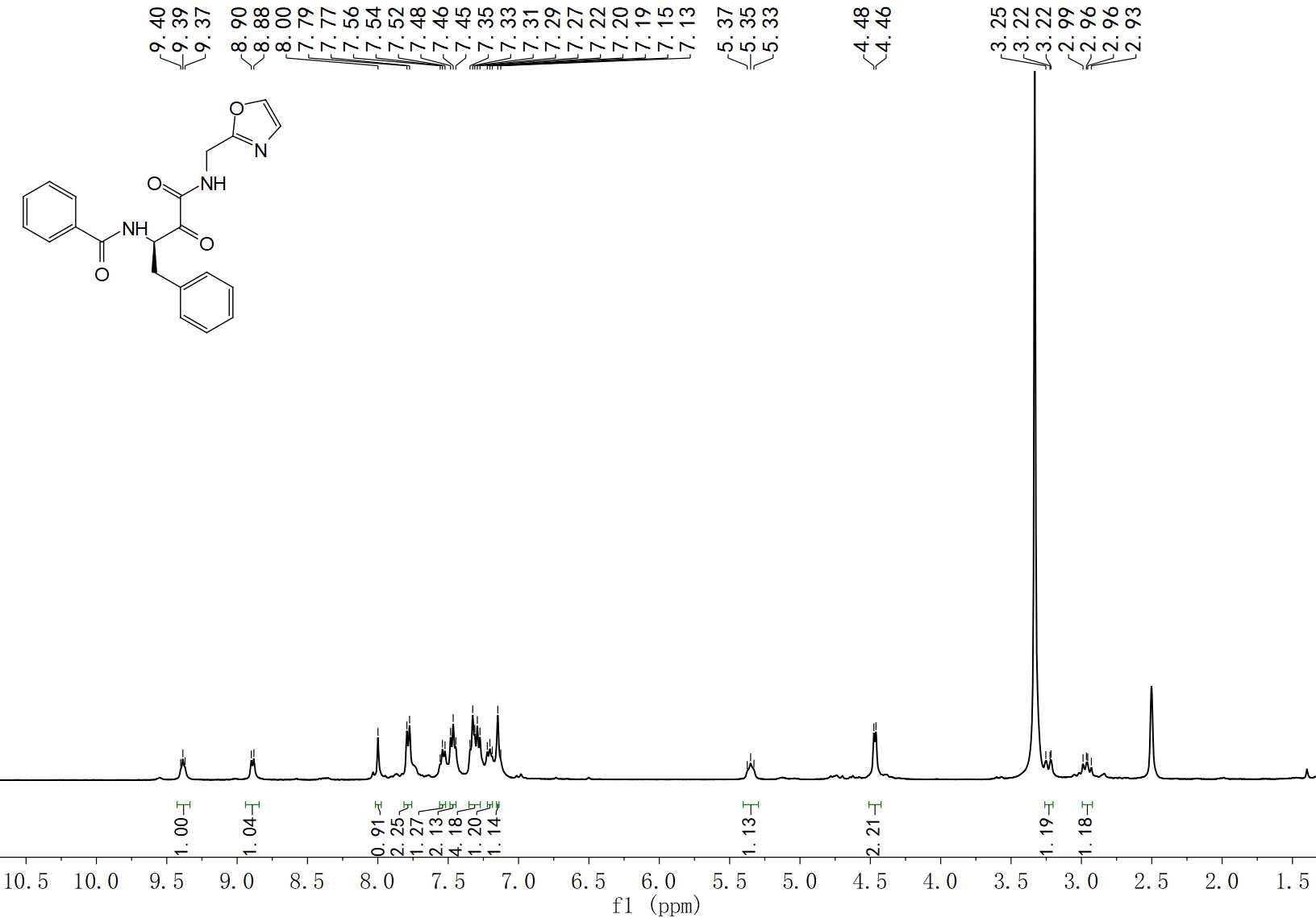


**^1^H NMR of 1d**


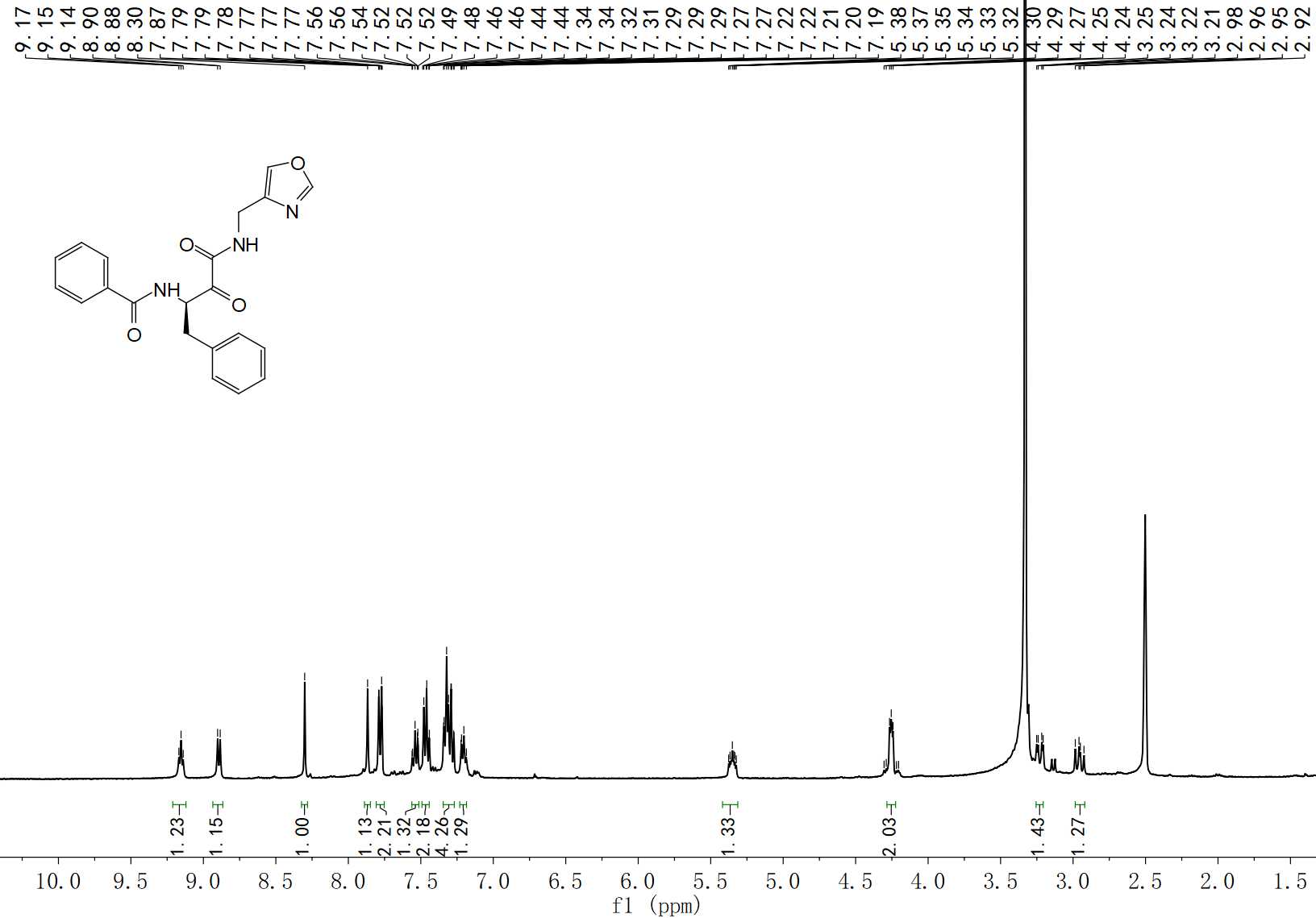


**^1^H NMR of 1e**


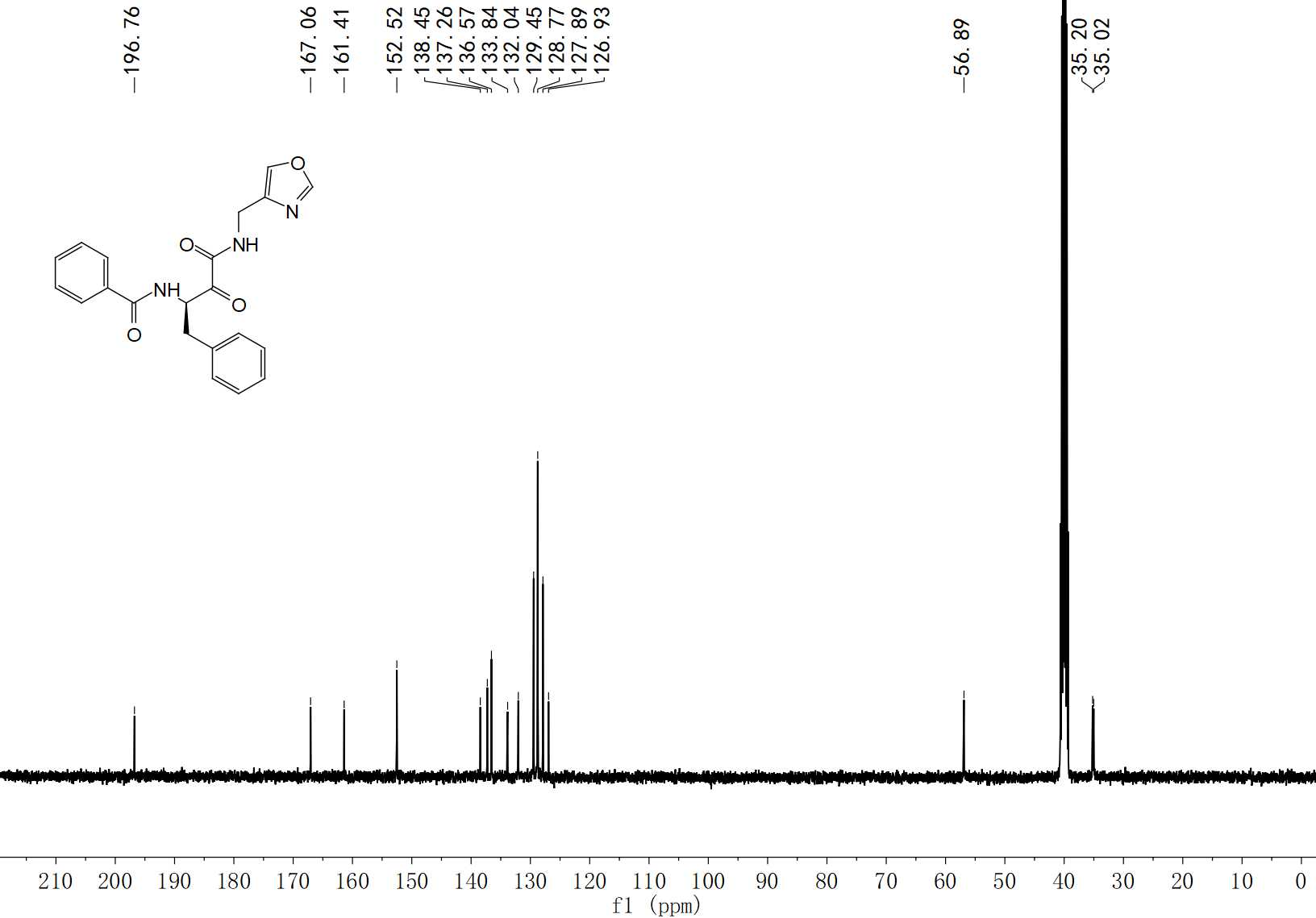


**^13^C NMR of 1e**


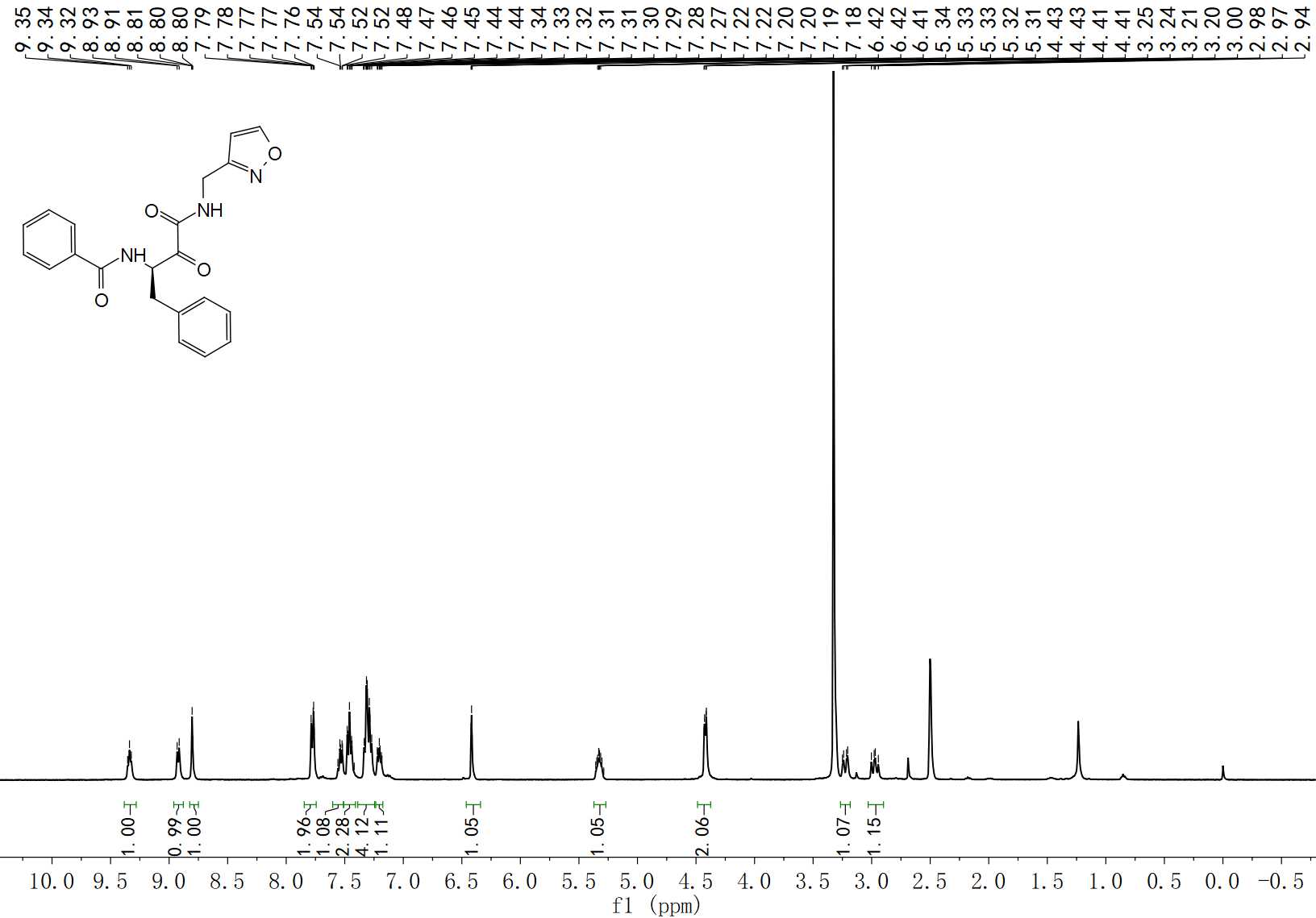


**^1^H NMR of 1f**


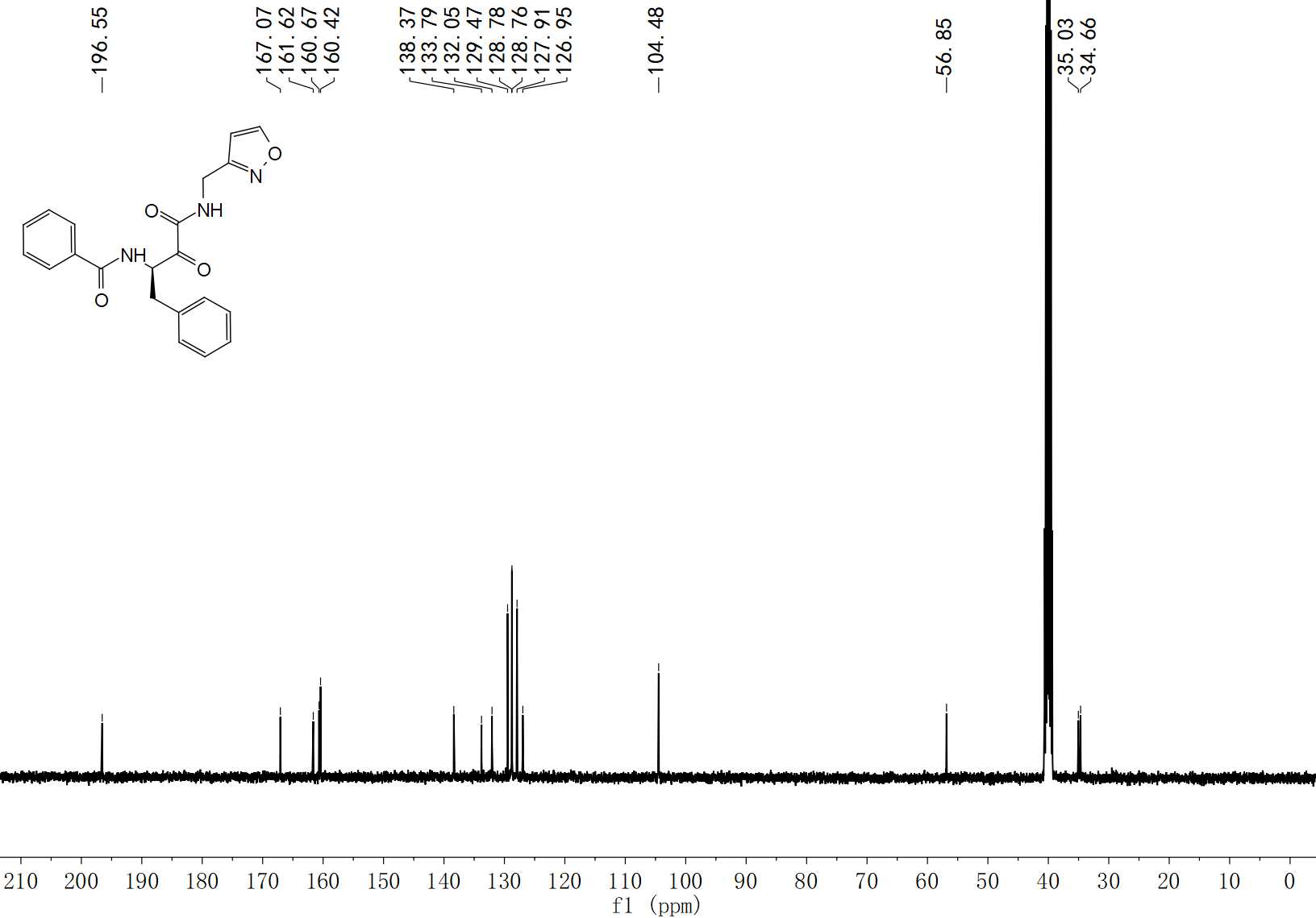


**^13^C NMR of 1f**


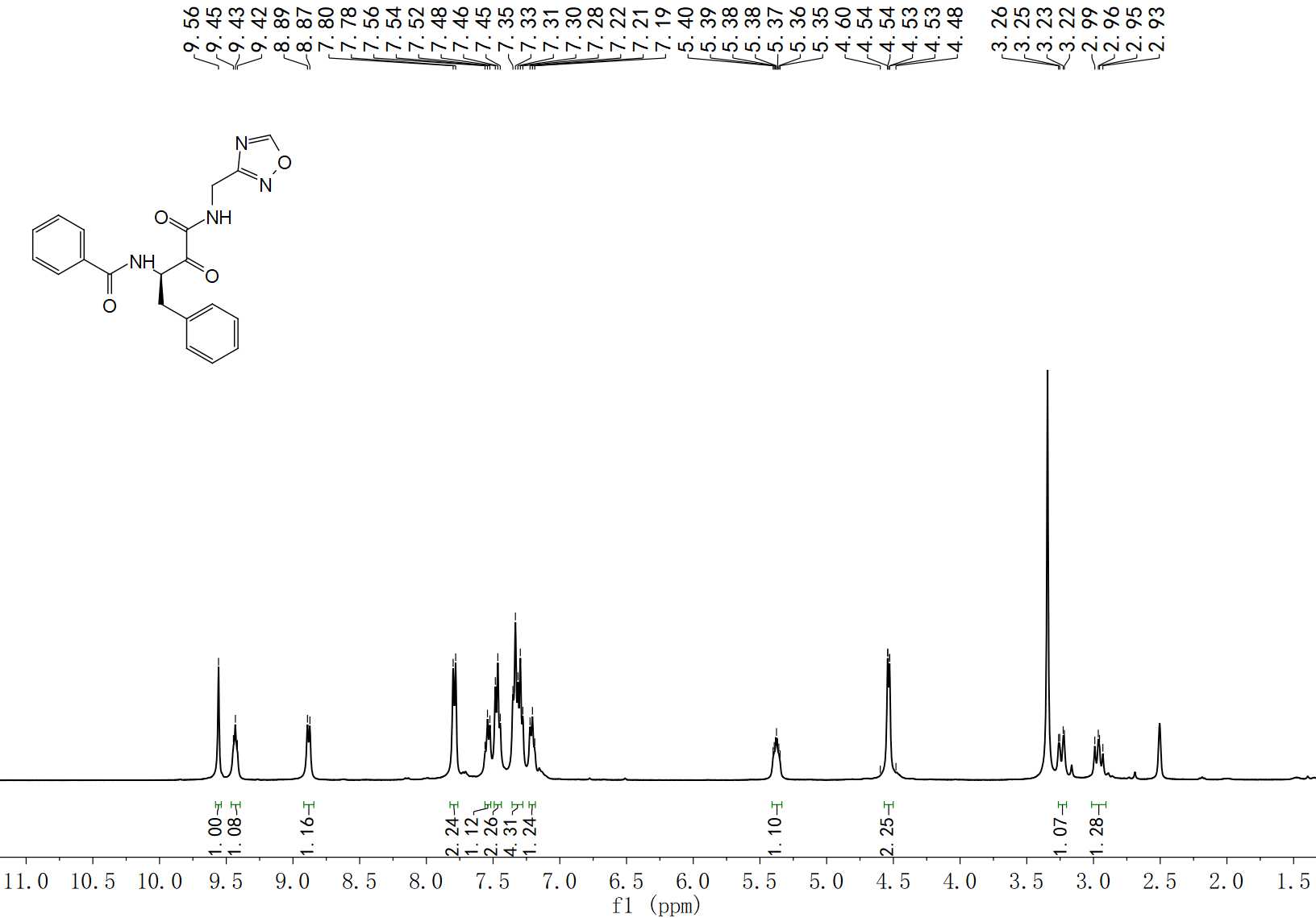


**^1^H NMR of 1g**


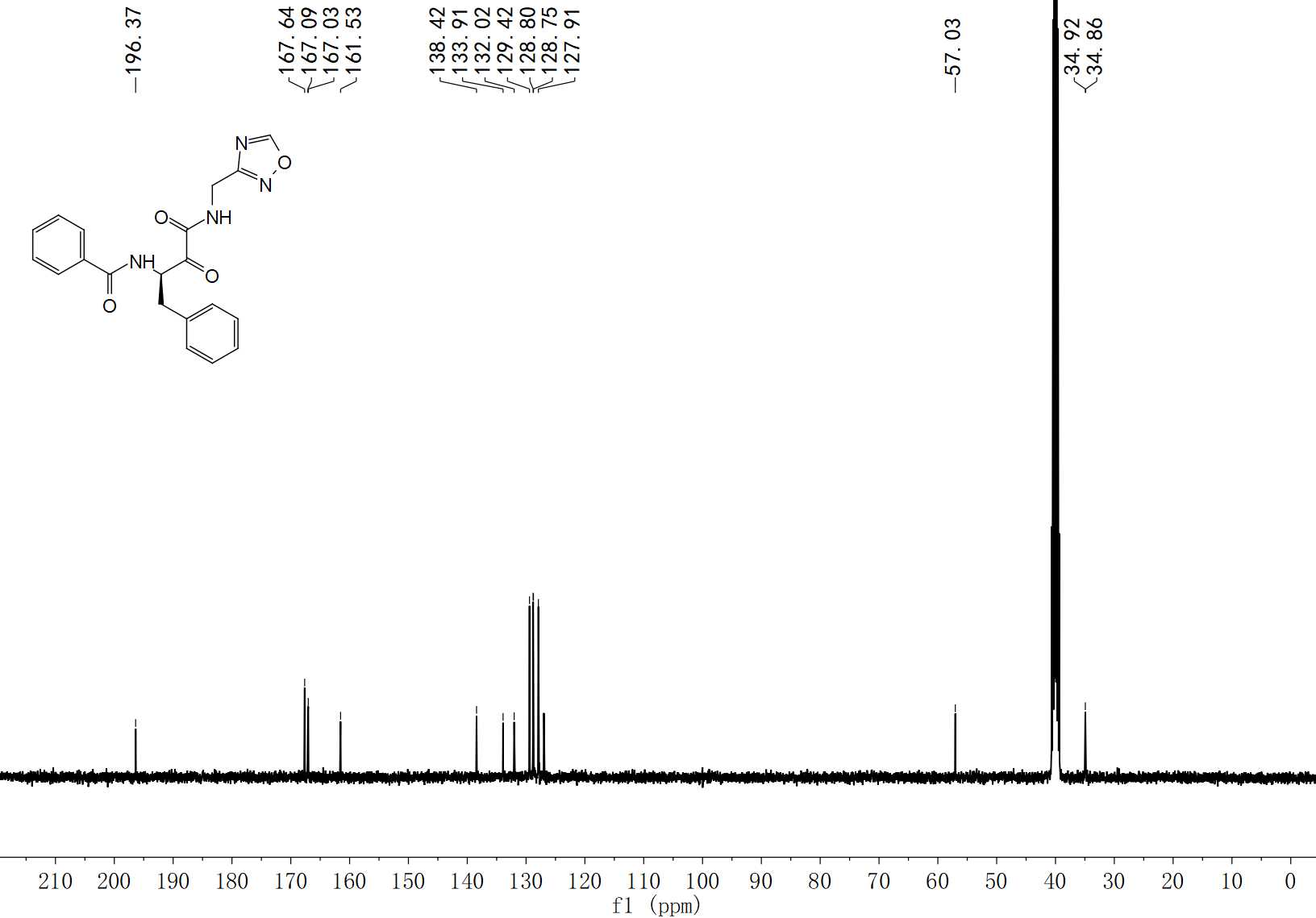


**^13^C NMR of 1g**


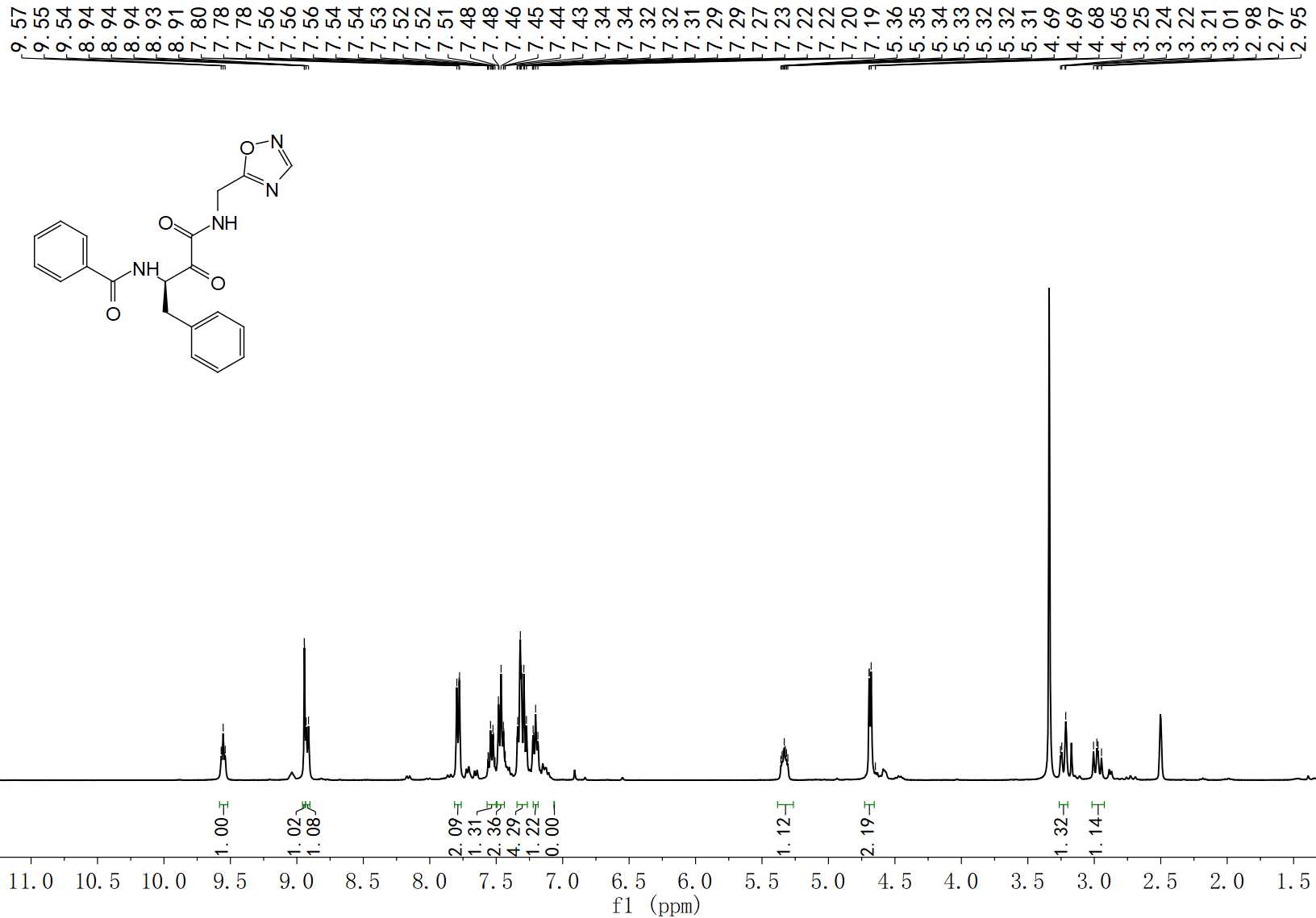


**^1^H NMR of 1h**


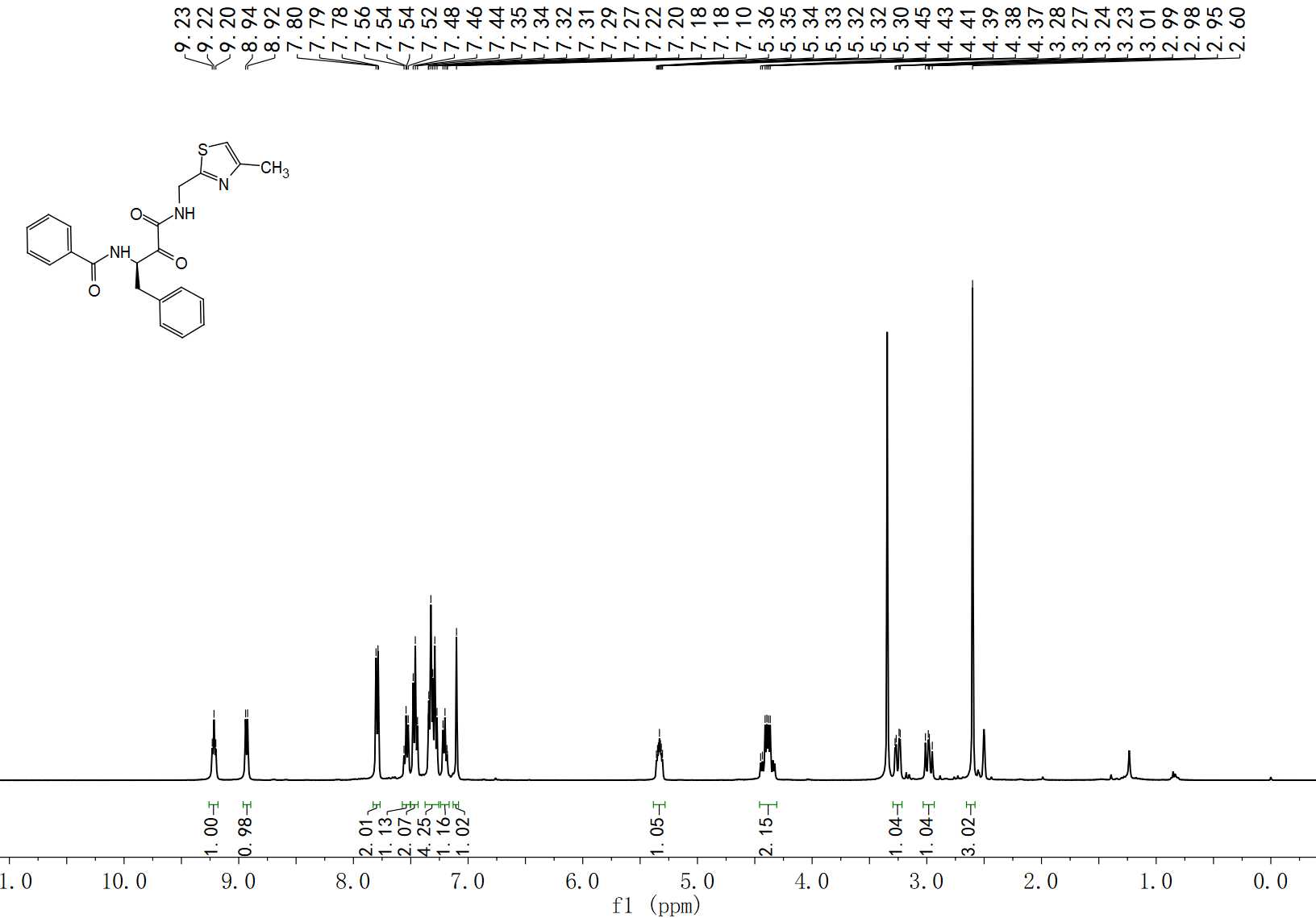


**^1^H NMR of 1i**


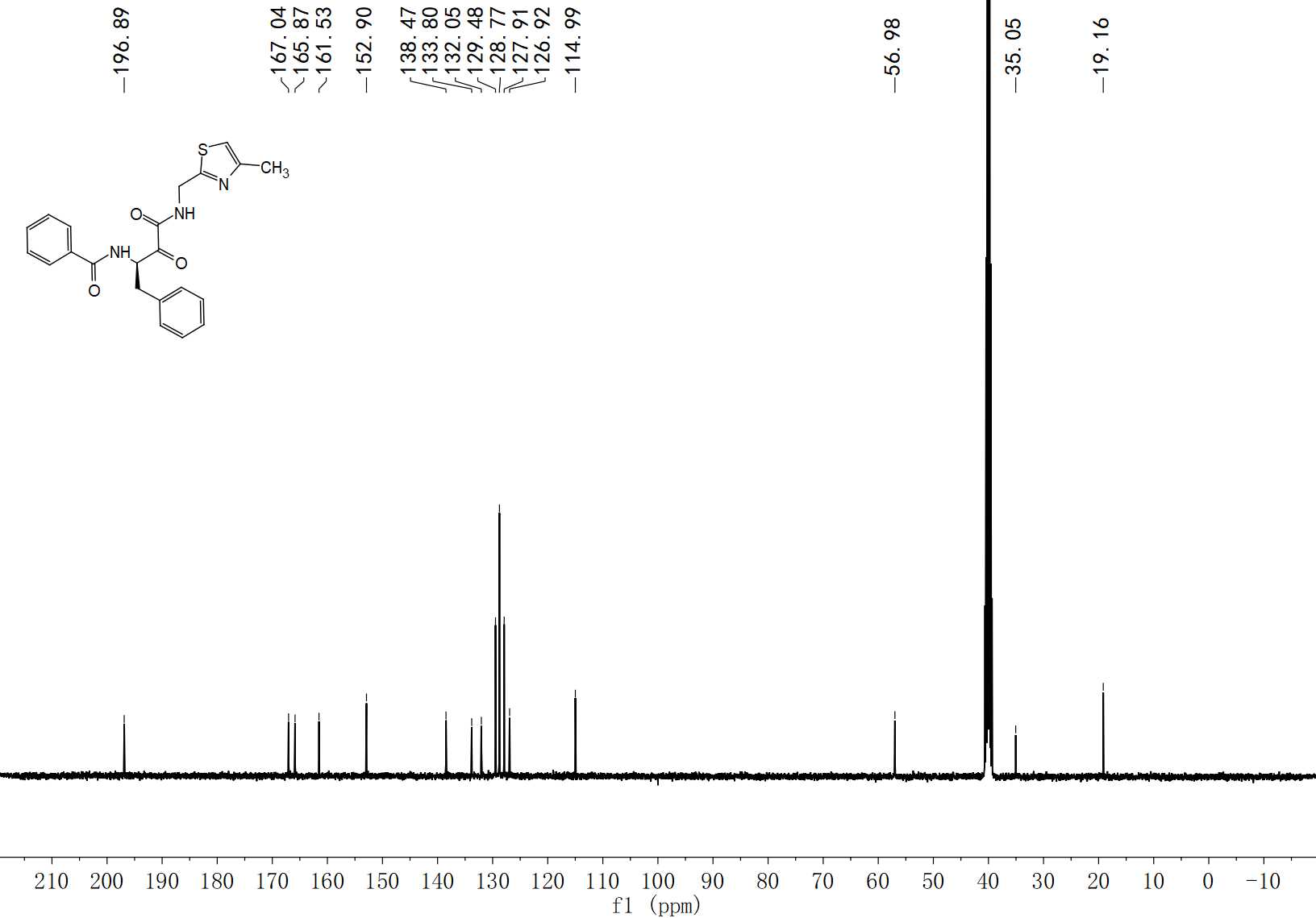


**^13^C NMR of 1i**


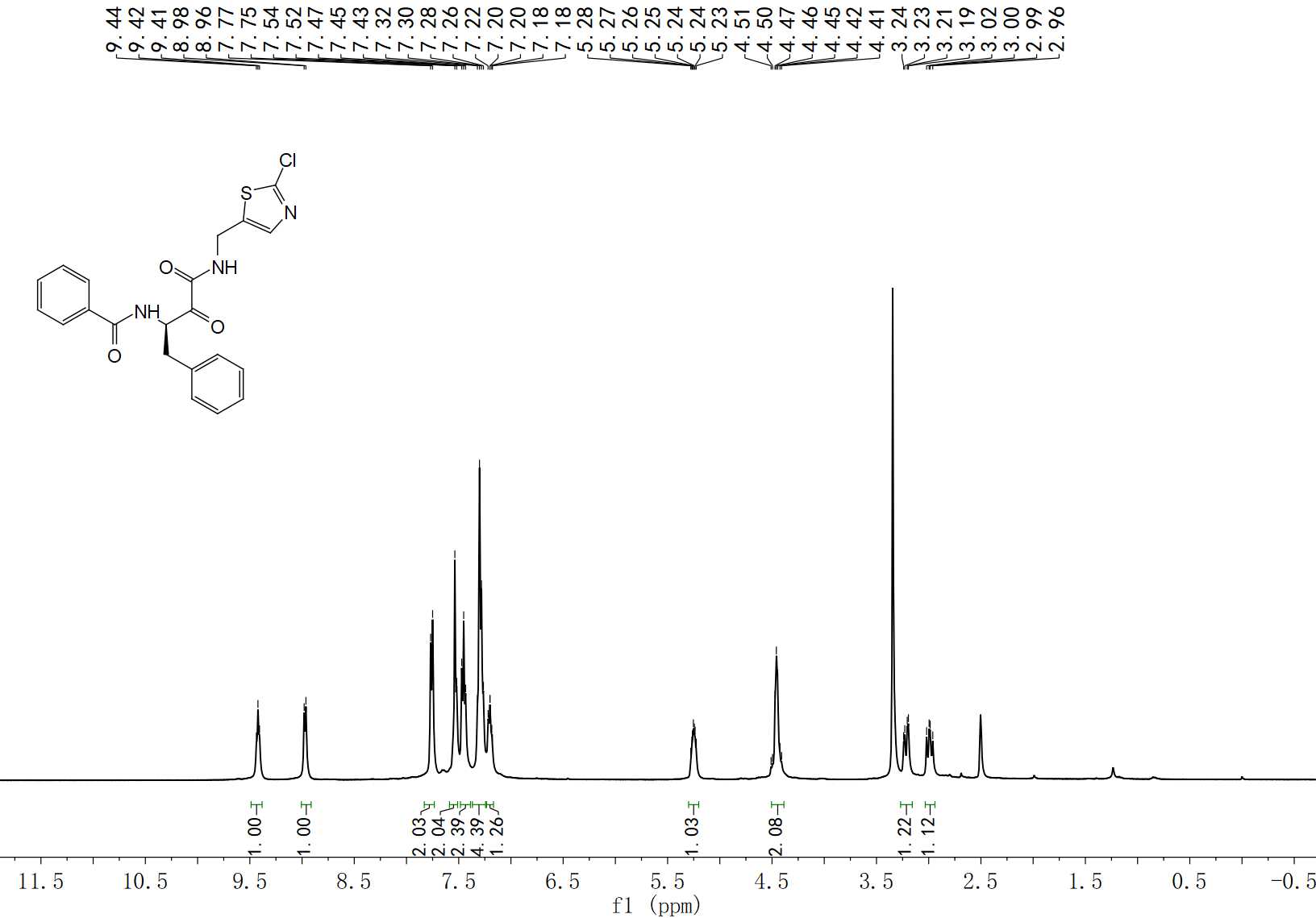


**^1^H NMR of 1j**


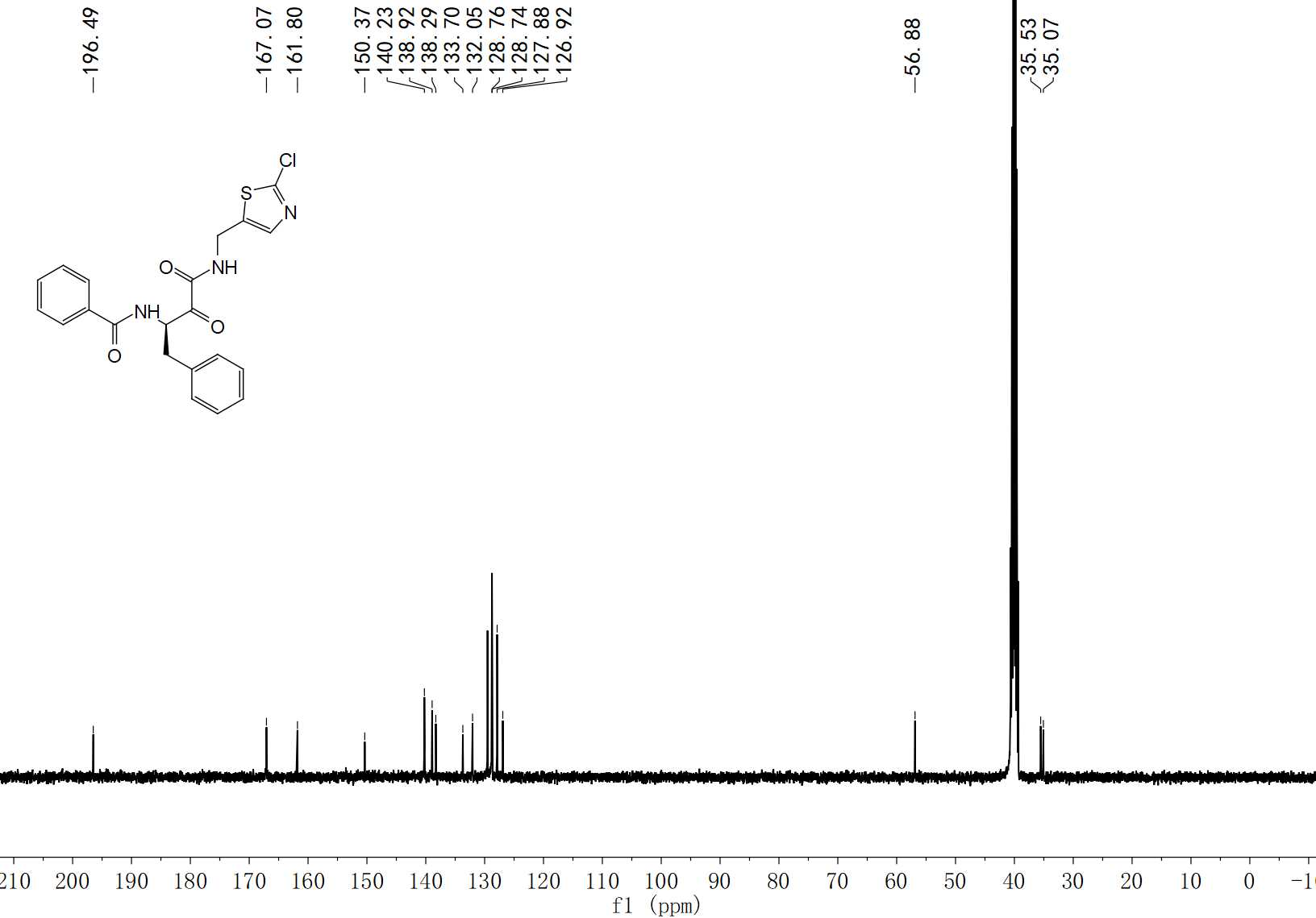


**^13^C NMR of 1j**


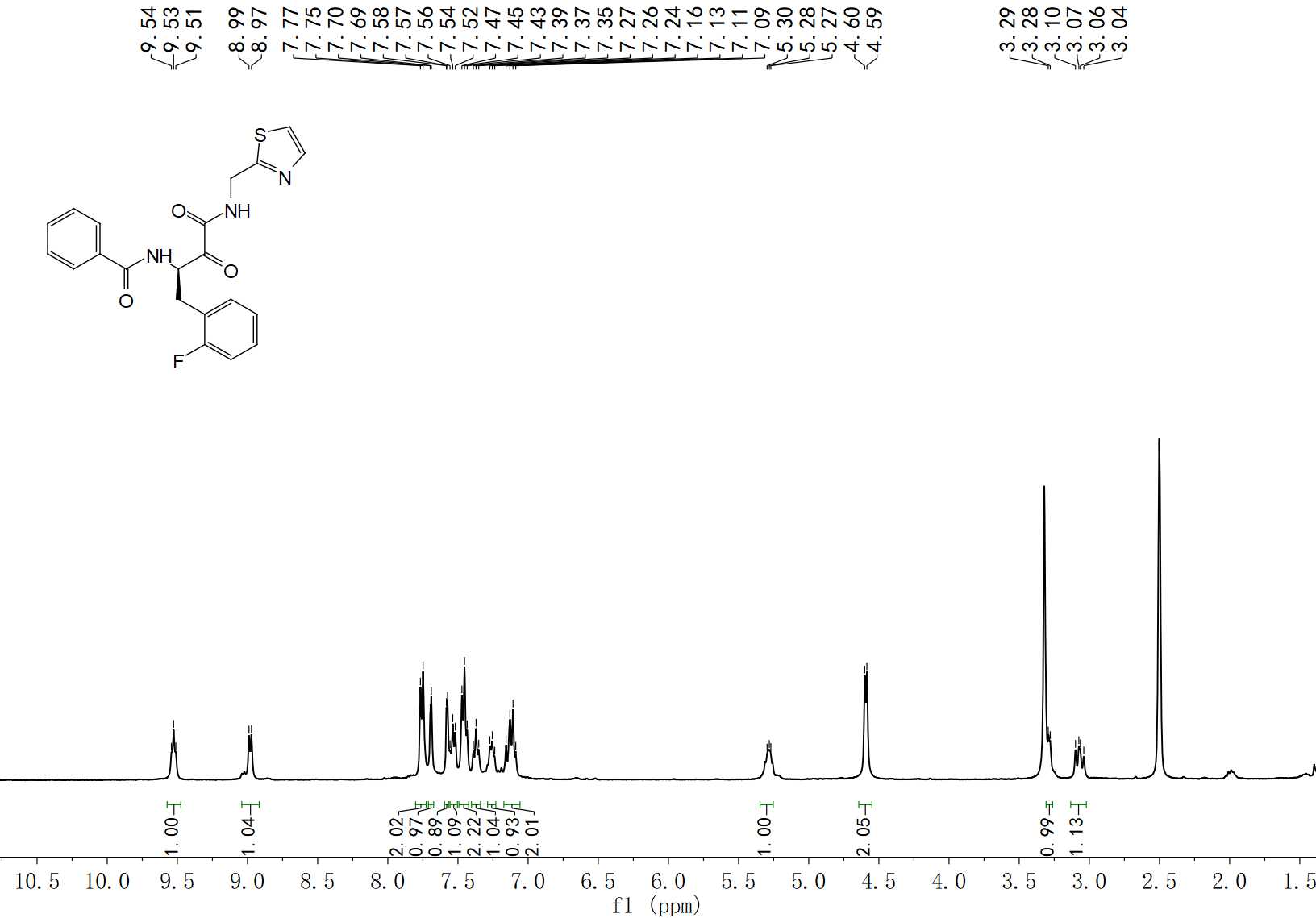


**^1^H NMR of 2a**


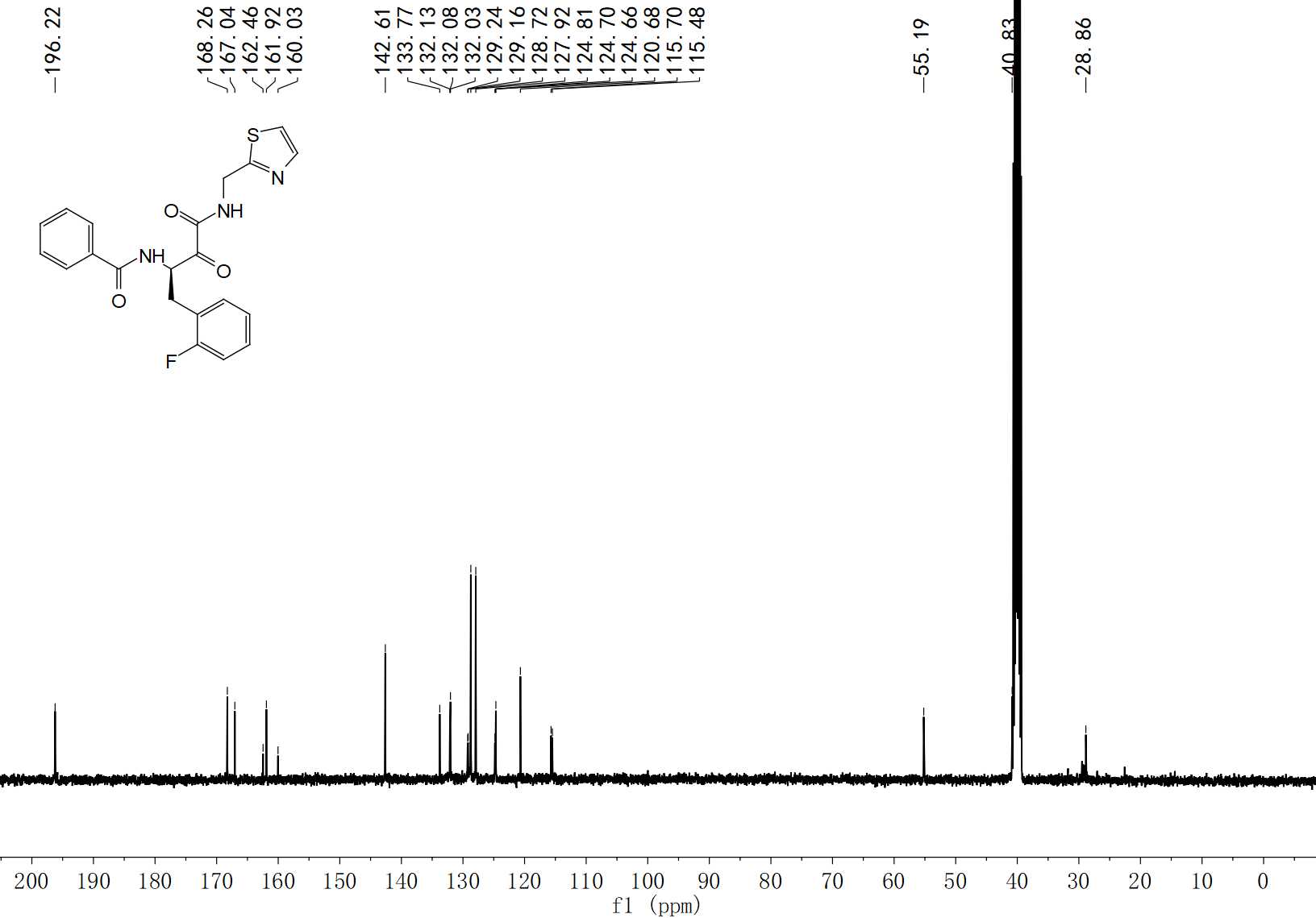


**^13^C NMR of 2a**


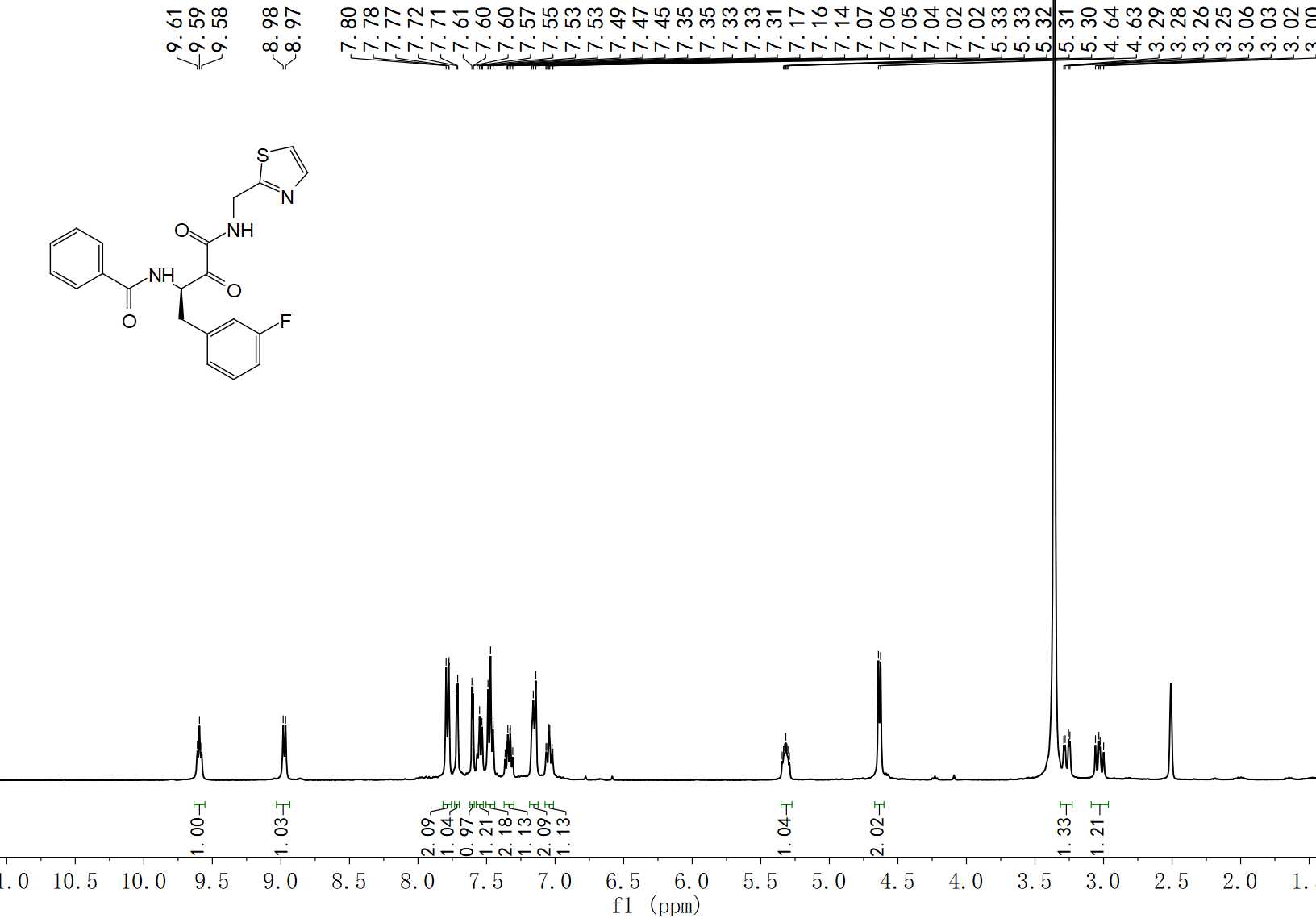


**^1^H NMR of 2b**


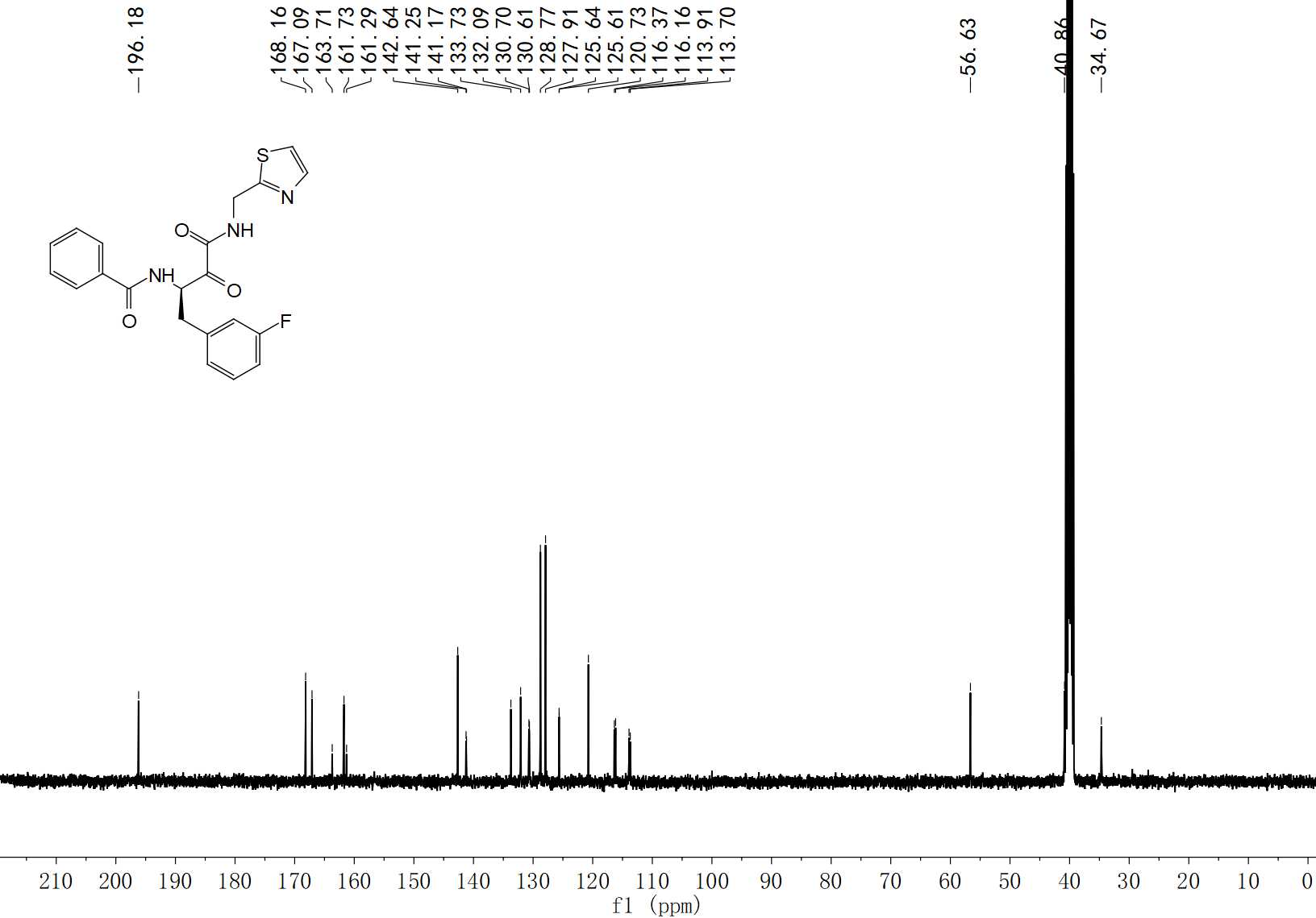


**^13^C NMR of 2b**


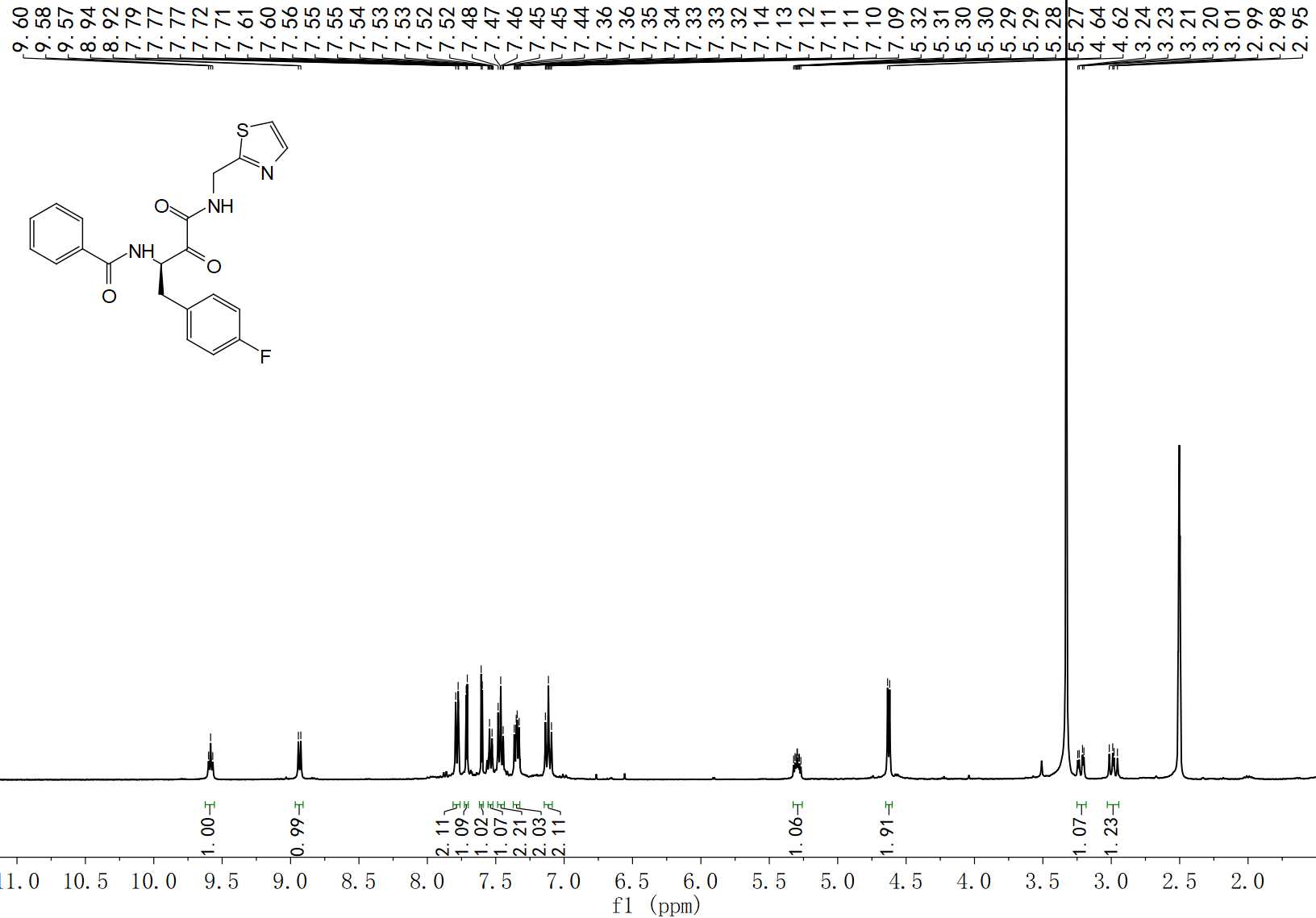


**^1^H NMR of 2c**


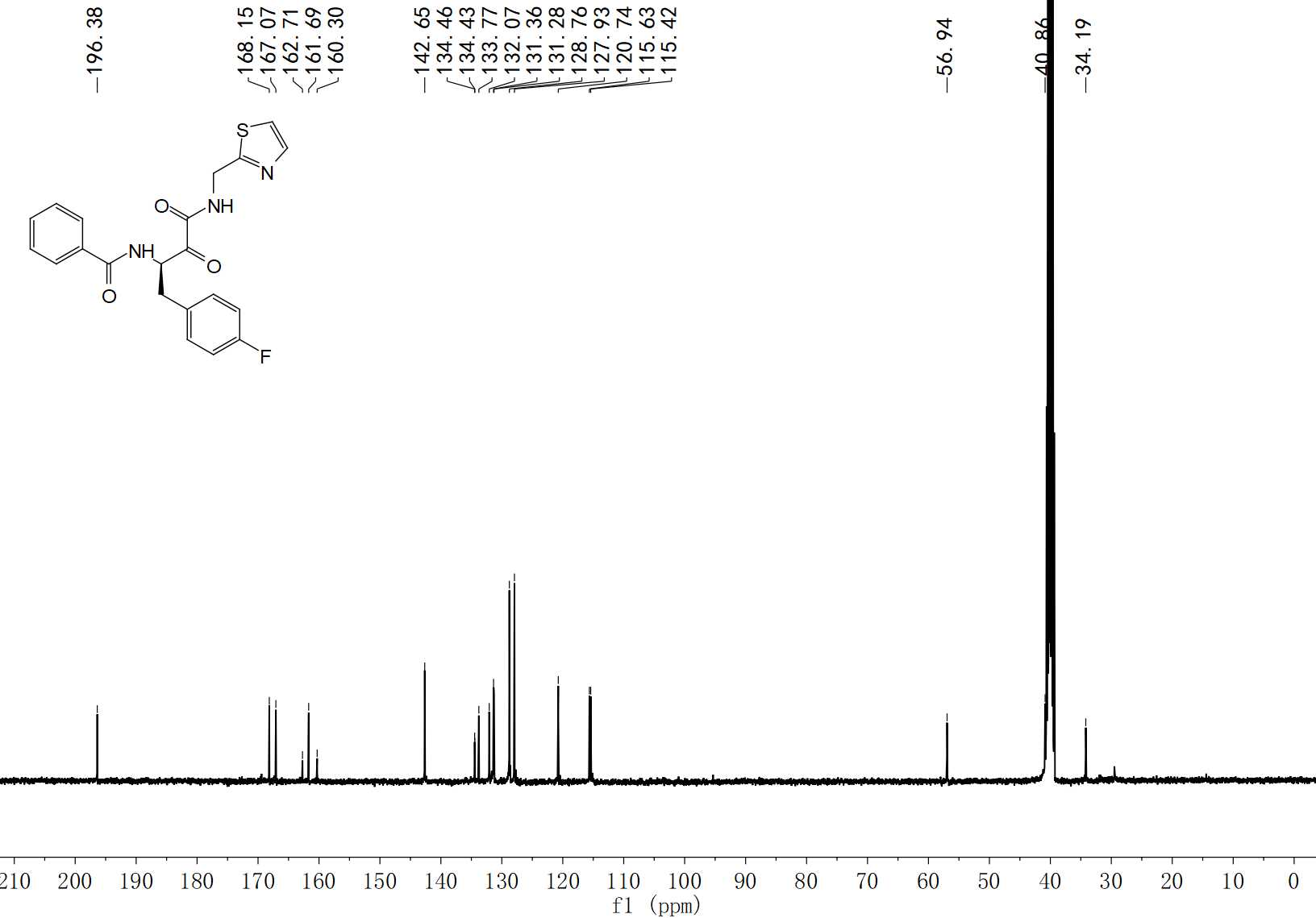


**^13^C NMR of 2c**


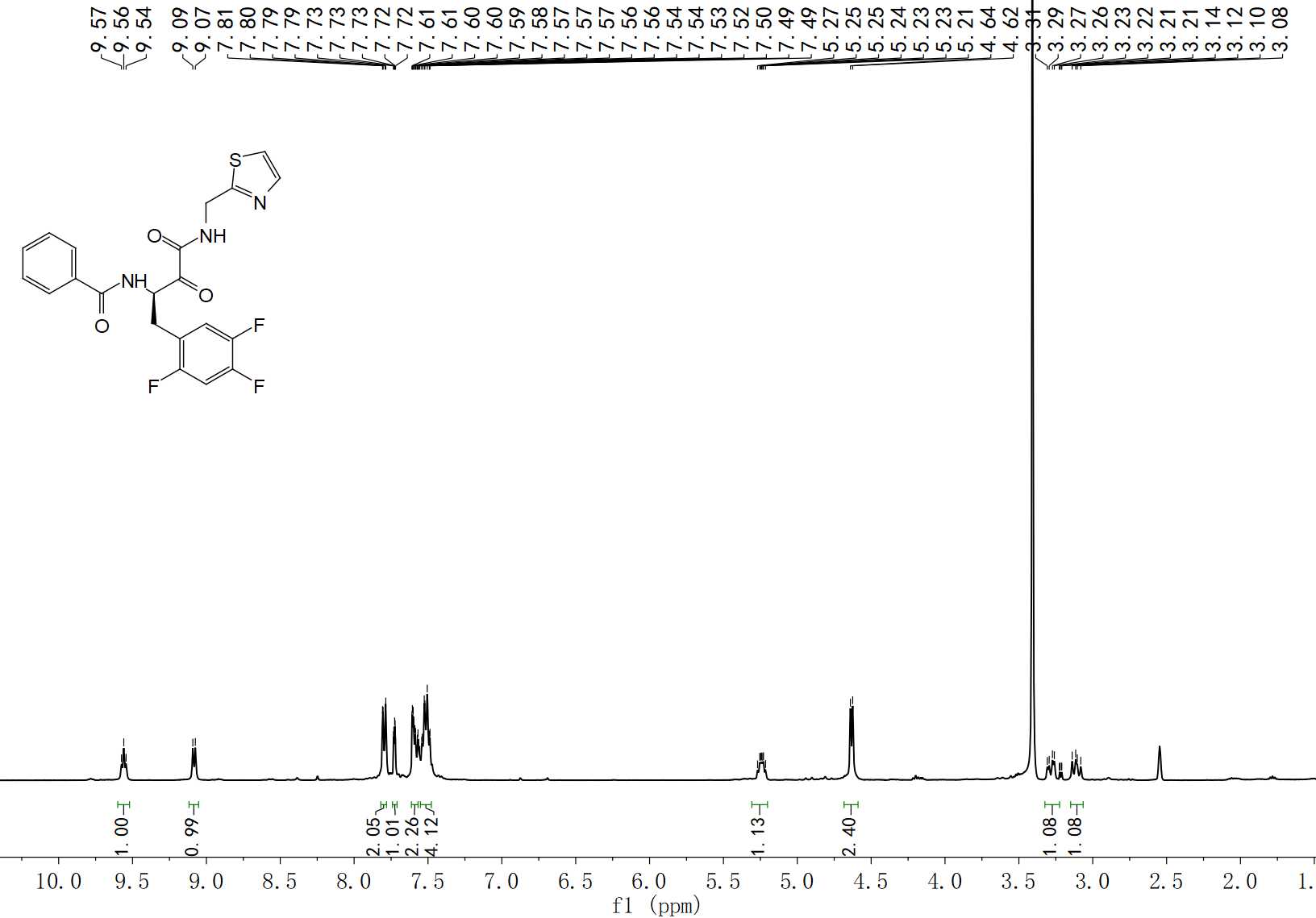


**^1^H NMR of 2d**


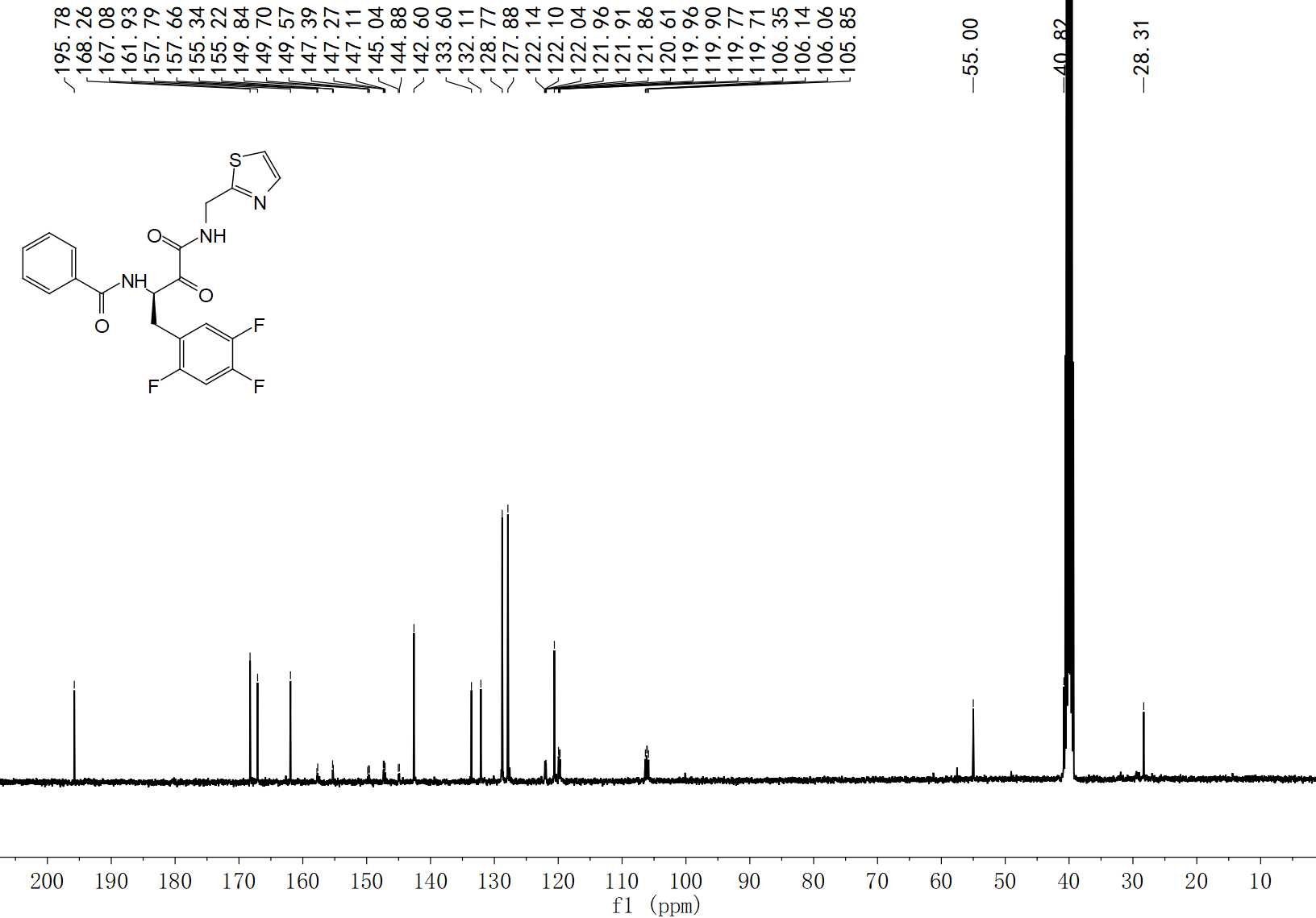


**^13^C NMR of 2d**


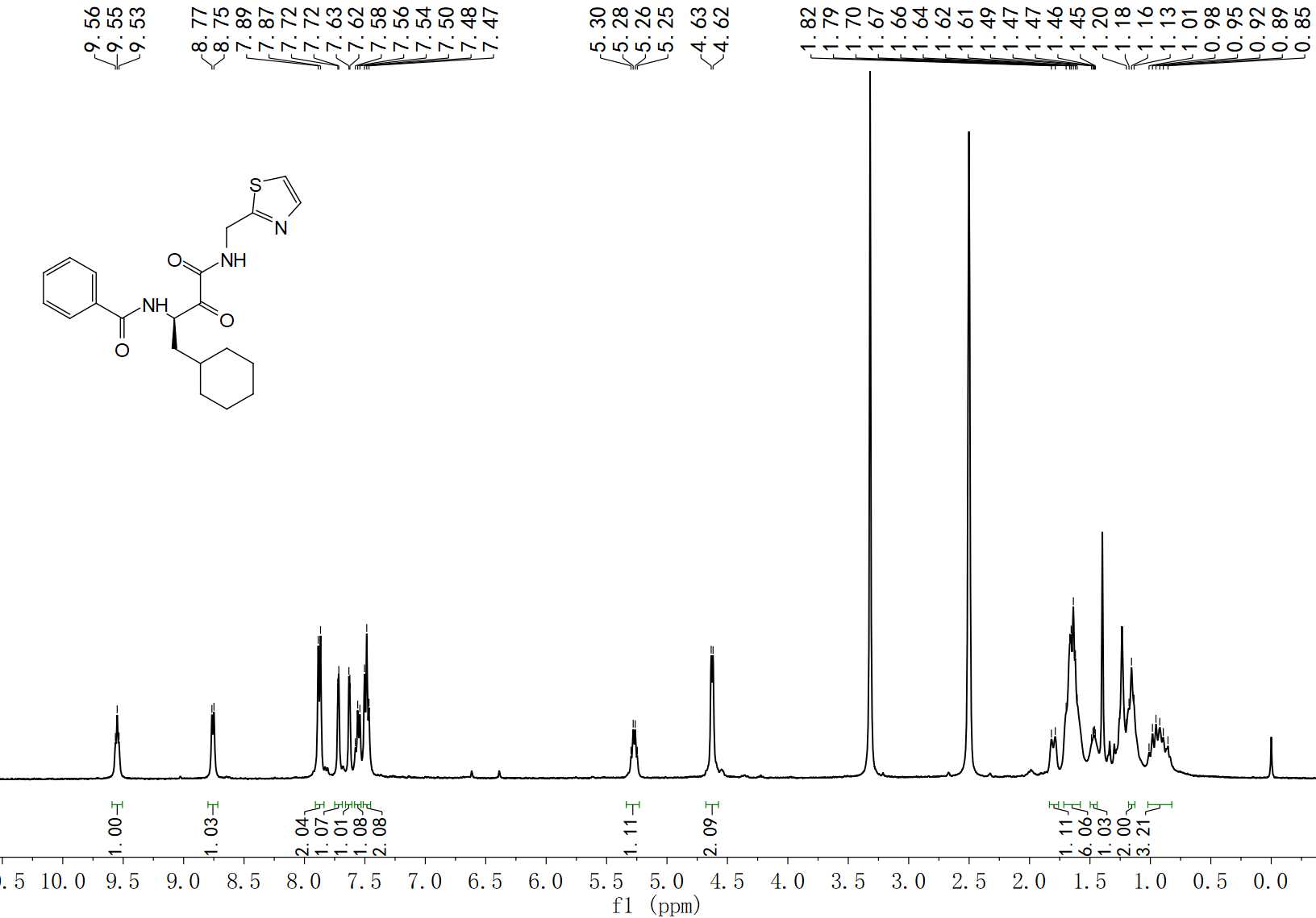


**^1^H NMR of 2e**


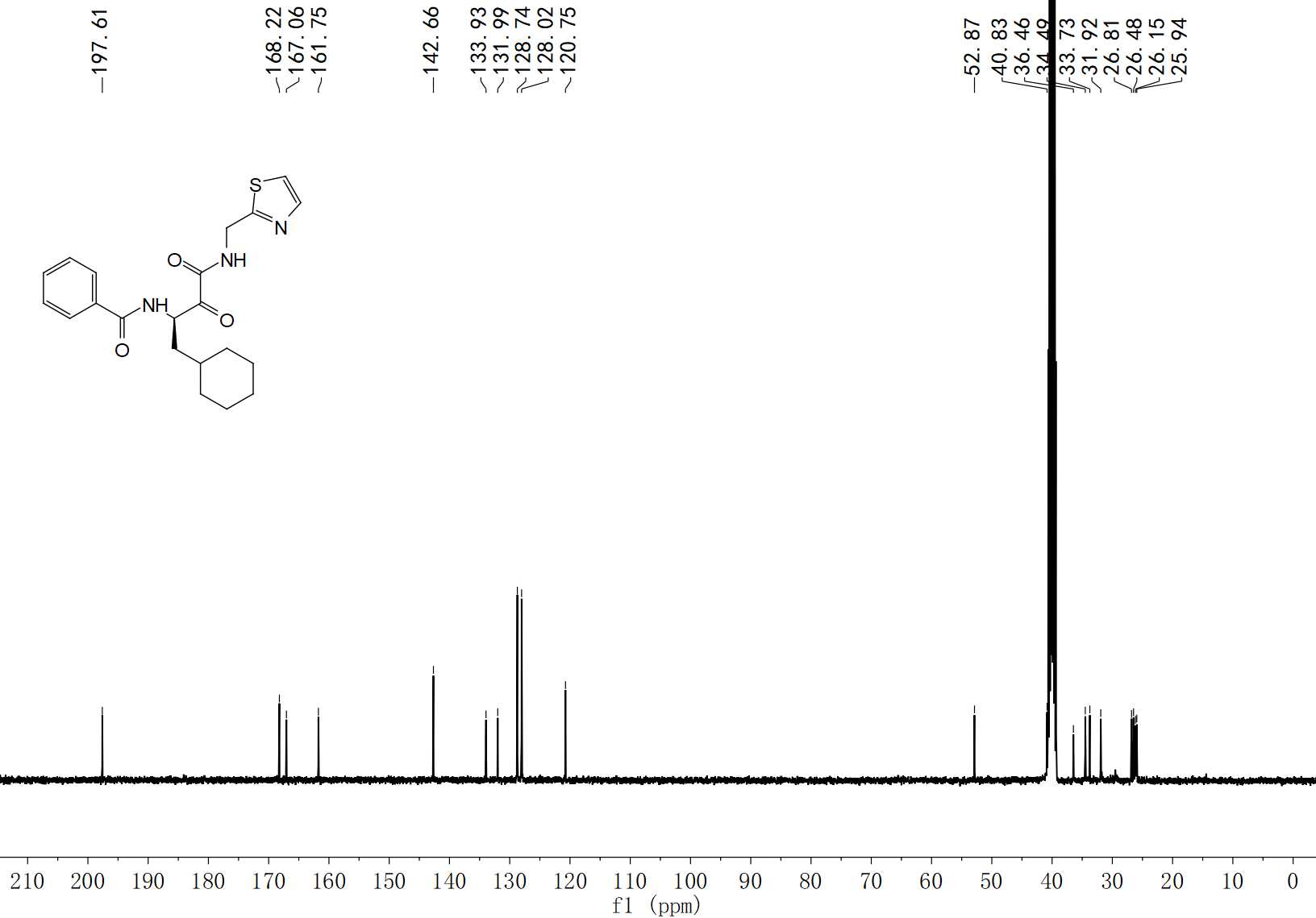


**^13^C NMR of 2e**


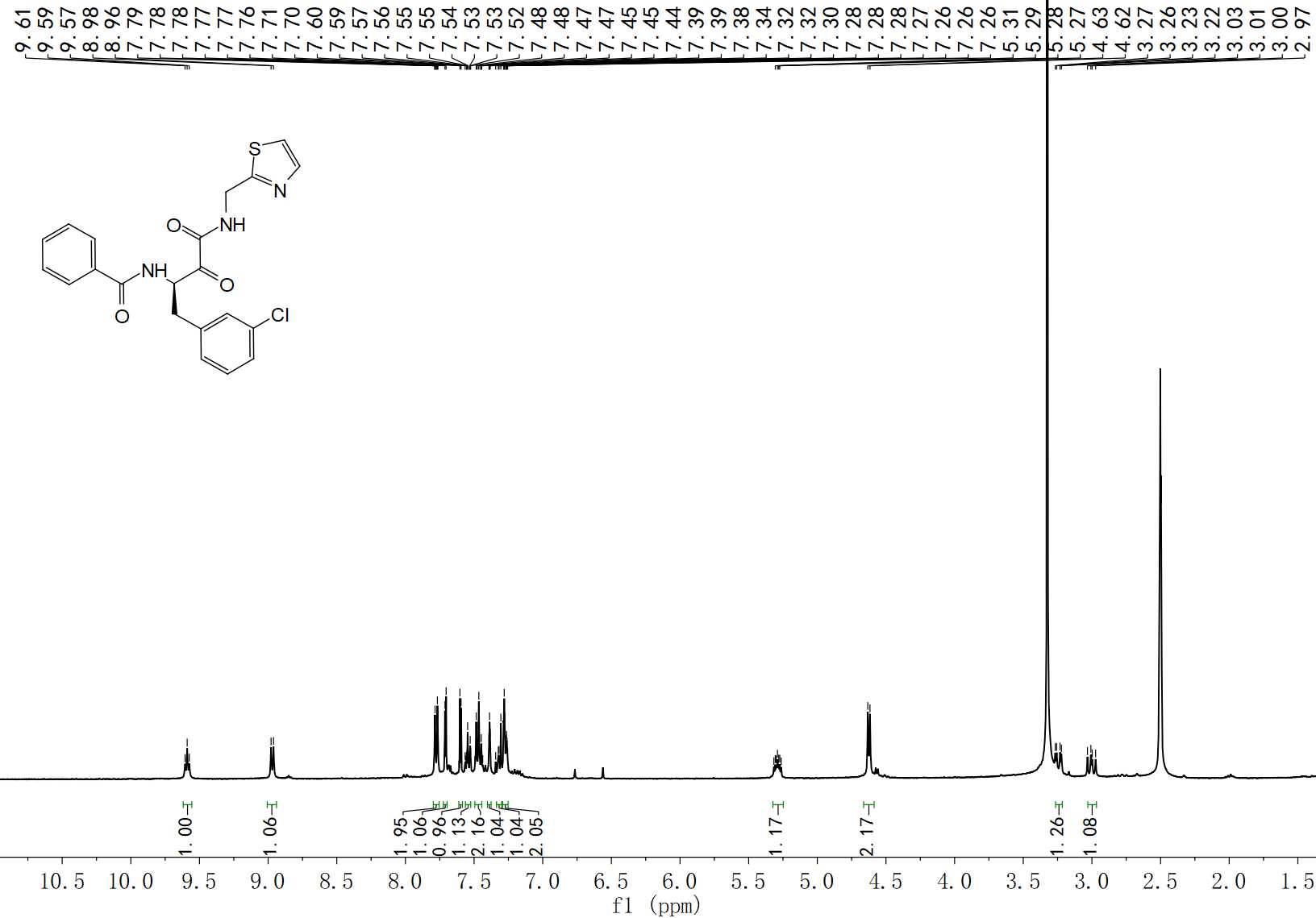


**^1^H NMR of 2f**


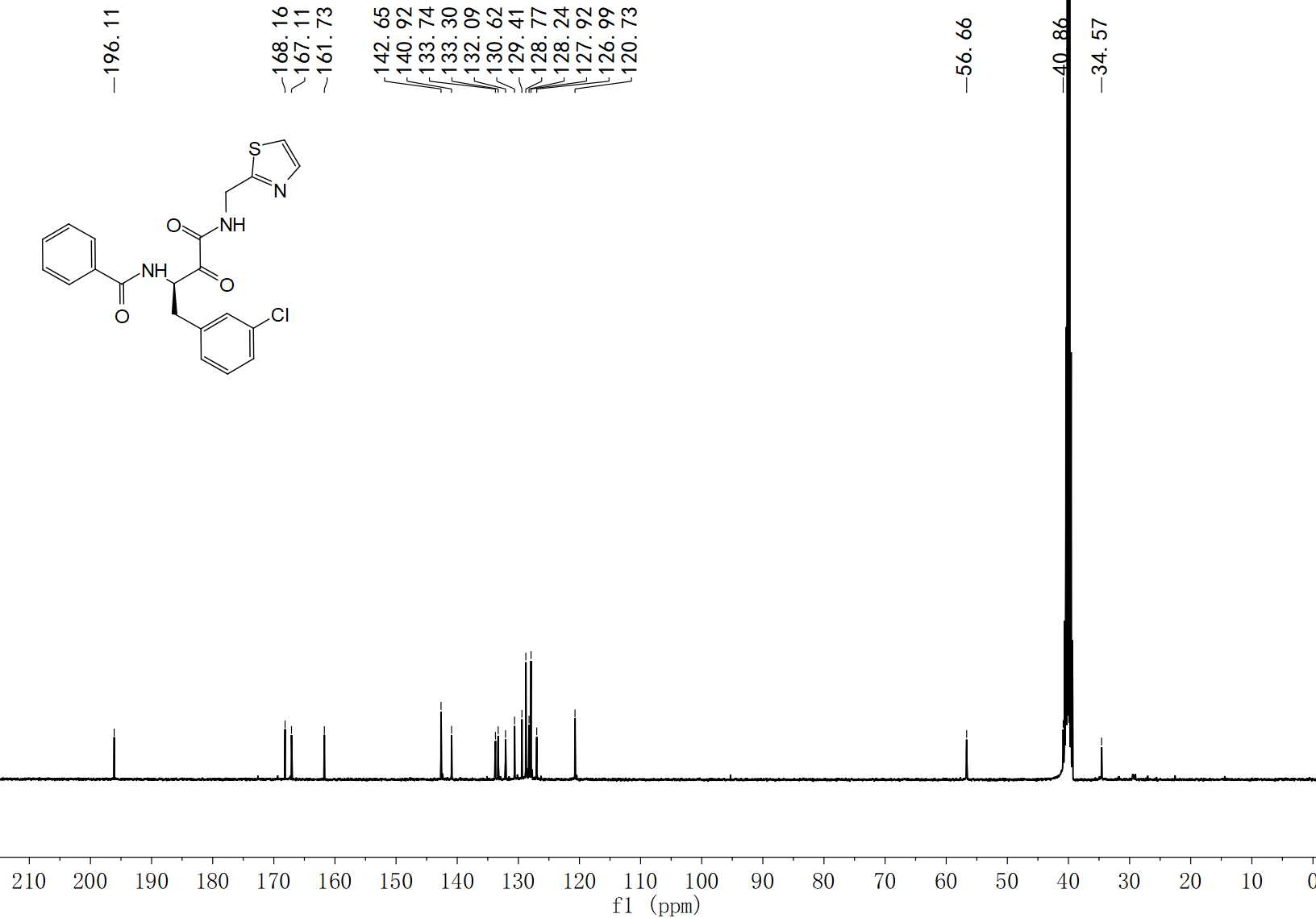


**^13^C NMR of 2f**


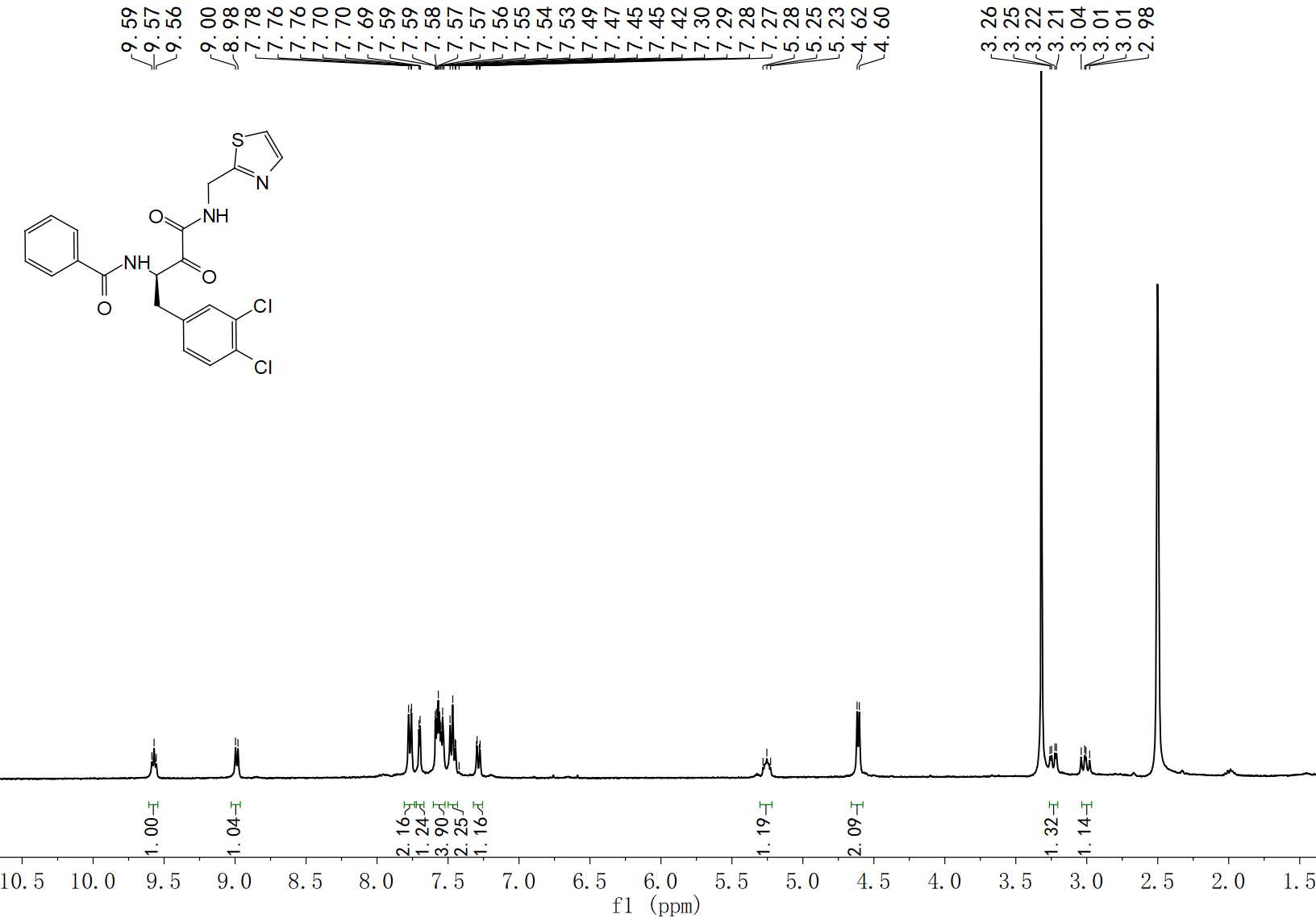


**^1^H NMR of 2g**


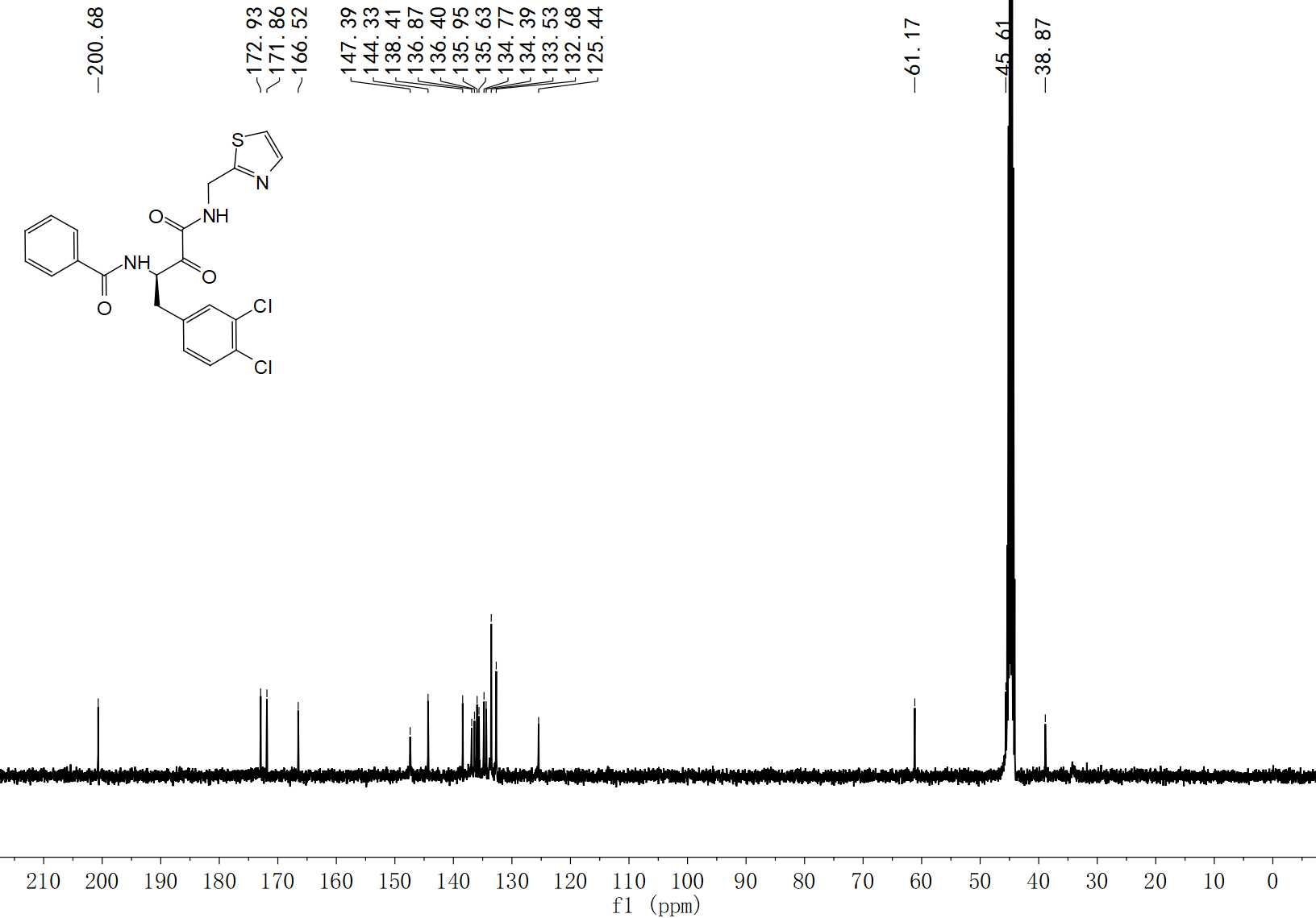


**^13^C NMR of 2g**


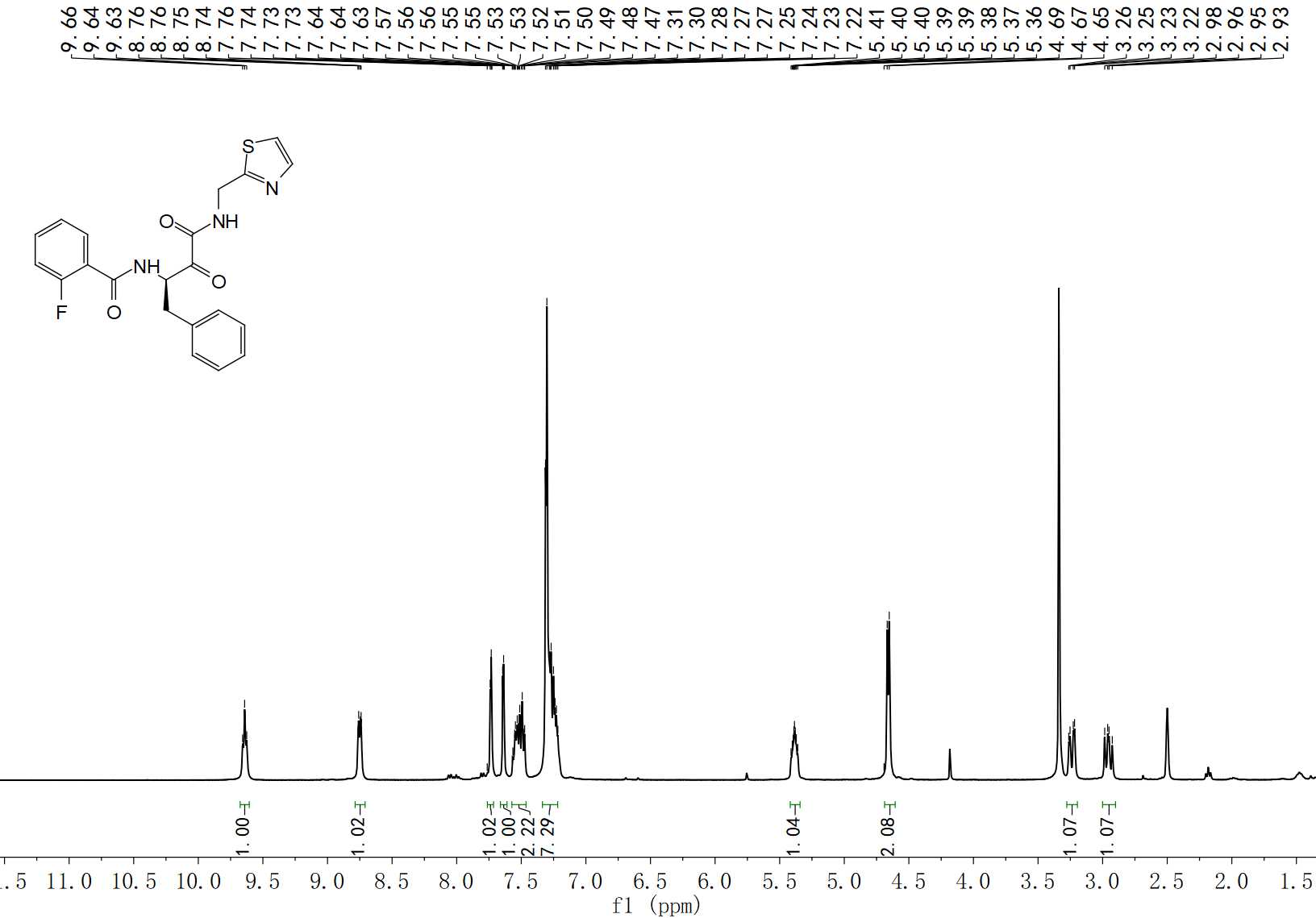


**^1^H NMR of 3a**


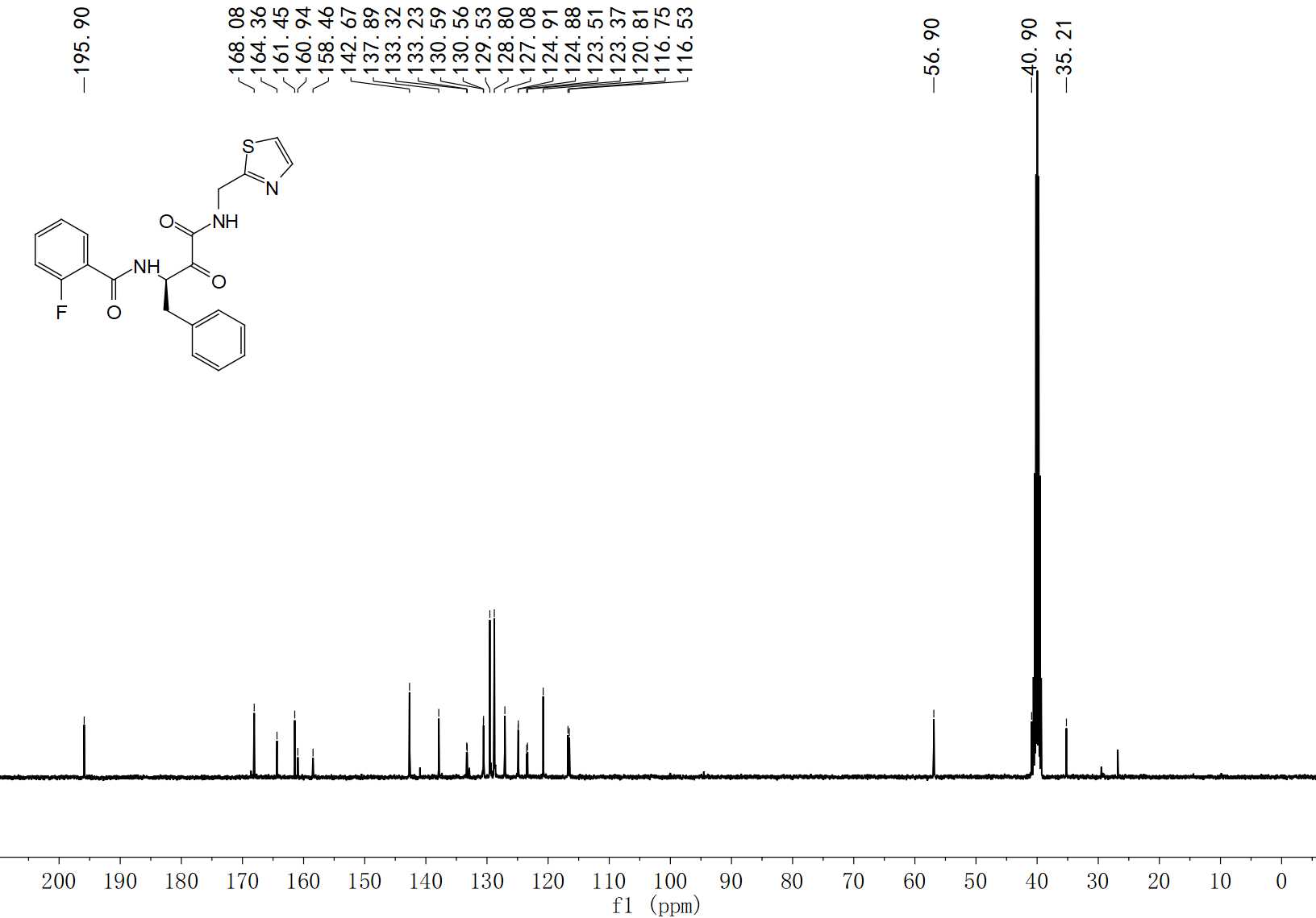


**^13^C NMR of 3a**


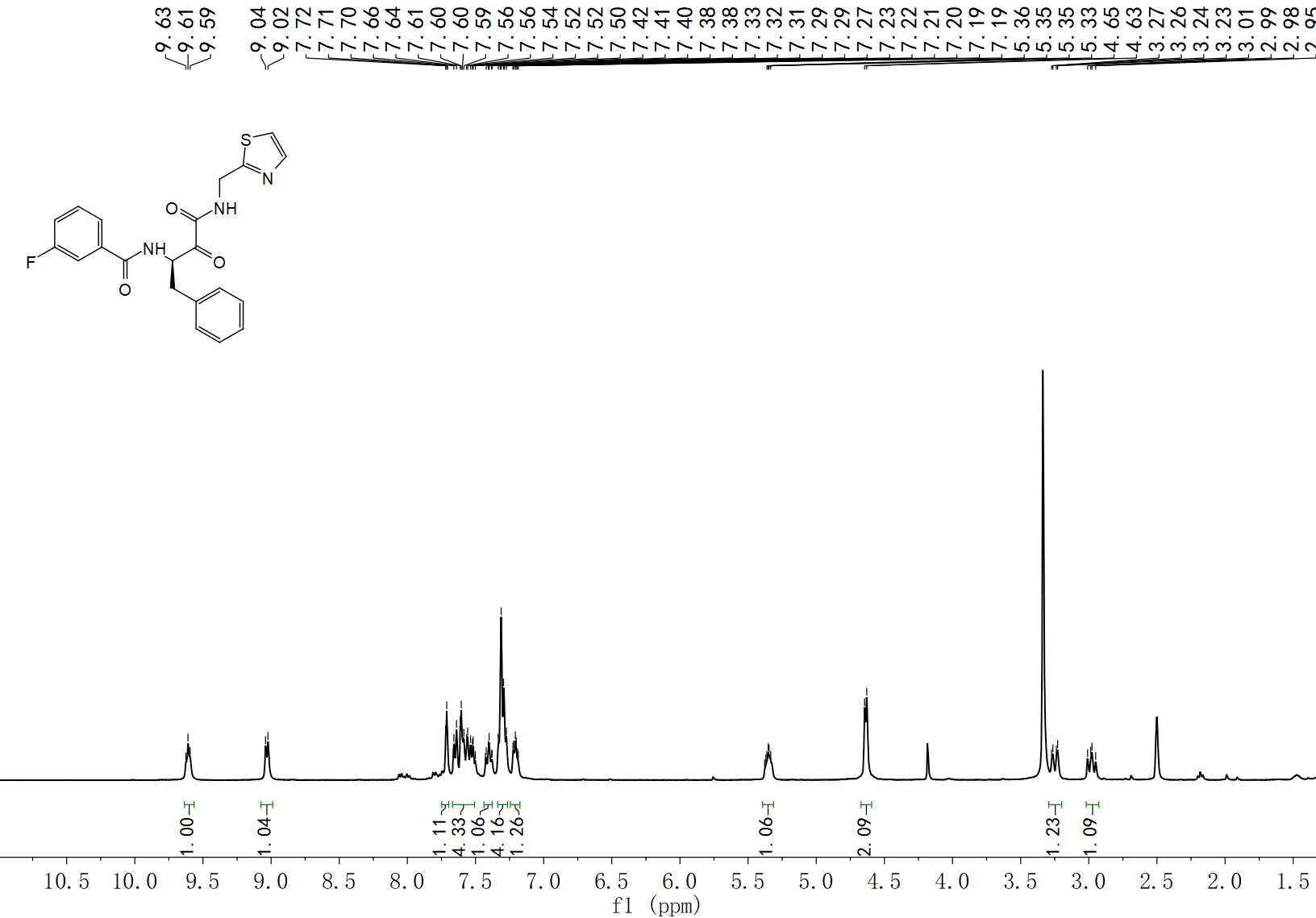


**^1^H NMR of 3b**


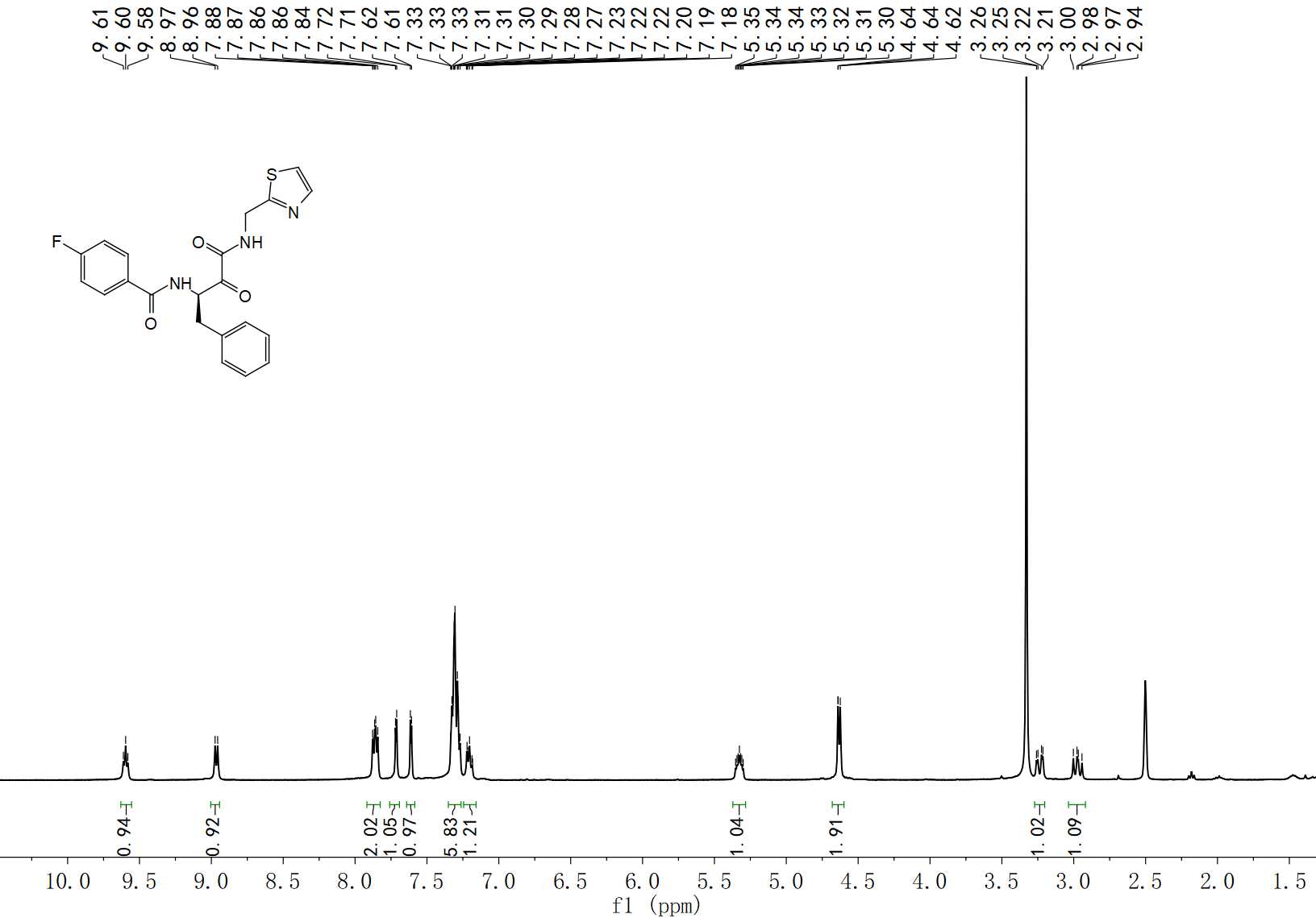


**^1^H NMR of 3c**


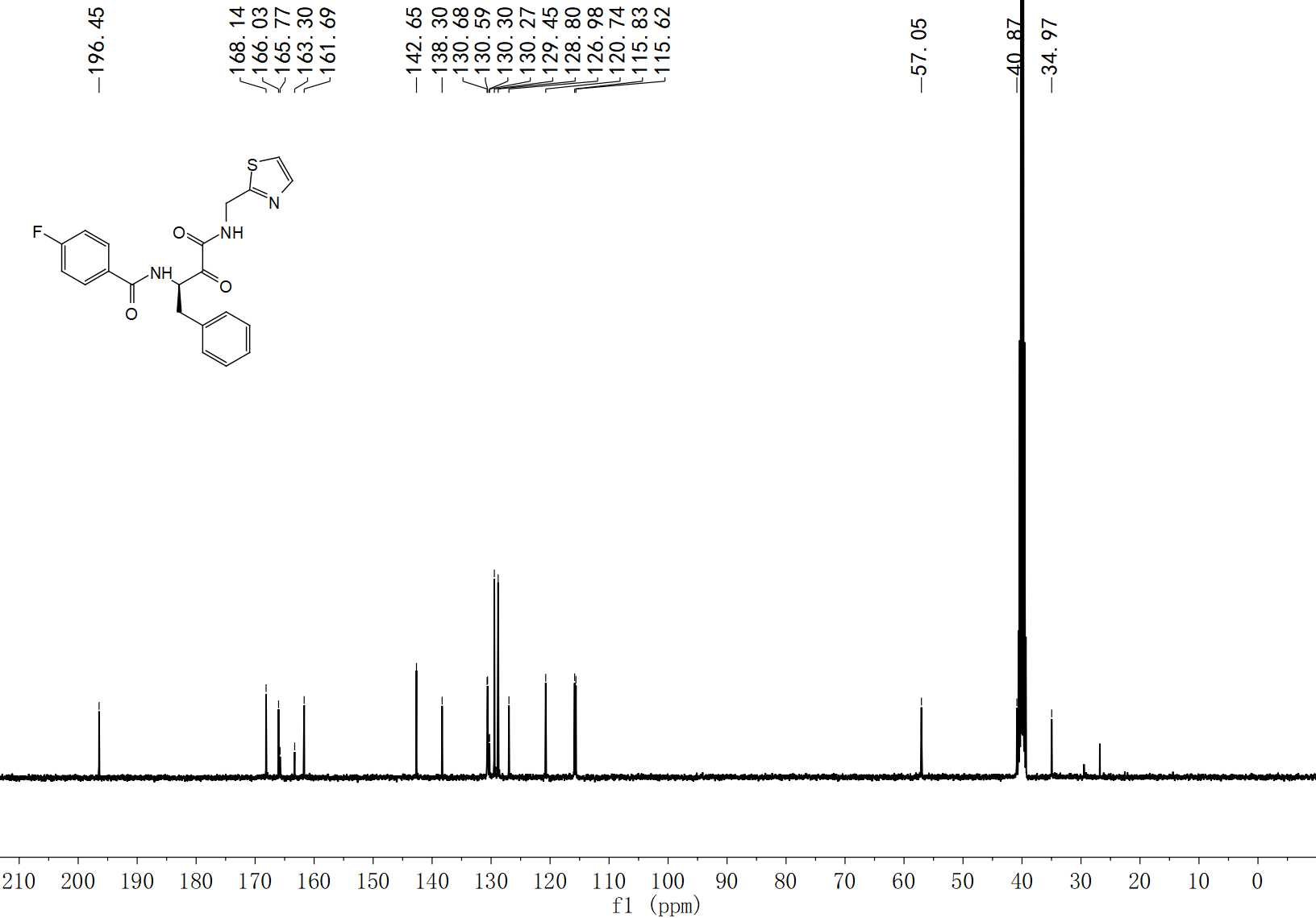


**^13^C NMR of 3c**


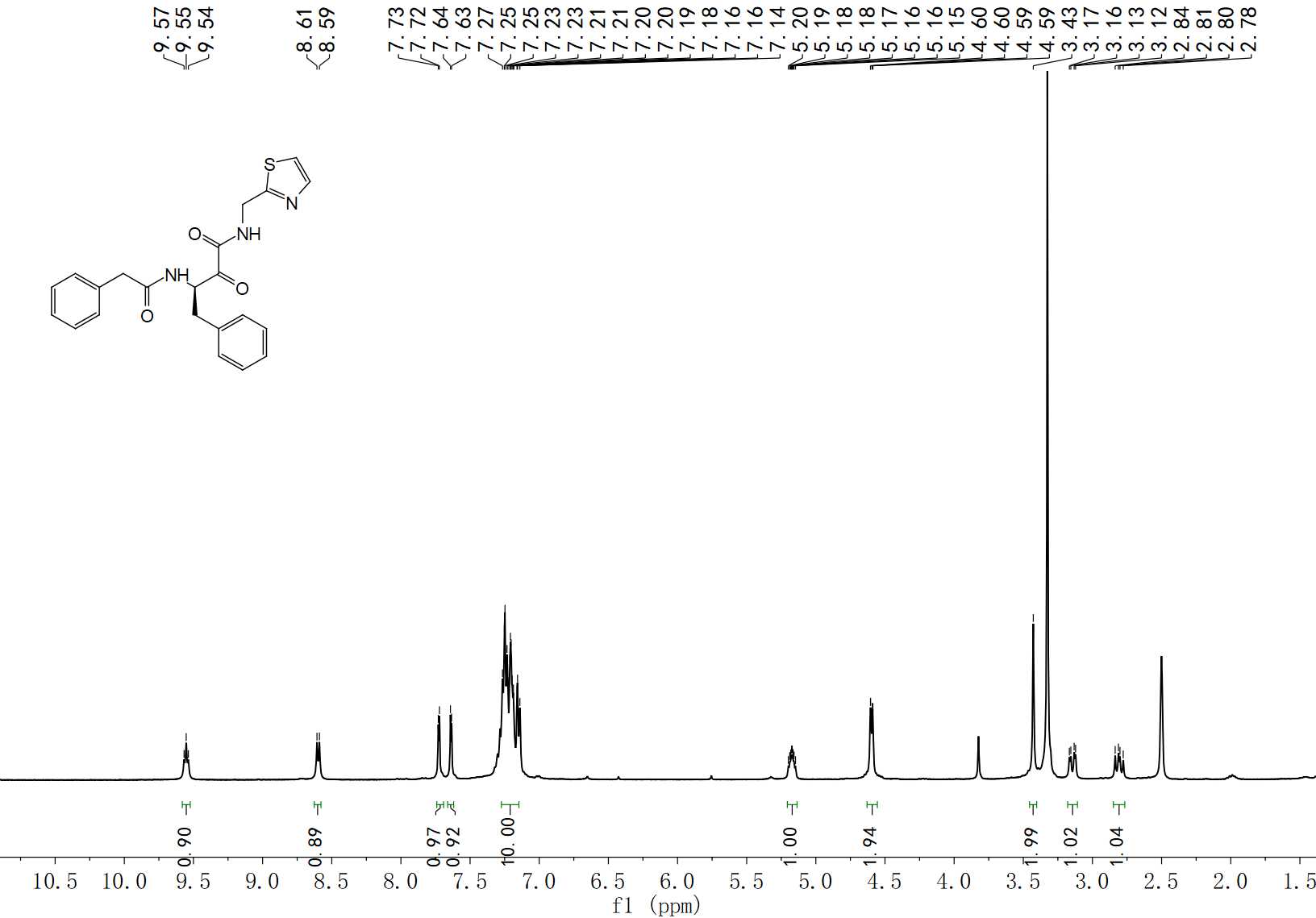


**^1^H NMR of 3d**


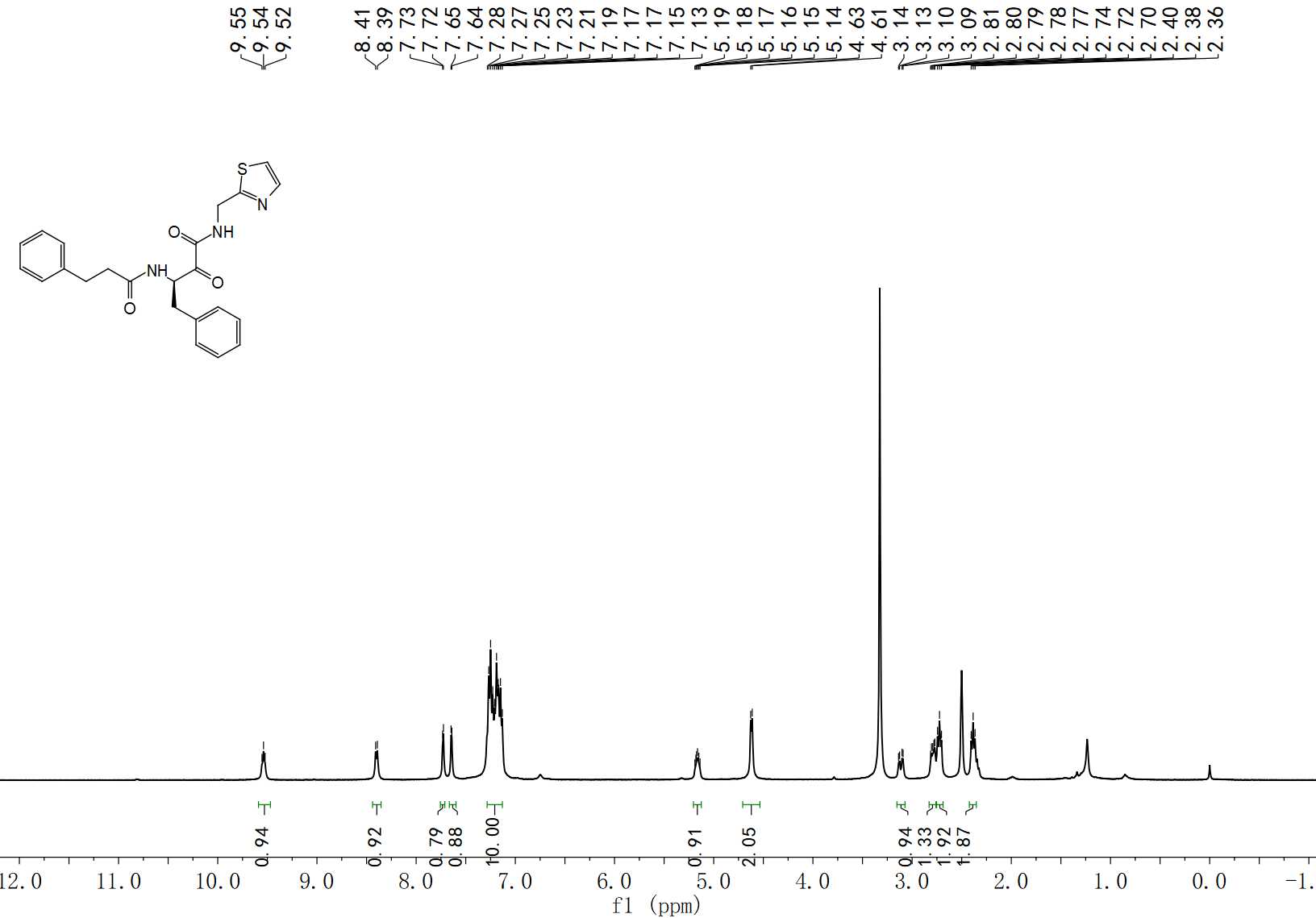


**^1^H NMR of 3e**


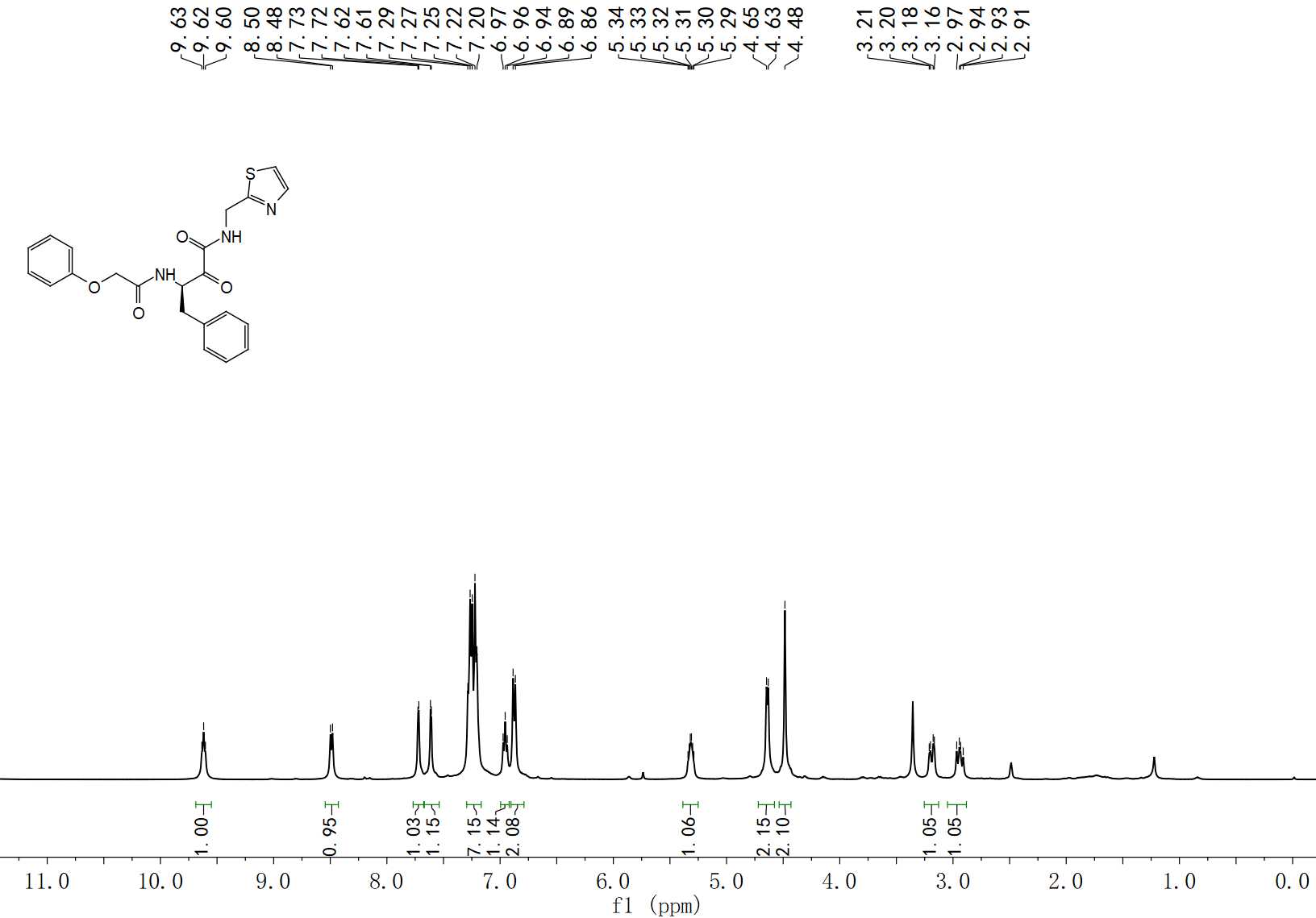


**^1^H NMR of 3f**


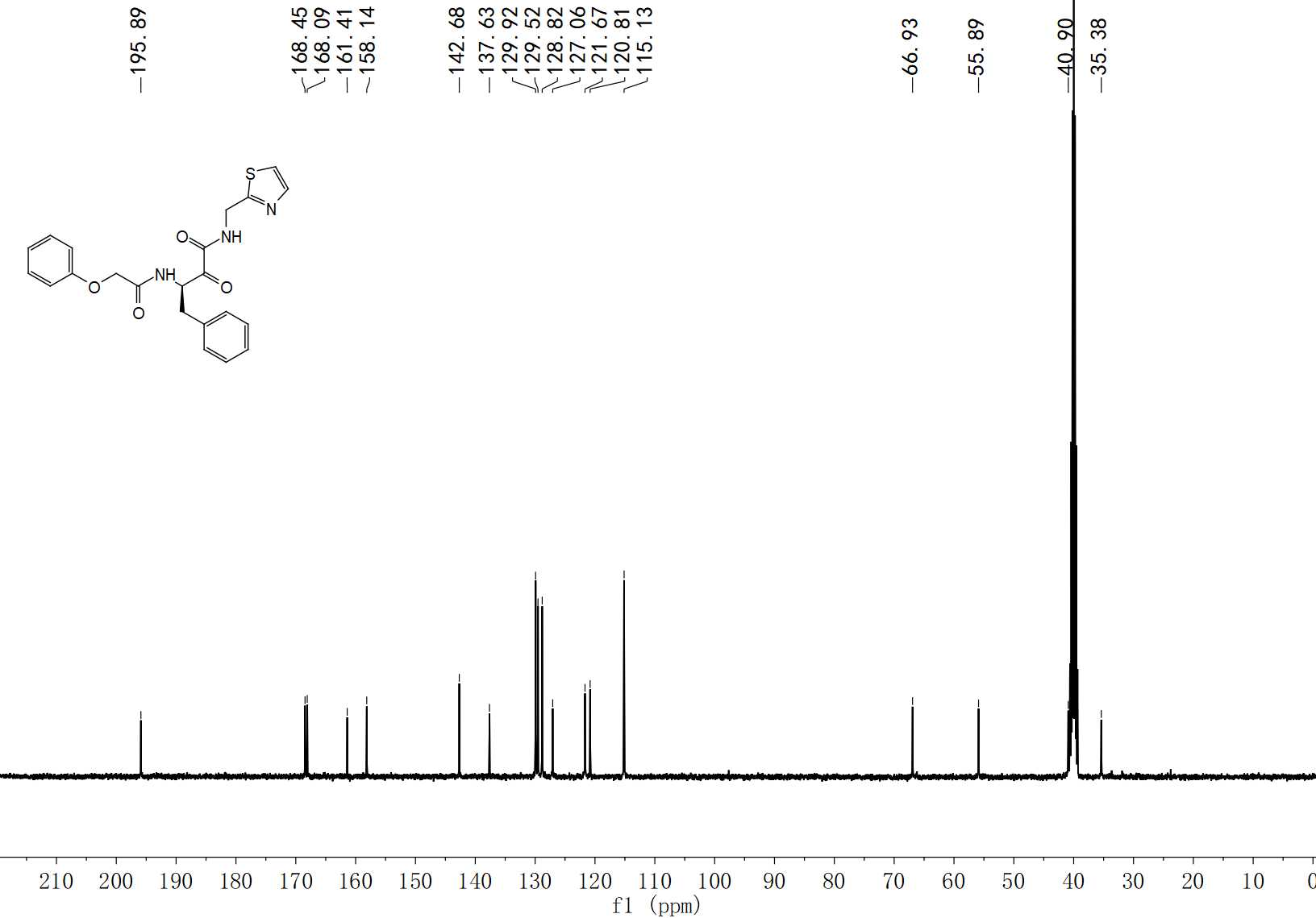


**^13^C NMR of 3f**


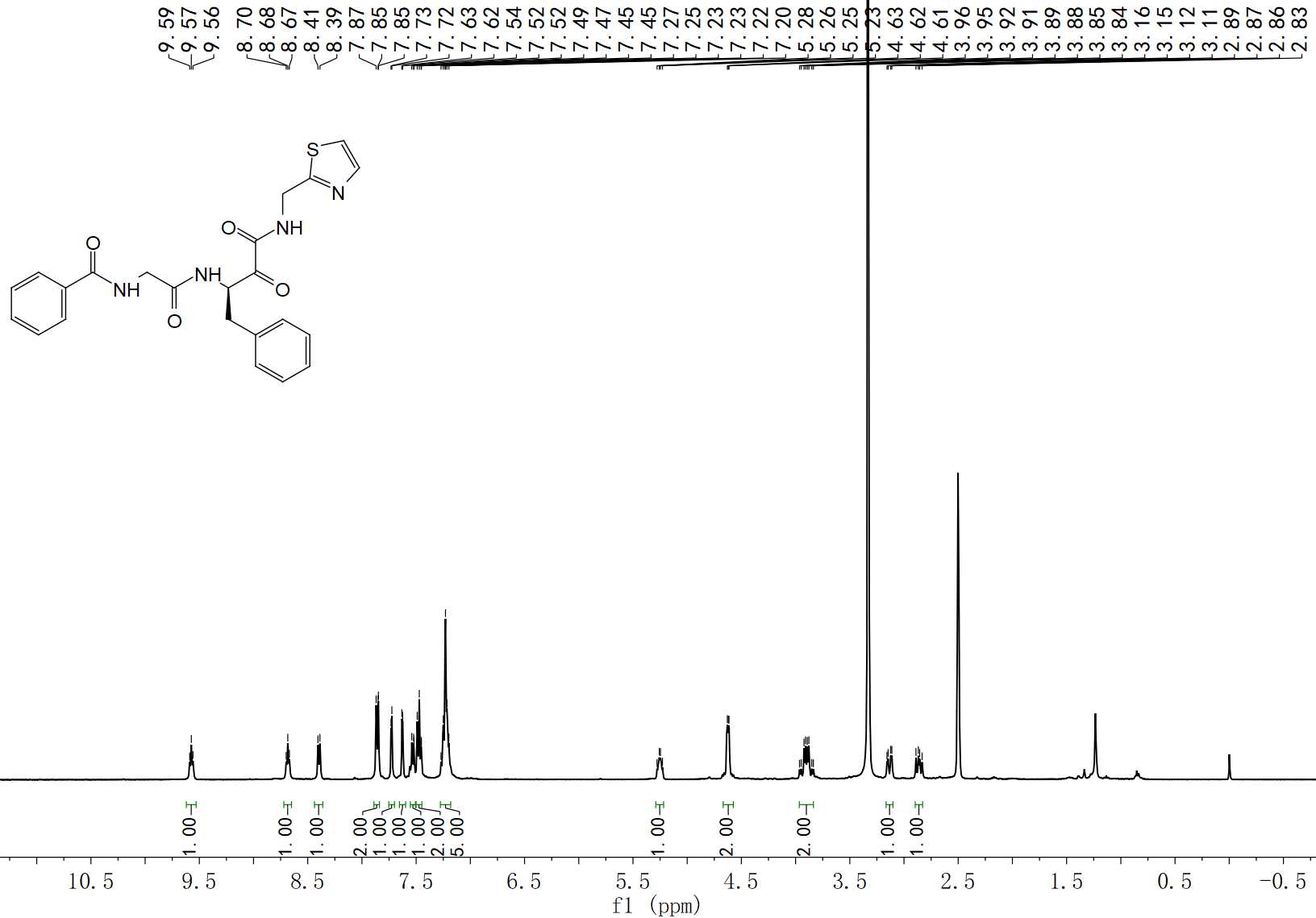


**^1^H NMR of 3g**


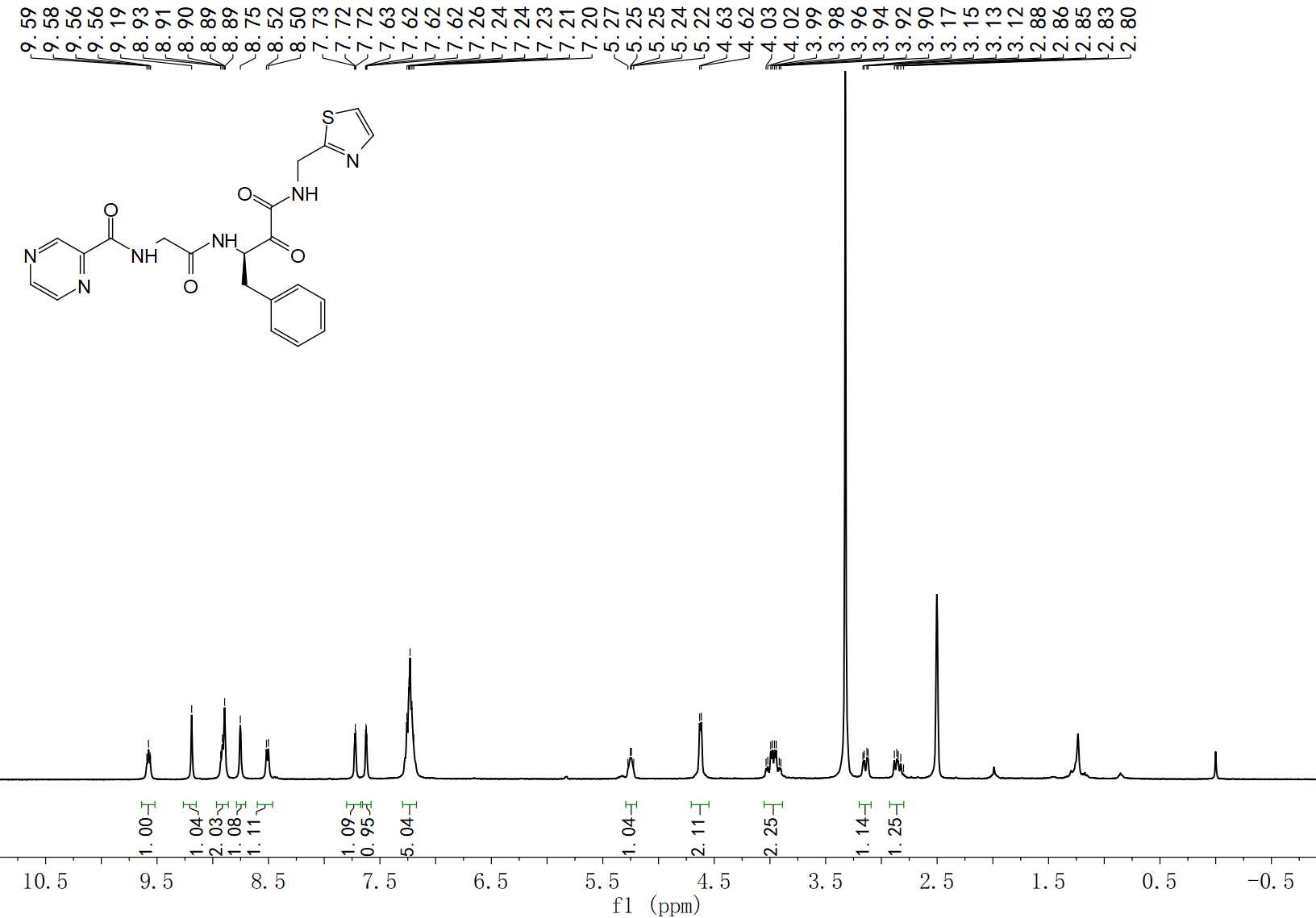


**^1^H NMR of 3h**


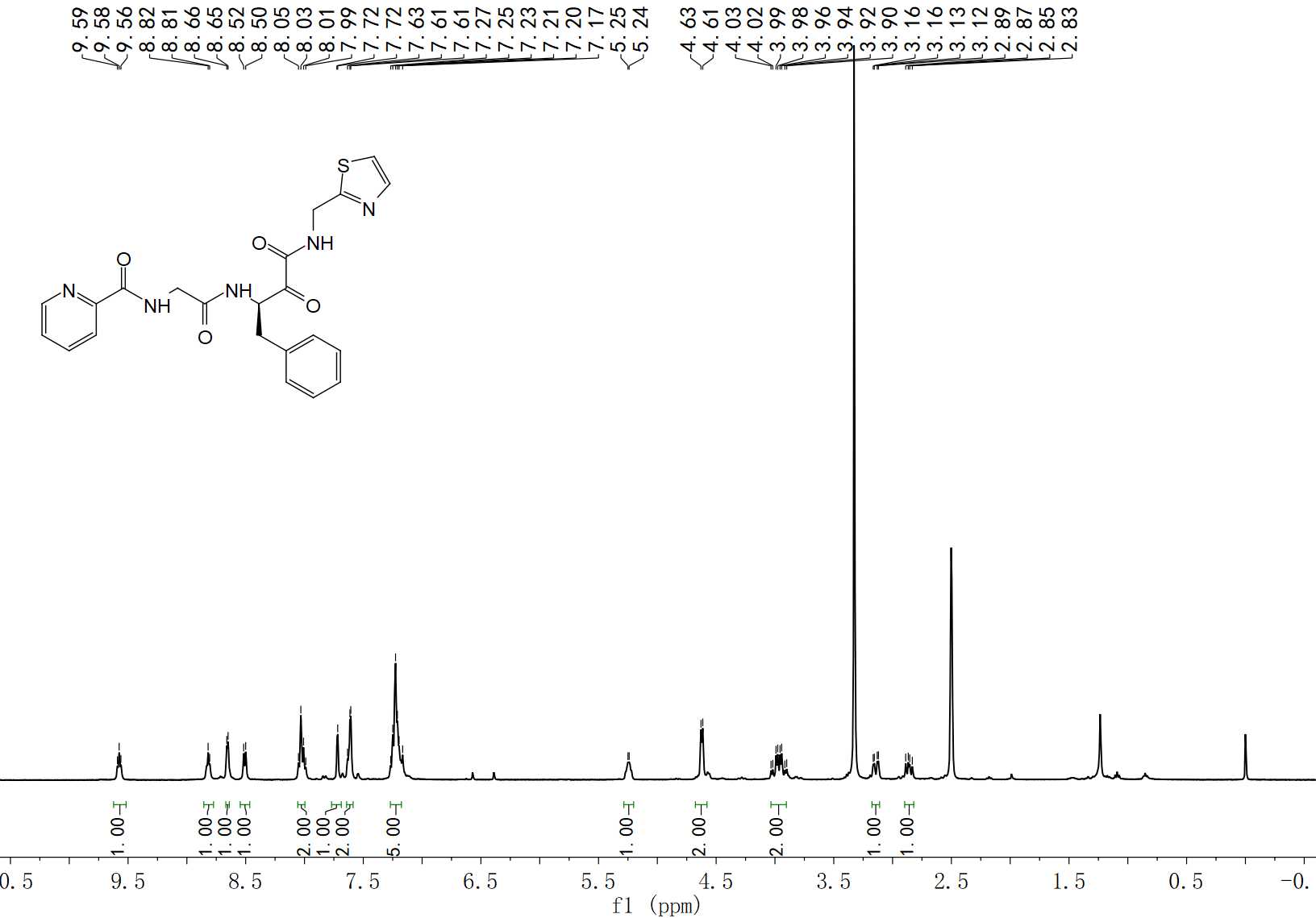


**^1^H NMR of 3i**


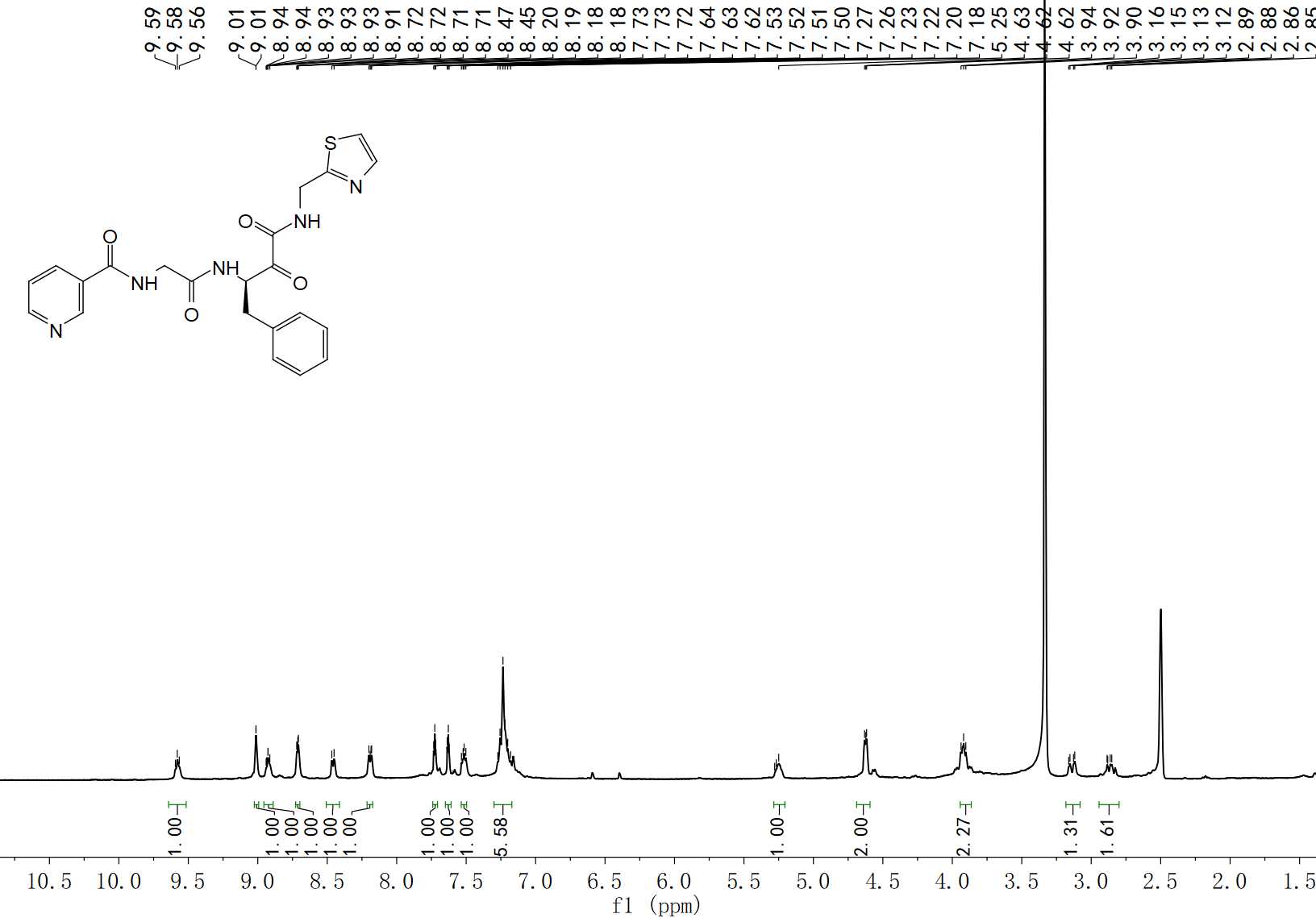


**^1^H NMR of 3j**


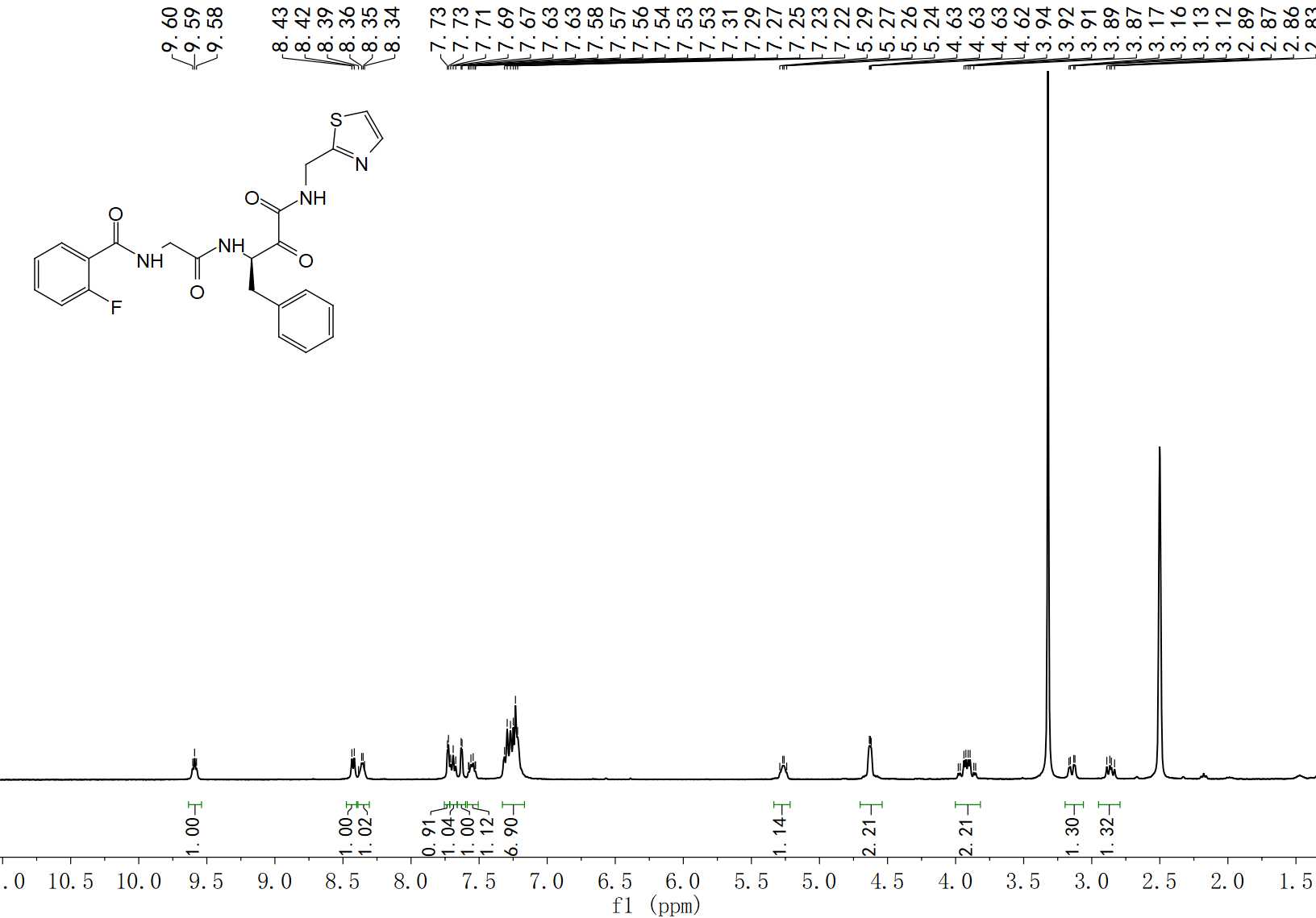


**^1^H NMR of 3k**


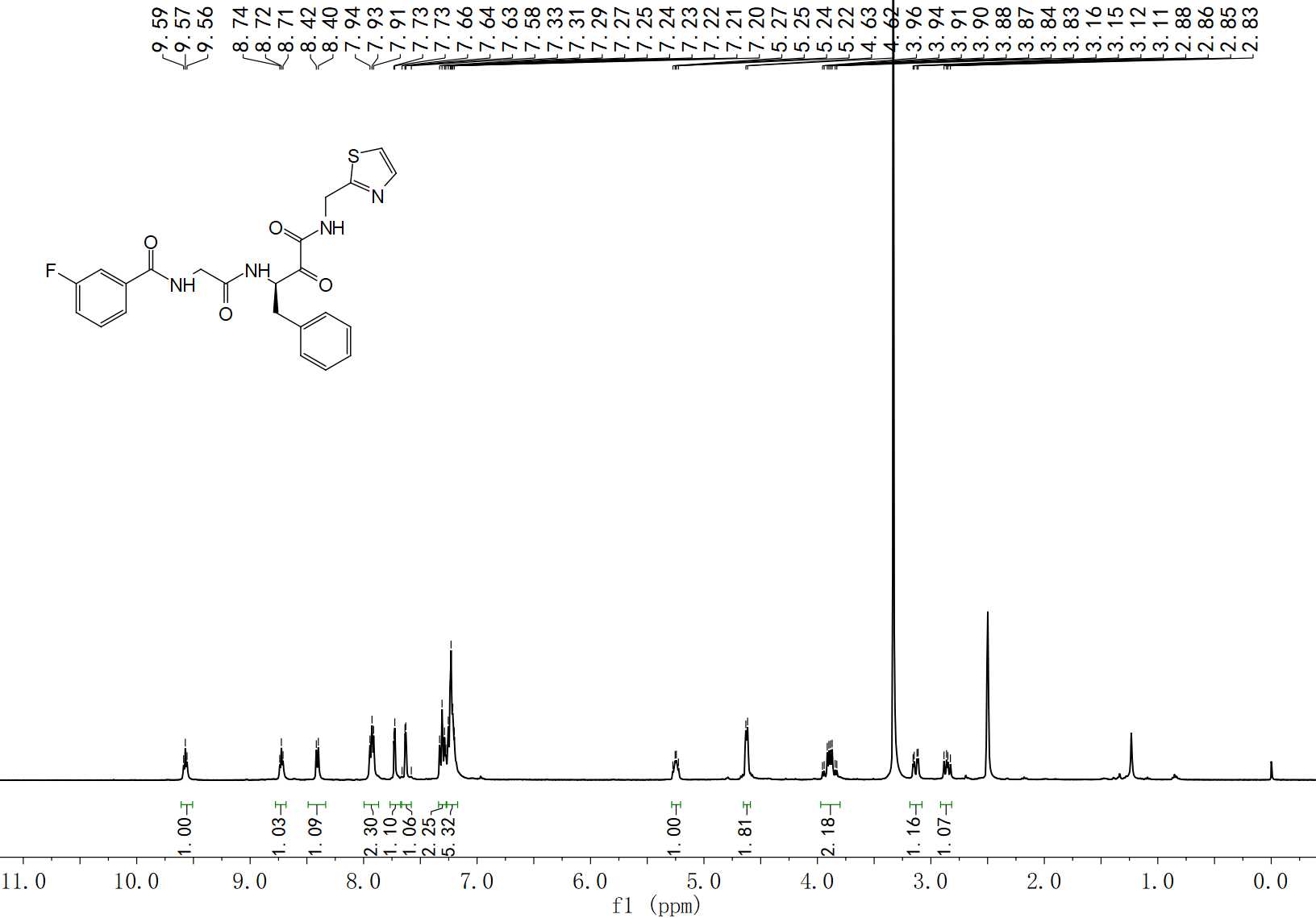


**^1^H NMR of 3l**


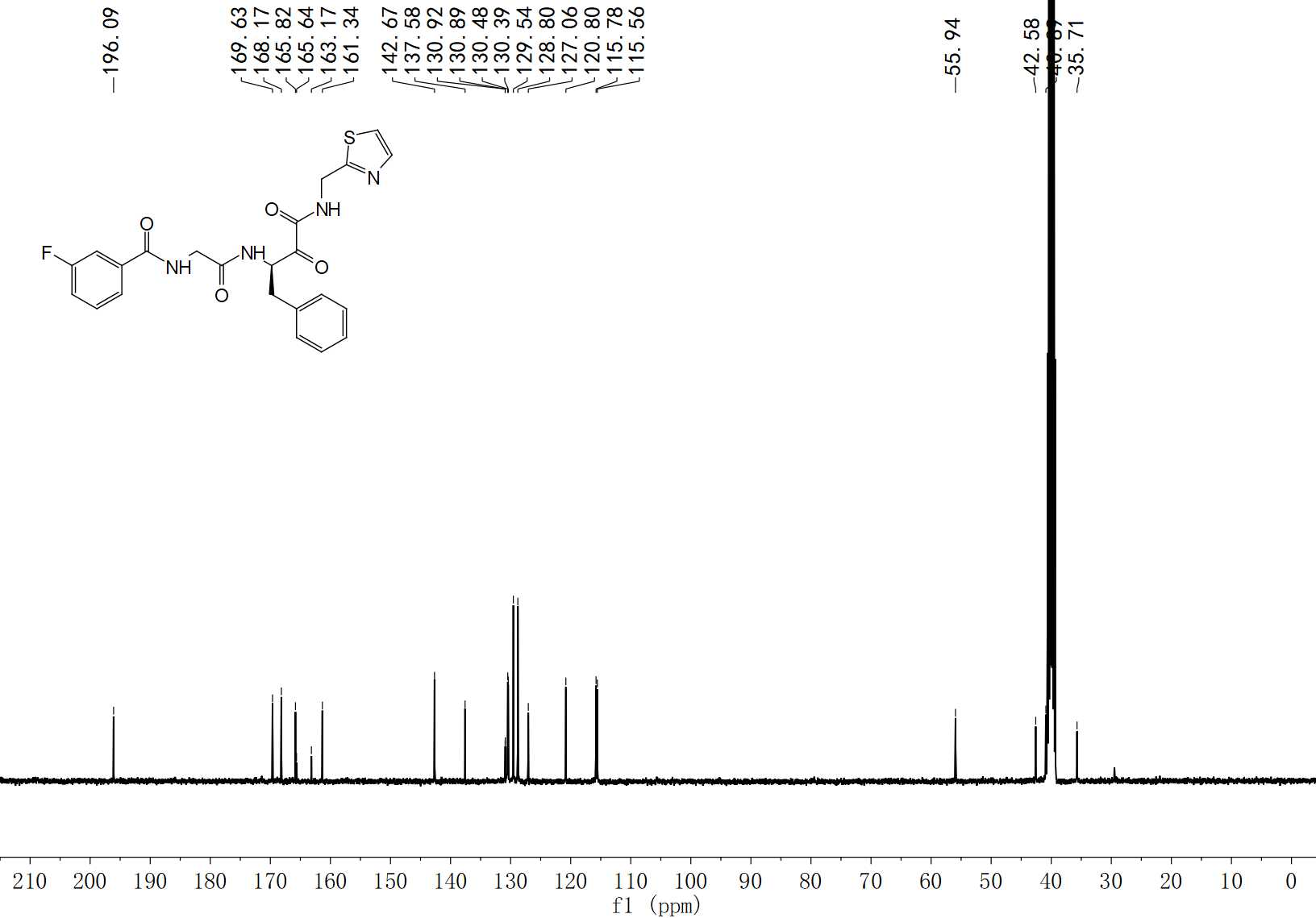


**^13^C NMR of 3l**


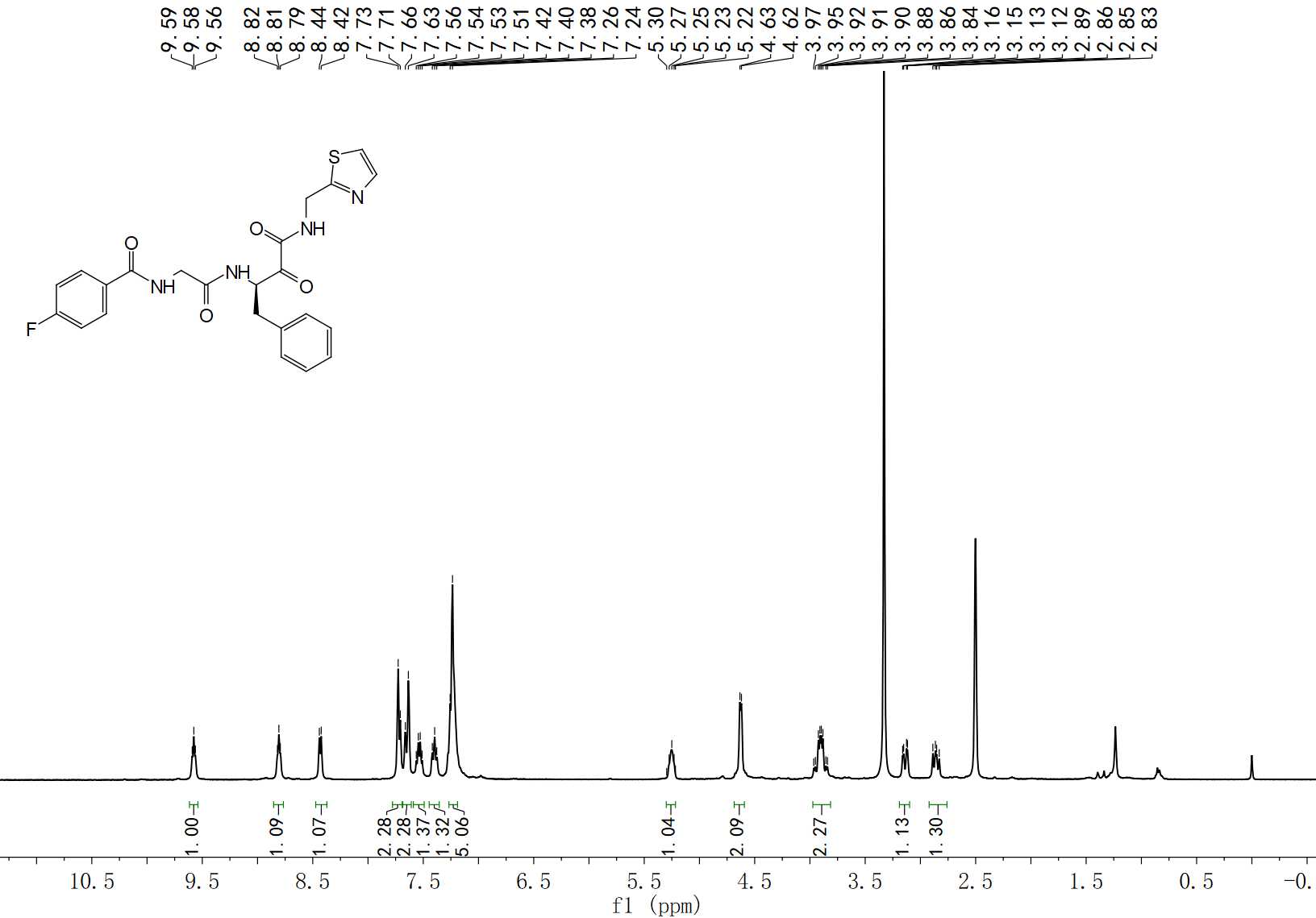


**^1^H NMR of 3m**


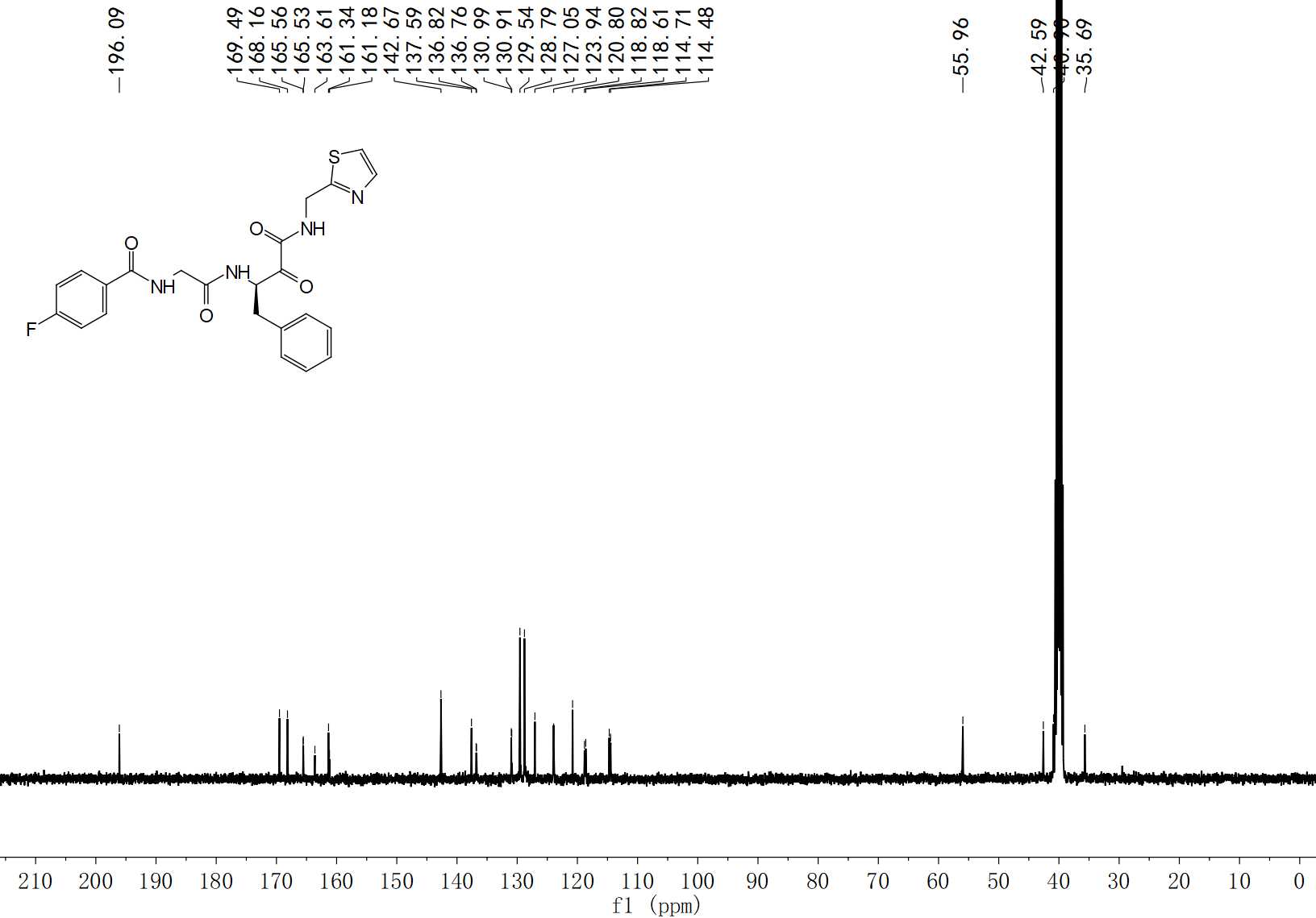


**^13^C NMR of 3m**


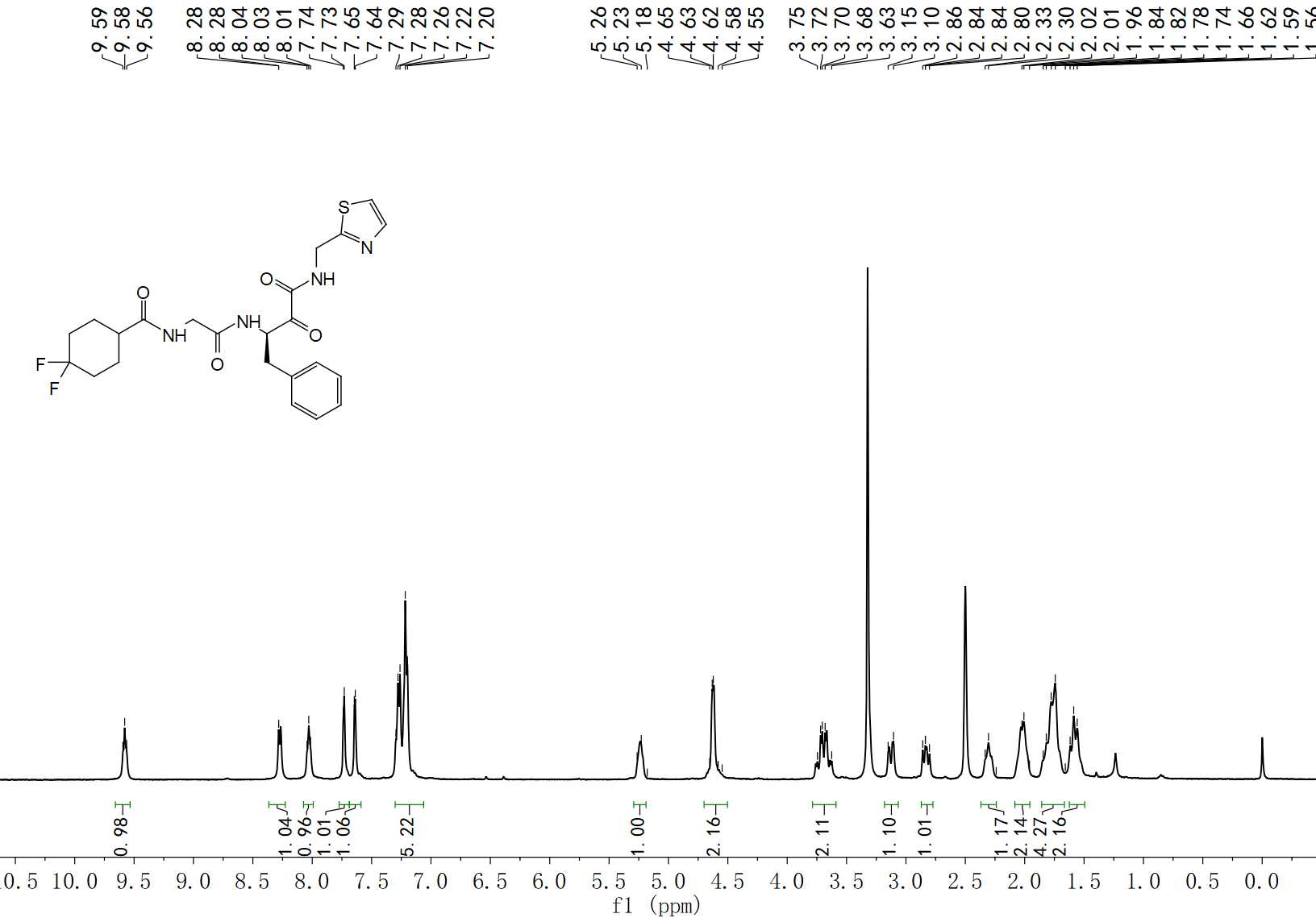


**^1^H NMR of 3n**


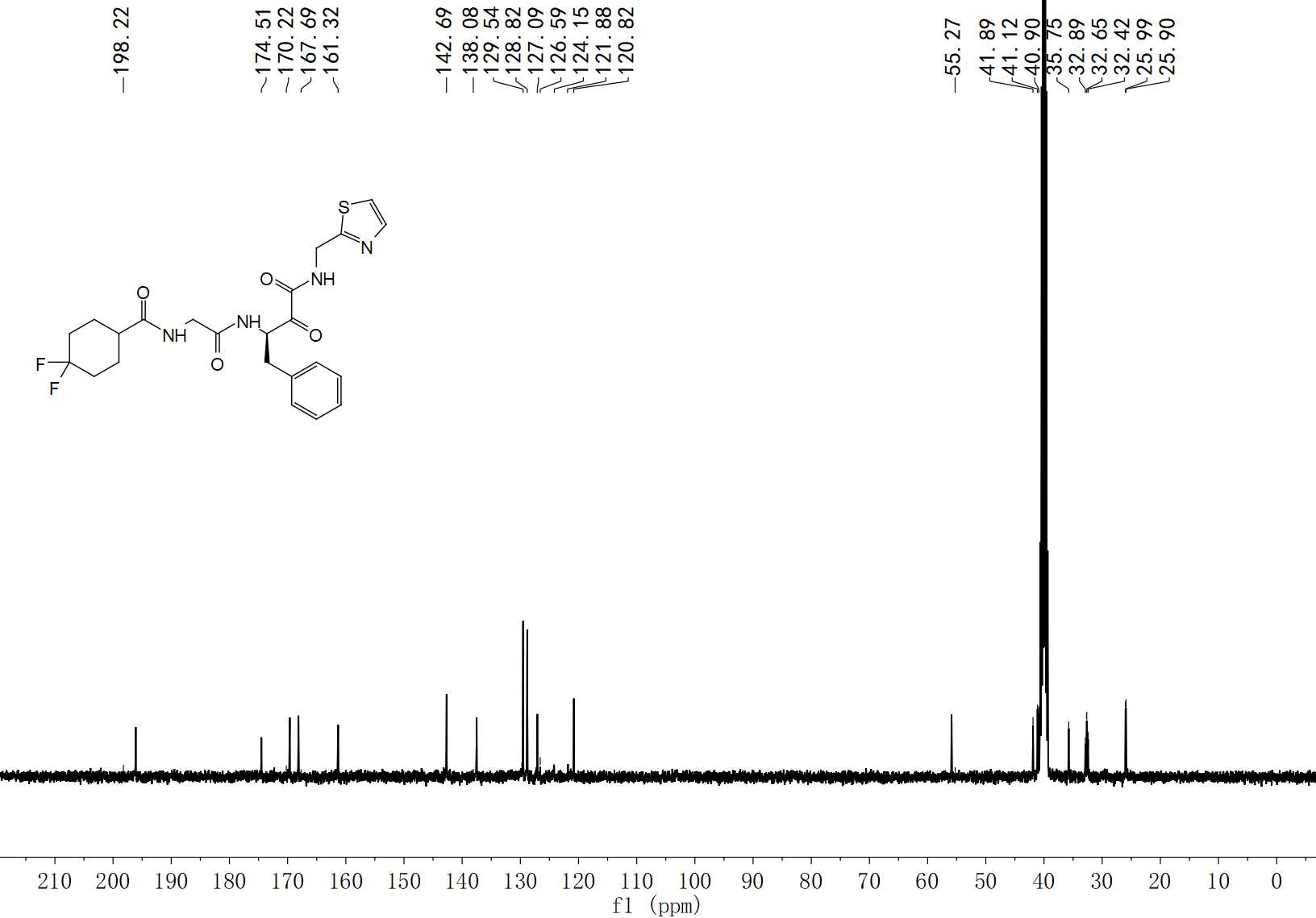


**^13^C NMR of 3n**


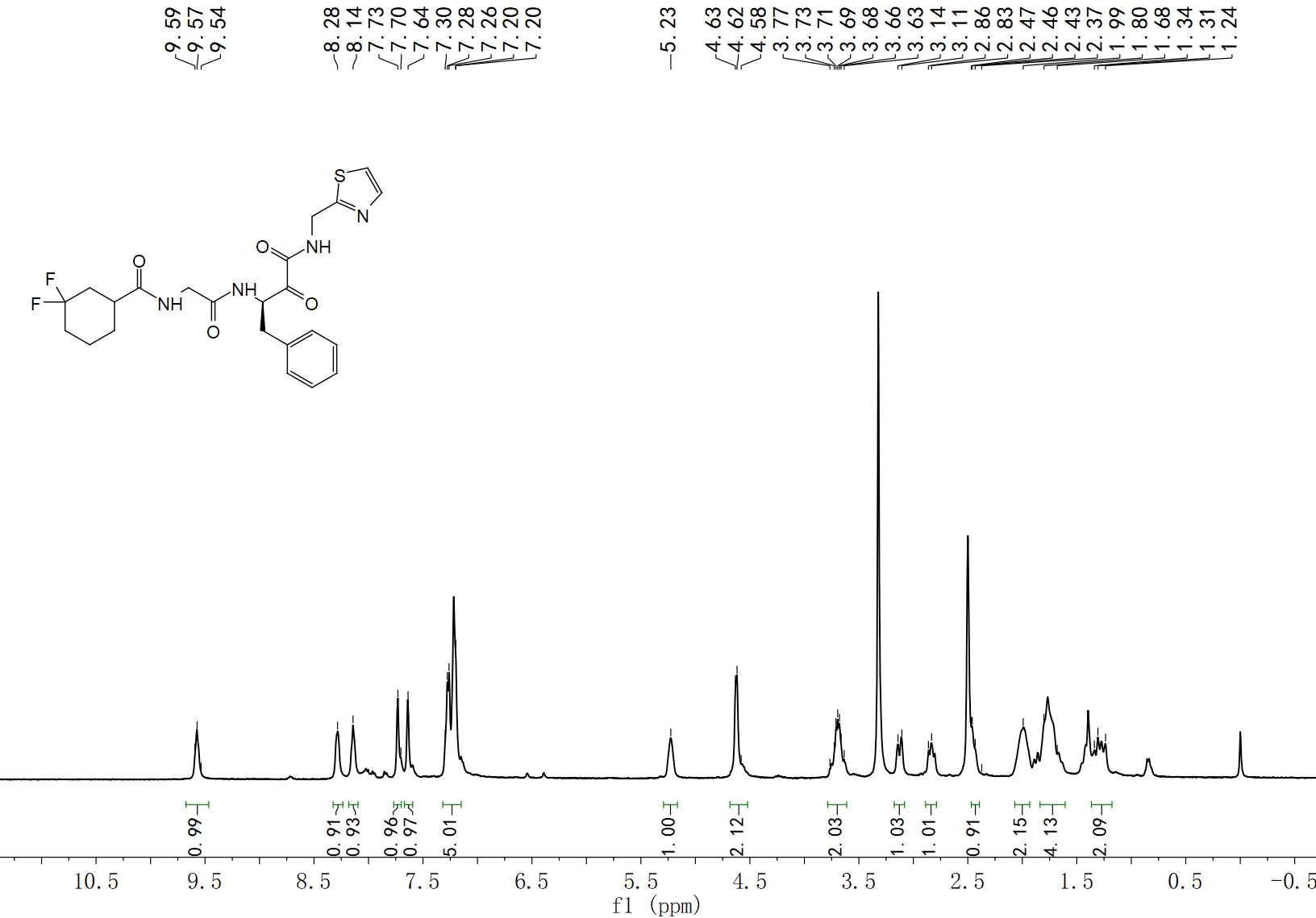


**^1^H NMR of 3o**


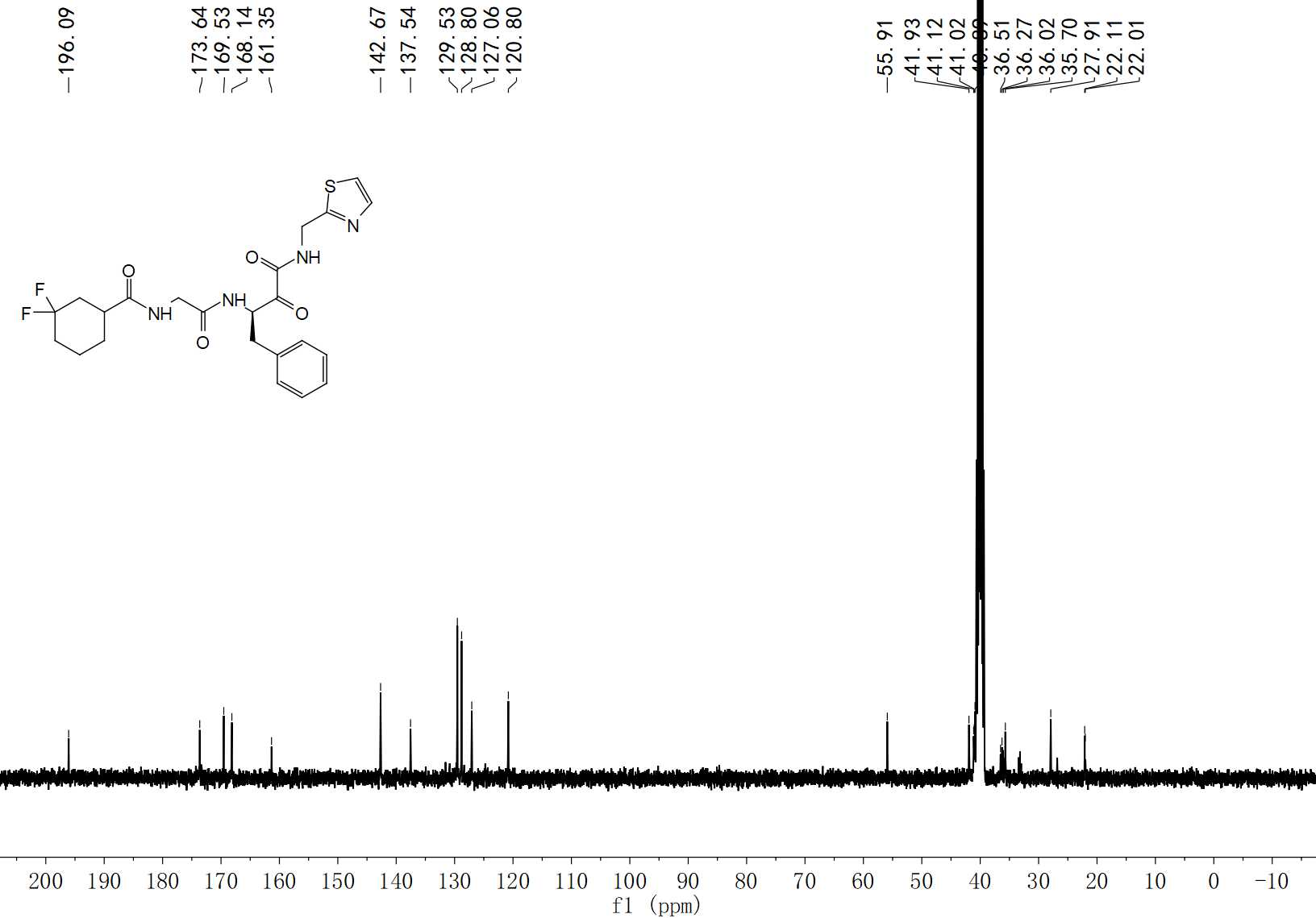


**^13^C NMR of 3o**


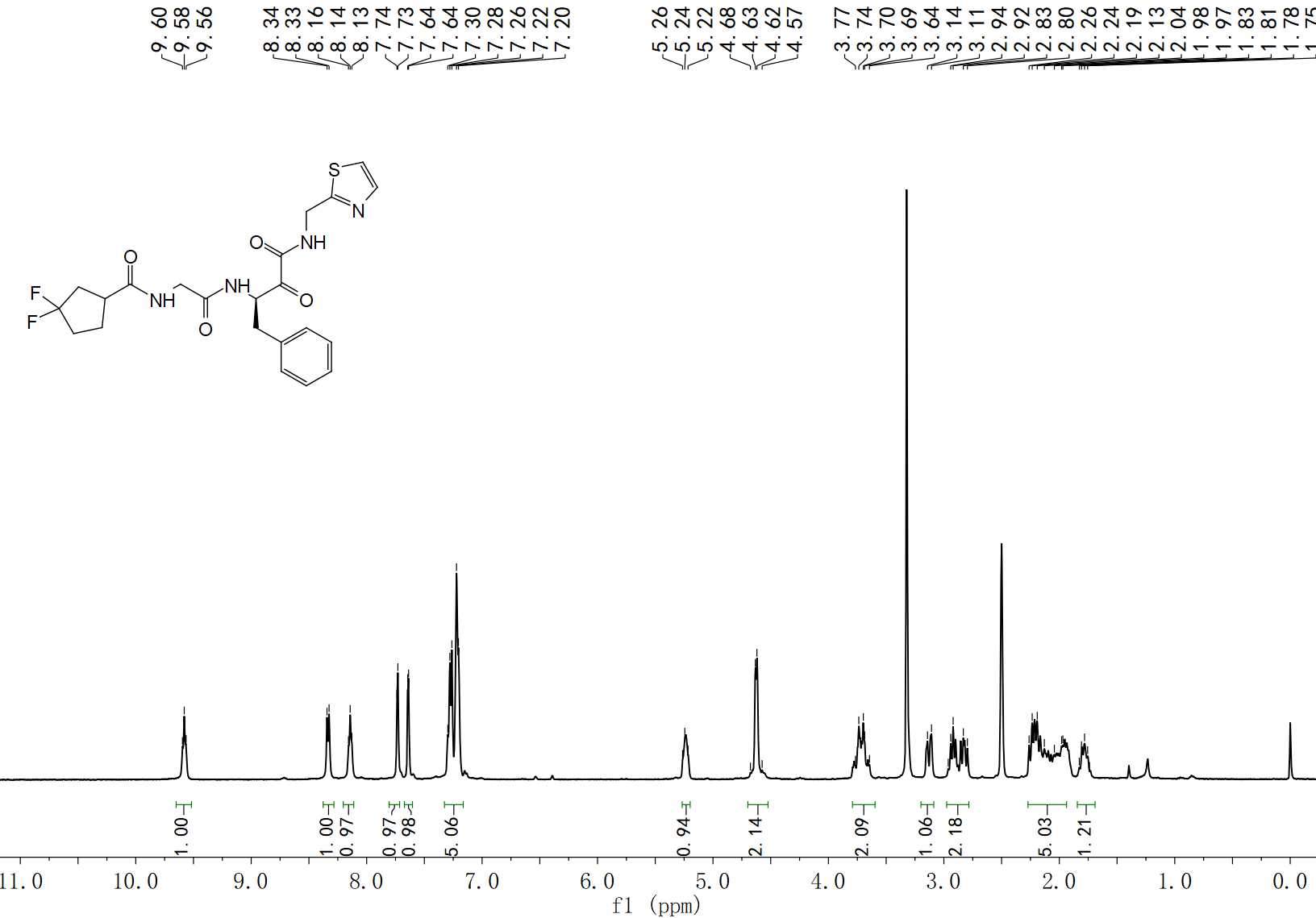


**^1^H NMR of 3p**


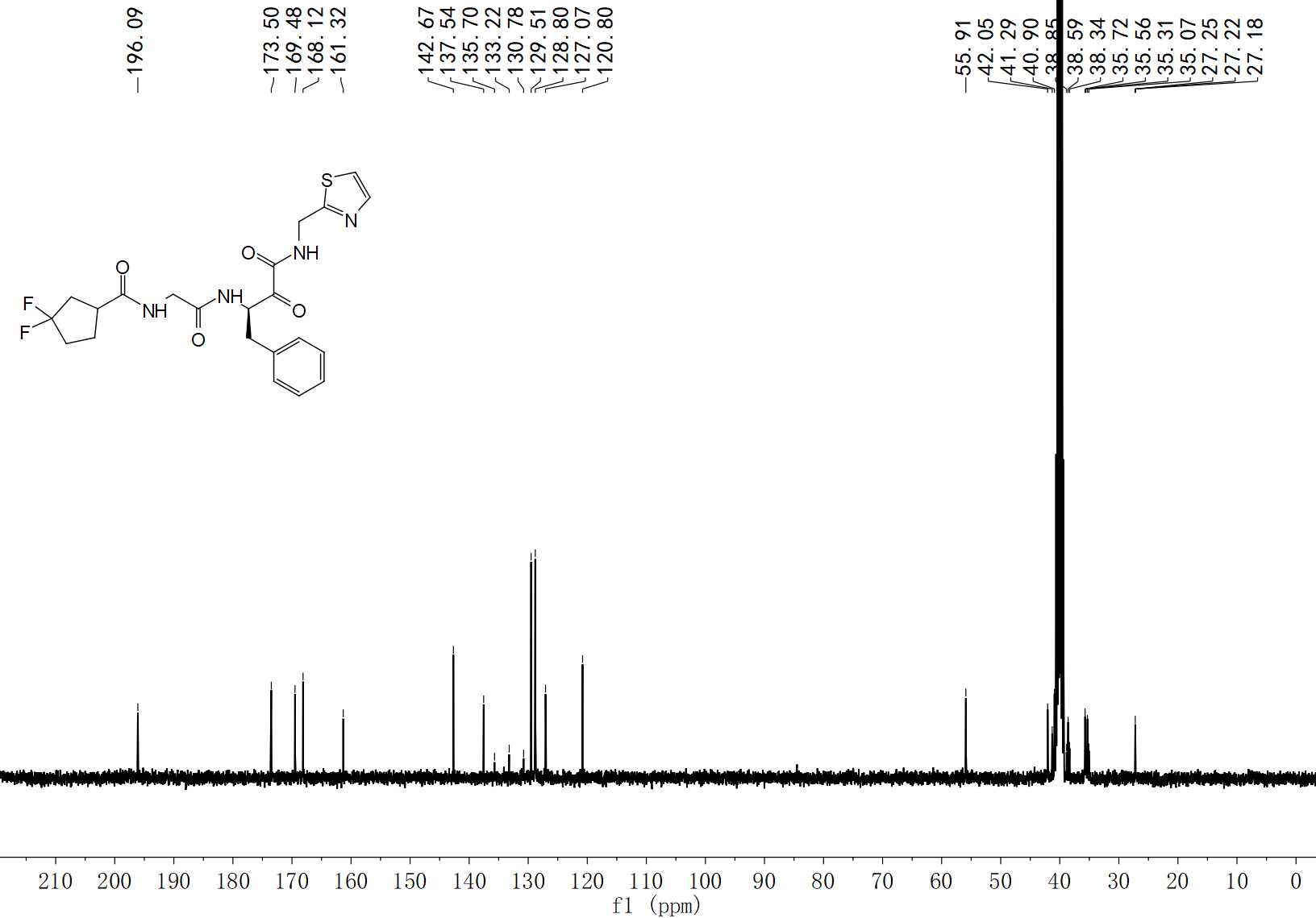


**^13^C NMR of 3p**


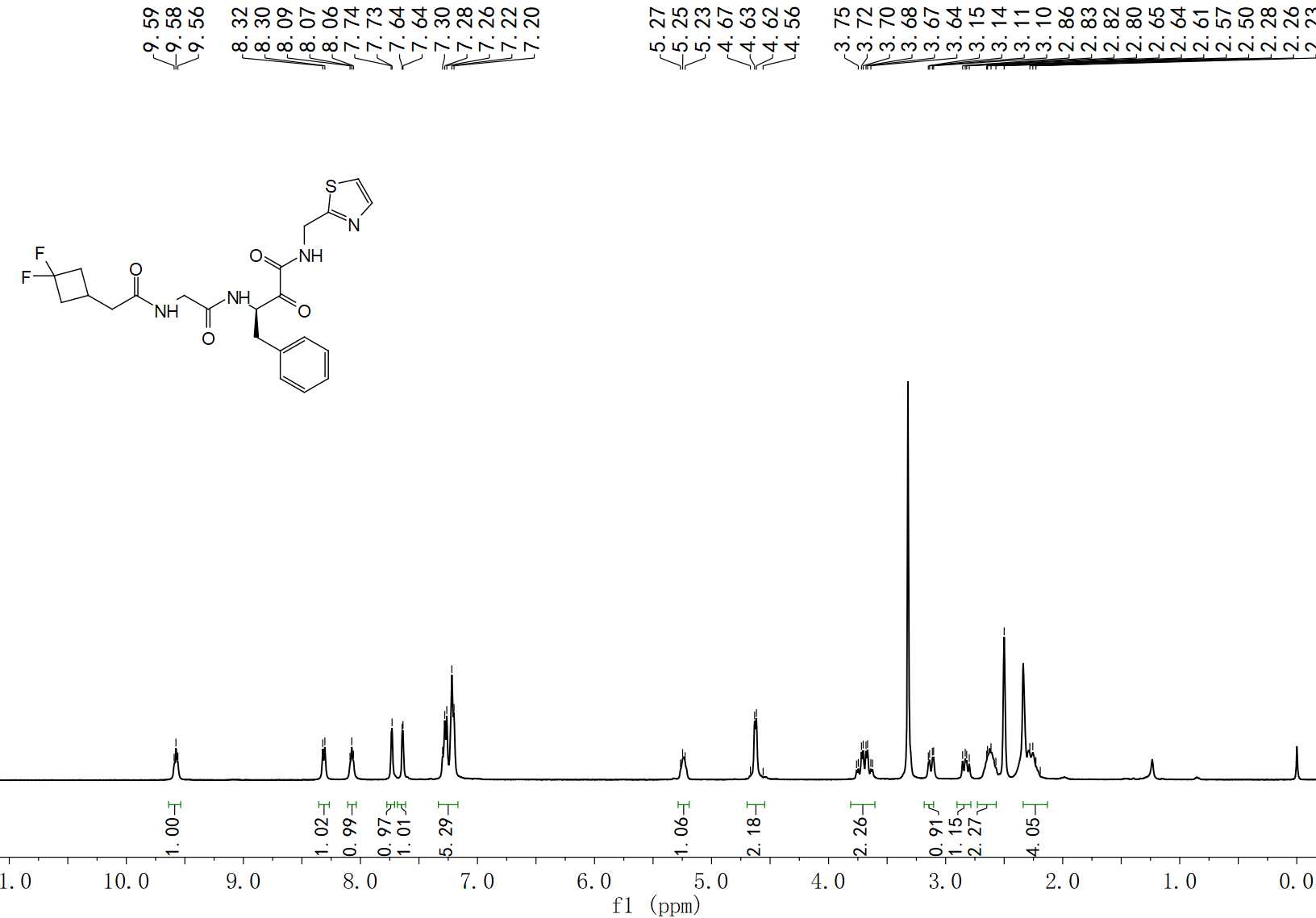


**^1^H NMR of 3q**


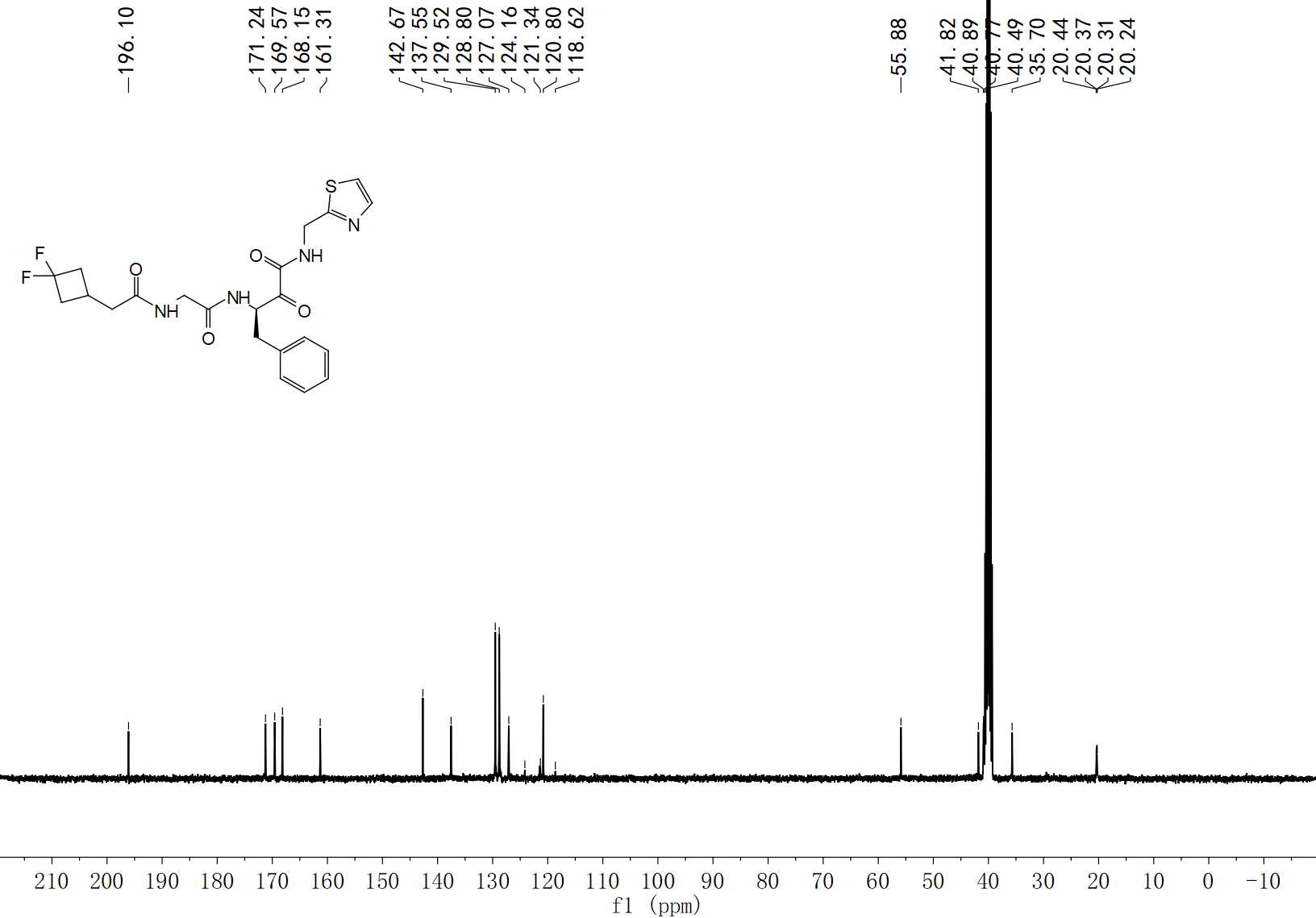


**^13^C NMR of 3q**


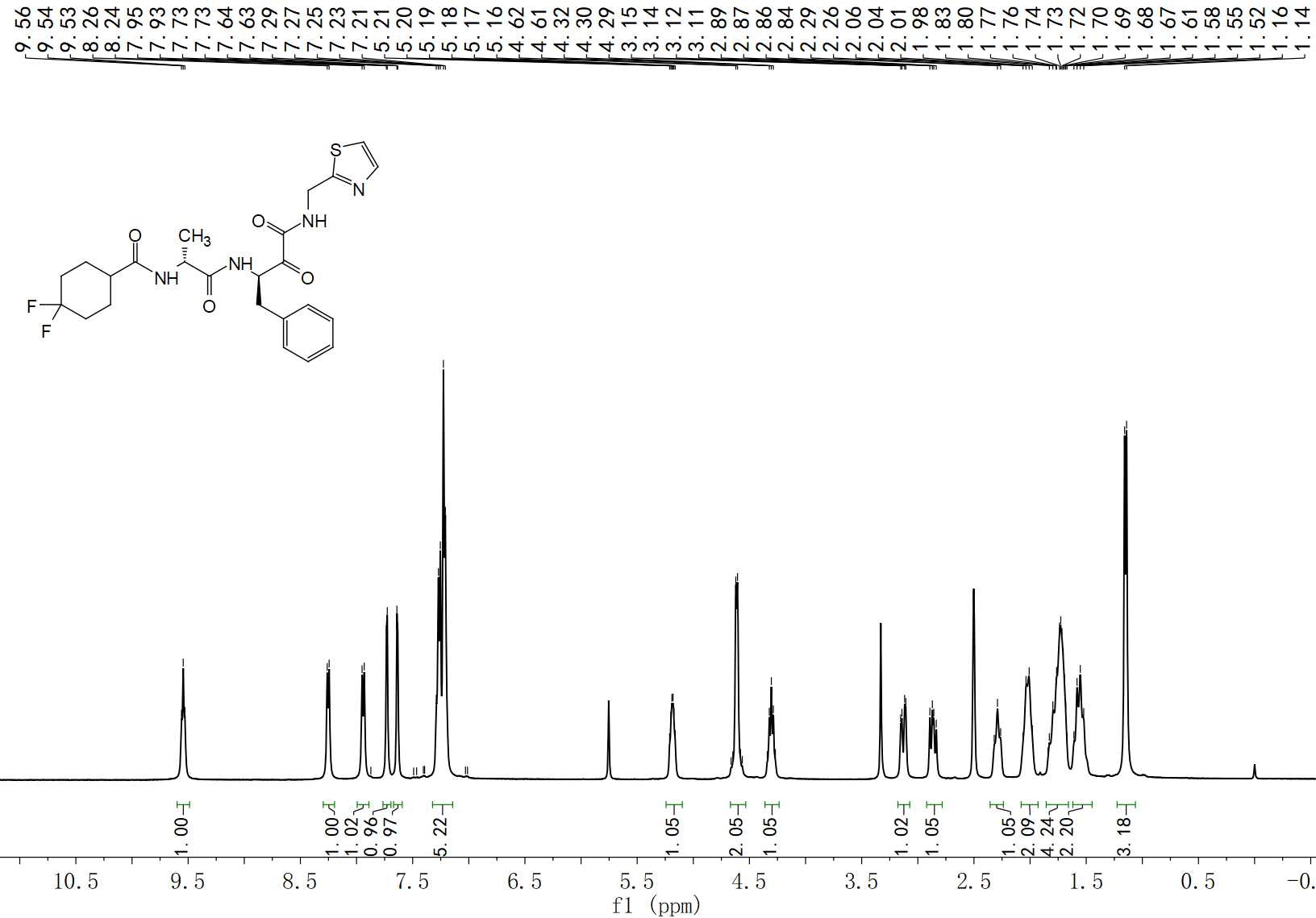


**^1^H NMR of 3r**


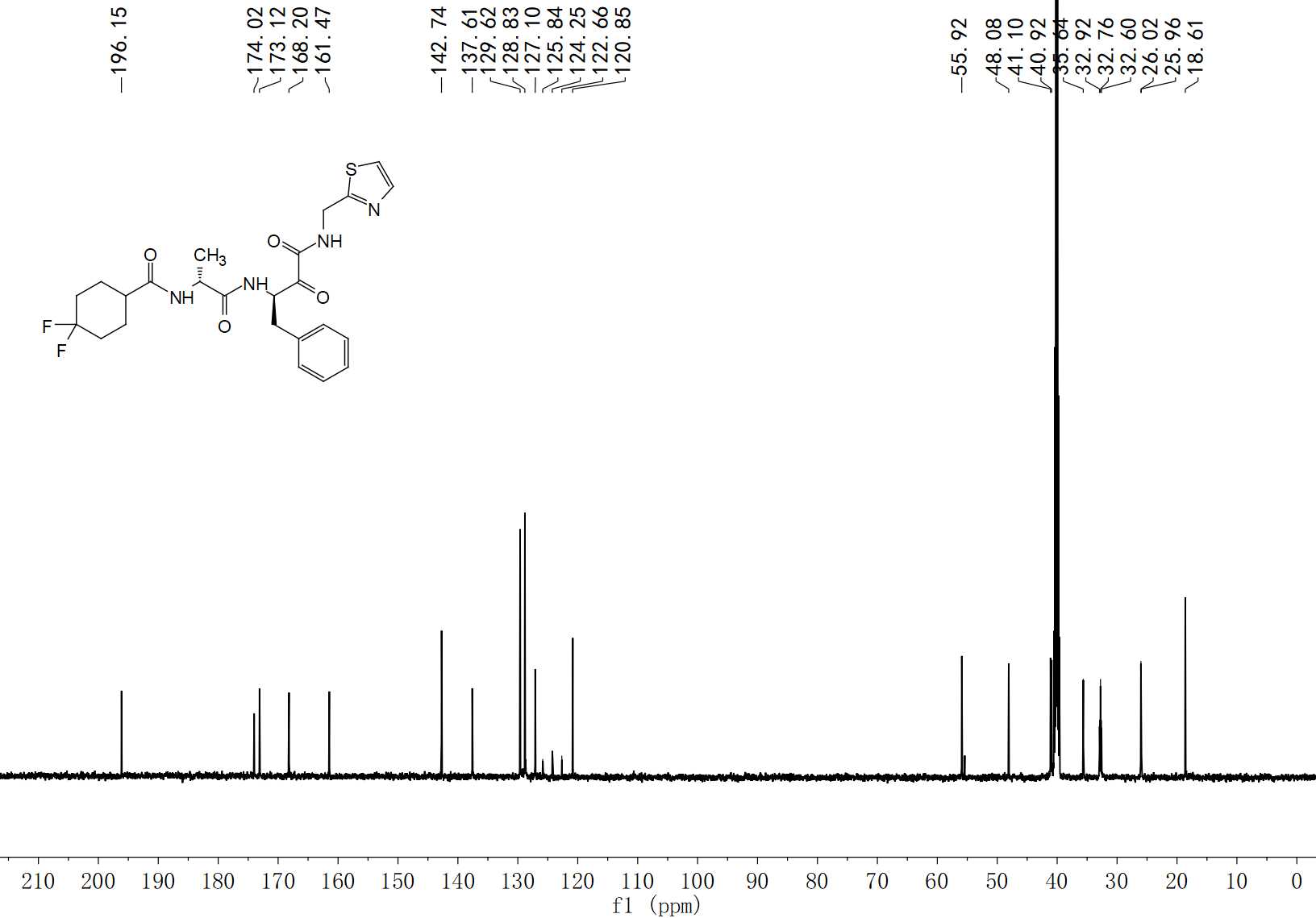


**^13^C NMR of 3r**


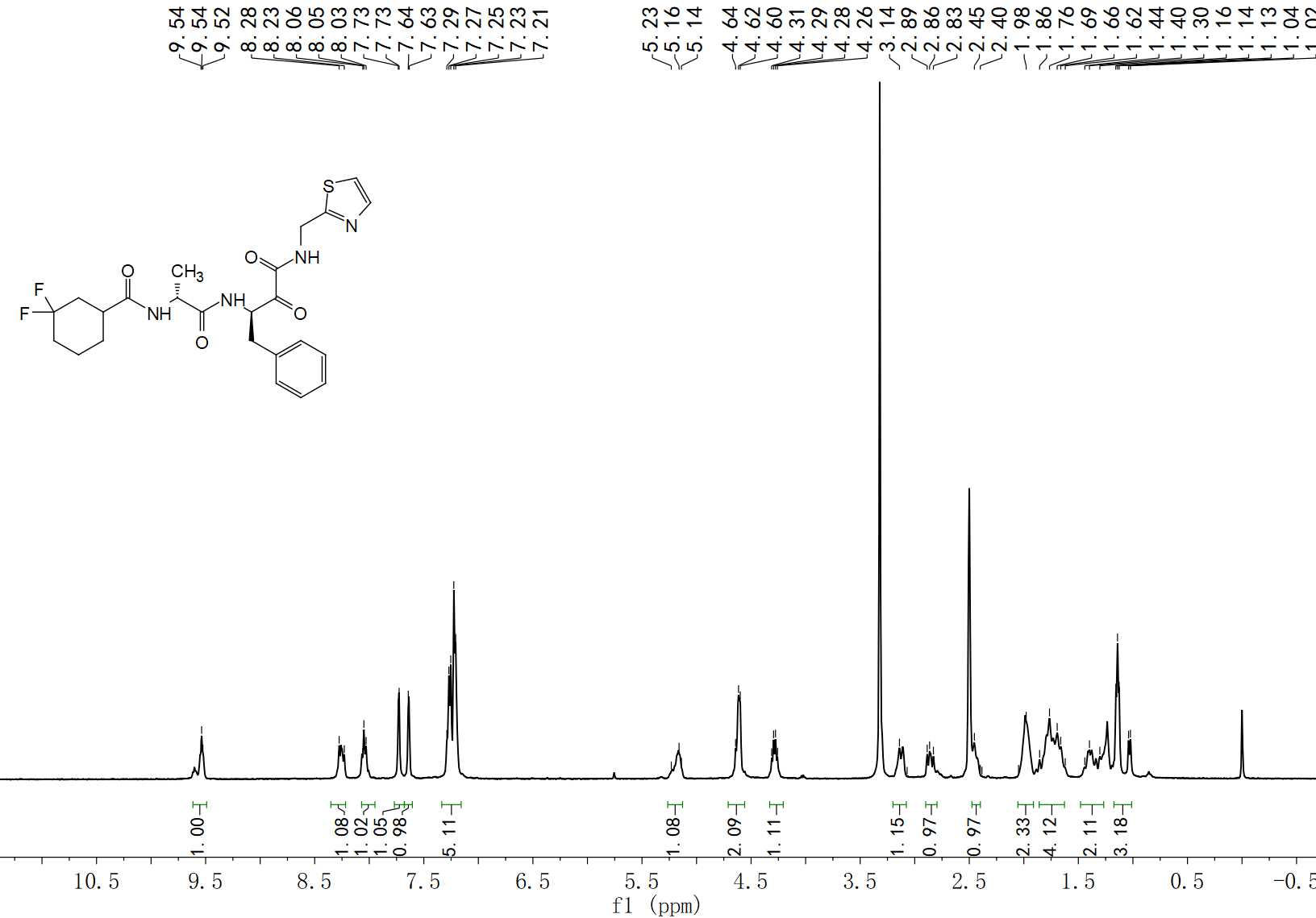


**^1^H NMR of 3s**


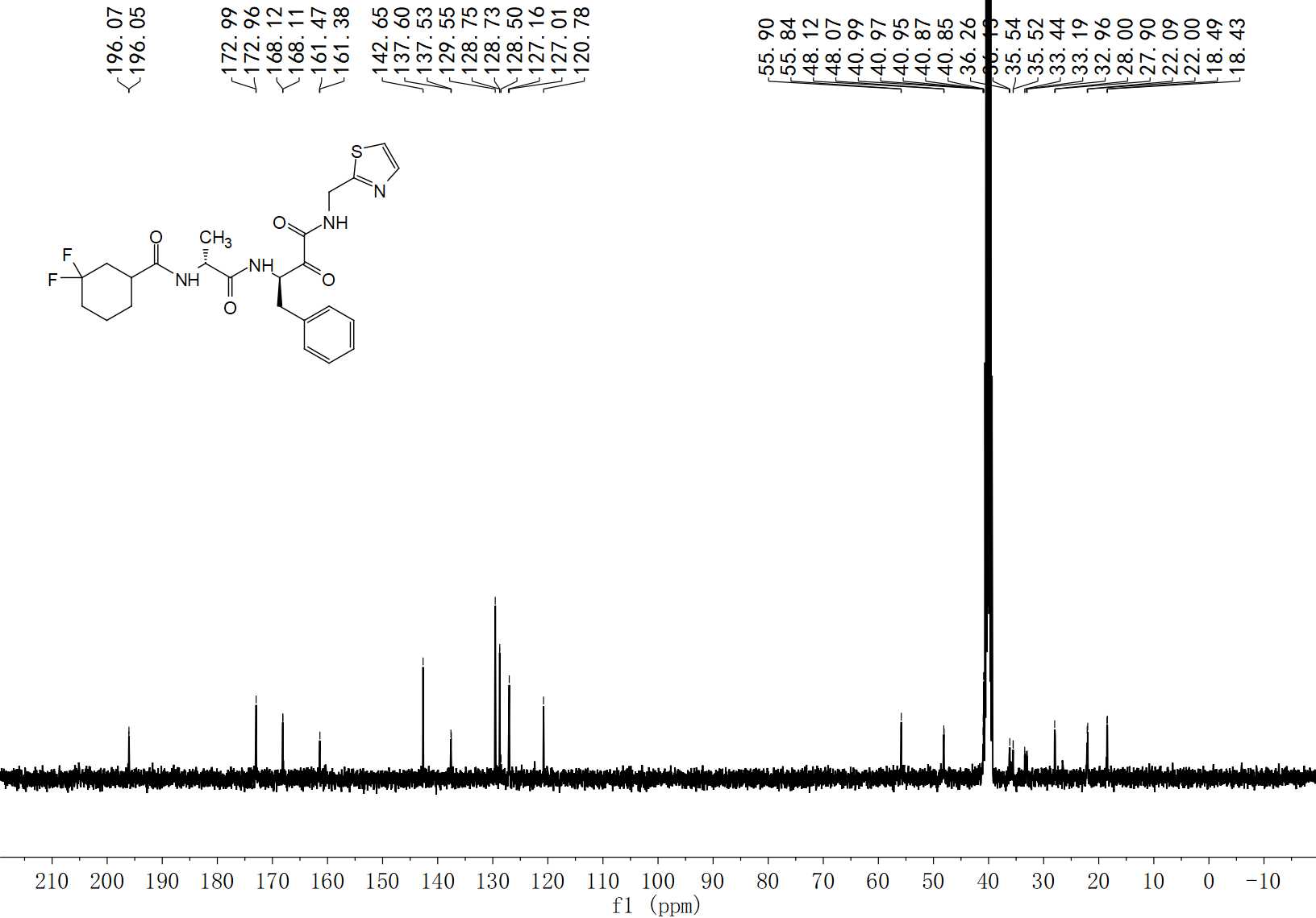


**^13^C NMR of 3s**


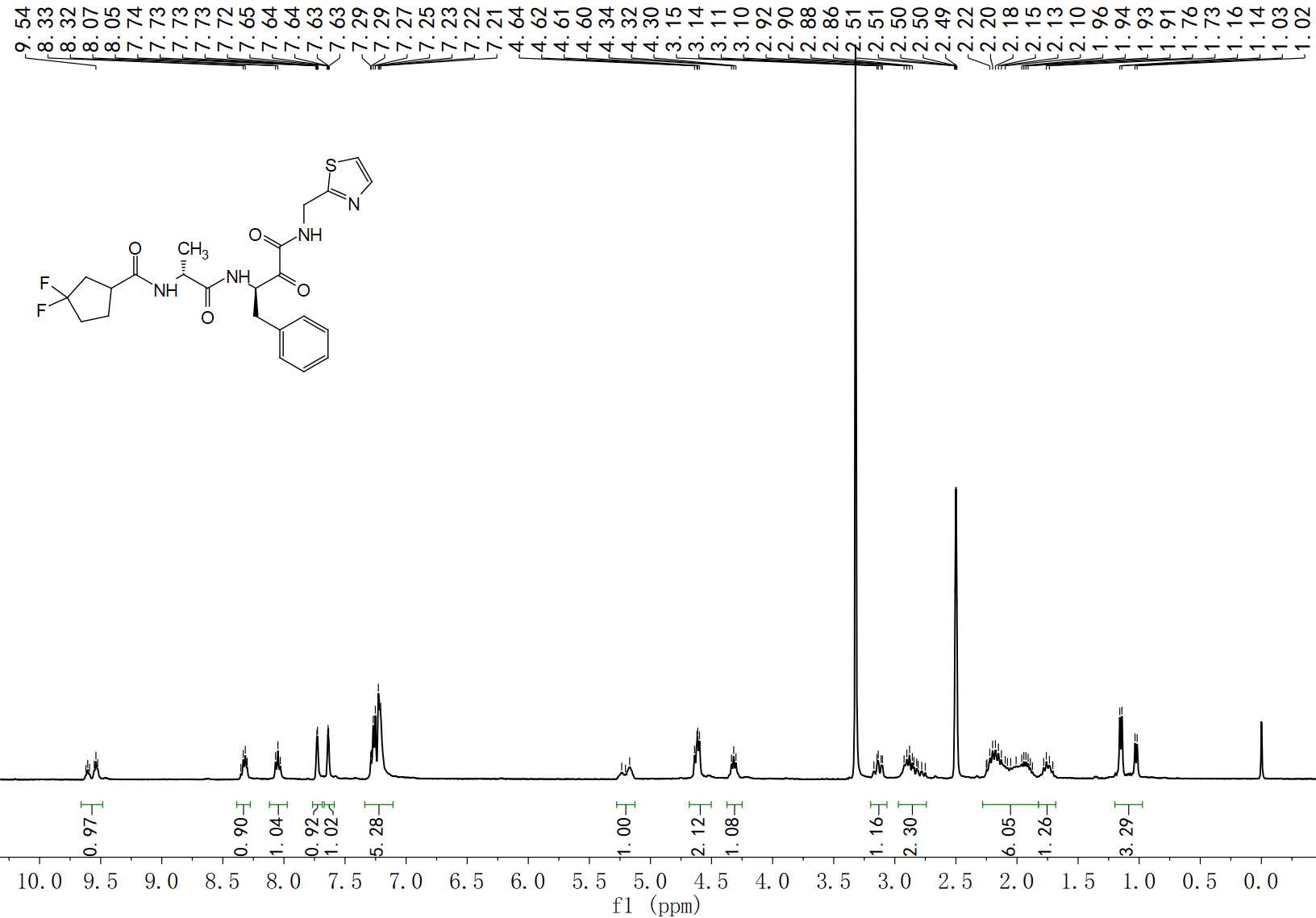


**^1^H NMR of 3t**


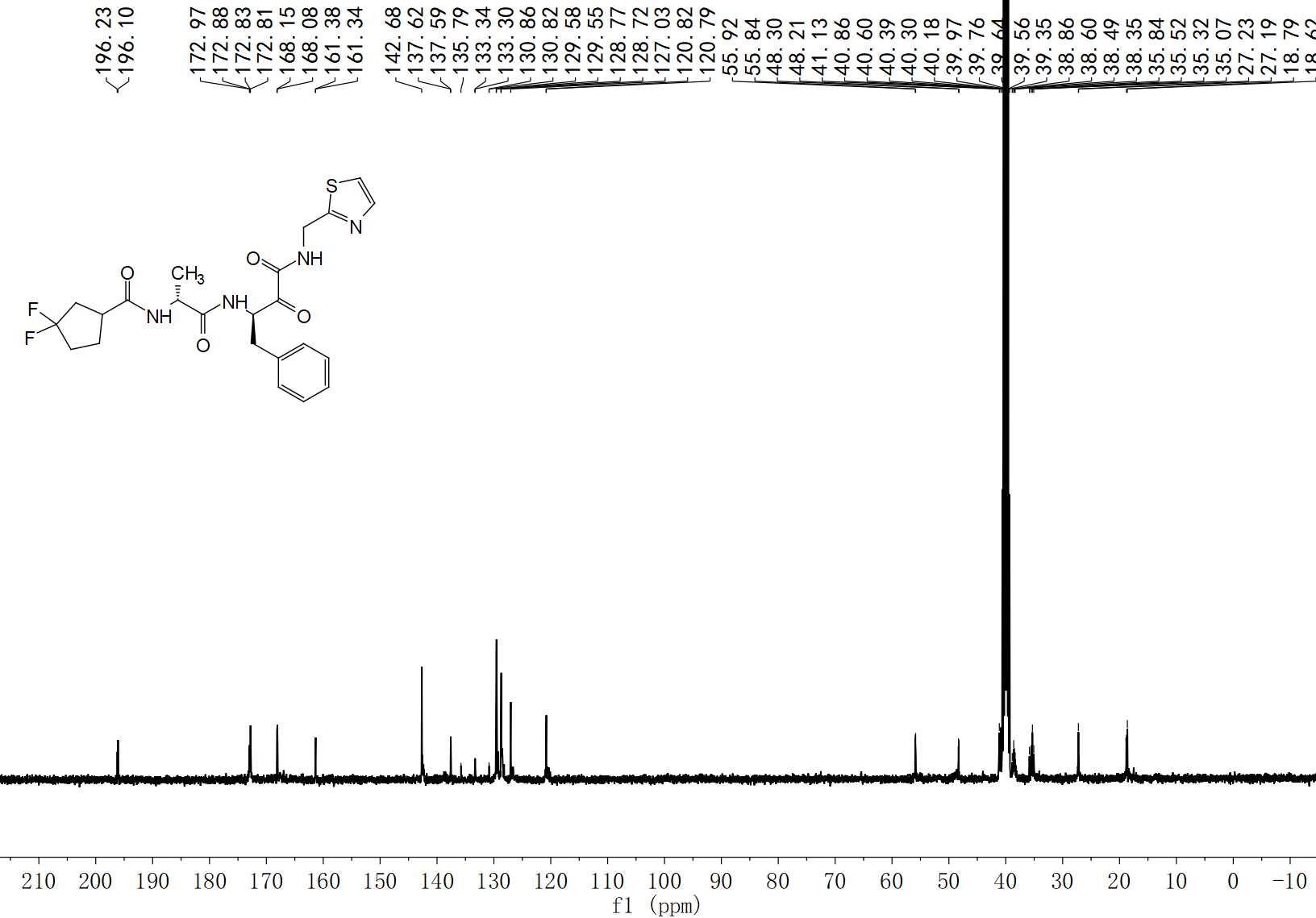


**^13^C NMR of 3t**


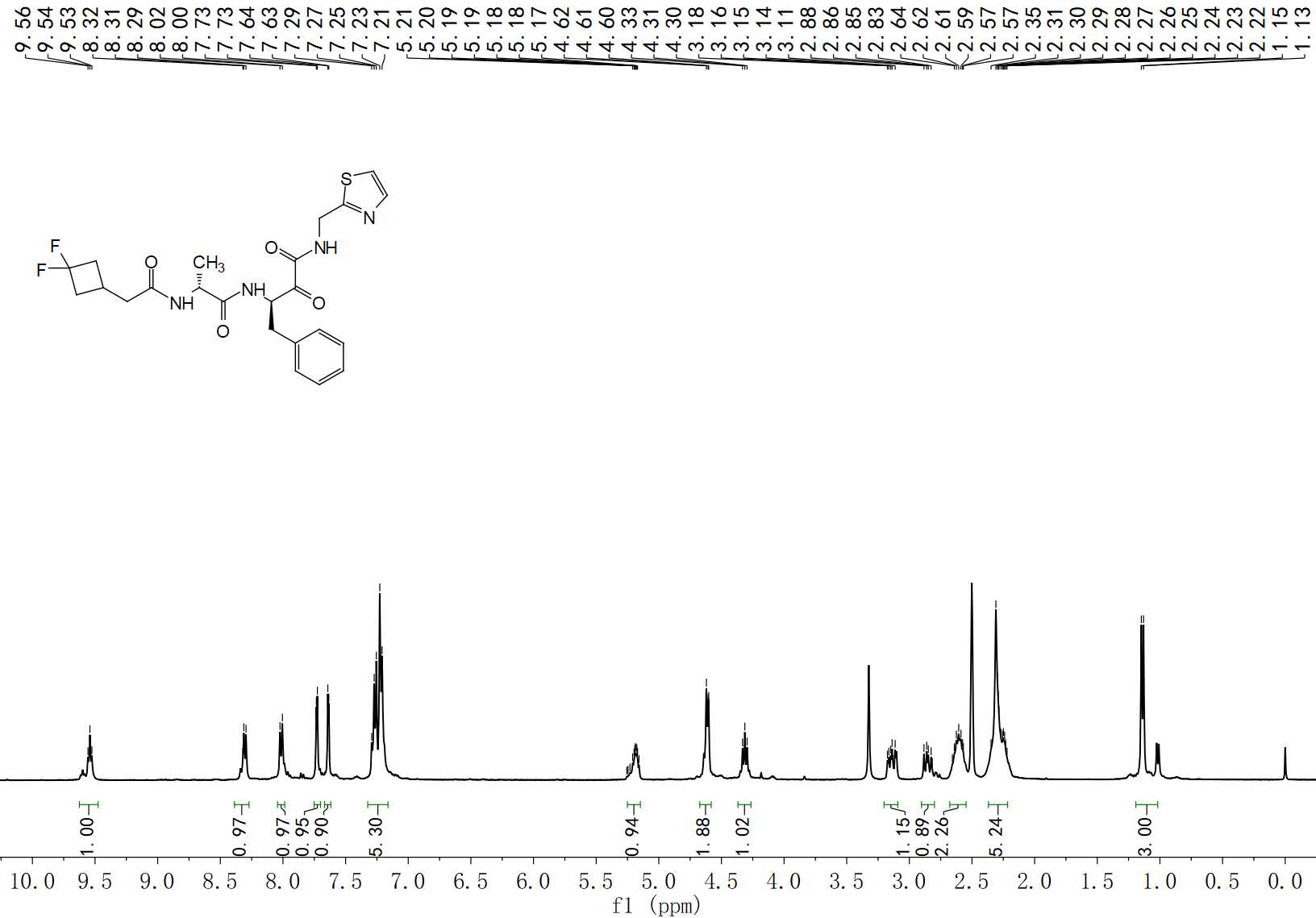


**^1^H NMR of 3u**


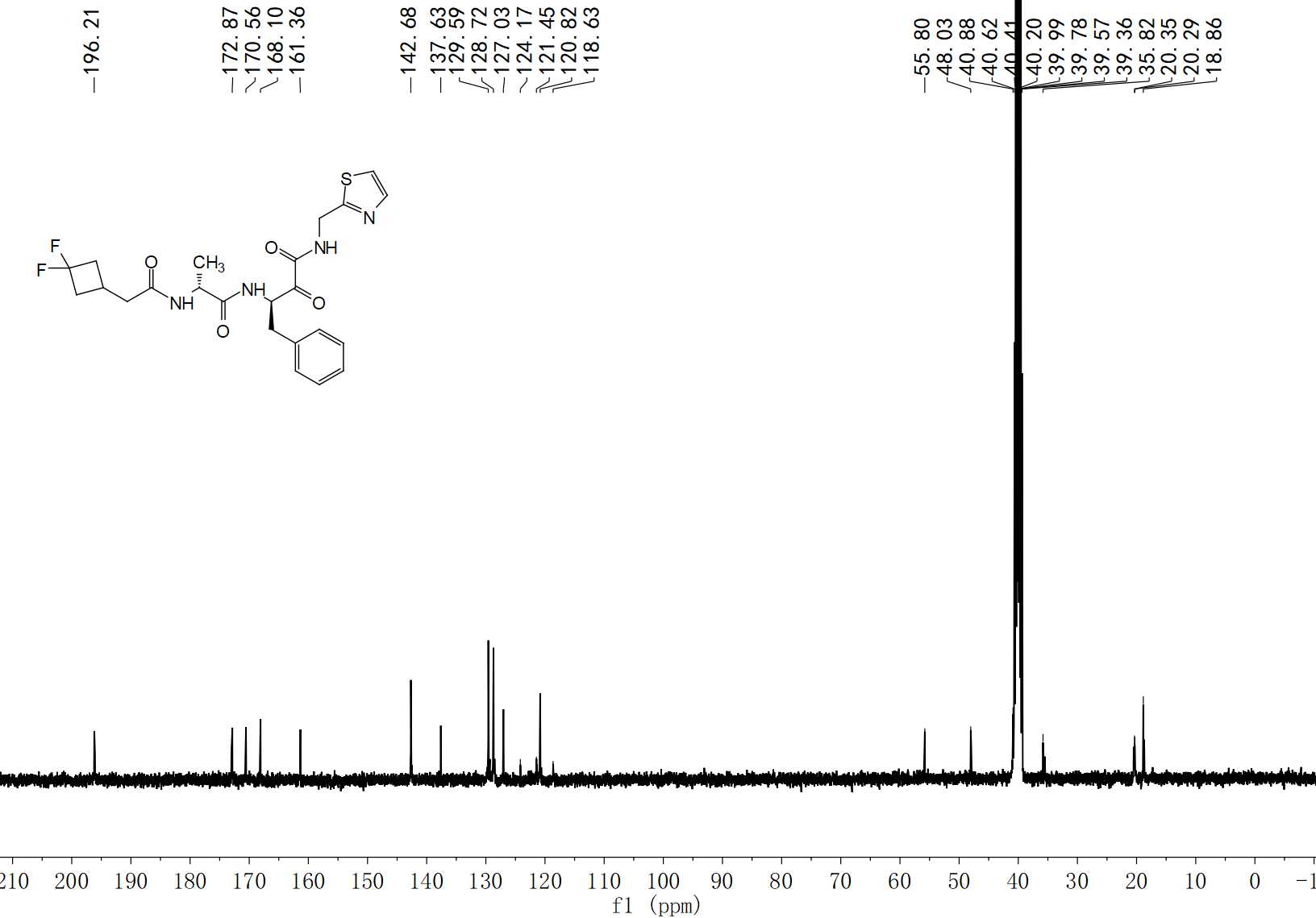


**^13^C NMR of 3u**


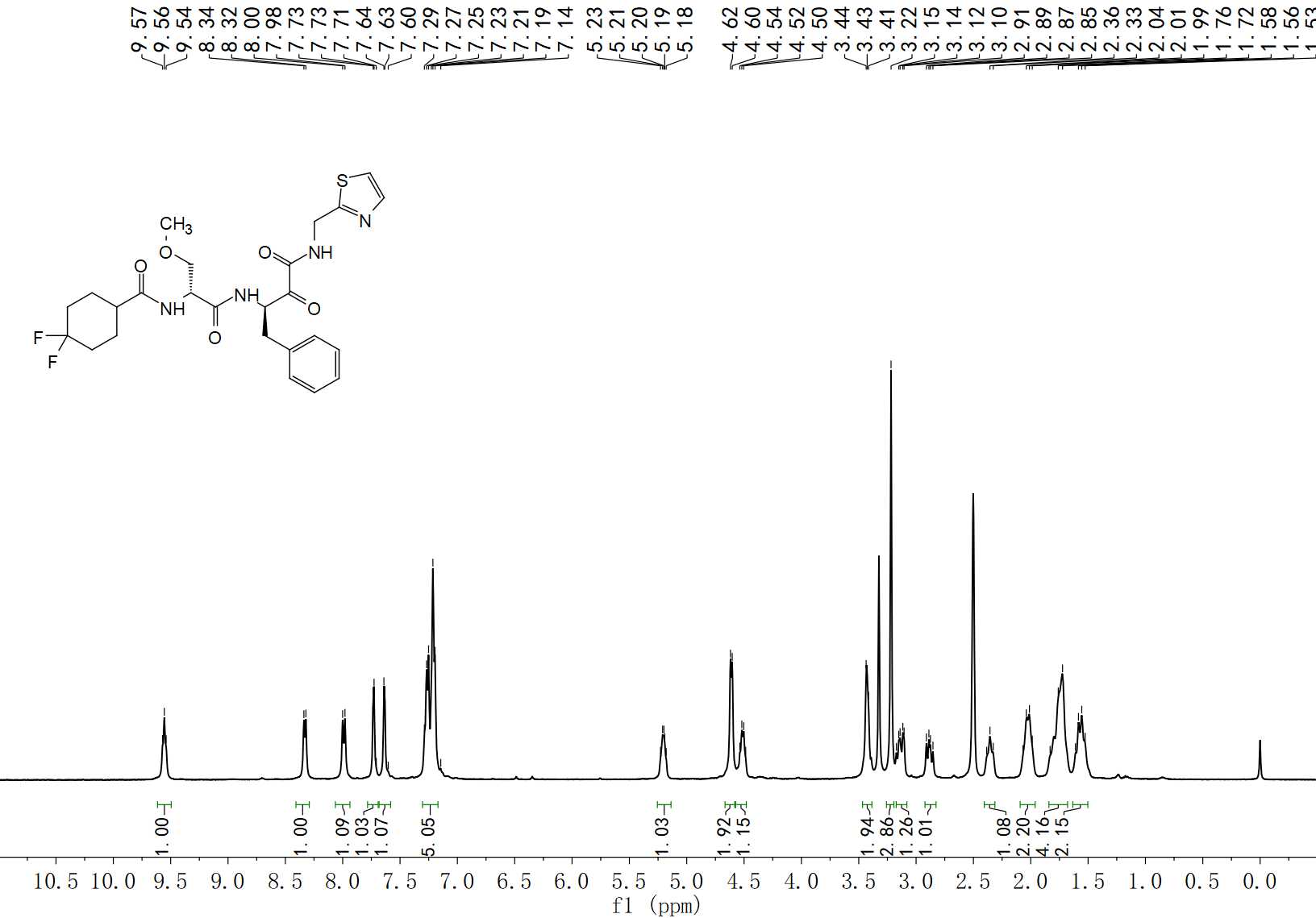


**^1^H NMR of 3v**


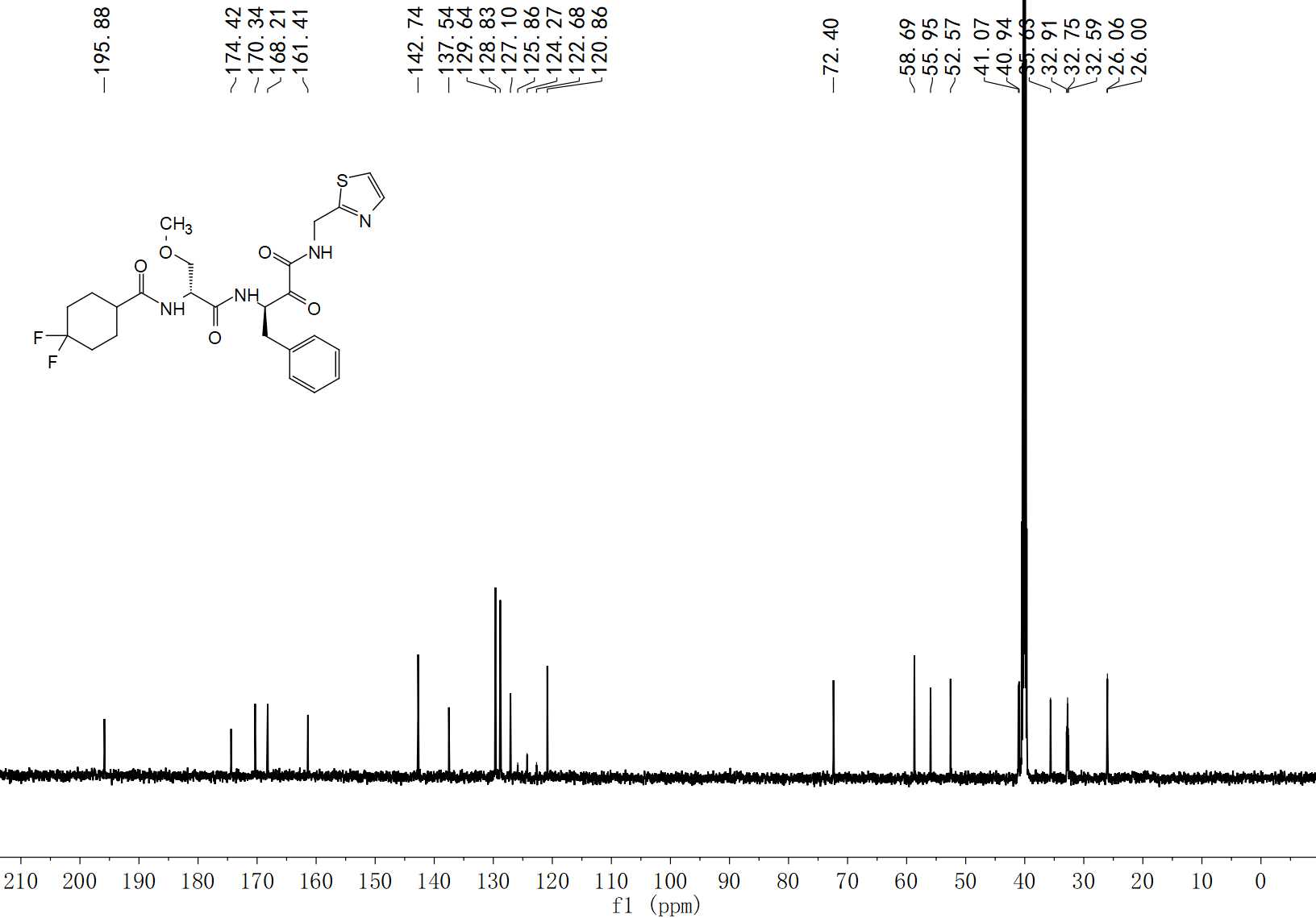


**^13^C NMR of 3v**


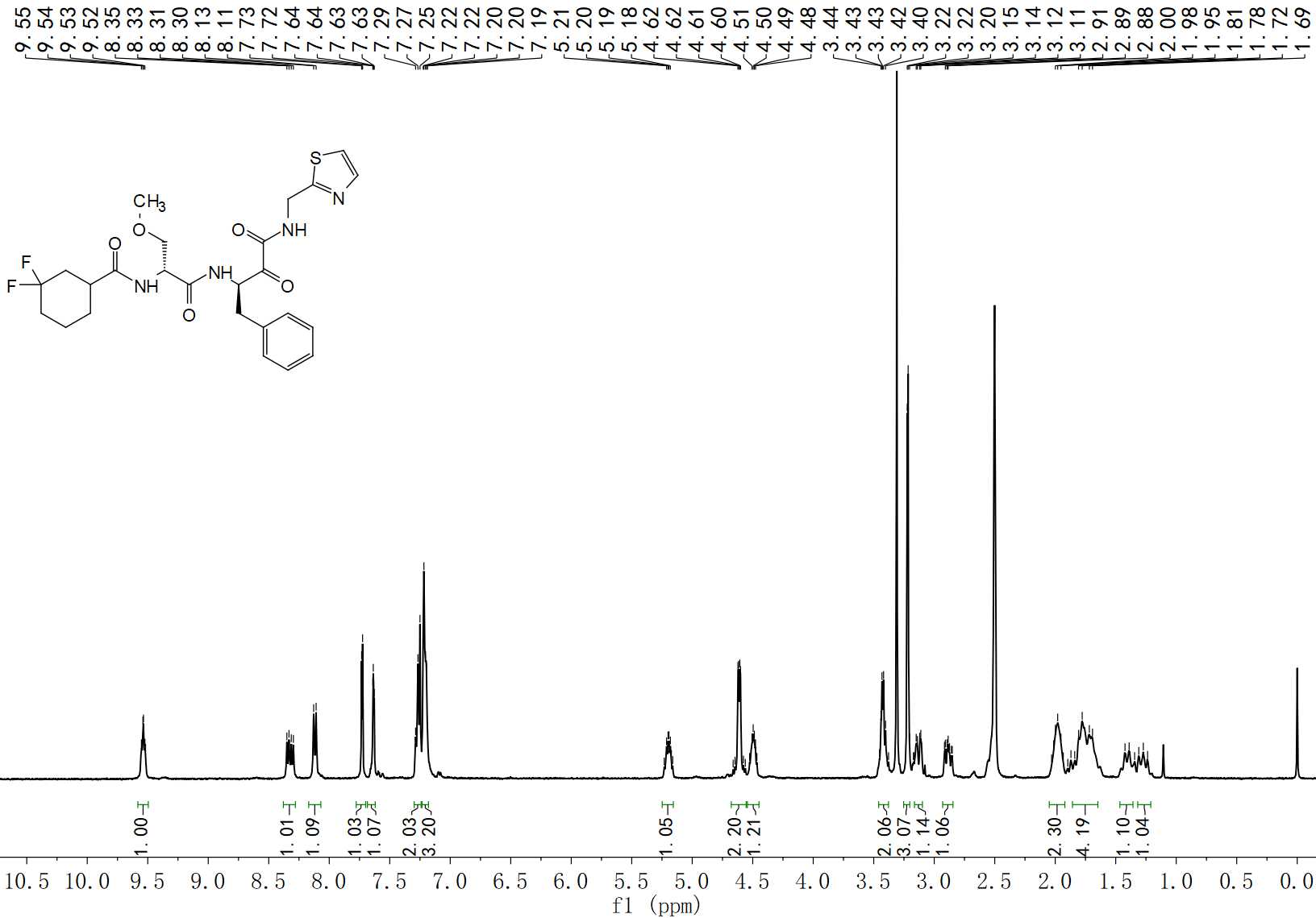


**^1^H NMR of 3w**


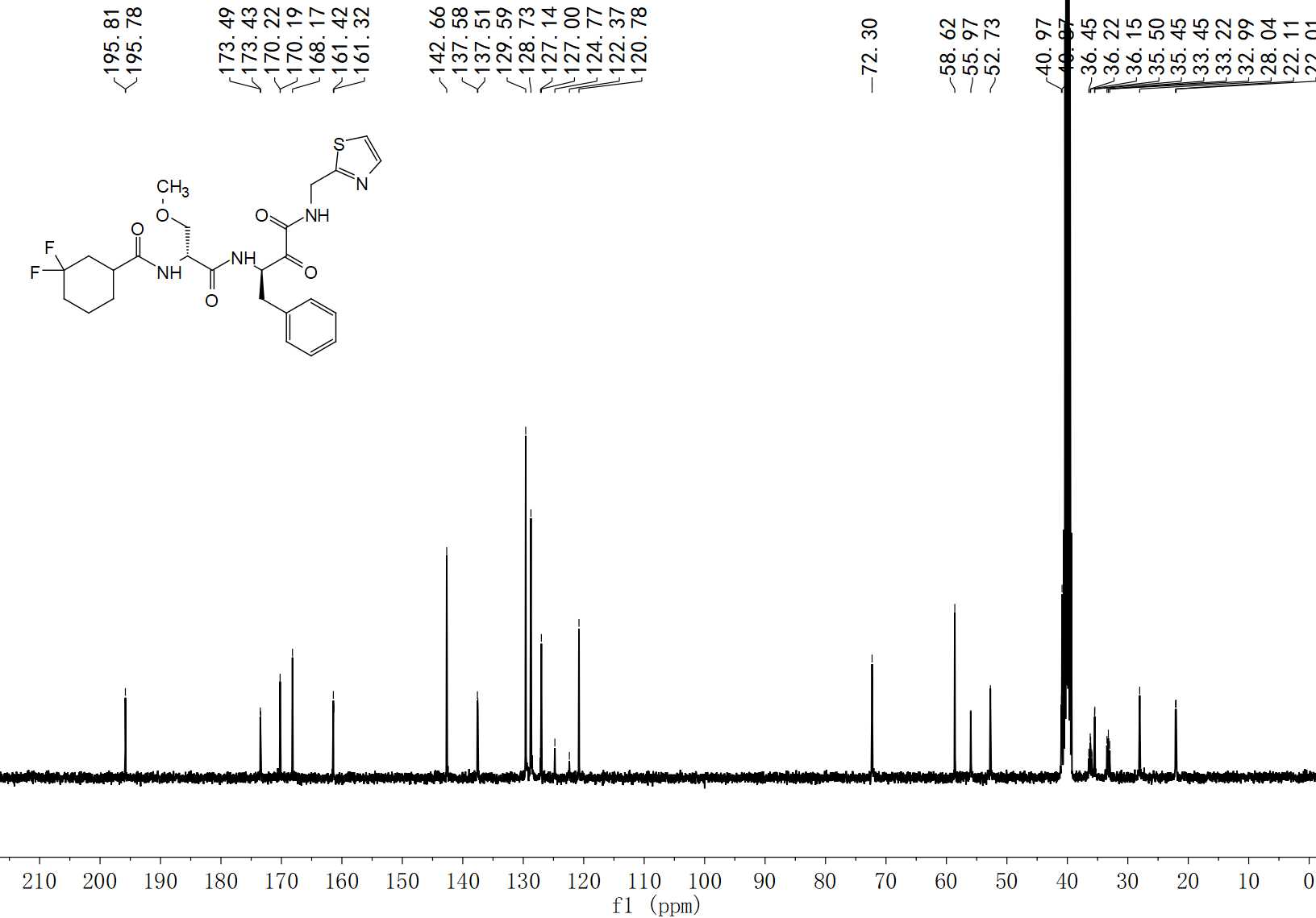


**^13^C NMR of 3w**


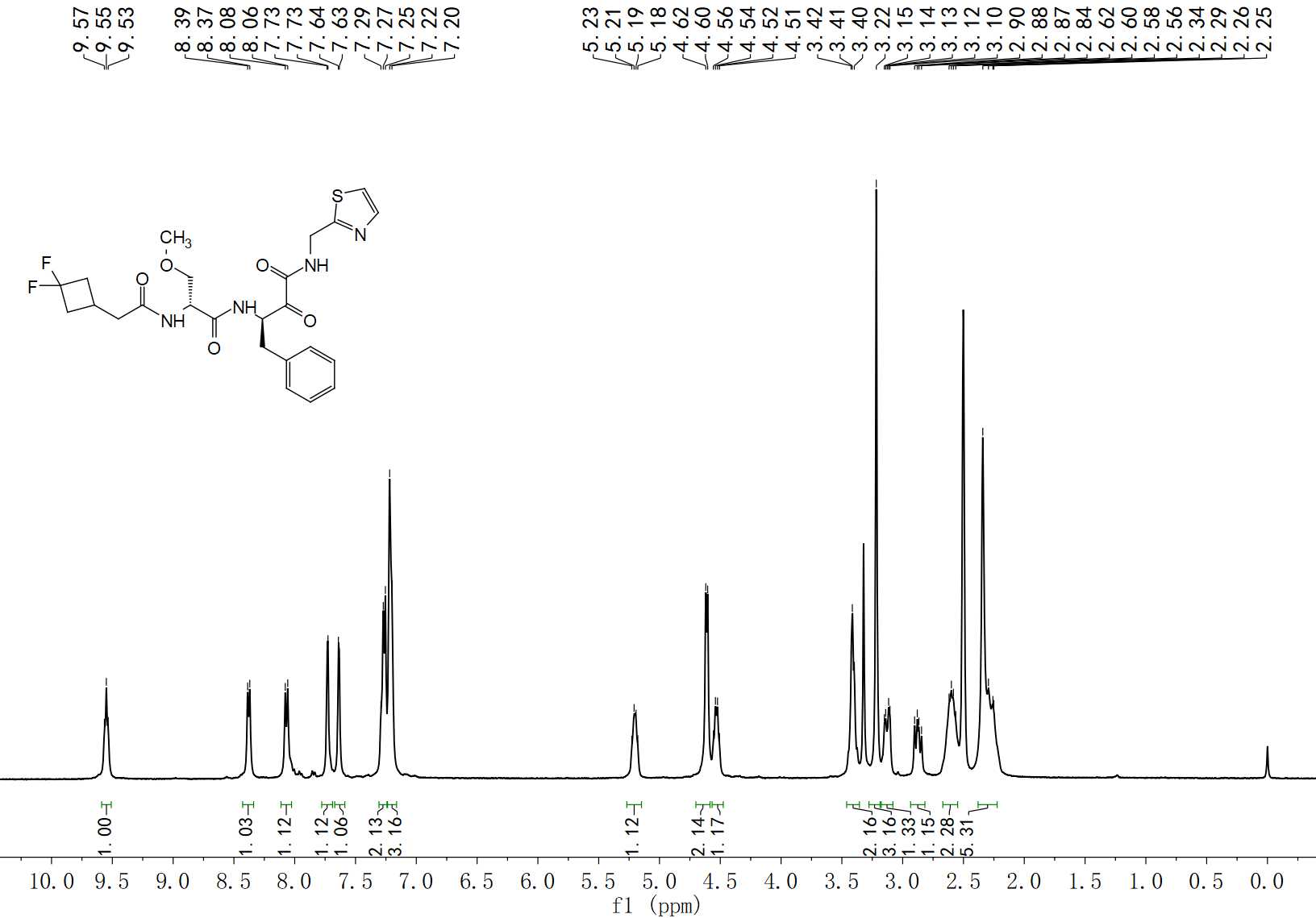


**^1^H NMR of 3x**


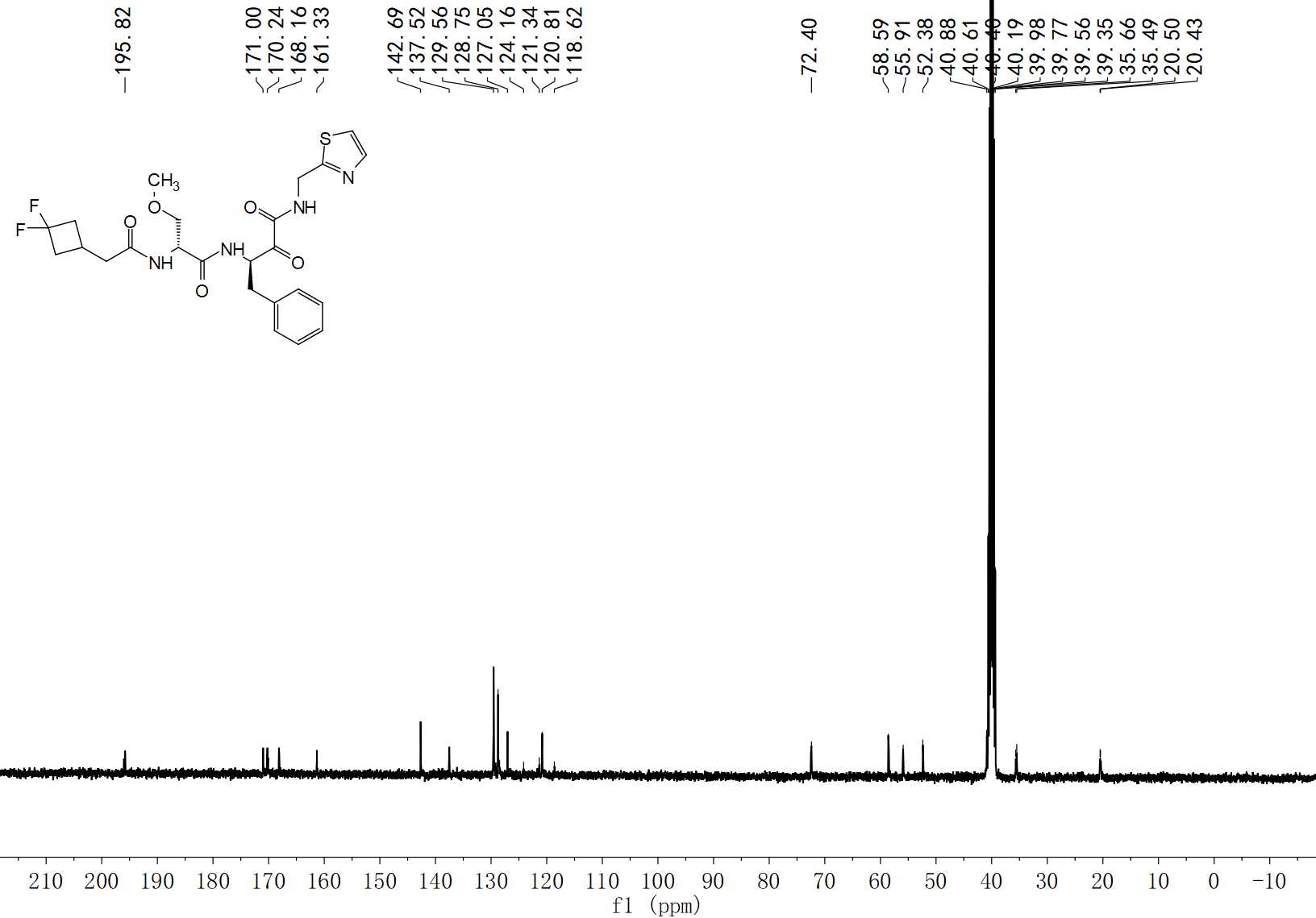


**^13^C NMR of 3x**


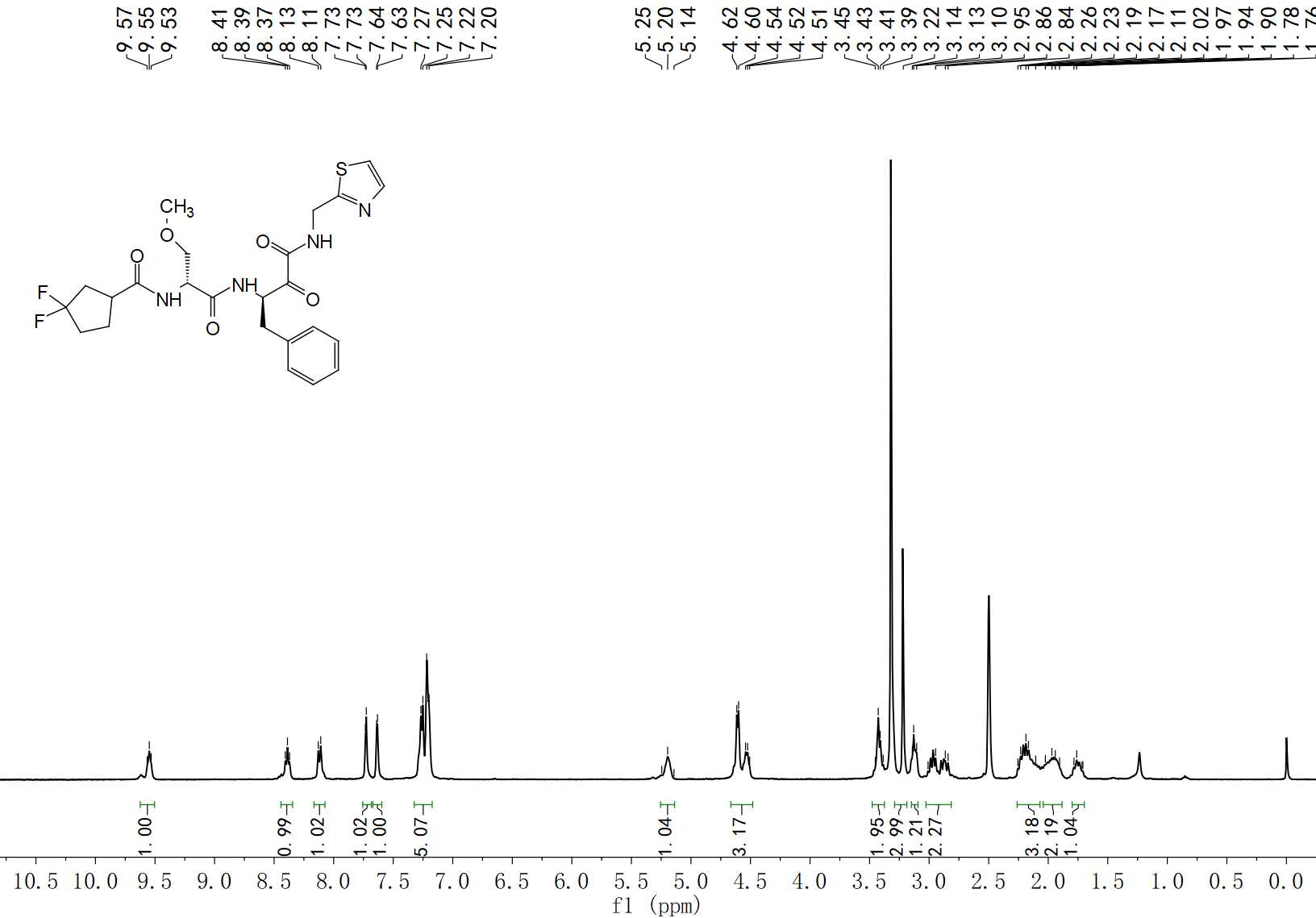


**^1^H NMR of 3y**


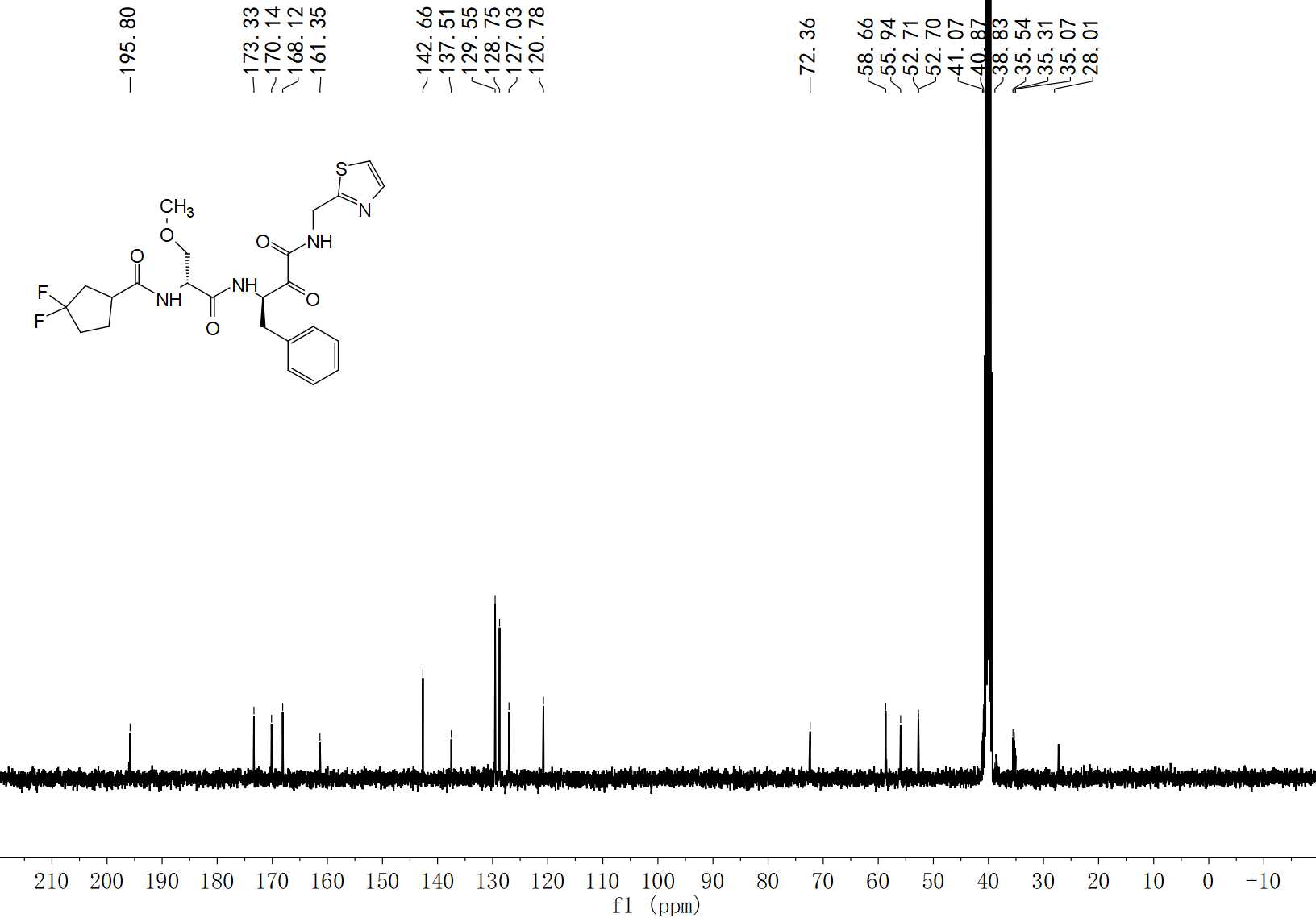


**^13^C NMR of 3y**

**HRMS data of products**

**HRMS of 1a**

**HRMS of 1b**

**HRMS of 1c**

**HRMS of 1d**

**HRMS of 1e**

**HRMS of 1f**

**HRMS of 1g**

**HRMS of 1h**

**HRMS of 1i**

**HRMS of 1j**

**HRMS of 2a**

**HRMS of 2b**

**HRMS of 2c**

**HRMS of 2d**

**HRMS of 2e**

**HRMS of 2f**

**HRMS of 2g**

**HRMS of 3a**

**HRMS of 3b**

**HRMS of 3c**

**HRMS of 3d**

**HRMS of 3e**

**HRMS of 3f**

**HRMS of 3g**

**HRMS of 3h**

**HRMS of 3i**

**HRMS of 3j**

**HRMS of 3k**

**HRMS of 3l**

**HRMS of 3m**

**HRMS of 3n**

**HRMS of 3o**

**HRMS of 3p**

**HRMS of 3q**

**
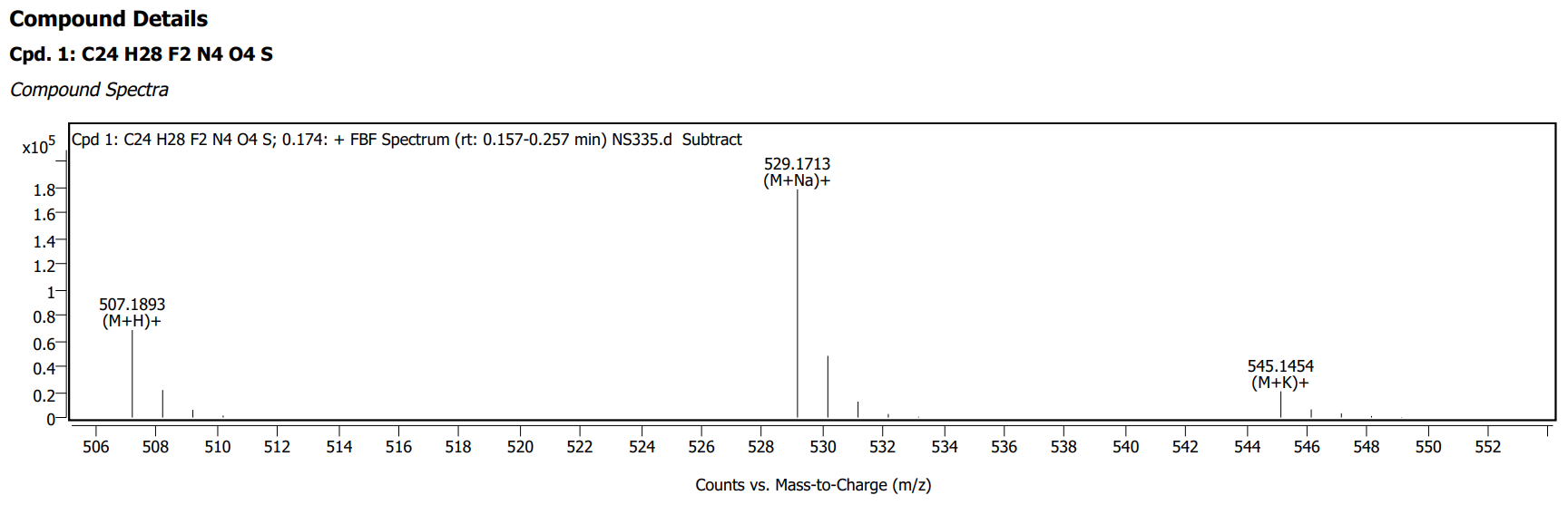
**

**HRMS of 3r**

**HRMS of 3s**

**
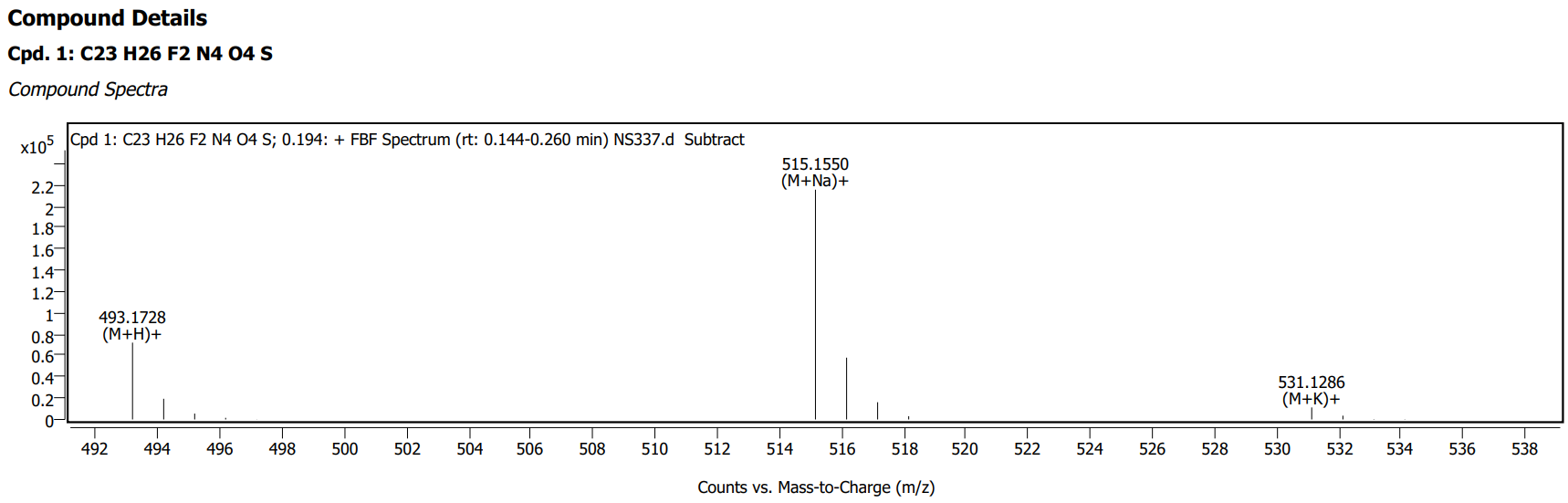
**

**HRMS of 3t**

**
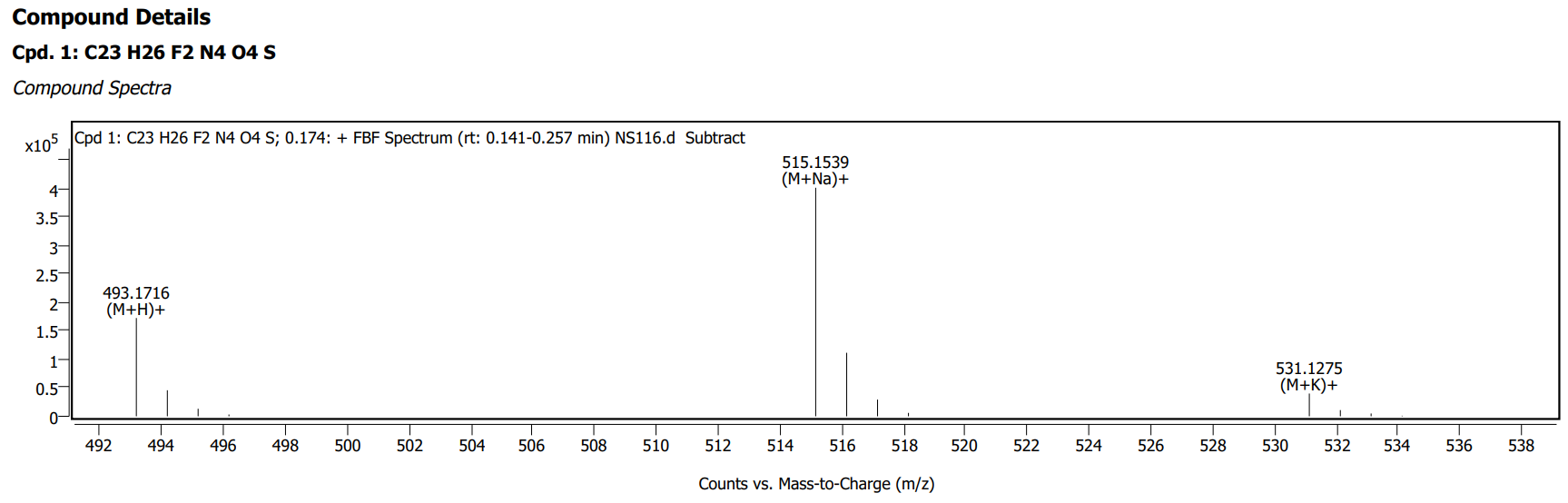
**

**HRMS of 3u**


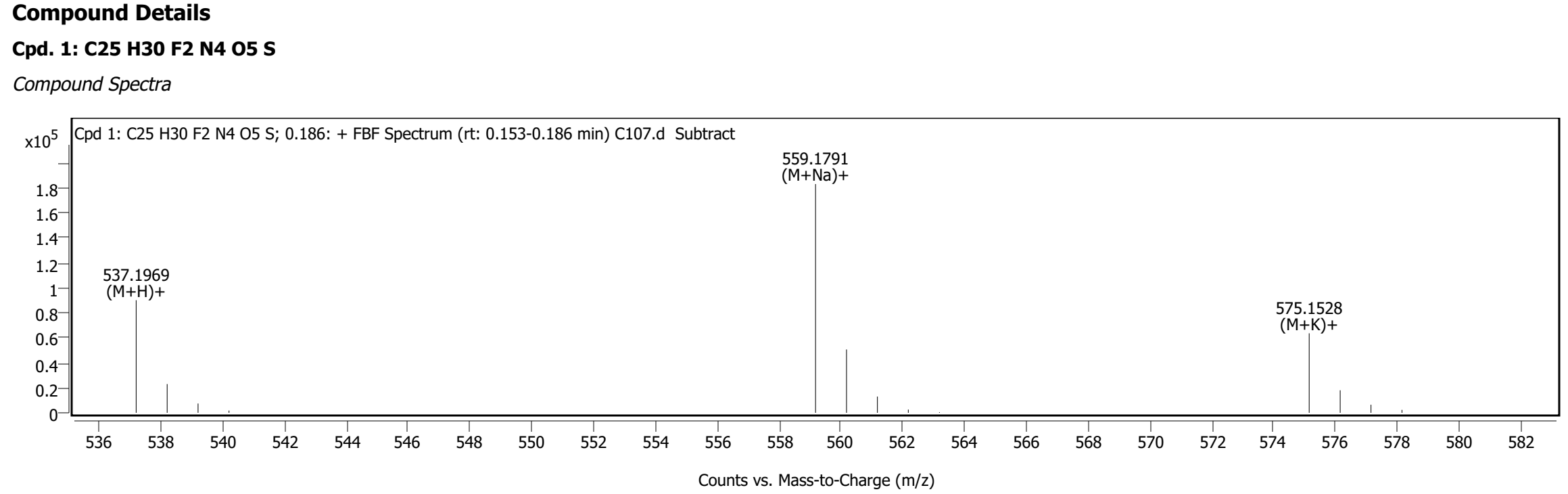


**HRMS of 3v**


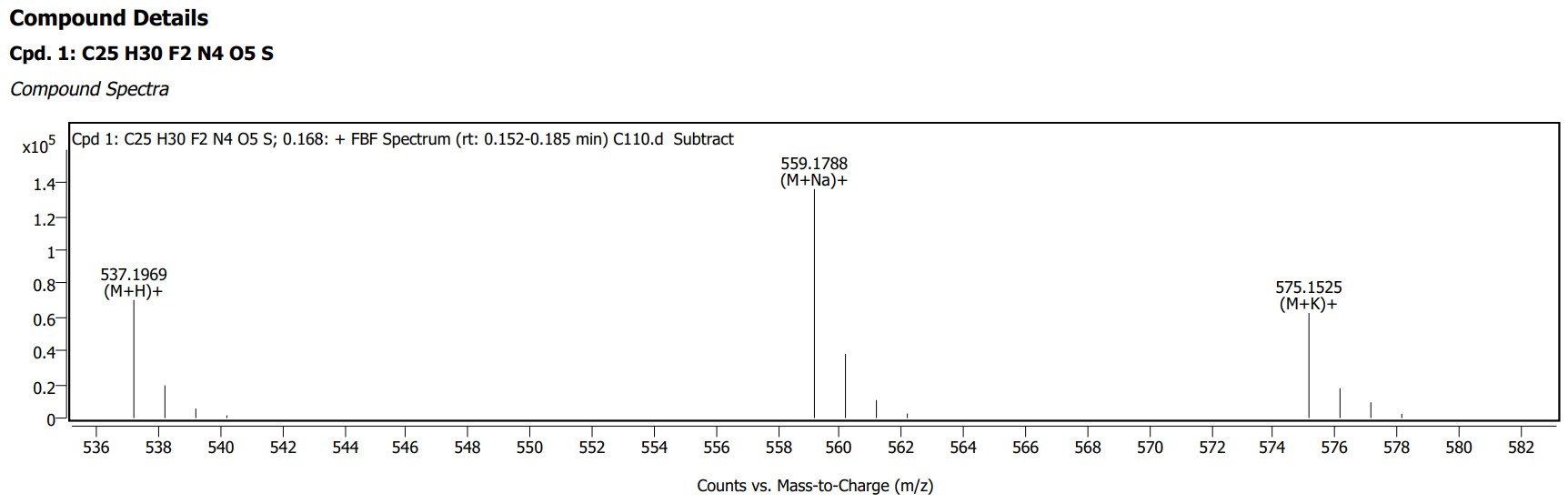


**HRMS of 3w**


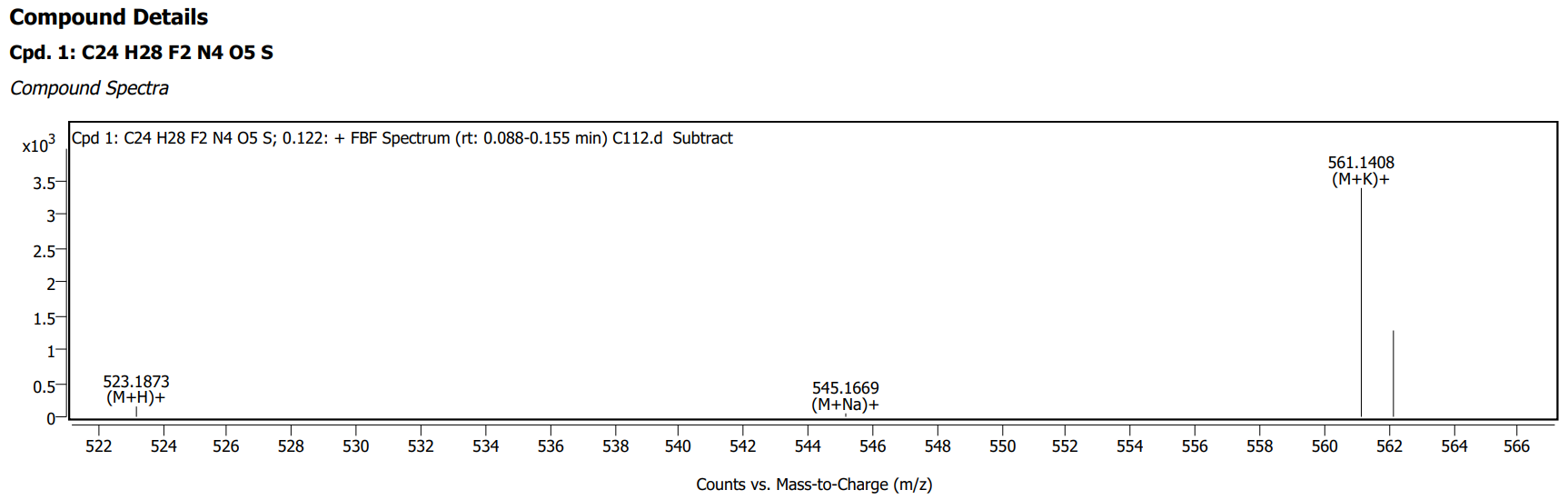


**HRMS of 3x**

**HRMS of 3y**

**Supplementary Fig. S1.** Chemical structures and enzymatic activities (measured by FRET) against SARS-CoV-2 M^pro^ for active compounds obtained in a screening campaign against our in-house chemical library.

**Supplementary Tables**

**Supplementary Table S1.** Data collection and structure refinement statistics.

|  | M^pro^-Hit-1 | M^pro^-SY110 |
| --- | --- | --- |
| **PDB code** | 8HHT | 8HHU |
| **Data collection** |  |  |
| Space group | *C2* | *C2* |
| Cell dimensions |  |  |
| *a*, *b*, *c* (Å) | 114.61, 54.16, 44.39 | 98.70, 82.41, 51.95 |
| α, β, γ (°) | 90.00, 102.10, 90.00 | 90.00, 114.68, 90.00 |
| Resolution (Å) | 48.76-1.95 (2.00-1.95) | 60.68-2.26 (2.33-2.26) |
| CC1/2 ^1^ | 0.999 (0.923) | 0.998 (0.968) |
| *R*merge^2^ | 0.046 (0.617) | 0.067 (0.619) |
| *R*meas | 0.050 (0.677) | 0.072 (0.672) |
| *R*pim ^3^ | 0.020 (0.273) | 0.028 (0.258) |
| *I* /  | 23.3 (3.0) | 14.0 (3.0) |
| Completeness (%) | 87.3 (99.6) | 98.8 (99.3) |
| Redundancy | 6.3 (5.9) | 6.8 (6.6) |
|  |  |  |
| **Refinement** |  |  |
| Resolution (Å) | 48.76-1.95 | 60.68-2.26 |
| No. reflections | 17,116 | 17,608 |
| *R*work ^4^ / *R*free | 0.196 / 0.246 | 0.185 / 0.227 |
| No. atoms |  |  |
| Protein | 2325 | 2347 |
| Ligand | 28 | 37 |
| Water | 199 | 217 |
| *B*-factors |  |  |
| Protein | 39.66 | 62.75 |
| Ligand | 44.79 | 79.51 |
| Water | 44.48 | 77.41 |
| R.m.s. deviations |  |  |
| Bond lengths (Å) | 0.009 | 0.008 |
| Bond angles (°) | 1.33 | 0.97 |
| Ramachandran plot (%)  Favored region  Allowed region  Outlier region | 98.3  1.7  0.00 | 98.7  1.3  0.00 |

^1^ CC1/2 is the correlation coefficient determined by two random half data sets^1^.

^2^ $R_{\mathrm{merge}}= \sum_{hkl} \sum_{i=1}^{n} \left| I_{i}\left( hkl \right)-\bar{I}\left( hkl \right) \right|/\sum_{hkl} \sum_{i=1}^{n} I_{i}\left( hkl \right)$.

^3^ $R_{\mathrm{pim}}= \sum_{hkl} \sqrt{1/\left( n-1 \right)}\sum_{i=1}^{n} \left| I_{i}\left( hkl \right)-\bar{I}\left( hkl \right) \right|/\sum_{hkl} \sum_{i=1}^{n} I_{i}\left( hkl \right)$^2^.

^4^ $R_{\mathrm{work}}= \sum_{hkl} \left| F_{o}\left( hkl \right)-F_{c}\left( hkl \right) \right|/\sum_{hkl} {|F}_{o}\left( hkl \right)|$. *R*free was calculated for a test set of reflections (~ 5.0%) omitted from the refinement.

**Supplementary Table S2.** Metabolic stability of test compounds in human liver microsomes (HLM)^a^.

| **Compound**^b^ | **Species** | | **Percent Remaining (%)** | | | | | | **T_1/2_**^c^ **(min)** | **CL_int_**^d^  **(mL/min/kg)** |
| --- | --- | --- | --- | --- | --- | --- | --- | --- | --- | --- |
|  |  |  | 0 min | 5 min | 15 min | 30 min | 45 min | 60 min |  |  |
| Ketanserin^e^ | human | Mean | 100.00 | 86.88 | 62.16 | 38.09 | 24.44 | 15.93 | 22.40 | 77.60 |
|  |  | RSD of Area Ratio | 0.01 | 0.00 | 0.01 | 0.03 | 0.02 | 0.02 |  |  |
| 3q | human | Mean | 100.00 | 85.97 | 63.13 | 40.06 | 30.28 | 19.07 | 25.54 | 68.06 |
|  |  | RSD of Area Ratio | 0.01 | 0.00 | 0.01 | 0.00 | 0.08 | 0.00 |  |  |
| 3r | human | Mean | 100.00 | 91.52 | 73.16 | 50.95 | 39.09 | 32.55 | 35.61 | 48.82 |
|  |  | RSD of Area Ratio | 0.00 | 0.03 | 0.01 | 0.02 | 0.03 | 0.01 |  |  |
| 3s | human | Mean | 100.00 | 81.68 | 64.18 | 41.99 | 28.40 | 18.95 | 25.42 | 68.38 |
|  |  | RSD of Area Ratio | 0.00 | 0.02 | 0.02 | 0.01 | 0.01 | 0.02 |  |  |
| 3t | human | Mean | 100.00 | 89.11 | 73.46 | 56.21 | 46.16 | 36.95 | 42.21 | 41.18 |
|  |  | RSD of Area Ratio | 0.02 | 0.01 | 0.01 | 0.01 | 0.01 | 0.03 |  |  |
| 3u | human | Mean | 100.00 | 84.12 | 64.81 | 47.02 | 39.81 | 29.04 | 35.08 | 49.55 |
|  |  | RSD of Area Ratio | 0.01 | 0.03 | 0.06 | 0.01 | 0.00 | 0.03 |  |  |
| 3v | human | Mean | 100.00 | 88.30 | 73.18 | 54.49 | 42.27 | 34.67 | 38.99 | 44.58 |
|  |  | RSD of Area Ratio | 0.01 | 0.01 | 0.02 | 0.00 | 0.00 | 0.00 |  |  |
| 3w (SY110) | human | Mean | 100.00 | 80.68 | 65.39 | 48.02 | 37.23 | 29.75 | 35.34 | 49.19 |
|  |  | RSD of Area Ratio | 0.04 | 0.00 | 0.02 | 0.02 | 0.01 | 0.02 |  |  |
| 3x | human | Mean | 100.00 | 91.06 | 71.51 | 50.53 | 34.27 | 26.49 | 30.40 | 57.18 |
|  |  | RSD of Area Ratio | 0.00 | 0.02 | 0.02 | 0.02 | 0.02 | 0.02 |  |  |
| Nirmatrelvir | human | Mean | 100.00 | 86.55 | 60.03 | 38.68 | 26.04 | 18.64 | 24.43 | 71.16 |
|  |  | RSD of Area Ratio | 0.04 | 0.02 | 0.01 | 0.00 | 0.03 | 0.02 |  |  |

^a^ Data shown represent at least two independent experiments.

^b^ Test compounds (1 μM) were examined in HLM (0.5 mg/mL protein concentration).

^c^ T_1/2_=0.693 / K (K is the rate constant from a plot of ln [concentration] vs. incubation time).

^d^ CL_int_ = (0.693 / T_1/2_) × {1 / [microsomal protein concentration (0.5 mg/mL)]} × Scaling Factors (1254.2).

^e^ Ketanserin was used as a reference compound.

**Supplementary Table S3.** Pharmacokinetic parameters of six compounds in SD rats^a^.

| **Compound** | **Admin** | **T_1/2_ (h)** | **T_max_ (h)** | **C_max_**  **(ng/mL)** | **AUC_last_**  **(h*ng/mL)** | **AUC_INF_obs_**  **(****h*ng/mL)** | **Vz_obs_**  **(L/kg)** | **CL**  **(mL/min/kg)** | **MTR_last_ (h)** | **F %** |
| --- | --- | --- | --- | --- | --- | --- | --- | --- | --- | --- |
| 3r | i.v.  (1 mg/kg) | 2.41 ± 2.24 | 0.08 ± 0.00 | 1105.29 ± 69.99 | 1011.17 ± 258.49 | 1020.76 ± 261.50 | 3.05 ± 2.08 | 16.98 ± 3.80 | 1.31 ± 0.54 | - |
|  | p.o.  (10 mg/kg) | 3.01 ± 0.91 | 2.67 ± 1.15 | 306.27 ± 32.12 | 2567.31 ± 678.74 | 2844.61 ± 326.65 | - | - | 4.89 ± 1.14 | 25.39 ± 6.71 |
| 3t | i.v.  (1 mg/kg) | 1.90 ± 0.27 | 0.08 ± 0.00 | 1317.59 ± 168.03 | 1292.28 ± 530.92 | 1329.00 ± 555.62 | 2.49 ± 1.61 | 14.52 ± 7.25 | 1.26 ± 0.42 | - |
|  | p.o.  (10 mg/kg) | 4.15 ± 1.48 | 1.67 ± 0.58 | 634.54 ± 161.39 | 6721.57 ± 2461.95 | 6903.48 ± 2619.87 | - | - | 5.90 ± 0.79 | 52.01 ± 19.05 |
| 3u | i.v.  (1 mg/kg) | 5.47 ±0.77 | 0.08 ± 0.00 | 1568.34 ± 201.27 | 2196.61 ± 634.20 | 2254.80 ± 672.40 | 3.65 ± 0.74 | 7.95 ± 2.80 | 3.19 ± 0.31 | - |
|  | p.o.  (10 mg/kg) | 5.84 ± 1.30 | 1.33 ± 0.58 | 734.80 ± 101.62 | 8180.11 ± 795.05 | 8696.48 ± 1124.53 | - | - | 6.57 ± 0.38 | 37.24 ± 3.62 |
| 3v | i.v.  (1 mg/kg) | 9.49 ± 2.16 | 0.08 ± 0.00 | 1697.71 ± 237.45 | 2574.49 ± 819.96 | 2829.72 ± 958.55 | 5.25 ± 2.13 | 6.48 ± 2.63 | 3.55 ± 0.52 | - |
|  | p.o.  (10 mg/kg) | 6.64 ± 1.17 | 4.00 ± 0.00 | 1006.49 ± 114.61 | 12675.30 ± 1265.92 | 13950.66 ± 782.95 | - | - | 7.63 ± 0.57 | 49.23 ± 4.92 |

| 3w (SY110) | i.v.  (1 mg/kg) | 4.67 ± 0.19 | 0.08 ± 0.00 | 1741.92 ± 285.19 | 2298.27 ± 587.40 | 2320.99 ± 591.73 | 3.03 ± 0.71 | 7.47 ± 1.67 | 2.34 ± 0.22 | - |
| --- | --- | --- | --- | --- | --- | --- | --- | --- | --- | --- |
|  | p.o.  (10 mg/kg) | 5.16 ± 2.94 | 4.00 ± 2.00 | 1430.18 ± 98.51 | 19018.08 ± 2192.99 | 20197.31 ± 2602.34 | - | - | 6.60 ± 0.89 | 82.75 ± 9.54 |
| 3x | i.v.  (1 mg/kg) | 5.09 ± 0.57 | 0.08 ± 0.00 | 1352.62 ± 116.10 | 2357.97 ± 638.69 | 2394.12 ± 644.94 | 3.28 ± 1.17 | 7.30 ± 1.90 | 3.18 ± 0.53 | - |
|  | p.o.  (10 mg/kg) | 4.67 ± 1.04 | 2.00 ± 0.00 | 805.87 ± 75.43 | 10791.37 ± 123.59 | 11166.54 ± 364.00 | - | - | 6.47 ± 0.50 | 45.77 ± 0.52 |

^a^ Data are shown as mean ± SD (*n* = 3).

**Supplementary Table S4.** Cytotoxicity of six compounds against selected cell lines^a^.

| **Compound** | **BEAS-2B** | **VeroE6** | **HUVEC** |
| --- | --- | --- | --- |
|  |  | **CC_50_ (μM)** |  |
| 3r | >500 | >500 | >500 |
| 3t | >500 | >500 | >500 |
| 3u | >500 | >500 | >500 |
| 3v | 158.60 ± 4.34 | 131.50 ± 19.81 | 129.20 ± 13.40 |
| 3w (SY110) | >500 | >500 | >500 |
| 3x | >500 | >500 | >500 |

^a^ Data are shown as mean ± SD, *n* = 3 biological replicates.

**Supplementary Table S5.** *In vitro* inhibition of human CYP activities by SY110 ^a^.

| **Isoform (substrate)** | **IC_50_ (μM)** |
| --- | --- |
| CYP1A2 (Phenacetin) | >30 |
| CYP2B6 (Bupropion) | >30 |
| CYP2C8 (Paclitaxel) | >30 |
| CYP2C9 (Diclofenac) | >30 |
| CYP2C19 (S-Mephenytoin) | >30 |
| CYP2D6 (Dextromethorphan) | >30 |
| CYP3A4 (Midazolam) | >30 |
| CYP3A4 (Testosterone) | >30 |

^a^ Samples were run in duplicate with *n* = 2.

**Supplementary Table S6.** Human plasma protein binding level of SY110 ^a^.

| **Compound^b^** | **Area Ratio** | | | **Fraction of Bound (%)** | **Fu (%)** |
| --- | --- | --- | --- | --- | --- |
|  | **Donor-0 h** | **Donor-5 h** | **Receiver-0 h** |  |  |
| Warfarin^c^ | 1.491 | 1.500 | 0.014 | 99.1 | 0.9 |
| SY110 | 4.235 | 3.370 | 1.013 | 69.9 | 30.1 |

^a^ Samples were run in duplicate with *n* = 2.

^b^ All compounds were tested at 1 μM.

^c^ Warfarin was used as a reference compound.

**Supplementary Table S7.** Pharmacokinetic parameters of SY110 in beagle dog, monkey and mice ^a^.

| **Compd.** | **Animal** | **Admin.** | **T_1/2_ (h)** | **T_max_ (h)** | **C_max_**  **(ng/mL)** | **AUC_last_**  **(h*ng/mL)** | **AUC_INF_obs_**  **(h*ng/mL)** | **MTR_last_**  **(h)** | **CL**  **(mL/min/kg)** | **Vz_obs_**  **(L/ kg)** | **F %** |
| --- | --- | --- | --- | --- | --- | --- | --- | --- | --- | --- | --- |
| **SY110** | Beagle Dog | i.v.  (1 mg/kg) | 0.61 ± 0.05 | 0.083 ± 0.00 | 1270.63 ± 332.60 | 623.96 ± 113.01 | 627.67 ± 113.38 | 0.47 ± 0.05 | 1625.21 ± 265.88 | 1419.64 ± 160.26 | - |
|  |  | p.o.  (10 mg/kg) | 5.59 ± 1.24 | 1.00 ± 0.00 | 3061.33 ± 136.00 | 8235.92 ± 370.33 | 8290.10 ± 377.59 | 2.14 ± 0.18 | - | - | 131.99 ± 5.94 |
|  | Monkey | i.v.  (1 mg/kg) | 0.73 ± 0.20 | 0.08 ± 0.00 | 567.18 ± 38.24 | 501.29 ± 59.91 | 505.62 ± 60.72 | 0.90 ± 0.18 | 1998.35 ± 257.17 | 2065.80 ± 374.39 | - |
|  |  | p.o.  (10 mg/kg) | 5.57 ± 1.05 | 2.33 ± 1.53 | 242.48 ± 54.21 | 1221.18 ± 56.84 | 1264.93 ± 72.03 | 4.71 ± 0.89 |  |  | 24.36 ± 1.13 |
|  | ICR Mice | i.v.  (1 mg/kg) | 0.27 ± 0.17 | 0.08 ± 0.00 | 285.09 ± 16.35 | 85.90 ± 7.60 | 87.12 ± 7.81 | 0.17 ± 0.03 | 192.28 ± 16.45 | 4.27 ± 2.29 | - |
|  |  | p.o.  (150 mg/kg) | 3.25 ± 1.03 | 0.33 ± 0.14 | 4078.10 ± 636.18 | 15134.02 ± 5136.94 | 15692.57 ± 4424.87 | 3.62 ± 1.18 | - | - | 67.42 ± 22.88 |

^a^ Data are shown as mean ± SD (*n* = 3).

**Supplementary Table S8.** Inhibitory activities of SY110 against several common mammalian proteases ^a^.

| **Protease** | **IC_50_ (μM)** |
| --- | --- |
| Human Thrombin | >100 |
| Human Cathepsin K | >100 |
| Human Cathepsin B | >100 |
| Human Caspase 2 | >100 |
| Human Cathepsin D | >100 |
| Neutrophil Elastase | >100 |
| Human Cathepsin L | >100 |

^a^ Data are shown as mean ± SD, *n* = 3 biological replicates.

**Supplementary Table S9.** *In vivo* toxicity study of SY110 in mice.

| **Study** | **Acute toxicity study** | | **Repeated dose two-week toxicity study** | |
| --- | --- | --- | --- | --- |
| **Compound** | SY110 | | | |
| **Administration** | p.o. | | | |
| **Dosage** | 1000 mg/kg | 800 mg/kg | 600 mg/kg | 400 mg/kg |
| **Number of animals** | 6 mice per group | | | |
| **Frequency** | Single dose | | Twice daily | |
| **Results** | No obvious toxicity  1. No mice died after receiving compounds.  2. No abnormalities in body weights and general status were observed in each group during the period of observation.  3. No obvious organic damage was observed in heart, liver, lung, kidney and spleen in each group at the end of observation. | | | |

**Supplementary Table S10.** Effects of SY110 by oral gavage on maternal data, cesarean section observation and fetal examination data in rat embryo-fetal development studies ^a^.

| Indicator | Dose (mg/kg) | | | |
| --- | --- | --- | --- | --- |
|  | 0 | 100 | 300 | 1000 |
| **Maternal data** | | | | |
| Number of Pregnant Dams | 7 | 6 | 7 | 5 |
| Maternal Body Weight (GD^b^ 20, g) | 455.2 ± 20.97 | 459.8 ± 35.07 | 465.3 ± 24.73 | 470.2 ± 37.26 |
| Maternal Body Weight Gain (GD^b^ 6−20, g) | 51.6 ± 14.3 | 46.2 ± 12.8 | 50.8 ± 8.7 | 52.0 ± 12.5 |
| **Cesarean section observation** | | | | |
| Corpora lutea | 17.4 ± 2.0 | 16.2 ± 2.1 | 17.7 ± 1.5 | 17.4 ± 2.5 |
| Implantation sites | 16.4 ± 1.6 | 15.0 ± 3.5 | 16.9 ± 1.5 | 17.0 ± 2.5 |
| Resorptions | 0.4 ± 0.8 | 0.5 ± 0.8 | 0.6 ± 0.5 | 0.6 ± 0.9 |
| Live embryos | 16.0 ± 1.2 | 14.5 ± 3.6 | 16.3 ± 1.6 | 16.4 ± 2.1 |
| **Fetal examination data** | | | | |
| **Fetal appearance** | | | | |
| Number Examined | 16.0 ± 1.2 | 14.5 ± 3.6 | 16.3 ± 1.6 | 16.4 ± 2.1 |
| Sex ratio (% male) | 41 ± 9.9 | 50 ± 13.8 | 47 ± 10.0 | 51 ± 19.0 |
| Fetal weight (g)/Litter | 4.082 ± 0.2461 | 4.230 ± 0.9638 | 4.175 ± 0.3757 | 4.027 ± 0.1891 |
| appearance malformation rate (%) | 0.0 ± 0.0 | 0.0 ± 0.0 | 0.0 ± 0.0 | 0.0 ± 0.0 |
| **Visceral** | | | | |
| Number Examined | 8.3 ± 0.5 | N.D. | N.D. | 8.4 ± 1.1 |
| Visceral malformation rate (%) | 3.6 ± 6.1 | N.D. | N.D. | 0.0 ± 0.0 |
| Abnormal position of umbilical artery | 1.8 ± 4.7 | N.D. | N.D. | 0.0 ± 0.0 |
| Absent of aortic arch | 1.8 ± 4.7 | N.D. | N.D. | 0.0 ± 0.0 |
| Absent of innominate artery | 1.8 ± 4.7 | N.D. | N.D. | 0.0 ± 0.0 |
| Abnormal initial position of common carotid artery | 1.8 ± 4.7 | N.D. | N.D. | 0.0 ± 0.0 |
| Abnormal initial position of subclavian artery | 1.8 ± 4.7 | N.D. | N.D. | 0.0 ± 0.0 |
| **Skeletal** | | | | |
| Number Examined | 7.7 ± 0.8 | N.D. | N.D. | 8.0 ± 1.0 |
| Sternebra | | | | |
| Unossified #5 and/or 6 – [V]^c^ | 7.1 ± 12.2 | N.D. | N.D. | 7.9 ± 7.4 |
| Incomplete ossification #5 and/or 6 – [V] ^c^ | 5.4 ± 6.8 | N.D. | N.D. | 2.2 ± 5.0 |
| Split #5 and/or 6– [M] ^c^ | 0.0 ± 0.0 | N.D. | N.D. | 2.9 ± 6.4 |
| Rib | | | | |
| 14 Ribs – [V] ^c^ | 0.0 ± 0.0 | N.D. | N.D. | 6.9 ± 10.1 |
| Shortened #13 – [V] ^c^ | 0.0 ± 0.0 | N.D. | N.D. | 2.9 ± 6.4 |
| Skull | | | | |
| Interparietal bone, incomplete ossification – [V] ^c^ | 1.8 ± 4.7 | N.D. | N.D. | 0.0 ± 0.0 |
| Parietal bone, incomplete ossification – [V] ^c^ | 3.6 ± 9.4 | N.D. | N.D. | 0.0 ± 0.0 |

^a^ Data are shown as mean ± SD.

^b^ GD = Gestation Day.

^c^ [M] = Malformation; [V] = Variation.

**Supplementary Table S11.** Primers used in site-directed mutagenesis PCR for each M^pro^ mutants.

| **Mutant** | **Sequence*** |
| --- | --- |
| P132H | 5' - GCAATGCGTC***AT***AATTTTACCATTAAGGGTAGTTTTCTG - 3' |
|  | 5' - GGTAAAATT***AT***GACGCATTGCACACTGATAAACGCC - 3' |
| Q192S | 5' – CAGACCGCA***AGT***GCCGCAGGCACCGATACCACCATTAC - 3' |
|  | 5' – TGCCTGCGGC***ACT***TGCGGTCTGGCGATCAACAAACG - 3' |
| Q192T | 5' – CAGACCGCA***ACC***GCCGCAGGCACCGATACCACCATTAC - 3' |
|  | 5' – TGCCTGCGGC***GGT***TGCGGTCTGGCGATCAACAAACG - 3' |
| Q192V | 5' – CAGACCGCA***GTG***GCCGCAGGCACCGATACCACCATTAC - 3' |
|  | 5' – TGCCTGCGGC***CAC***TGCGGTCTGGCGATCAACAAACG - 3' |
| M165T | 5' – TATGCATCAT***ACC***GAACTGCCGACCGGCGTTCATGC - 3' |
|  | 5' – TCGGCAGTTC***GGT***ATGATGCATATAGCAGAAGCTCAC - 3' |
| H172F | 5' – CCGACCGGCGTT***TTC***GCCGGCACCGATCTGGAAGGTAATT - 3' |
|  | 5' – CGGTGCCGGC***GAA***AACGCCGGTCGGCAGTTCCATATGATGC -3' |
| H172Q | 5' – GACCGGCGTT***CAG***GCCGGCACCGATCTGGAAGG - 3' |
|  | 5' – CGGTGCCGGC***CTG***AACGCCGGTCGGCAGTTCCATATG - 3' |
| L50F | 5' – CGAAGATATG***TTC***AATCCGAATTATGAAGATCTGCTG - 3' |
|  | 5' – AATTCGGATT***GAA***CATATCTTCGCTGGTACAAATAAC - 3' |
| E166A &  L167F | 5' – GCATCATATG***GCATTC***CCGACCGGCGTTCATGCCGGCAC - 3' |
|  | 5' – GCCGGTCGG***GAATGC***CATATGATGCATATAGCAGAAGCT - 3' |
| E166N | 5' – ATCATATG***AAT***CTGCCGACCGGCGTTCATGCCGGCACCG - 3' |
|  | 5' – CGGTCGGCAG***ATT***CATATGATGCATATAGCAGAAGCTCAC - 3' |
| H172Y | 5' – GGCGTT***TAT***GCCGGCACCGATCTGGAAGGTAATTTTTATG - 3' |
|  | 5' – GGTGCCGGC***ATA***AACGCCGGTCGGCAGTTCCATATGATG - 3' |
| E166V | 5' – ATCATATG***GTG***CTGCCGACCGGCGTTCATGCCGGCACCG - 3' |
|  | 5' – CGGTCGGCAG***CAC***CATATGATGCATATAGCAGAAGCTCAC - 3' |
| G143S | 5' – TTCTGAAC***AGC***AGTTGCGGTAGCGTTGGTTTTAATATTG - 3' |
|  | 5' – GCAACT***GCT***GTTCAGAAAACTACCCTTAATGGTAAAATTC - 3' |
| Q189E | 5' – TGATCGC***GAA***ACCGCACAGGCCGCAGGCACCGATACCAC - 3' |
|  | 5' – GTGCGGT***TTC***GCGATCAACAAACGGGCCATAAAAATTAC - 3' |
| △P168 | 5' – TATGGAACTG*ACCGGCGTTCATGCCGGCACCGATCTG - 3' |
|  | 5' - ATGAACGCCGGT*CAGTTCCATATGATGCATATAGCAG - 3' |
| A173V | 5' – GCGTTCAT***GTT***GGCACCGATCTGGAAGGTAATTTTTATGG - 3' |
|  | 5' – CGGTGCC***AAC***ATGAACGCCGGTCGGCAGTTCCATATGATG - 3' |

**Supplementary References**

1. Karplus P. A., & Diederichs, K. Linking crystallographic model and data quality. *Science* **336**, 1030-1033 (2012).
2. Weiss, M. S., & Hilgenfeld, R. On the use of the merging R factor as a quality indicator for X-ray data. *J. Appl. Crystallogr.* **30**, 203-205 (1997).
